# A comprehensive collection of systems biology data characterizing the host response to viral infection

**DOI:** 10.1038/sdata.2014.33

**Published:** 2014-10-14

**Authors:** Brian D. Aevermann, Brett E. Pickett, Sanjeev Kumar, Edward B. Klem, Sudhakar Agnihothram, Peter S. Askovich, Armand Bankhead, Meagen Bolles, Victoria Carter, Jean Chang, Therese R.W. Clauss, Pradyot Dash, Alan H. Diercks, Amie J. Eisfeld, Amy Ellis, Shufang Fan, Martin T. Ferris, Lisa E. Gralinski, Richard R. Green, Marina A. Gritsenko, Masato Hatta, Robert A. Heegel, Jon M. Jacobs, Sophia Jeng, Laurence Josset, Shari M. Kaiser, Sara Kelly, G. Lynn Law, Chengjun Li, Jiangning Li, Casey Long, Maria L. Luna, Melissa Matzke, Jason McDermott, Vineet Menachery, Thomas O. Metz, Hugh Mitchell, Matthew E. Monroe, Garnet Navarro, Gabriele Neumann, Rebecca L. Podyminogin, Samuel O. Purvine, Carrie M. Rosenberger, Catherine J. Sanders, Athena A. Schepmoes, Anil K. Shukla, Amy Sims, Pavel Sova, Vincent C. Tam, Nicolas Tchitchek, Paul G. Thomas, Susan C. Tilton, Allison Totura, Jing Wang, Bobbie-Jo Webb-Robertson, Ji Wen, Jeffrey M. Weiss, Feng Yang, Boyd Yount, Qibin Zhang, Shannon McWeeney, Richard D. Smith, Katrina M. Waters, Yoshihiro Kawaoka, Ralph Baric, Alan Aderem, Michael G. Katze, Richard H. Scheuermann

**Affiliations:** 1 J. Craig Venter Institute, La Jolla, CA 92037, USA; 2 Northrop Grumman Information Systems, Health IT, Rockville, MD 20850, USA; 3 Department of Epidemiology, University of North Carolina at Chapel Hill, Chapel Hill, NC 27599-7400, USA; 4 Seattle Biomedical Research Institute, Seattle, WA 98109, USA; 5 Oregon Clinical & Translational Research Institute, Portland, Oregon 97239-3098, USA; 6 Division of Bioinformatics and Computational Biology, Department of Medical Informatics and Clinical Epidemiology, Oregon Health Sciences University, Portland, Oregon 97239-3098, USA; 7 Department of Microbiology and Immunology, University of North Carolina at Chapel Hill, Chapel Hill, North Carolina 27599-7290, USA; 8 Department of Microbiology, University of Washington, Seattle, WA 98195, USA; 9 Biological Sciences Division, Pacific Northwest National Laboratory, Richland, WA 99352, USA; 10 Department of Immunology, St. Jude Children’s Research Hospital, Memphis, TN 38105-3678, USA; 11 School of Veterinary Medicine, Department of Pathobiological Sciences, Influenza Research Institute, University of Wisconsin-Madison, Madison, WI 53706, USA; 12 Department of Genetics, University of North Carolina at Chapel Hill, Chapel Hill, NC 27599-7264, USA; 13 Division of Animal influenza, Harbin Veterinary Research Institute, Chinese Academy of Agricultural Sciences, Harbin, Heilongjiang Province 150001, China; 14 Environmental Molecular Sciences Laboratory, Pacific Northwest National Laboratory, Richland, WA 99354, USA; 15 Washington National Primate Research Center, University of Washington, Seattle, WA 98195, USA; 16 Department of Pathology, University of California, San Diego, CA 92093, USA

## Abstract

The Systems Biology for Infectious Diseases Research program was established by the U.S. National Institute of Allergy and Infectious Diseases to investigate host-pathogen interactions at a systems level. This program generated 47 transcriptomic and proteomic datasets from 30 studies that investigate *in vivo* and *in vitro* host responses to viral infections. Human pathogens in the *Orthomyxoviridae* and *Coronaviridae* families, especially pandemic H1N1 and avian H5N1 influenza A viruses and severe acute respiratory syndrome coronavirus (SARS-CoV), were investigated. Study validation was demonstrated via experimental quality control measures and meta-analysis of independent experiments performed under similar conditions. Primary assay results are archived at the GEO and PeptideAtlas public repositories, while processed statistical results together with standardized metadata are publically available at the Influenza Research Database (www.fludb.org) and the Virus Pathogen Resource (www.viprbrc.org). By comparing data from mutant versus wild-type virus and host strains, RNA versus protein differential expression, and infection with genetically similar strains, these data can be used to further investigate genetic and physiological determinants of host responses to viral infection.

## Background & Summary

With the recognition that host responses to pathogen infection play key roles in disease severity and mortality, virologists have shifted toward integrated systems biology approaches to identify therapeutics that target host pathways^1,2^. To support the cross-disciplinary approaches necessary to address a systems-level analysis, the Division of Microbiology and Infectious Diseases (DMID) at the National Institute of Allergy and Infectious Disease (NIAID) established the Systems Biology for Infectious Diseases Research (SysBio) program that provided support for four centers from 2008–2013: Systems Influenza, Systems Virology, Systems Biology of Enteropathogens, and Mycobacterium tuberculosis Systems Biology^3^. The Systems Influenza and Systems Virology centers focused on elucidating the mechanisms of how viral regulation of the host cellular circuitry contributes to viral replication and disease severity, thereby elucidating host pathways that could serve as potential new therapeutic targets.

Several publications have reported the findings from these virology-focused SysBio centers. Overall characterization of the host responses demonstrated that pro-inflammatory interferon (IFN) signalling pathways were enriched following infection with either influenza A or Betacoronavirus infection *in vitro*^4,5^ and *in vivo*^6,7^. These studies characterized several aspects of viral-host interactions that influence disease severity. Experiments designed to tease out the contribution of the virus and the host to the severity of host response reported that specific mutations in the H5N1 viral genome affected the kinetics and magnitude of the host response, rather than changes in specific host response factors^8^. Another study found that early response signatures to influenza virus infection correlated with the severity of late disease^9^. Lipidomics analysis of mice infected with various influenza A virus strains of varying pathogenicity have also been reported^10^. Similarly, the disease severity of SARS-CoV correlated with the dysregulation of the urokinase pathway and an increase in fibrinolysin pathway activity^7^. An integrated network interrogation approach using both transcriptomic and proteomic data sets predicted a subset of key regulator molecules responding to SARS-CoV and influenza infections that could be used as host targets for therapeutic intervention^11,12^. Genetic modification of non-structural protein 16 in SARS-CoV showed enhanced susceptibility to type I and III interferon responses, making it a good vaccine strain candidate^13^.

To support the broad dissemination of research datasets from these SysBio centers and other supported programs, DMID established the Bioinformatics Resource Centers for Infectious Diseases (BRC) program to provide public database and analysis resources for the infectious disease research community. A data dissemination working group, consisting of representatives from NIAID, the SysBio centers, and the BRCs was established to develop a data management and release approach that would maximize the reuse potential of the generated data. The working group leveraged existing public archiving strategies to store the primary assay results and devised metadata standards to represent the experiment descriptions and interpreted results in a consistent and standardized way. The final derived host response data and associated structured standardized metadata were made publically available at the BRC appropriate for the system under study. As the virus-centric BRCs, the Influenza Research Database (IRD, www.fludb.org)^14^ and the Virus Pathogen Database and Analysis Resource (ViPR, www.viprbrc.org)^15^ host data produced by the Systems Influenza and Systems Virology centers.

Here we describe 47 transcriptomic and proteomic datasets from 30 studies generated by these SysBio centers and made available through public resources. These studies characterized the host response to infection by members of the *Orthomyxoviridae* and *Coronaviridae* virus families, including pandemic influenza A H1N1 virus, highly pathogenic H5N1 avian influenza (HPAI) virus, severe acute respiratory syndrome coronavirus (SARS-CoV), and Middle East respiratory syndrome coronavirus (MERS-CoV); all of which are capable of causing severe respiratory infections and pose significant threats to humans on a global scale^4,16^. Host responses were evaluated in either *in vivo* (mouse) or *in vitro* (human cell line) model systems using three major experimental designs: longitudinal time-course, dose response, and genetic modification (involving both genetically manipulated viruses and hosts) comparisons. Host responses to these manipulated variables were assessed using transcriptomic (gene expression microarray) and proteomic (mass spectrometry) assays. Together, this collection represents the first coordinated effort to create a systems level description of host-pathogen interactions using multiple viral strains, host models, and ‘-omics’ technologies.

## Methods

**Figure 1:**
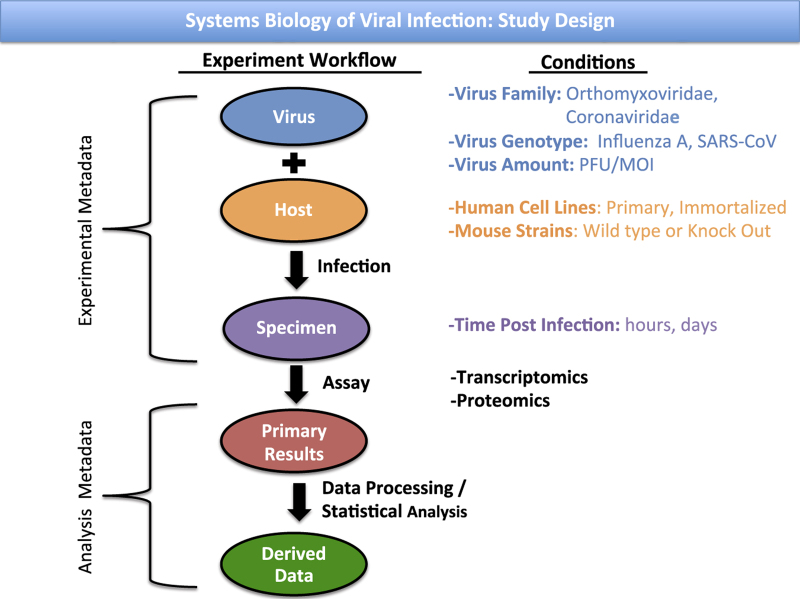
Overall study design for the study of the systems biology of viral infection. The ovals down the center represent steps in the experimental workflow common to all datasets. Text on the left gives the type of metadata required to describe the components of the workflow. On the right lists the experiment conditions investigated at each step throughout these datasets.

The experimental designs reported here include longitudinal (i.e. time course), dose response, and genetic modification of both virus and host strain comparisons. The intent of these designs was to assemble a unified view of the virulence mechanisms and replication strategies resulting from viral regulation of host cellular processes. For the present experiments, either transcriptomic (using gene expression microarrays) or proteomic (using liquid chromatography coupled with mass spectrometry) methodologies were used to collect the reported data. The materials used, sample preparation protocols, validation procedures, data processing, and hypothesis testing that were performed are described below. Throughout the Methods section, Study IDs are appended to indicate which studies used a given method. A summary of the overall experiment workflow and the experiment factor conditions that were compared is presented in Fig. 1. The relationship between the experiment workflow and the experimental metadata, analysis metadata, primary assay results, and derived data is also presented. Study designs for all experiments, including relevant repository identifiers, are summarized in Table 1 (available online only), with individual RNA experiment samples detailed in Table 2 (available online only) and individual protein experiment samples detailed in Table 3 (available online only). Sample tracking from animal subjects to experiment samples is given in Table 4 (available online only).

### Viruses

Within the studies reported here, the host response to viral agents from two major viral families, Orthomyxoviridae and Coronaviridae, are characterized. From Orthomyxoviridae, two influenza A subtypes H5N1 and H1N1 were chosen in order to investigate influenza viruses with higher and lower reported pathogenicity, respectively. Highly Pathogenic Avian Influenza H5N1 (HPAI) has been commonly referred to as ‘Avian Flu’ as it is endemic in bird populations. HPAI is known to exhibit ‘stuttering transmission’ in humans, with a relatively high mortality rates (>50%), but to-date has not acquired efficient human-to-human transmission. This high mortality rate together with its pathological similarities to the 1918 H1N1 ‘Spanish Flu’ make it an important model to study and prevent future lethal pandemics. On the other hand, the more recent seasonal and pandemic H1N1 strains are considered to have lower pathogenicity, rarely causing severe disease or death. Since its emergence, pandemic H1N1 has been found to recur in the human population as a seasonal influenza. H1N1 influenza isolates used in these studies include recent pandemic strains, previous seasonal strains, and a reconstructed 1918 strain.

Virus preparations for the Influenza A/Vietnam/1203/2004 (H5N1) (abbreviated as VN1203) isolate were derived from a plasmid-based reverse-genetic system as previously described^17,18^. Four A/Viet Nam/1203/2004 mutant viruses that contain modified pathogenicity determinants were similarly generated by reverse genetics coupled with site-directed mutagenesis^17–19^, including H5N1 VN1203-HAavir in which the hemagglutinin (HA) surface protein poly-basic cleavage site was mutated^20^; H5N1 VN1203-PB2627E which possesses a K to E amino acid substitution at residue 627 of the PB2 protein^21^; H5N1 VN1203-NS1trunc, in which a stop codon was introduced at amino acid 124 of the NS1 protein open reading frame^8^; and H5N1 VN1203-PB1F2del, in which the PB1-F2 open reading frame was eliminated^8^. Additional natural influenza strains used in these studies include: A/California/04/2009 (H1N1) (abbreviated as CA04), A/Netherlands/602/2009 (H1N1) (abbreviated as NL602; kindly provided by Ron Fouchier, Erasmus Medical Center, Rotterdam, Netherlands)^22^, A/Mexico/4482/09 (H1N1) (Mex/4482), A/New Jersey/8/1976 (H1N1) (NJ/8/76), and A/Brisbane/59/07 (H1N1) (Brisbane/59/07)^6^. The reconstructed r1918 strain was also included in one study^23^. All virus strains were propagated and amplified in Madin-Darby canine kidney (MDCK) cells at 37**°** C in an atmosphere of 5% CO_2_, except NJ/8/76, which was propagated in 10-day-old embryonated hens’ eggs^6^. (CA04M001, ICL004, ICL006, ICL010, ICL011, ICL012, IM001, IM002, IM004, IM005, IM006A, IM006B, IM007, IM009, IM010, SBRI_AA_E1).

The PR8, X31, and VN1203(6+2) influenza A viruses were generated using the 8-plasmid influenza reverse genetics system^24^. All three viruses are based on PR8 (A/Puerto Rico/8/34), a mouse-adapted H1N1 strain originally derived from a human isolate. X31 is a mouse adapted H3N2 strain with the 6 internal genes of PR8 and the HA and NA derived genes from A/Aichi/2/1968. rgVN1203(6+2) (VN) contains the 6 internal genes of PR8 and the H5N1 HA and NA genes from A/Vietnam/1203/2004 with the polybasic cleavage site of the HA modified to restrict its replication to the airway^25,26^. (SBRI_AA_E1).

In addition to the influenza A strains, the studies reported here investigate recently characterized Coronaviridae strains. These strains are all members of the betacoronavirus genus including Severe Acute Respiratory Syndrome (SARS-CoV) and Middle Eastern Respiratory Syndrome (MERS-CoV) Coronaviruses. Both viruses cause severe acute respiratory illness, with SARS-CoV and MERS-CoV infection resulting in an overall mortality rate of ~10% and 38%, respectively. SARS-CoV was marked by its ease of transmissibility between humans, while MERS-CoV appears to be more limited in its human transmissibility. These viruses represent emerging pathogens not formerly recognized as a threat to human health, which adds an aspect of urgency to their research and characterization.

The SARS-CoV virus preparations used in these studies include a wild-type infectious clone derived from SARS-CoV (abbreviated icSARS-CoV), icSARS-CoV dORF6 in which accessory ORF6 was deleted, and Bat-SRBD, a reconstruction from a Bat-SCoV consensus genome with SARS-CoV receptor-binding domain (RBD)^27^. These strains were obtained from the Baric laboratory’s existing infectious clone constructs as previously described^28–30^. Recombinant mouse-adapted SARS-CoV (MA15)^31^, SARS-CoV dNSP16 (ref. 13), and human coronavirus Middle East Respiratory Syndrome Coronavirus strain EMC 2012 (abbreviated MERS-CoV) (from Bart L. Haagmans Erasmus Medical Center, Rotterdam, Netherlands) were also evaluated. All strains belonging to the *Betacoronavirus* genus were propagated in Vero E6 cells. For more details, see virus strain descriptions given in Table 5 (available online only). (ECL001, SCL005, SCL006, SHAE002, SHAE003, SHAE004, SM001, SM003, SM004, SM007, SM009, SM012, SM014, SM015, SM019, SM020).

### Human cell lines, infections, and extractions

Most cell line studies involving infection with influenza A viruses were conducted in Calu-3 cells, a human lung adenocarcinoma cell line (kindly provided by Raymond Pickles; University of North Carolina, Chapel Hill, NC, USA). Cells were maintained in a 1:1 mixture of Dulbecco’s modified Eagle’s medium and Ham’s F12 nutrient medium (DF12; Invitrogen, Carlsbad, CA) supplemented with 10% fetal bovine serum. For infection, Calu-3 cells were plated in 6 well plates at 1x10^6^ cells per well. Cells were washed after 24 hours and infected 48 hours after plating. Infections were performed by removing culture medium, washing cells twice with 1x PBS, and inoculating each well with 300 μl of DF12 supplemented with 0.3% bovine serum albumin (DF12-BSA) containing virus at a multiplicity of infection of 1 to 3 plaque forming units per cell. Cultures were mock-infected by inoculating with DF12-BSA lacking virus. Cells were then incubated for 40 min at 37**°** C, and after the inoculum was removed, cells were washed with 1x PBS and cultured in 2 mL of DF12-BSA containing L-(tosylamido-2-phenyl) ethyl chloromethyl ketone (TPCK)-treated trypsin. At each sample collection time point, aliquots of medium were reserved for plaque assays (see below). Time zero samples were immediately harvested using the appropriate procedure depending on the assay to be performed.

Studies examining members of the *Betacoronavirus* genus were performed in a clonal population of Calu-3 cells sorted for high levels of expression of the SARS-CoV cellular receptor angiotensin-converting enzyme 2 (ACE2), referred to as Calu-3 2B4 cells (kindly provided by Chien K. Tseng, University of Texas Medical Branch, Galveston, TX)^32^. Cells were maintained in 1x MEM (Gibco catalog number 11095) supplemented with defined Fetal Bovine Serum (HyClone catalog number SH30070.03) and antibiotic/antimycotic (100x Gibco catalog number 15240). Infections of Calu-3 2B4 cells were performed in a similar manner to the above Calu-3 cells. In the case of Calu-3 2B4 cells, 1.5×10^6^ cells per well were seeded with 3 mL of complete culture medium. Culture medium was refreshed after 24 hours and infections administered after 48 hours as above. (SCL005, SCL006).

A subset of studies involving infection with influenza A virus and members of the *Betacoronavirus* genus were performed in human airway epithelial (HAE) cells, which were obtained from airway specimens resected from patients undergoing surgery under University of North Carolina Institutional Review Board, with approved protocols by the Cystic Fibrosis Center Tissue Culture Core. Primary cells were plated at a density of 2.5x10^5^ cells per well on permeable Transwell-COL (12-mm-diameter) supports. HAE cultures were generated over 6 to 8 weeks, forming well-differentiated polarized cultures that resembled *in vivo* pseudostratified mucociliary epithelium^33^. Infection of HAE cultures began by washing with 1x PBS followed by challenge with 200 μL of either viral agent or mock to the apical surface. Cultures were incubated at 37**°** C for 2 hours and the inoculum and unbound viruses were removed by washing three times with 1x PBS. Apical wash samples were harvested to analyze viral growth kinetics and titers by plaque assay in Vero E6 cells (see below). (SHAE002, SHAE003, SHAE004).

Isolation of RNA from cell cultures was performed as follows: cell monolayers were first washed with 5 mL of cold PBS and then lysed directly in the culture dish by adding 1 mL of TRIZOL reagent. Cells were pipetted up and down until cells had a uniform color and consistency. They were then transferred to a 2 ml polypropylene tube (O-ring, screw cap), vortexed thoroughly, and incubated for 5 minutes at room temperature. Lysates were flash frozen on dry ice and stored at −80**°** C until shipping on dry ice. (ICL004, ICL006, ICL010, ICL011, ICL012, ECL001, SCL005, SCL006, SHAE002, SHAE003, SHAE004).

Isolation of protein from cell cultures was performed as follows: culture media was removed and cells were washed with 1 mL cold 150 mM (pH 8.5) ammonium bicarbonate buffer. This process was repeated for a total of 3 washes. 300 μL of cold 8 M urea dissolved in 50 mM ammonium bicarbonate buffer [pH 7.8] was then added directly to the well. Cells were scraped into a pre-sterilized 2 ml siliconized tube and incubated at room temperature for 15 minutes minutes to inactive viruses. Samples were then frozen in liquid nitrogen or dry ice/ethanol bath and stored at −80 °C until further processing. (ICL004, ICL006, ICL010, ICL011, ICL012, SCL005, SCL006).

### Animal strains, infections, and extractions

The following mouse strains were obtained from the Jackson Laboratory (Bar Harbor, ME, USA): C57BL/6 J (stock no. 000664), Serpine1^−/−^ (stock no. 002507), PLAT^−/−^ (stock no. 002508), B6;129S6-*Ppp1r14c*^*tm1Uhl*^/J (stock no. 013041), B6.129S2-*Tnfrsf1b*^*tm1Mwm*^/J (stock no. 002620), B6.129S7-*Tnfrsf1b*^*tm1Imx*^/J (stock no. 003243), B6.129S4-*Timp1*^*tm1Pds*^/J (stock no. 006243), B6.129P2-*Cxcr3*^*tm1Dgen*^/J (stock no. 005796), and B6.129-Ido1^tm1Alm^/J (stock no. 005867). Young female BALB/c (Six-to eight-week-old) mice were purchased from Charles River Laboratories (Wilmington, MA, USA). All animal housing, care and treatment were conducted in accordance with the University of Wisconsin-Madison School of Veterinary Medicine Animal Care and Use Committee, or by the University of North Carolina Institutional Animal Care and Use Committee or under the guidance of the CDC's Institutional Animal Care and Use Committee in an animal facility accredited by the Association for Assessment and Accreditation of Laboratory Animal Care International. For virus infections, mice were anesthetized by isofluorane inhalation (VN1203) or lightly anesthetized with ketamine/xylazine (SARS-CoV and CA04) and intranasally inoculated with either 50 μL of phosphate-buffered saline (PBS) alone (mock infection), or PBS containing a viral agent. Individual or group mouse body weights were collected on a daily basis to monitor the disease course, and mice were humanely euthanized upon reaching the experimental endpoint (i.e. sample collection or severe clinical symptoms). Weight loss significance was determined by Student’s *t* test (Microsoft Excel), and significance in survival data was determined by the Mantel-Cox test (GraphPad). Both viral titers and qPCR assays were used to confirm the infection status of all mice (see below). For more details, see animal strain and cell line descriptions given in Table 6 (available online only).

To prevent RNA degradation, immediately after dissection, lung tissues were directly submerged in RNAlater stabilization solution (Ambion, Catalog number AM7021) following manufacturer’s recommendations. Specifically, tissue was cut into small chunks (<0.5 cm in any single dimension) and placed immediately into 10-20 volumes (w/v) of RNAlater. Samples were thoroughly immersed in RNAlater solution and incubated overnight at 4**°** C, followed by freezing at −80**°** C.

Homogenization for later RNA isolation was performed as follows. Lung tissues were thawed, removed from RNAlater, dabbed on a kimwipe to remove excess liquid, transferred to 1 ml TRIzol and homogenized using a TissueLyser or MagnaLyser using several 30 second bursts to prevent overheating of the sample. Samples were transferred to a clean microfuge tube and stored at −80**°** C until RNA isolation. (CA04M001, IM001, IM002, IM004, IM005, IM006A, IM006B, IM007, IM009, IM010, SM001, SM003, SM004, SM007, SM009, SM012, SM014, SM015, SM019, SM020).

Homogenization of tissue for protein isolation was performed as follows: lung tissue were rinsed in 50 mM ammonium bicarbonate to remove blood and other fluid, then frozen at −80 °C and stored until the next step. The tissue was homogenized in 2 ml of 8 M urea dissolved in 50 mM ammonium bicarbonate buffer [pH 7.8]. The lung homogenates were then centrifuged at low speed (e.g. 5000xg) to remove large pieces of debris. The supernatants were transferred to new 2 mL siliconized microcentrifuge tubes, and incubated at room temperature for 1 hour to inactivate viruses. Samples were then frozen in liquid nitrogen or dry ice/ethanol bath and stored at −80 °C until further processing. (CA04M001, IM001, IM002, IM004, IM005, IM006A, IM006B, IM007, SM001).

Alternatively, mice were anesthetized with 350 μL of Avertin (2, 2, 2-Tribromoethanol) administered intraperitoneally. The virus inoculum was administered intranasally in a total volume of 30 μL (10^5^ PFU of virus in 1x PBS). After infection, mice were kept on a heating pad until they regained consciousness. At 12 hours following infection, mice were sacrificed by CO_2_ inhalation. A hole was clipped in the trachea and bronchoalveolar fluid (BALF) collected. Lungs were perfused three times with 1 mL cold 1x PBS, for total of 3 mL. Whole lungs were then removed and homogenized in 1 mL TRIzol. (SBRI_AA_E1).

### Viral titers and infection confirmation

Influenza viral titers were determined by standard plaque assay^34^. Briefly, MDCK cells were plated in 6-well plates, washed twice and then inoculated with 100 μL of 10-fold diluted virus in minimum essential medium supplemented with 0.3% BSA (MEM-BSA). Cells were incubated at room temperature for 1 hour, after which the inoculation solution was then removed. Cells were washed three times with PBS and overlaid with 1% Seaplaque agarose in MEM-BSA, supplemented with TPCK-treated trypsin (final 0.5 μg/mL). After the agarose solidified, plates were incubated upside down at 37**°** C and 5% CO_2_ for 48 hours. To visualize and count plaques, all wells were fixed with 10% phosphate-buffered formalin overnight, after which the agarose plugs were removed and plates were dried. (CA04M001, ICL004, ICL006, ICL010, ICL011, ICL012, IM001, IM002, IM004, IM005, IM006A, IM006B, IM007, IM009, IM010, SBRI_AA_E1).

Plaque assays for members of the *Betacoronavirus* genus were performed on supernatants collected at various times post-infection. Briefly, 5x10^5^ Vero E6/Vero 81 cells were plated in 6-well plates. 10-fold dilutions of harvested viruses were generated in 1 mL of PBS. Then 200 μL of diluted virus was added to each well. Inoculated cells were incubated at 37**°** C for 1 hour (rocking gently every 15 minutes). Then 3 mL per well of agarose, consisting of 2x DMEM, 1.6% Seaplaque low melting point agarose, 10% final concentration fetal clone II for SARS-CoV and 4% for MERS-CoV, and 1x Antibiotic/Antimycotic was added to the cells. After solidification, plates were incubated face up at 37**°** C and 5% CO_2_ for ~48 hours. 1x neutral red solution was added to the top of the agar and incubated for at least 2 hours. Neutral red was then removed and the plaques were counted. (ECL001, SCL005, SCL006, SHAE002, SHAE003, SHAE004, SM001, SM003, SM004, SM007, SM009, SM012, SM014, SM015, SM019, SM020).

In addition to viral titers, real-time PCR quantification of viral genomic and viral mRNA species was performed to confirm viral infection and for comparisons between time points both within an infection and across studies. For *Betacoronavirus* genus, first-strand cDNA synthesis was performed using 500 ng of total RNA and Thermo-Script reverse transcriptase (Invitrogen) according to the manufacturer’s protocol. For influenza, first-strand cDNA synthesis was performed similar to above except 15 pmol of RNA-specific primers for VN1203, CA04 or NL602 nucleoprotein genomic RNA and mRNA and endogenous control were used to prime the reactions. Primers for NP RNAs were designed with unique 5′ sequences, thereby ensuring differentiation between genomic RNA and mRNA species. The reverse transcriptase reaction primer sequences used were: NP genomic RNA (VN1203, CA04, and NL602) - 5′GGCCGTCATGGTGGCGAATAGCAAAAGCAGGGTAGATAATCACTC3′. NP mRNA (VN1203) - 5′CCAGATCGTTCGAGTCGTTTTTTTTTTTTTTTTTCTTTAATTGTC3′. NP mRNA (NL602 and CA04) - 5′CCAGATCGTTCGAGTCGTTTTTTTTTTTTTTTTTCAACTGTCATACTC3′. RPL14 (human endogenous control) - 5′TTCAATCTTCTTGGCCCATC3′. RPL10 (mouse endogenous control) - 5′GAACGATTTGGTAGGGTATAGGAG3′.

The qPCR assay was performed using a SYBR green kit (Applied Biosystems, Carlsbad, CA, USA) with specific primers for the different RNA species, according to the manufacturer’s standard protocol. Relative RNA quantities were determined using the comparative threshold cycle (*CT*) method, with human RPL14 or mouse RPL10 as endogenous. The qPCR primer sequences for endogenous controls used were:

RPL14 forward - 5′TTCATCCTCAAGTTTCCGC3′

RPL14 reverse - 5′TTCAATCTTCTTGGCCCATC3′

RPL10 forward - 5′TGAAGACATGGTTGCTGAGAAG3′

RPL10 reverse - 5′GAACGATTTGGTAGGGTATAGGAG3′.

The SARS-CoV qPCR primer sequences used were:

Forward primer for genomic RNA and mRNAs - 5′CCTACCCAGGAAAAGCCAAC3′.

Genomic RNA reverse primer - 5′GGACGAAACCGTAAGCAGTC3′

S mRNA reverse primer - 5′ACCGGTCAAGGTCACTAC3′

E mRNA reverse primer - 5′GCAAGAATACCACGAAAGCA3′.

The MERS-CoV qPCR primer sequences used were:

Forward primer for genomic RNA and mRNAs - 5′GAATAGCTTGGCTATCTCAC3′

Genomic RNA reverse primer - 5′CACAATCCCACCAGACAA3′

ORF2 reverse primer - 5′AGTGTATCATTGTCACGGATAAG3′

ORF5 reverse primer - 5′ACTAGCTGGACGGGTTTA3′

The Influenza qPCR primers sequences used were:

Genomic forward primer (VN1203, NL602 and CA04) - 5′GGCCGTCATGGTGGCGAAT3′

Genomic reverse primer (VN1203) - 5′GTTCCCCACCAGTTTCCATC3′

Genomic reverse primer (NL602 and CA04) - 5′GCTCCCCACCAGTCTCCATT3′

Forward mRNA primer (VN1203, NL602 and CA04) - 5′CCAGATCGTTCGAGTCGT3′

Reverse mRNA primer (VN1203, NL602 and CA04) - 5′CGAACCCGATCGTGCCTTCC3′

### Isolation of total RNA and quality control

Isolation of RNA from Trizol lysates was performed using Qiagen RNeasy Mini kit (Qiagen Inc., Valencia, CA). The steps of the protocol were performed as follows: lysates were mixed by vortexing followed by a 5-minute incubation at RT. To each sample, 200 μL of chloroform per mL Trizol was added and the tubes shook vigorously for 15 seconds. In many cases only a portion of the 1 ml Trizol lysate was used and the volume of chloroform was adjusted accordingly. After incubation for 10 minutes at RT, samples were spun at 12,000 g for 15 minutes at 4**°** C. The aqueous phase was transferred to a clean tube and an equal volume of RLT +BME added. The volume was then doubled by adding 70% ethanol and mixed well by pipetting. This solution was applied to a RNeasy mini column and the RNA was isolated from the column per the manufacturer’s instructions. Appropriate amounts of RNA for quality control were removed and the remaining RNA was frozen on dry ice and store at −80**°** C. Determination of RNA quality and quantity was done using an Agilent Bioanalyzer 2100 and a Nanodrop spectrophotometer following the manufacturers’ recommendations. (CA04M001, ICL004, ICL006, ICL010, ICL011, ICL012, IM001, IM002, IM004, IM005, IM006A, IM006B, IM007, IM009, IM010, ECL001, SCL005, SCL006, SHAE002, SHAE003, SHAE004, SM001, SM003, SM004, SM007, SM009, SM012, SM014, SM015, SM019, SM020).

Alternatively, chloroform (0.2 mL per 1 mL TRIzol) was added to samples, tubes were inverted 20 times, and incubated at room temperature for 3 minutes. Samples were centrifuged at 12,000 g for 15 minutes at 4° C. The aqueous phase (top phase) was transferred to a new tube and an equal volume of isopropyl alcohol was added and samples incubated for 10 minutes. Tubes were centrifuged at 12,000 g for 10 minutes at 4° C. The RNA pellet was washed once with 70% ethanol and re-suspended in 100 μL of H_2_0, 1 μL of glycogen and 1/10 volume of 3 M NaAc. RNA was re-precipitated with an equal volume of 100% ethanol and washed 2 times with 70% ethanol. The dried pellet was resuspended in 30 μL water. The RNA concentration and purity was determined by absorbance at 260 nm, 270 nm, and 280 nm using a plate reader. RNA integrity was determined using a BioAnalyzer (Agilent, Santa Clara, CA). (SBRI_AA_E1).

### Labeling and quantification of total RNA for agilent microarray

Fluorescently labeled probes were generated from each total RNA sample using Agilent one-color Low Input Quick Amp Labeling Kit (Agilent Technologies) following the manufacturer’s instructions. The cRNA probe synthesis began with adding 100 ng total intact RNA to 3.25 μL water, combining RNA and Master Mix (consisting of 1.0 μL H2O, 0.25 μL Spike-in (dilution 2), 0.8 μL T7 Promoter Primer, 2.05 μL Total Volume per reaction), denaturing for 10 minutes at 65**°** C, and incubating at 4**°** C for 5 minutes. Next, double-stranded cDNA was synthesized by combining the above cRNA with 4.7 μL per reaction of First Strand mix (consisting of 2.0 μL 5X first stand Buffer1, 1.0 μL 0.1 M DTT, 0.5 μL 10 mM dNTP mix, and 1.2 μL AffinityScript RNase Block Mix) at room temperature followed by incubations at 40**°** C for 2 hours, 70**°** C for 15 minutes, and 4**°** C for 5 minutes.

Labeling of each sample was done by adding 6.0 μL of transcription master mix (consisting of 0.75 μL H_2_O, 3.2 μL 5X Transcription Buffer, 0.6 μL 0.1 M DTT, 1.0 μL NTP Mix, 0.21 μL T7 RNA Polymerase Blend, and 0.24 μL of Cy3 per reaction), mixing by pipette, and incubating at 40**°** C for 2 hours in the dark. Next, probes were purified with the Qiagen Rneasy kit (RNA cleanup protocol). After 84 μL of nuclease free water was added (to 100 μL total volume), samples were transferred to 1.5 mL microfuge tubes. Next, 350 μL of RLT, and 250 μL of 100% ethanol were added and mixed thoroughly by pipetting. 700 μL of this mixture was then applied to a column, centrifuged 30 seconds at 13,000 rpm at 4**°** C (discard flow through and collection tube). The column was then transferred to a new collection tube, 500 μL of RPE washing buffer was added, and centrifuged for 30 seconds at 13,000 rpm at 4**°** C (discard flow through, reuse tube). Another 500 μL of RPE was added and centrifuged at 60 seconds at 13,000 rpm at 4**°** C, then transferred to a clean microfuge tube and spun for 30 seconds on max to dry column.

To elute labelled RNA, the column was transferred to a clean labeled collection microfuge tube and 30μL of nuclease free H_2_O was added to the center of the column for 60 seconds followed by a 60 second centrifugation at 13,000 rpm at 4**°** C. A small aliquot (~4 μL) was removed for quantification (see below). Samples were quick frozen on dry ice and stored at −80**°** C while protected from light. (CA04M001, ICL004, ICL006, ICL010, ICL011, ICL012, IM001, IM002, IM004, IM005, IM006A, IM006B, IM007, IM009, IM010, ECL001, SCL005, SCL006, SHAE002, SHAE003, SHAE004, SM001, SM003, SM004, SM007, SM009, SM012, SM014, SM015, SM019, SM020).

Quantification of cRNA probes was done by the Nanodrop spectrophotometry. For each sample 1.2 μL of purified probe was measured and cRNA concentration (ng/μL) and dye incorporation (pmol/μL) recorded. Specific activity was calculated by: (dye incorporation/ cRNA concentration)*1000. Results greater than 8.0 were used for hybridization.

### Agilent microarray hybridization and scanning

In summary, 250 ng of each RNA sample was hybridized to either an Agilent 4x44K mouse whole-genome oligonucleotide microarray G4122F (design ID 014868) or 4X44K human HG (design ID 014850) array according to the manufacturer’s instructions (details below).

Prior to hybridization, cRNA was first fragmented by mixing Cy3-cRNA per reaction volume of 55.0 μL (consisting of Cy3-cRNA 1650 ng, 11 μL of 10X Blocking Reagent, and Nuclease free H_2_O; Bring volume to 52.8 μL), with fragmentation buffer (consisting of 2.2 μL 25X fragmentation buffer) and then incubating at 60**°** C for 30 minutes in the dark. 55 μL of 2X Gene Expression Hybridization buffer HI-RPM was then added (Total volume=110 μL). Samples were mixed well by pipetting, and spun for 1 minute at room temperature at 13,000 rpm.

Subsequent to fragmentation, hybridization of 100 μL of the fragmented cRNA samples was transferred from above onto the center of each of the wells (4x44K format has 4 wells per slide with one sample per well). A slide was carefully lowered, bar code facing up, onto the bead of liquid, followed by the metal chamber cover and clamp. The chamber was rotated vertically to wet the gasket and make sure that all bubbles were mobile. Two chambers were load at a time into the hybridization oven, set at 65**°** C and level 10-rotation speed (10 rpm) for 17 hours.

Slides were then washed once with slide cover on in Gene Expression Wash Buffer 1, then cover was pried off and the slide washed in Gene Expression Wash Buffer 1 for a second time. Next, slides were washed in Gene Expression Wash Buffer 1 on a stir plate for ~1 minute and placed into pre-warmed Gene Expression Wash buffer 2 on a stir plate for ~1 minute. The excess liquid was blotted onto a Kimwipe and the dry slides placed into a light tight slide box to be scanned at once.

Dry slides were scanned on an Agilent DNA microarray scanner (model G2505B) using the XDR setting. Raw images were analyzed using the Agilent Feature Extraction software (version 9.5.3.1) and the GE1-v5_95_Feb07 extraction protocol. (CA04M001, ICL004, ICL006, ICL010, ICL011, ICL012, IM001, IM002, IM004, IM005, IM006A, IM006B, IM007, IM009, IM010, ECL001, SCL005, SCL006, SHAE002, SHAE003, SHAE004, SM001, SM003, SM004, SM007, SM009, SM012, SM014, SM015, SM019, SM020).

### Agilent microarray processing and expression analysis

Normalization and QA/QC: Raw data consisted of the output from the Agilent Feature Extraction (AFE) software. Data was read into the R/Bioconductor Framework and visualization of raw intensity level was performed using boxplots, histograms, and correlation plots. All intensities were background corrected using the norm-exp method and quantile normalized using Agi4x44PreProcess and RMA Bioconductor packages. Distribution of control probes was examined and then those probes were removed. Descriptive statistics were reported for non-control replicate probes and the intensity distributions were examined via histograms. All non-control replicate probes were then summarized by taking the mean intensity measurement. To assess the quality of the reading for each probe, five Agilent QC flags were considered. The first is a test for minimal spot diameter and signal greater than 1.5 times background noise (gIsFound); the second requires positive intensity and background-subtracted intensity greater than 2.6 times background noise (gIsWellAboveBG); the third is true if more than 50% of spot pixels are greater than the saturation threshold (gIsSaturated); the fourth examines variance of spot pixels (gIsFeatNonUnifOL); the fifth checks if replicate probe is greater than 1.42 times the interquartile range (gIsFeatPopnOL).

A given probe must pass all 5 QC flags for all treatment (i.e., infected) replicates of at least one experimental time point. This allowed a conservative criteria regarding probe quality, but was moderate enough to detect transcripts that may be expressed only briefly (i.e., as little as one time point) during an infection time course. It was imperative that this filtering occurred before differential expression analysis so that low intensity (below background) and low quality probes were not considered in the analysis. Final QA/QC involved CV plots, heatmaps, cluster plots, scatter plots and PCA as needed in order to assess if there were sample outliers or additional batch effects.

Differential expression analysis was performed by comparing infected samples to time-matched mock-infected controls, based on a linear model fit for each transcript/probe using the R package Limma^35^. Fold changes were calculated by taking the average log_2_ probe intensity of infected replicates and subtracting the average log_2_ probe intensity of mock-infected replicates from the same time point. Significant differential expression of a probe was defined using the following criteria: log_2_ fold change >|1.5| and an adjusted false discovery rate (FDR) *P* value of <0.05 for a given time point.

Software Utilized: Custom R Bioconductor workflow that includes the Agi4x44 Pre-Process and RMA Bioconductor packages^36^ plus in house scripts for QA/QC visualization and differential expression analysis. (CA04M001, ICL004, ICL006, ICL010, ICL011, ICL012, IM001, IM002, IM004, IM005, IM006A, IM006B, IM007, IM009, IM010, ECL001, SCL005, SCL006, SHAE002, SHAE003, SHAE004, SM001, SM003, SM004, SM007, SM009, SM012, SM014, SM015, SM019, SM020).

### Positive-strand cDNA synthesis, fragmentation, and single-stranded cDNA for affymetrix microarray analysis

cDNA was synthesized from isolated RNA (250 ng from each sample) using the Ambion WT kit (Life Technologies, Grand Island, NY, USA - http://tools.lifetechnologies.com/content/sfs/manuals/cms_064619.pdf) following the manufacturers protocol. Briefly: First strand cDNA was prepared from total RNA using reverse transcriptase and specific primers (5 μL of First-Strand master mix) resulting in single stranded cDNA which contains a T7 promoter sequence. Second strand cDNA was synthesized using Second-Strand master mix and the newly created double stranded DNA was isolated. Antisense cRNA was synthesized from the second strand DNA using T7 RNA polymerase then stored at 4 °C overnight. In the next step, cRNA was purified using cRNA Binding Mix and the yield was determined by absorbance at 260 nm. Purified cRNA was used to synthesize positive sense strand cDNA using reverse transcriptase and random primers. In this reaction, dUTP was added at a fixed ratio relative to dTTP. cRNA was then hydrolyzed using RNase H and cDNA purified using cDNA Binding Mix and cDNA yield determined by absorbance at 260 nm.

Single stranded cDNA was prepared for chip hybridization using the Affymetrix GeneChip WT Terminal Labeling Kit (Affymetrix, Santa Clara, CA, http://www.umich.edu/~caparray/Files/protocols/affymetrix/Ambion%20WT%20Labelling%20and%20Hyb.pdf) following the manufacturer’s protocol. Briefly: Single-stranded cDNA was fragmented using Fragmentation master mix from the kit, then labeled by terminal deoxynucleotidyl transferase (TdT) using the Affymetrix proprietary DNA Labeling Reagent. The labeled mix was then hybridized to GeneChip Mouse Exon 1.0 ST Arrays in a hybridization oven for 17 hours. (SBRI_AA_E1).

### Affymetrix microarray processing and expression analysis

Probe intensities were measured using the Affymetrix GeneChip Scanner 3000 and processed into CEL files using the Affymetrix GeneChip Operating Software. Microarrays were normalized at the gene level using the BrainArray custom CDF (Entrez Gene, Version 14) for probeset definitions and RMA as implemented in the justRMA function of the Bioconductor package affy for background adjustment, quantile normalization, and summarization. Linearized data was imported into Genedata Analyst software (Genedata AG, Basel, Switzerland). ANOVA was performed on all samples (100 balanced permutations) and genes with a permutation Q-value of 0.01 or less and fold change of at least 2 (between any two groups) were exported. (SBRI_AA_E1).

### Preparation of protein extracts for proteomic analysis

Protein concentrations of cell lysates or lung tissue homogenates were determined by BCA protein assay. The range of final protein amount was between 100-226 μg with an average of 150 μg per sample. Each sample was then diluted to uniform volume in 50 mM ammonium bicarbonate, pH 7.8. Proteins were reduced with 10 mM dithiothreitol, followed by alkylation of free sulfhydryl groups with 40 mM iodoacetamide at 37**°** C in the dark; each reaction was performed for 1 hour at 37**°** C with constant shaking at 550 rpm. Denatured and reduced samples were diluted 10-fold with 50 mM ammonium bicarbonate, pH 7.8, and CaCl_2_ was added to a final concentration of 1 mM prior to enzymatic digestion. Sequencing-grade modified trypsin was activated by adding 20 μL of 50 mM ammonium bicarbonate, pH 7.8, to 20 μg lyophilized trypsin and incubating for 10 min at 37**°** C. Activated trypsin was added to the samples at 1:50 (w/w) trypsin-to-protein ratio, and samples were digested at 37**°** C for 3 hours with constant shaking at 800 rpm; reactions were quenched by rapid freezing in liquid nitrogen. Digested samples were desalted using solid phase extraction columns (Discovery C18, Supelco, Bellefonte, PA, USA), which were conditioned with 3 mL of methanol and rinsed with 2 mL of 0.1% trifluoroacetic acid (TFA) in water. Digest-loaded columns were washed with 4 mL of H_2_O/acetonitrile (95:5, v/v) containing 0.1% TFA, and peptides were eluted with 1 mL of acetonitrile/H_2_O (80:20, v/v) containing 0.1% TFA. Samples were concentrated to 100 μL *in vacuo* (Speed-Vac SC 250 Express, Thermo Savant, Holbrook, NY, USA), and a BCA protein assay was performed to verify final peptide concentrations. Samples were stored at −80**°** C until either strong cation exchange fractionation followed by liquid chromatography-tandem mass spectrometry (LC-MS/MS) or quantitative LC-MS analyses. (CA04M001, ICL004, ICL006, ICL010, ICL011, ICL012, IM001, IM002, IM004, IM005, IM006A, IM006B, IM007, SCL005, SCL006, SM001).

### Strong cation exchange fractionation

Strong cation exchange fractionation was performed on pooled cell or lung protein digests using an Agilent 1100 HPLC System (Agilent, Palo Alto, CA, USA) equipped with a quaternary pump, degasser, diode array detector, peltier-cooled autosampler, and fraction collector (set at 4° C). Peptides (350 μg per injection) were separated with a PolySulfoethyl A (PolyLC Inc., Columbia, MD, USA) column (200 mm × 2.1 mm; 5 μm particles with 300-Å pores) with a 10 mm×2.1 mm guard column packed with the same material at a flow rate of 0.2 mL/min. The solvents consisted of (a) 10 mM ammonium formate, pH 3.0, and 25% acetonitrile, and (b) 500 mM ammonium formate [pH 6.8] and 25% acetonitrile. The following linear gradient was used: 100% solvent (a) for 10 min; ramp to 50% solvent (b) in 40 min; ramp to 100% solvent (b) in the next 10 min; followed by a 10 min hold in 100% solvent (b). Routinely, 24 fractions were collected from minute 30 to minute 65 of the gradient, and they were subsequently dried *in vacuo* and stored at −80**°** C until LC-MS/MS analysis. (CA04M001, ICL004, ICL006, ICL010, ICL011, ICL012, IM001, IM002, IM004, IM005, IM006A, IM006B, IM007, SCL005, SCL006, SM001).

### Reversed-phase capillary LC-MS-MS and LC-MS analyses

Capillary LC-MS/MS analysis was used to generate separate accurate mass and time (AMT) tag databases for virus-infected cells and lung homogenates (see below). For this, dried peptide fractions from pooled samples were reconstituted in 30 μL of 25 mM ammonium bicarbonate, pH 7.8, and analyzed using a 4-column custom-built capillary LC system coupled online to a linear ion trap mass spectrometer (LTQ; Thermo Scientific, San Jose, CA, USA) by way of an in-house manufactured electrospray ionization interface. Electrospray emitters were custom made using 150 μm outer diameter (o.d.) x 20 μm inner diameter (i.d.) chemically etched fused silica. Reversed-phase capillary columns were prepared by slurry packing 3-μm Jupiter C18 bonded particles (Phenomenex, Torrence, CA, USA) into a 75 μm x 65 cm fused silica capillary (Polymicro Technologies, Phoenix, AZ, USA) using 0.5 cm sol-gel plugs for particle retention. Mobile phases consisted of (a) 0.1% formic acid in water and (b) 0.1% formic acid in acetonitrile, and they were degassed on-line using a Degasys Model DG-2410 vacuum degasser (Dionex, Germany); the HPLC system was equilibrated at 10,000 psi with 100% mobile phase (a) for initial starting conditions. After loading 2.5 μg of peptides onto the column, the mobile phase was held at 100% mobile phase (a) for 50 min. Exponential gradient elution was initiated 50 min after sample loading with a column flow rate of 300 nL/min, and the mobile phase was ramped from 0% to 55% mobile phase (b) over 100 min using a 2.5 mL stainless steel mixing chamber, followed by a rapid increase to ~100% (b) for 10 min to wash the column. To identify the eluting peptides, the LTQ was operated in a data-dependent MS/MS mode (400-2,000 *m/z*), in which a full MS scan was followed by ten MS/MS scans using a normalized collision energy of 35%. A dynamic exclusion window of 1 min was used to discriminate against previously analyzed ions. The temperature of the heated capillary and the electrospray ionization (ESI) voltage were 200**°** C and 2.2 kV, respectively.

Following AMT tag database generation, capillary LC-MS analyses were performed on all virus-infected and mock-infected samples to generate quantitative data using the AMT tag approach. For this, individual dried peptide samples (see above) were reconstituted in 30 μL of 25 mM ammonium bicarbonate, pH 7.8, and analyzed in either duplicate or triplicate and in random order using identical chromatographic and electrospray conditions as for capillary LC-MS/MS analyses. The LC system was interfaced to an Exactive Orbitrap mass spectrometer (Thermo Scientific), and the temperature of the heated capillary and the ESI voltage were 250**°** C and 2.2 kV, respectively. Data were collected over the mass range 400-2,000 *m/z*. (CA04M001, ICL004, ICL006, ICL010, ICL011, ICL012, IM001, IM002, IM004, IM005, IM006A, IM006B, IM007, SCL005, SCL006, SM001).

### Development of the AMT tag database for virus-infected model systems

A novel AMT database was generated for peptides within the specific model system, using mock-infected and virus-infected samples. To generate the AMT tag database, aliquots of the virus infected or mock-infected samples were combined to make the appropriate sample pools. Each pool was subjected to strong cation exchange fractionation as described above, and each fraction was analyzed by capillary LC- MS/MS. The SEQUEST analysis software was used to match the MS/MS fragmentation spectra with sequences from the appropriate UniProt/Swiss-Prot protein database. When searching, SEQUEST used a dynamic mass modification on methionine residues corresponding to oxidation (15.9949 Da) and a static mass modification on cysteinyl residues to account for alkylation by iodoacetamide (57.0215 Da). Peptides passing the following filter criteria were stored as AMT tags in a Microsoft SQL Server database: 1) SEQUEST DelCn2 value≥0.10 (normalized Xcorr difference between the top scoring peptide and the second highest scoring peptide in each MS/MS spectrum) and 2) for the mouse lung tissue database, a SEQUEST correlation score (Xcorr)≥1.6, 2.4, and 3.2 for fully tryptic peptides with 1+, 2+, and 3+ charge states, respectively, and Xcorr≥4.3, and 4.7 for partially tryptic or non-tryptic protein terminal peptides with 2+, and 3+ charge states, respectively. While for the human cell line database, a SEQUEST correlation score (Xcorr)≥2, 2.6, and 3.5 for fully tryptic peptides with 1+, 2+, and 3+ charge states, respectively, and Xcorr≥2.5, 3.6, and 4.1 for partially tryptic or non-tryptic protein terminal peptides with 1+, 2+, and 3+ charge states, respectively. These filter criteria resulted in estimated peptide false discovery rates of <2% based on target-decoy database searches for both lung and cell samples. Non-tryptic peptides were excluded, and a minimum peptide length of 6 amino acid residues was required. The elution times for these peptides were normalized to a range of 0 to 1 using a predictive peptide LC normalized elution time (NET) model and linear regression. A NET average and standard deviation are assigned to each identified peptide if the same peptide is observed in multiple analyses. Both calculated monoisotopic masses and observed NETs of identified peptides were included in the AMT tag database. From the primary data records corresponding to the data used to generate the AMT tag database (PASS00416 and PASS00417), the user can download the raw data, the assembled database, and parameter files necessary to recreate the AMT-tag approach. In addition to the data files, a detailed tutorial has been made available for download. (CA04M001, ICL004, ICL006, ICL010, ICL011, ICL012, IM001, IM002, IM004, IM005, IM006A, IM006B, IM007, SCL005, SCL006, SM001).

### Processing of quantitative LC-MS datasets

Quantitative LC-MS datasets were processed using the PRISM Data Analysis system^37^, which is a series of software tools developed in-house (e.g. Decon2LS^38^ and VIPER^39^; freely available at http://ncrr.pnl.gov/software/). Individual steps in this data processing approach are reviewed here^40^. The peptide identities for detected features in each dataset (i.e. a single LC-MS analysis) were determined for features matched to AMT tags with high confidence based upon the accurate measured monoisotopic masses and NETs for each of the peptides in the filtered AMT tag databases within initial search tolerances of ±6 ppm and ±0.025 NET for monoisotopic mass and elution time, respectively. The peptides identified from this matching process were retained as matrices for subsequent data analysis. (CA04M001, ICL004, ICL006, ICL010, ICL011, ICL012, IM001, IM002, IM004, IM005, IM006A, IM006B, IM007, SCL005, SCL006, SM001).

### Proteomic data statistical processing

The integrated LC-MS peak intensity values (i.e. abundances) for the final peptide identifications were processed in a series of steps using MatLab R2010b that included quality control, normalization, protein quantification, and comparative statistical analyses. Quality control processing was performed to identify and remove peptides with an insufficient amount of data across the set of samples^41^, and LC-MS runs that showed significant deviation from the standard behavior of all LC-MS analyses^42^, using a significance level of 0.0001 (ref. 43). The peptide abundance values were normalized across the technical replicates using a rank invariant subset of peptides (p-value threshold of 0.1) followed by median absolute deviation centering of the data. Normalized log_10_ abundance values were averaged across the technical replicates within each biological sample. Proteins were quantified using a standard R-Rollup method^44^ using the most abundant reference peptide--after filtering the peptides that were redundant, had low data content, or were outside the dominant significance pattern. Comparative statistical analyses of time-matched mock with viral infected samples were performed using a Dunnett adjusted t-test to assess differences in protein average abundance. (CA04M001, ICL004, ICL006, ICL010, ICL011, ICL012, IM001, IM002, IM004, IM005, IM006A, IM006B, IM007, SCL005, SCL006, SM001).

## Data Records

Study designs for all experiments, including relevant repository identifiers, are summarized in Table 1 (Available online only), with individual RNA experiment samples detailed in Table 2 (Available online only) and individual protein experiment samples detailed in Table 3 (Available online only). Sample tracking from animal subjects to experiment samples is given in Table 4 (Available online only). Primary assay results (txt and mzXML files) are archived in public repositories as listed below (Data Record 1 and 2). Derived data resulting from processing and hypothesis testing accompanied by structured metadata describing both the experimental and data analysis methods are available at the Influenza Research Database and the Viral Pathogen Resource (Data Record 3).

### Data Record 1

Primary data for Agilent and Affymetrix microarray experiments are available at the NCBI Gene Expression Omnibus (GEO, http://www.ncbi.nlm.nih.gov/geo/) under the accession numbers GSE37569 (Data Citation 1)^8^, GSE45042 (Data Citation 2)^5^, GSE28166 (Data Citation 3)^4,45^, GSE37571 (Data Citation 4), GSE43203 (Data Citation 5), GSE43204 (Data Citation 6), GSE33263 (Data Citation 7)^8,45^, GSE36328 (Data Citation 8)^6^, GSE37572 (Data Citation 9)^8^, GSE43301 (Data Citation 10)^8^, GSE43302 (Data Citation 11)^8^, GSE44441 (Data Citation 12), GSE44445 (Data Citation 13)^8^, GSE40792 (Data Citation 14), GSE37245 (Data Citation 15)^9^, GSE33267 (Data Citation 16)^5,12^, GSE37827 (Data Citation 17), GSE47960 (Data Citation 18)^11^, GSE47961 (Data Citation 19)^11^, GSE47962 (Data Citation 20)^11^, GSE33266 (Data Citation 21), GSE50000 (Data Citation 22), GSE51386 (Data Citation 23), GSE50878 (Data Citation 24), GSE51387 (Data Citation 25), GSE49262 (Data Citation 26), GSE49263 (Data Citation 27), GSE40824 (Data Citation 28), GSE40827 (Data Citation 29).

### Data Record 2

Raw data files and associated parameter files (.raw,.mzXML) for all proteomics experiments including those used for AMT-TAG database generation have been uploaded to PeptideAtlas (http://www.peptideatlas.org). Experiment accession numbers are as follows: PASS00416 (Data Citation 30), PASS00417 (Data Citation 31), PASS00418 (Data Citation 32), PASS00419 (Data Citation 33), PASS00420 (Data Citation 34), PASS00421 (Data Citation 35), PASS00422 (Data Citation 36), PASS00423 (Data Citation 37), PASS00424 (Data Citation 38), PASS00425 (Data Citation 39), PASS00426 (Data Citation 40), PASS00427 (Data Citation 41), PASS00428 (Data Citation 42), PASS00429 (Data Citation 43), PASS00430 (Data Citation 44), PASS00431 (Data Citation 45), PASS00432 (Data Citation 46), PASS00433 (Data Citation 47).

### Data Record 3

Structured experiment metadata and derived data (.xls,.txt) including fold change values and p-values are available in the Host Factor Component of the Influenza Research Database (IRD, www.fludb.org) and the Virus Pathogen Resource (ViPR, www.viprbrc.org) under accession numbers: IRD_SV_CA04M001-P (Data Citation 48), IRD_SV_ICL004-P (Data Citation 49), IRD_SV_ICL006-P (Data Citation 50), IRD_SV_ICL010-P (Data Citation 51), IRD_SV_ICL011-P (Data Citation 52), IRD_SV_ICL012-P (Data Citation 53), IRD_SV_IM001-P (Data Citation 54), IRD_SV_IM004-P (Data Citation 55), IRD_SV_IM005-P (Data Citation 56), IRD_SV_IM006A-P (Data Citation 57), IRD_SV_IM006B-P (Data Citation 58), IRD_SV_IM007-P (Data Citation 59), IRD_SV_SCL005-P (Data Citation 60), IRD_SV_SCL006-P (Data Citation 61), IRD_SV_SM001-P (Data Citation 62), IRD_SV_CA04M001-R (Data Citation 63), IRD_SV_ECL001-R (Data Citation 64), IRD_SV_ICL004-R (Data Citation 65), IRD_SV_ICL006-R (Data Citation 66), IRD_SV_ICL010-R (Data Citation 67), IRD_SV_ICL011-R (Data Citation 68), IRD_SV_ICL012-R (Data Citation 69), IRD_SV_IM001-R (Data Citation 70), IRD_SV_IM002-R (Data Citation 71), IRD_SV_IM004-R (Data Citation 72), IRD_SV_IM005-R (Data Citation 73), IRD_SV_IM006A-R (Data Citation 74), IRD_SV_IM006B-R (Data Citation 75), IRD_SV_IM007-R (Data Citation 76), IRD_SV_IM009-R (Data Citation 77), IRD_SV_IM010-R (Data Citation 78), IRD_SI_SBRI_AA_E1 (Data Citation 79), IRD_SV_SCL005-R (Data Citation 80), IRD_SV_SCL006-R (Data Citation 81), IRD_SV_SHAE002-R (Data Citation 82), IRD_SV_SHAE003-R (Data Citation 83), IRD_SV_SHAE004-R (Data Citation 84), IRD_SV_SM001-R (Data Citation 85), IRD_SV_SM003-R (Data Citation 86), IRD_SV_SM004-R (Data Citation 87), IRD_SV_SM007-R (Data Citation 88), IRD_SV_SM009-R (Data Citation 89), IRD_SV_SM012-R (Data Citation 90), IRD_SV_SM014-R (Data Citation 91), IRD_SV_SM015-R (Data Citation 92), IRD_SV_SM019-R (Data Citation 93), IRD_SV_SM020-R (Data Citation 94).

## Technical Validation

Two approaches were used to establish the technical validity of the datasets described. First, the biological samples used to generate the transcriptomic and proteomic assay results reported here have also been assessed using various criteria to ensure technical validity. For example, productive viral infections following inoculation of animals used for *in vivo* studies was confirmed by examining viral titers, viral mRNA levels, and body weight loss to quantify virus replication and host response to infection as detailed below. For *in vitro* studies, viral genomic RNA, viral mRNA, and viral titers were quantified to ensure that the virus was actively replicating and transcribing its genome in cell culture. Second, the processed data produced from *in vivo* and *in vitro* studies were compared, using Boolean logic, to quantify the number of overlapping significant genes identified in multiple studies performed under similar experimental conditions as an assessment of general reproducibility of the derived results between experiments.

### *In vivo* validation

To validate the *in vivo* studies, mean viral mRNA levels, mean % body weight, and mean viral titers were examined as surrogates for viral infection in the animal subjects. The CA04M001 and IM001 experiments, in which mice were infected with influenza A virus strains A/California/04/2009 (pandemic H1N1) and A/Viet Nam/1203/2004 (H5N1) respectively, were used as representative datasets for this validation process. For both experiments, the viral mRNA levels indicate that the virus was actively transcribing mRNA within the host organism with mean viral mRNA levels peaking at either 2 or 4 days post infection (dpi), when either a high or low initial inoculum was used, respectively^8^. Similarly, the mice that were exposed to virus in either experiment showed a dose-dependent decrease in mean % body weight. The viral growth kinetics for A/California/04/2009 in the CA04M001 study showed mean viral titers peaking at 2 dpi regardless of the viral concentration present in the initial inoculation. In contrast, the growth kinetics for A/Viet Nam/1203/2004 used in the IM001 study showed mean viral titers peaking at 2 days post infection when the initial inoculum contained either 10^3^ or 10^4^ plaque forming units (PFU) of virus; whereas mean viral titers were highest at 4 dpi when the initial inoculum contained 10^2^ PFU of virus.

### *In vitro* validation

For *in vitro* studies, measurements from study ICL004, performed in Calu-3 cells infected with the A/Viet Nam/1203/2004 influenza virus strain, were used as representative of all studies. Mean viral genomic RNA levels increased logarithmically between 0 and 12 hours post infection (hpi), eventually peaking at 18 hpi before decreasing at 24 hpi. Similarly, mean viral mRNA levels increased exponentially between 0 and 7 hpi, where they peaked before decreasing through the 24-hour time point. Mean viral titers were observed to increase logarithmically between the 7 and 12-hour time points, and then continued to rise through 24 hpi^8^.

Overall, the metrics used to validate the *in vivo* and *in vitro* studies indicate that the biological samples that were taken from these sample sources were productively infected with the intended virus. Consequently, any downstream assays that were performed on these biological samples should give rise to biologically valid results.

### Reproducibility of derived results

The primary assay results obtained from the transcriptomic and proteomic experiments were processed to identify specific RNAs or peptides that were differentially expressed under a particular set of conditions (e.g. virus amount, time post infection, virus strain, etc.) in comparison with relevant control samples to derive so called ‘Host Factor Biosets’. Another measure of technical validity of the combined laboratory and analytical workflow is that the Host Factor Biosets derived from similar experiment samples treated in a similar way should have similar membership, thereby indicating experimental reproducibility. To examine experimental reproducibility, we determined the overlap that was observed between all experiments that used the same virus strains in the same infection system. In the evaluation reported here, we used datasets reporting results from wild-type A/California/04/2009 (H1N1), A/Viet Nam/1203/04 (H5N1), and SARS-CoV as being representatives of all experiments. In order to avoid complications related to slight differences in infection kinetics, we took the union of all biosets across the entire time course from each experiment (i.e. the non-redundant set of differentially expressed genes) for comparison. This approach can lead to a discrepancy between the total number of genes in each experiment due to differences in number of time points sampled. As an alternative strategy, the data could also be more strictly paired using specific time points and dosages.

Seven situations in which the same treatment conditions (viral strain and viral amount) were reproduced in separate experiments were identified. Since the magnitude of the responses and the number of time points differ between experiments, we calculated the percentage overlap of the number of host factors using the smallest set as the denominator for the sake of comparison. The smallest set was chosen as the denominator to calculate percentage overlap, which may not be an ideal comparison since this would tend to result in higher reproducibility values than if the largest set was used as the denominator. However, since we are primarily interested in measuring the commonality between similar experiments that have slight variations in study designs and are inherently noisy, we chose to focus on determining if the smaller set is largely a subset of the larger sets, which would demonstrate that the these significant hits are reproducible even in the presence of confounding variables such as biological and technical variation.

**Figure 2:**
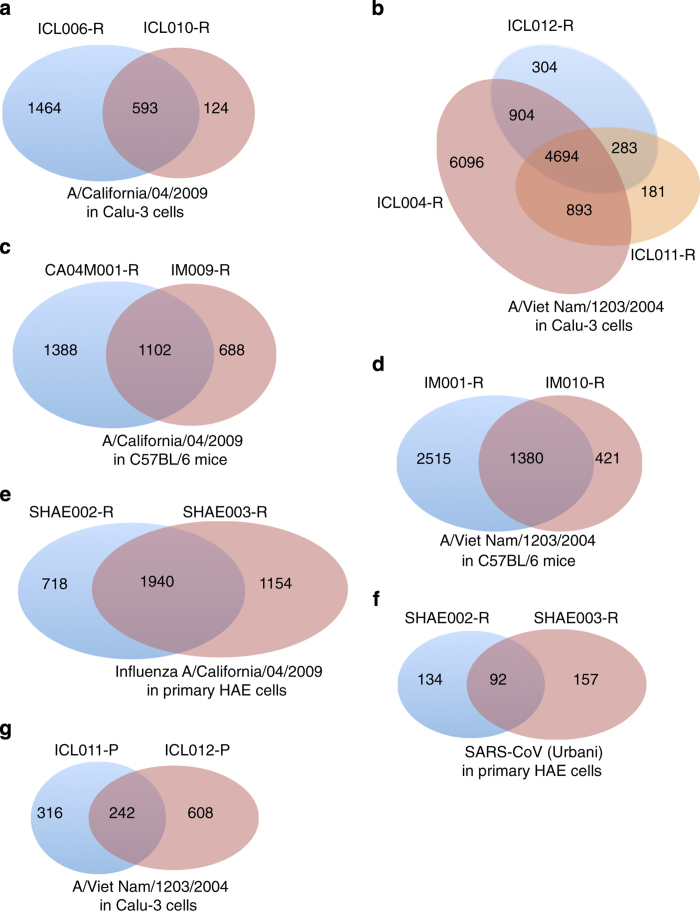
Computational validation of results. (**a**) Shows the comparison performed between all significant host factors identified by transcriptomics in the ICL006-R and ICL010-R experiments, both of which involved infection of Calu-3 cells with A/California/04/2009 at 3 multiplicity of infection (MOI). (**b**) Compares all significant host factors identified by transcriptomics in the ICL004-R, ICL011-R and ICL012-R experiments, which involved infection of Calu-3 cells with the A/Viet Nam/1203/2004 strain at 1 MOI. (**c**) A comparison between all significant host factors identified by transcriptomics in the CA04M001-R and IM009-R experiments, both of which involved infection of C57BL/6 mice with 10^6^ plaque forming units (PFU) of A/California/04/2009. (**d**) Compares all significant host factors identified by transcriptomics in the IM001-R and IM010-R experiments, both of which involved infection of C57BL/6 mice with 10^3^ PFU of A/Viet Nam/1203/2004. (**e**) Compares all significant host factors identified by transcriptomics in the SHAE002-R and SHAE003-R experiments, which involved infection of HAE cells with A/California/04/2009 at 2 MOI. (**f**) Compares all significant host factors identified by transcriptomics in the SHAE002-R and SHAE003-R experiments, which involved infection of HAE cells with the SARS-CoV Urbani strain at 2 MOI. (**g**) Compares all significant host factors identified by proteomics in the ICL011-P and ICL012-P experiments, which involved infection of Calu-3 cells with A/Viet Nam/1203/2004 at 1 MOI.

For transcriptomic experiments the following overlap was observed: 83% for A/California/04/2009 in Calu-3 (Fig. 2a), 91% for A/Viet Nam/1203/2004 in Calu-3 (Fig. 2b, 3-way comparison), 62% for A/California/04/2009 in C57BL/6 (Fig. 2c), 77% for A/Viet Nam/1203/2004 in C57BL/6 (Fig. 2d), 72% for A/California/04/2009 in HAE cells (Fig. 2e), and 40% for SARS-CoV in HAE cells (Fig. 2f). For the one replicated proteomic experiment, in which A/Viet Nam/1203/2004 was used to infect Calu-3, 43% overlap was observed (Fig. 2g). The reproducibility of host responses to both pandemic and highly pathogenic avian influenza viruses was high in both the Calu-3 cell line and mouse infection systems. Reproducibility using HAE was somewhat lower both in terms of the absolute number of host factor responding (Fig. 2e) and the percentage overlap (only 39% in Fig. 2f), perhaps due to the variability in establishing similar cell culture conditions using primary epithelial cells. Reproducibility of proteomic results was also somewhat lower with an overlap of 43% following infection of Calu-3 with A/Viet Nam/1203/2004 (Fig. 2g).

In general, the reproducibility of this collection of experiments using well-controlled standard operating procedures was quite high. In other microarray studies it has been shown that technical variability can result in 10% difference between replicate RNA samples^46,47^, while biological variability can cause anywhere from 10% to 30% differences depending on the biological system sampled^48^. In addition, variability in stimulation conditions has been found to have an even more profound impact than either technical or biological variability^47^. Thus, the studies compared here demonstrated considerable reproducibility in results in spite of the number of sources of variability inherent in their designs. Furthermore, reduction in the number of false positive results in any individual experiment (high specificity) could be achieved by focusing on the host factors that were reproducibly identified in similar experiments. Reduction in the number of false negative results (high sensitivity) could be achieved by using the union of host factors identified among similar experiments.

Overall, the results from the independent *in vitro* and *in vivo* validation experiments and the high degree of reproducibility of the derived host factor lists show that the datasets described here are of high quality and can be useful for in-depth investigation and hypothesis generation in follow-up analyses.

## Usage Notes

The systems biology studies described herein were designed to characterize how the host responds to viral infection. Separate experiments were performed to measure the response to multiple independent variables that include sampling at multiple time points after infection, at different viral dosages, in various model systems, and in systems with various genetic backgrounds (both viral and host). A number of publications have reported the use of these datasets to address questions concerning pathogenesis. For instance, comparisons between the similarities and dissimilarities of the host response at early and late stages of infection with genetically modified viral strains have been performed to identify possible correlates of disease severity^9^. Likewise, the host response was measured in different model systems to identify host factors that have a role in disease progression^45^. Lastly, all of these variables were combined to conduct a detailed evaluation of HPAI H5N1 infection based on time, dosage, and genetically-modified viruses^8^.

Various analytical and statistical tools can be used to interpret the results from systems biology experiments. The commonly-used methods fall into three categories: enrichment analysis, co-expression network analysis, and transcription factor predictions. Enrichment analysis tools, such as DAVID or GSEA^49–51^, use diverse statistical methods to identify annotations that are over-represented in a list of significant genes or proteins. Co-expression network analysis methods, such as Weighted Correlation Network Analysis (WGCNA)^52^, find correlated patterns across multiple microarray samples. Lastly, transcription factor prediction tools, such as PSCAN and PRISM^53,54^, examine the promoter regions of co-expressed genes to identify over-represented transcription factor binding sites. All of these tools allow researchers to generate hypotheses concerning how different components of the biological system being studied are modulated.

The host factor component within IRD and ViPR contains the processed data derived from these studies focusing primarily on the host response to virus infection. Here we present several scientific use cases that highlight some of the exploration and data mining uses that are currently possible using these data in the IRD/ViPR systems.

### Host responses to related viruses

Comparisons of host responses to infection by viruses that are phylogenetically related but associated with subtle or dramatic differences in disease symptoms or severity can be used to help identify the genetic determinants of virulence.

The IM002 experiment was designed to interrogate the different host responses that occurred when mice were infected with more distantly related H1N1 influenza A viruses: A/Brisbane/59/2007, A/New Jersey/8/1976, A/Mexico/4482/2009, and a reconstructed ‘Spanish flu’ strain r1918 at 1, 3, and 5 days post infection. Although all four viruses are of the H1N1 subtype, they differ in their apparent virulence characteristics in humans, with the original isolates of the A/New Jersey/8/1976, A/Mexico/4482/2009, and the r1918 strain all described as causing severe respiratory illness, whereas the A/Brisbane/59/2007 strain was isolated from a relatively mild case, and was included in the trivalent vaccine from 2008-2010. Previous analyses of the pathways and functions that were over-represented in the list of differentially expressed genes revealed that inflammatory response, cell-to-cell signaling, immune cell trafficking, lipid metabolism, amino acid metabolism, cardiovascular disease, and cancer gene responses were associated with virulence differences^6^.

**Figure 3:**
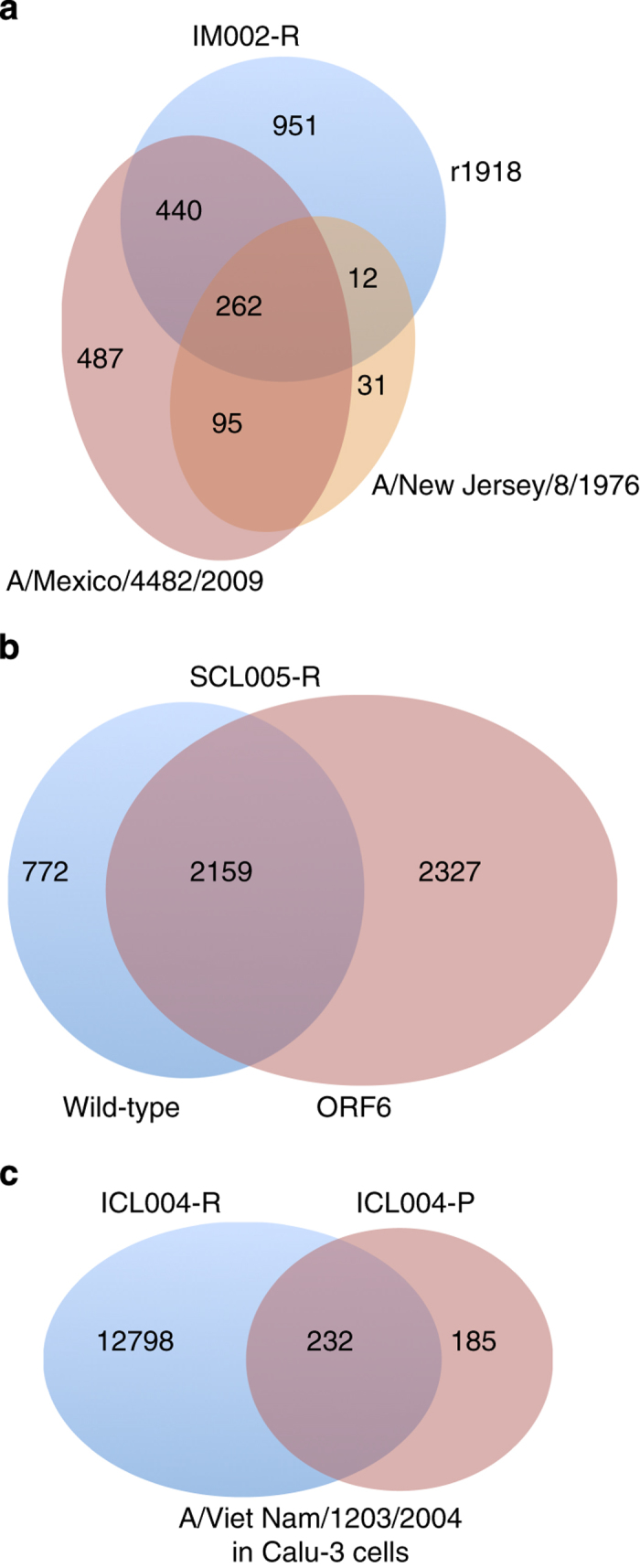
Comparative analyses usage examples. (**a**) Compares the significant host factors identified in the IM002-R experiment by transcriptomics at all time points by infecting C57BL/6 mice with 10^6^ PFU of H1N1 influenza strains A/Mexico/4482/2009, A/New Jersey/8/1976, or r1918. (**b**) Shows the comparison of significant factors identified by transcriptomics in the SCL005-R experiment that infected Calu-3 cells at 5 MOI with either wild-type SARS-CoV or a mutant containing a functional ORF6 knockout. (**c**) Results from the ICL004-R and ICL004-P experiments show the overlapping and unique host factors identified by transcriptomics and proteomics respectively for time points after (and including) 12 hpi in Calu-3 cells infected at 1 MOI with influenza strain A/Viet Nam/1203/2004.

To illustrate one potential way to compare datasets in ViPR/IRD, we quantified the overlap between differentially expressed genes identified from samples infected with this collection of ‘pre-pandemic’ H1N1 influenza A virus. As before, we compared the non-redundant union of significant genes derived from the transcriptomic results reported in the IM002 experiment to determine differences detected in mice infected with these viruses. Although the pairwise comparisons of the host response to virus infection were somewhat variable, the differences appeared to correlate with the virulence of the infecting virus. No overlap was observed between all three virulent viruses and the A/Brisbane/59/2007 strain. In contrast, 262 host factors (66%) were shared in mice infected with the three virulent viruses (Fig. 3a). Performing a functional annotation analysis for this list of 262 genes identified the same list of GO terms as reported in the original study^6^ except for the exclusion of cardiovascular disease and cancer.

Similar analyses designed to compare the host response to other related viruses could be performed using the datasets that are currently available in IRD/ViPR. For example, the ICL010-R experiment examines the host transcriptional response to 2 different 2009 influenza A (H1N1) pandemic strains in Calu-3 cells at 12-, 24, and 48 hpi. The ECL001-R and SCL005-R experiments could be analysed to better understand the similarities and differences in the host response during infection with either MERS or SARS-CoV in Calu-3 cells across multiple time points.

### Host responses to mutant viruses

In order to investigate how specific viral proteins might function to regulate host intracellular processes, experiments were performed to identify shared and unique host genes that are differentially expressed in hosts infected with either wild-type or mutant viruses^5,11,13^. For example, analysis of experimental results involving variants of SARS-CoV with a deletion in the ORF6 coding region performed in human cell lines (SCL005) or mice (SM012) determined that antagonism of karyopherin-based transport was an important function of the ORF6 gene product in SARS-CoV. As nuclear signaling disruption is a strategy used by many viral pathogens, identifying trends in these data presents a possible area for general therapeutic intervention.

Using Host Factor Bioset data in ViPR/IRD comparing human cell lines infected with either wild-type or ORF6 mutant viruses, 74% of significant host factors were shared between the two infection conditions (Fig. 3b). When an enrichment analysis was performed on the non-overlapping host factors (i.e. unique to infection with the ORF6 mutant strain), we found enrichment for terms relating to cell proliferation and immune response (*P*<0.05), which matches what had been reported previously^12^.

Similar analyses can be performed with data from other studies in IRD/ViPR to compare infection with wild-type versus genetically-engineered mutant strains. For instance, there are multiple studies that characterize the host responses to SARS-CoV variant strains in a human cell line (SCL006) and a mouse model (SM003, and SM012), and host responses to four influenza mutant variants of VN1203 in mice (IM004, IM005, IM006A, IM006B, and IM007) and a human cell line (ICL011 and ICL012).

### Differences between RNA and protein responses

While proteins carry the functional workload in cells, it is still challenging to achieve the levels of dynamic range necessary to measure their abundances and activities at a comprehensive genome-wide level to support systems-level analysis, given practical constraints upon measurement throughput and sensitivity. In contrast, while gene expression arrays allow for the comprehensive measurement of gene expression levels across the entire genome, these levels do not directly reflect functional physiology mediated by the encoded proteins. Therefore, it is interesting to compare quantitative proteomic and transcriptomic experiments using similar samples to estimate how often transcriptional profiles are reflected in protein profiles.

Prior reports have compared transcriptomic and proteomic data as a way of validating experimental results^12^. In these cases, proteomic data was integrated with the corresponding transcriptomic data to improve the biological networks produced and to support the associated statistical analysis of SARS-CoV host responses in general, and the karyopherin transport hubs in particular.

We performed a similar comparison of RNA and protein Host Factor Biosets in ViPR/IRD from the same tissue/cell samples using either microarray or mass spectrometry technologies (ICL004). In this case, transcriptomic data from the 12 h time point of human Calu-3 cells infected with A/Viet Nam/1203/2004 influenza virus was chosen because this time point contained the highest number of significant probes. These transcriptomic data were compared with a pooled list from the time-matched and all subsequent time points from the related proteomics data in order to control for the ‘lag’ that occurs between when the changes in mRNA expression are translated into corresponding changes in the translated protein product. We found approximately 56% overlap between the statistically significant RNA at 12 hpi and the statistically significant protein at the combined 12 and 18/24 hpi time points (Fig. 3c), similar to what has been reported previously^55,56^.

This type of analysis can be conducted on a number of other studies hosted by ViPR/IRD that evaluated both the transcript and protein host responses. These experiments included influenza infection in human cell lines (ICL006, ICL010, ICL011, ICL012), mice (CA04M001, IM001, IM004, IM005, IM006A, IM006B, IM007), and SARS-CoV infections in human cell lines (SCL005, SCL006) and mice (SM001).

### Meta-analysis

While many of the usage examples provided involve comparative analysis of results obtained in the same experiment, in some cases similar comparisons can be performed between experiments. Indeed, the Validation section focused on the reproducibility of derived results and involved the comparison of similar experiment conditions performed in separate experiments. Combining and contrasting results from different studies is often referred to as meta-analysis. One of the goals behind making these datasets freely available through public database resources like GEO, ViPR and IRD is to support their incorporation into meta-analysis approaches that include new experiments being performed by the research community. To facilitate this kind of meta-analysis, considerable effort has been dedicated to providing complete descriptions of all experimental and data analysis methodologies, combined with structured, standardized metadata describing selected key aspects of the experiment design, especially regarding the experiment factors that serve as the independent (manipulated) variables in the study in ViPR and IRD. Clear representations of the experiment metadata will make it relatively simple to appropriately combine these primary and derived results with data from future related experiments for meta-analysis.

### Re-analysis

Although we have emphasized the use of the derived Host Factor Biosets, in every case a close linkage is maintained between the processed data and the primary assay results from which they were derived-even though they may be stored in distinct resources. Thus, the final usage example is related to the re-processing and re-analysis of primary assay results.

Different processing algorithm(s) chosen to perform a specific processing or analysis step in a multistep workflow can yield different results. Many bioinformatics data processing workflows currently implement a ‘one size fits all’ approach with a single algorithm being applied to all datasets without regard for the specific nuances of each individual dataset. The problem with this approach is that the processing algorithms and parameters that are ideal for one dataset may not be optimal for other datasets—especially when dealing with different experimental systems. Hence, processing of the data using a single universally-applied algorithm may not sufficiently address the individual requirements of each dataset. To truly solve this underlying problem, an analytical workflow that explores the relevant parameter space for a diverse set of algorithms would need to be implemented. This approach would provide an objective and automated process to intelligently ‘choose’ the optimal combination of parameters and algorithms that produce the best set of results for each individual dataset. Such an approach could be implemented in analytical workflow suites like Galaxy or GenePattern^57,58^ to objectively and confidently reprocess the raw data from each ‘-omics’ dataset in a data-driven fashion to further improve the quality of the derived data. Until such a system is developed, we advocate for using the processed data that was provided by the original submitters and subjected to the peer-review process when performing meta-analyses given its overall high quality.

## Additional information

Tables 1–6 are only available in the online version of this paper.

**How to cite:** Aevermann, B. D. *et al.* A comprehensive collection of systems biology data characterizing the host response to viral infection. *Sci. Data* 1:140033 doi: 10.1038/sdata.2014.33 (2014).

## Supplementary Material



## Figures and Tables

**Table 1:** Study and experiment level metadata

**Study**	**Study Name**	**Experiment ID**	**Analyte Type**	**Treatment Agent**	**Dose**	**Time Post Administration**	**Study Design**	**Host Species**	**Strain/Line**	**Sample Source**	**Institution**	**Principle Investigator**	**Primary Public Repository**	**Primary Archive Accession**	**BRC**	**BRC ACCESSION**
**CA04M001**	CA04M001 :A/CA/04/09 (H1N1) infection in C57BL6 mice with variable doses and times post infection.	CA04M001-P	Protein Quantification	A/California/04/2009 (H1N1)	1.0E04, 1.0E05 PFU	1,2,4,7 days	Dose Response, Longitudinal	Mouse	C57BL/6	Lung Tissue	Systems Virology	Michael G. Katze	PeptideAtlas	PASS00423	IRD/ViPR	IRD_SV_CA04M001-P
**CA04M001**	CA04M001 :A/CA/04/09 (H1N1) infection in C57BL6 mice with variable doses and times post infection.	CA04M001-R	Transcript Quantification	A/California/04/2009 (H1N1)	1.0E03, 1.0E04, 1.0E05, 1.0E06 PFU	1,2,4,7 days	Dose Response, Longitudinal	Mouse	C57BL/6	Lung Tissue	Systems Virology	Michael G. Katze	GEO	GSE37569	IRD/ViPR	IRD_SV_CA04M001-R
**ECL001**	ECL001: MERS-CoV infection in Calu3 cells: A time course	ECL001-R	Transcript Quantification	MERS-CoV	5 MOI	0,3,7,12,18,24 h	Longitudinal	Human	Calu3 cells	Cell Line	Systems Virology	Michael G. Katze	GEO	GSE45042	IRD/ViPR	IRD_SV_ECL001-R
**ICL004**	ICL004: A/Vietnam/1203/2004 (H5N1) infection in Calu3 cell: A time course	ICL004-P	Protein Quantification	A/Vietnam/1203/2004 (H5N1)	1 MOI	0/3,7,12,18/24 h	Longitudinal	Human	Calu3 cells	Cell Line	Systems Virology	Michael G. Katze	PeptideAtlas	PASS00424	IRD/ViPR	IRD_SV_ICL004-P
**ICL004**	ICL004: A/Vietnam/1203/2004 (H5N1) infection in Calu3 cell: A time course	ICL004-R	Transcript Quantification	A/Vietnam/1203/2004 (H5N1)	1 MOI	0,12,18,24,3,7 h	Longitudinal	Human	Calu3 cells	Cell Line	Systems Virology	Michael G. Katze	GEO	GSE28166	IRD/ViPR	IRD_SV_ICL004-R
**ICL006**	ICL006: A/CA/04/09 (H1N1) infection in Calu3 cell: A time course	ICL006-P	Protein Quantification	A/California/04/2009 (H1N1)	3 MOI	0,3,7,12,18,24,30,36,48 h	Longitudinal	Human	Calu3 cells	Cell Line	Systems Virology	Michael G. Katze	PeptideAtlas	PASS00425	IRD/ViPR	IRD_SV_ICL006-P
**ICL006**	ICL006: A/CA/04/09 (H1N1) infection in Calu3 cell: A time course	ICL006-R	Transcript Quantification	A/California/04/2009 (H1N1)	3 MOI	0,12,18,24,3,30,36,48,7 h	Longitudinal	Human	Calu-3 cells	Cell Line	Systems Virology	Michael G. Katze	GEO	GSE37571	IRD/ViPR	IRD_SV_ICL006-R
**ICL010**	ICL010: A/Netherlands/602/2009 (H1N1) and A/CA/04/2009 (H1N1) infection in Calu3 cells: A time course	ICL010-P	Protein Quantification	A/Netherlands/602/2009 (H1N1)	3 MOI	0,3,7,12,18,24,30,36,48 h	Longitudinal	Human	Calu-3 cells	Cell Line	Systems Virology	Michael G. Katze	PeptideAtlas	PASS00426	IRD/ViPR	IRD_SV_ICL010-P
**ICL010**	ICL010: A/Netherlands/602/2009 (H1N1) and A/CA/04/2009 (H1N1) infection in Calu3 cells: A time course	ICL010-R	Transcript Quantification	A/California/04/2009 (H1N1),NL602 (H1N1)	3 MOI	0,12,24,48,3,7,18,30,36 h	Longitudinal	Human	Calu-3 cells	Cell Line	Systems Virology	Michael G. Katze	GEO	GSE40844	IRD/ViPR	IRD_SV_ICL010-R
**ICL011**	ICL011: VN1203 PB2-627E and PB1-F2del infection in Calu3 cells: A time course	ICL011-P	Protein Quantification	A/Vietnam/1203/2004 (H5N1), A/Vietnam/1203-CIP048_RG2 (H5N1), A/Vietnam/1203-CIP048_RG3 (H5N1)	1 MOI	7,24,0,3,12,18 h	Genetic, Longitudinal	Human	Calu-3 cells	Cell Line	Systems Virology	Michael G. Katze	PeptideAtlas	PASS00427	IRD/ViPR	IRD_SV_ICL011-P
**ICL011**	ICL011: VN1203 PB2-627E and PB1-F2del infection in Calu3 cells: A time course	ICL011-R	Transcript Quantification	A/Vietnam/1203/2004 (H5N1), A/Vietnam/1203-CIP048_RG2 (H5N1), A/Vietnam/1203-CIP048_RG3 (H5N1)	1 MOI	7,12,18,24,0,3 h	Genetic, Longitudinal	Human	Calu-3 cells	Cell Line	Systems Virology	Michael G. Katze	GEO	GSE43203	IRD/ViPR	IRD_SV_ICL011-R
**ICL012**	ICL012: Vietnam/1203/2004 (H5N1) and Vietnam/1203-NS1-trunc124/2004 (H5N1) infection in Calu3 cells: A time course	ICL012-P	Protein Quantification	A/Vietnam/1203/2004 (H5N1), A/Vietnam/1203-CIP048_RG4 (H5N1)	1 MOI	24,7,0,3,12,18 h	Genetic, Longitudinal	Human	Calu-3 cells	Cell Line	Systems Virology	Michael G. Katze	PeptideAtlas	PASS00428	IRD/ViPR	IRD_SV_ICL012-P
**ICL012**	ICL012: Vietnam/1203/2004 (H5N1) and Vietnam/1203-NS1-trunc124/2004 (H5N1) infection in Calu3 cells: A time course	ICL012-R	Transcript Quantification	A/Vietnam/1203/2004 (H5N1), A/Vietnam/1203-CIP048_RG4 (H5N1)	1 MOI	7,24,0,3,12,18 h	Genetic, Longitudinal	Human	Calu-3 cells	Cell Line	Systems Virology	Michael G. Katze	GEO	GSE43204	IRD/ViPR	IRD_SV_ICL012-R
**IM001**	IM001:A/Vietnam/1203/2004(H5N1) infection in C57BL6 mice with variable doses and times post infection.	IM001-P	Protein Quantification	A/Vietnam/1203/2004 (H5N1)	1.0E02, 1.0E03, 1.0E04 PFU	1,2,4,7 days	Dose Response, Longitudinal	Mouse	C57BL6	Lung Tissue	Systems Virology	Michael G. Katze	PeptideAtlas	PASS00418	IRD/ViPR	IRD_SV_IM001-P
**IM001**	IM001:A/Vietnam/1203/2004(H5N1) infection in C57BL6 mice with variable doses and times post infection.	IM001-R	Transcript Quantification	A/Vietnam/1203/2004 (H5N1)	1.0E02, 1.0E03, 1.0E04 PFU	1,2,4,7 days	Dose Response, Longitudinal	Mouse	C57BL/6	Lung Tissue	Systems Virology	Michael G. Katze	GEO	GSE33263	IRD/ViPR	IRD_SV_IM001-R
**IM002**	IM002: Influenza A/NJ/8/76 (H1N1), A/Mexico/4482/2009 (H1N1), Brisbane/59/2007 (H1N1) or reconstructed 1918 (H1N1) infection in BALB/c mice: A time course	IM002-R	Transcript Quantification	A/New Jersey/8/76 (H1N1), A/Mexico/4482/2009 (H1N1), A/Brisbane/59/2007 (H1N1), Reconstructed 1918 (H1N1)	1.0E06 PFU	1,3,5 days	Genetic, Longitudinal	Mouse	BALB/c	Lung Tissue	Systems Virology	Michael G. Katze	GEO	GSE36328	IRD/ViPR	IRD_SV_IM002-R
**IM004**	IM004:VN1203 HA avirluent mutant virus ( A/Vietnam/1203-CIP048_RG1/2004 (H5N1)) infection in C57BL6 mice: A time course	IM004-P	Protein Quantification	A/Vietnam/1203-CIP048_RG1 (H5N1)	1.0E04 PFU	4,7,1,2 days	Longitudinal	Mouse	C57BL/6	Lung Tissue	Systems Virology	Michael G. Katze	PeptideAtlas	PASS00419	IRD/ViPR	IRD_SV_IM004-P
**IM004**	IM004:VN1203 HA avirluent mutant virus ( A/Vietnam/1203-CIP048_RG1/2004 (H5N1)) infection in C57BL6 mice: A time course	IM004-R	Transcript Quantification	A/Vietnam/1203-CIP048_RG1 (H5N1)	1.0E04 PFU	1,2,4,7 days	Longitudinal	Mouse	C57BL/6	Lung Tissue	Systems Virology	Michael G. Katze	GEO	GSE37572	IRD/ViPR	IRD_SV_IM004-R
**IM005**	IM005: Vietnam/1203-PB2-627E/2004 (H5N1) infection in C57 Black 6 Mice: A time course	IM005-P	Protein Quantification	A/Vietnam/1203-CIP048_RG2 (H5N1)	1.0E04 PFU	1,2,4,7 days	Longitudinal	Mouse	C57BL/6	Lung Tissue	Systems Virology	Michael G. Katze	PeptideAtlas	PASS00420	IRD/ViPR	IRD_SV_IM005-P
**IM005**	IM005: Vietnam/1203-PB2-627E/2004 (H5N1) infection in C57 Black 6 Mice: A time course	IM005-R	Transcript Quantification	A/Vietnam/1203-CIP048_RG2 (H5N1)	1.0E04 PFU	4,1,2,7 days	Longitudinal	Mouse	C57BL/6	Lung Tissue	Systems Virology	Michael G. Katze	GEO	GSE43301	IRD/ViPR	IRD_SV_IM005-R
**IM006A**	IM006A: Vietnam/1203-PB1-F2del/2004 (H5N1) infection in C57 Black 6 Mice: A time course	IM006A-P	Protein Quantification	A/Vietnam/1203-CIP048_RG3 (H5N1)	1.0E03 PFU	1,2,4,7 days	Longitudinal	Mouse	C57BL/6	Lung Tissue	Systems Virology	Michael G. Katze	PeptideAtlas	PASS00422	IRD/ViPR	IRD_SV_IM006A-P
**IM006A**	IM006A: Vietnam/1203-PB1-F2del/2004 (H5N1) infection in C57 Black 6 Mice: A time course	IM006A-R	Transcript Quantification	A/Vietnam/1203-CIP048_RG3 (H5N1)	1.0E03 PFU	1,2,4,7 days	Longitudinal	Mouse	C57BL/6	Lung Tissue	Systems Virology	Michael G. Katze	GEO	GSE43302	IRD/ViPR	IRD_SV_IM006A-R
**IM006B**	IM006B: Vietnam/1203-PB1-F2del/2004 (H5N1) infection in C57 Black 6 Mice: A time course	IM006B-P	Protein Quantification	A/Vietnam/1203-CIP048_RG3 (H5N1)	1.0E04 PFU	1,2,4 days	Longitudinal	Mouse	C57BL/6	Lung Tissue	Systems Virology	Michael G. Katze	PeptideAtlas	PASS00422	IRD/ViPR	IRD_SV_IM006B-P
**IM006B**	IM006B: Vietnam/1203-PB1-F2del/2004 (H5N1) infection in C57 Black 6 Mice: A time course	IM006B-R	Transcript Quantification	A/Vietnam/1203-CIP048_RG3 (H5N1)	1.0E04 PFU	1,2,7,4 days	Longitudinal	Mouse	C57BL/6	Lung Tissue	Systems Virology	Michael G. Katze	GEO	GSE44441	IRD/ViPR	IRD_SV_IM006B-R
**IM007**	IM007: Vietnam/1203-NS1trunc-124/2004 (H5N1) infection in C57 Black 6 Mice: A time course	IM007-P	Protein Quantification	A/Vietnam/1203-CIP048_RG4 (H5N1)	1.0E03, 1.0E04 PFU	2,4,7,1 days	Dose Response, Longitudinal	Mouse	C57BL/6	Lung Tissue	Systems Virology	Michael G. Katze	PeptideAtlas	PASS00421	IRD/ViPR	IRD_SV_IM007-P
**IM007**	IM007: Vietnam/1203-NS1trunc-124/2004 (H5N1) infection in C57 Black 6 Mice: A time course	IM007-R	Transcript Quantification	A/Vietnam/1203-CIP048_RG4 (H5N1)	1.0E03, 1.0E04 PFU	1,2,4,7 days	Dose Response, Longitudinal	Mouse	C57BL/6	Lung Tissue	Systems Virology	Michael G. Katze	GEO	GSE44445	IRD/ViPR	IRD_SV_IM007-R
**IM009**	IM009: Influenza A/CA/04/2009 (H1N1) or, mouse-adapted A/CA/04/2009 (H1N1) infection in BALB/c mice: A time course	IM009-R	Transcript Quantification	A/California/04/2009 (H1N1), MA1-A/ California/04/2009 (H1N1)	1.0E06 PFU	1,3,5 days	Genetic, Longitudinal	Mouse	BALB/c	Lung Tissue	Systems Virology	Michael G. Katze	GEO	GSE36328	IRD/ViPR	IRD_SV_IM009-R
**IM010**	IM010: Vietnam/1203/2004 (H5N1) infection in knockout mutants of C57 Black 6 Mice	IM010-R	Transcript Quantification	A/Vietnam/1203/2004 (H5N1)	1.0E03 PFU	2,6 days	Genetic, Longitudinal	Mouse	C57BL/6,IDO1,Tnfrsf1b	Lung Tissue	Systems Virology	Michael G. Katze	GEO	GSE40792	IRD/ViPR	IRD_SV_IM010-R
**SBRI_AA_E1**	SBRI_AA: PR8, X31, VN6+2 12hrs infection in whole lung tissue. 10^5 pfu.	SBRI_AA_E1	Transcript Quantification	A/Aichi/02/68 (HA, NA) x A/Puerto Rico/8/34 (X31), A/Puerto Rico/8/34 (H1N1),A/Vietnam/1203/04 (HA Vn1203, NA) x A/Puerto Rico/8/34 (VN6+2)	1.0E05 PFU	12 h	Genetic	Mouse	C57BL/6J	Lung Tissue	Systems Influenza	Alan Aderem	GEO	GSE37245	IRD/ViPR	IRD_SI_SBRI_AA_E1
**SCL005**	SCL005: SARS Urbani or delta ORF6 Urbani infection in Calu3 cells with variable doses and times post infection.	SCL005-P	Protein Quantification	icSARS CoV Urbani,icSARS dORF6	5 MOI	0,3,7,12,60,72,24,30,36,48,54 h	Genetic, Longitudinal	Human	Calu-3 cells	Cell Line	Systems Virology	Michael G. Katze	PeptideAtlas	PASS00430	IRD/ViPR	IRD_SV_SCL005-P
**SCL005**	SCL005: SARS Urbani or delta ORF6 Urbani infection in Calu3 cells with variable doses and times post infection.	SCL005-R	Transcript Quantification	icSARS CoV Urbani,icSARS dORF6	5 MOI	0,12,24,3,30,36,48,54,60,7,72 h	Genetic, Longitudinal	Human	Calu-3 cells (clone 2B5)	Cell Line	Systems Virology	Michael G. Katze	GEO	GSE33267	IRD/ViPR	IRD_SV_SCL005-R
**SCL006**	SCL006: icSARS urbani vs icSARS BatSRBD mutant infection in Calu3 cell: A time course	SCL006-P	Protein Quantification	icSARS Bat SRBD,icSARS CoV Urbani	1 MOI	0,3,7,12,24,30,36,48,54,60,72 h	Genetic, Longitudinal	Human	Calu-3 cells	Cell Line	Systems Virology	Michael G. Katze	PeptideAtlas	PASS00431	IRD/ViPR	IRD_SV_SCL006-P
**SCL006**	SCL006: icSARS urbani vs icSARS BatSRBD mutant infection in Calu3 cell: A time course	SCL006-R	Transcript Quantification	icSARS Bat SRBD,icSARS CoV Urbani	1 MOI	0,12,24,30,36,48,54,60,7,72 h	Genetic, Longitudinal	Human	Calu-3 cells	Cell Line	Systems Virology	Michael G. Katze	GEO	GSE37827	IRD/ViPR	IRD_SV_SCL006-R
**SHAE002**	SHAE005: SARS-CoV, deltaORF6, BatSRBD mutants and A/CA/04/2009 (H1N1) infection of HAE cells: A time course	SHAE002-R	Transcript Quantification	A/California/04/2009 (H1N1),icSARS Bat SRBD,icSARS CoV Urbani,icSARS dORF6	2 MOI	96,0,12,24,36,48,60,72,84,6,18 h	Genetic, Longitudinal	Human	HAE cells	Cell Line	Systems Virology	Michael G. Katze	GEO	GSE47960	IRD/ViPR	IRD_SV_SHAE002-R
**SHAE003**	SHAE005: SARS-CoV, deltaORF6, BatSRBD mutants and A/CA/04/2009 (H1N1) infection of HAE cells: A time course	SHAE003-R	Transcript Quantification	A/California/04/2009 (H1N1),icSARS Bat SRBD,icSARS CoV Urbani,icSARS dORF6	2 MOI	24,48,60,72,84,96,0,6,12,18,36 h	Genetic, Longitudinal	Human	HAE cells	Cell Line	Systems Virology	Michael G. Katze	GEO	GSE47961	IRD/ViPR	IRD_SV_SHAE003-R
**SHAE004**	SHAE005: SARS-CoV, deltaORF6, BatSRBD mutants and A/CA/04/2009 (H1N1) infection of HAE cells: A time course	SHAE004-R	Transcript Quantification	A/California/04/2009 (H1N1),icSARS Bat SRBD,icSARS CoV Urbani,icSARS dORF6	1,2 MOI	0,12,24,36,48,60,72,84,96,6,18 h	Genetic, Longitudinal	Human	HAE cells	Cell Line	Systems Virology	Michael G. Katze	GEO	GSE47962	IRD/ViPR	IRD_SV_SHAE004-R
**SM001**	SM001: SARS MA15 infection in C57BL6 mice with variable doses and times post infection.	SM001-P	Protein Quantification	SARS CoV MA15	1.0E02, 1.0E03, 1.0E04, 1.0E05 PFU	1,2,4,7 days	Dose Response, Longitudinal	Mouse	C57BL6	Lung Tissue	Systems Virology	Michael G. Katze	PeptideAtlas	PASS00433	IRD/ViPR	IRD_SV_SM001-P
**SM001**	SM001: SARS MA15 infection in C57BL6 mice with variable doses and times post infection.	SM001-R	Transcript Quantification	SARS CoV MA15	1.0E02, 1.0E03, 1.0E04, 1.0E05 PFU	1,2,4,7 days	Dose Response, Longitudinal	Mouse	C57BL/6	Lung Tissue	Systems Virology	Michael G. Katze	GEO	GSE33266	IRD/ViPR	IRD_SV_SM001-R
**SM003**	SM003: icSARS, SARS MA15 or SARS BatSRBD mutant virus infection of C57 Black 6 mice: A time course	SM003-R	Transcript Quantification	SARS CoV MA15,icSARS Bat SRBD,icSARS CoV Urbani	1.0E04, 1.0E05 PFU	1,2,4,7 days	Genetic, Dose Response, Longitudinal	Mouse	C57BL/6	Lung Tissue	Systems Virology	Michael G. Katze	GEO	GSE50000	IRD/ViPR	IRD_SV_SM003-R
**SM004**	SM004: SARS MA15 infection in C57 Black 6, TIMP1 and Serpine1 (PAI1) knock-out mice: A time course	SM004-R	Transcript Quantification	SARS CoV MA15	1.0E04 PFU	4,7 days	Dose Response, Longitudinal	Mouse	C57BL/6,Serpine1 (PAI1),TIMP1	Lung Tissue	Systems Virology	Michael G. Katze	GEO	GSE51386	IRD/ViPR	IRD_SV_SM004-R
**SM007**	SM007: SARS MA15 infection in CXCR3 knockout mutant of C57 Black 6 Mice.	SM007-R	Transcript Quantification	SARS CoV MA15	1.0E04 PFU	7,1,2,4 days	Dose Response, Longitudinal	Mouse	CXCR3,C57BL/6	Lung Tissue	Systems Virology	Michael G. Katze	GEO	GSE50878	IRD/ViPR	IRD_SV_SM007-R
**SM009**	SM009: SARS MA15 infection in C57 Black 6 and PLAT knock-out mice: A time course	SM009-R	Transcript Quantification	SARS CoV MA15	1.0E05 PFU	4,7 days	Dose Response, Longitudinal	Mouse	PLAT,C57BL/6	Lung Tissue	Systems Virology	Michael G. Katze	GEO	GSE51387	IRD/ViPR	IRD_SV_SM009-R
**SM012**	SM012: SARS deltaORF6 mutant infection in C57 Black 6 Mice: A time course	SM012-R	Transcript Quantification	SARS CoV MA15,icSARS dORF6	1.0E06 PFU	4,1,2,7 days	Genetic, Longitudinal	Mouse	C57BL/6	Lung Tissue	Systems Virology	Michael G. Katze	GEO	GSE49262	IRD/ViPR	IRD_SV_SM012-R
**SM014**	SM012: SARS nsp16 mutant infection in C57 Black 6 Mice: A time course	SM014-R	Transcript Quantification	SARS CoV MA15,icSARS dNSP16	1.0E06 PFU	1,2,4,7 days	Genetic, Longitudinal	Mouse	C57BL/6	Lung Tissue	Systems Virology	Michael G. Katze	GEO	GSE49263	IRD/ViPR	IRD_SV_SM014-R
**SM015**	SM015: SARS MA15 infection in Tnfrsf1a/1b knockout mutant of C57 Black 6 Mice.	SM015-R	Transcript Quantification	SARS CoV MA15	1.0E05 PFU	7,4 days	Longitudinal	Mouse	C57BL/6,Tnfrsf1a/1b	Lung Tissue	Systems Virology	Michael G. Katze	GEO	GSE43301	IRD/ViPR	IRD_SV_SM015-R
**SM019**	SM019: SARS MA15 infection in Tnfrsf1b knockout mutant of C57 Black 6 Mice.	SM019-R	Transcript Quantification	SARS CoV MA15	1.0E05 PFU	7,4 days	Longitudinal	Mouse	C57BL/6,Tnfrsf1b	Lung Tissue	Systems Virology	Michael G. Katze	GEO	GSE40824	IRD/ViPR	IRD_SV_SM019-R
**SM020**	SM020: SARS MA15 infection in ppp1r14c knockout mutant of C57 Black 6 Mice.	SM020-R	Transcript Quantification	SARS CoV MA15	1.0E05 PFU	7,4 days	Longitudinal	Mouse	C57BL/6,ppp1r14c	Lung Tissue	Systems Virology	Michael G. Katze	GEO	GSE40827	IRD/ViPR	IRD_SV_SM020-R

**Table 2:** Experiment samples from transcriptomic assays

**Experiment ID**	**Experiment Sample ID**	**Biological Sample Type**	**PUBLIC REPOSITORY NAME**	**REPOSITORY ID**
CA04M001-R	CA04M001_10^3_1d_2_RNA_ExpSam	RNA	GEO	GSM921810
CA04M001-R	CA04M001_10^3_1d_3_RNA_ExpSam	RNA	GEO	GSM921811
CA04M001-R	CA04M001_10^3_1d_4_RNA_ExpSam	RNA	GEO	GSM921812
CA04M001-R	CA04M001_10^3_2d_1_RNA_ExpSam	RNA	GEO	GSM921813
CA04M001-R	CA04M001_10^3_2d_2_RNA_ExpSam	RNA	GEO	GSM921814
CA04M001-R	CA04M001_10^3_2d_3_RNA_ExpSam	RNA	GEO	GSM921815
CA04M001-R	CA04M001_10^3_4d_1_RNA_ExpSam	RNA	GEO	GSM921816
CA04M001-R	CA04M001_10^3_4d_2_RNA_ExpSam	RNA	GEO	GSM921817
CA04M001-R	CA04M001_10^3_4d_3_RNA_ExpSam	RNA	GEO	GSM921818
CA04M001-R	CA04M001_10^3_4d_4_RNA_ExpSam	RNA	GEO	GSM921819
CA04M001-R	CA04M001_10^3_7d_2_RNA_ExpSam	RNA	GEO	GSM921820
CA04M001-R	CA04M001_10^3_7d_3_RNA_ExpSam	RNA	GEO	GSM921821
CA04M001-R	CA04M001_10^3_7d_4_RNA_ExpSam	RNA	GEO	GSM921822
CA04M001-R	CA04M001_10^4_1d_1_RNA_ExpSam	RNA	GEO	GSM921823
CA04M001-R	CA04M001_10^4_1d_2_RNA_ExpSam	RNA	GEO	GSM921824
CA04M001-R	CA04M001_10^4_1d_3_RNA_ExpSam	RNA	GEO	GSM921825
CA04M001-R	CA04M001_10^4_1d_4_RNA_ExpSam	RNA	GEO	GSM921826
CA04M001-R	CA04M001_10^4_2d_1_RNA_ExpSam	RNA	GEO	GSM921827
CA04M001-R	CA04M001_10^4_2d_2_RNA_ExpSam	RNA	GEO	GSM921828
CA04M001-R	CA04M001_10^4_2d_3_RNA_ExpSam	RNA	GEO	GSM921829
CA04M001-R	CA04M001_10^4_2d_4_RNA_ExpSam	RNA	GEO	GSM921830
CA04M001-R	CA04M001_10^4_4d_1_RNA_ExpSam	RNA	GEO	GSM921831
CA04M001-R	CA04M001_10^4_4d_2_RNA_ExpSam	RNA	GEO	GSM921832
CA04M001-R	CA04M001_10^4_4d_3_RNA_ExpSam	RNA	GEO	GSM921833
CA04M001-R	CA04M001_10^4_7d_1_RNA_ExpSam	RNA	GEO	GSM921834
CA04M001-R	CA04M001_10^4_7d_2_RNA_ExpSam	RNA	GEO	GSM921835
CA04M001-R	CA04M001_10^4_7d_3_RNA_ExpSam	RNA	GEO	GSM921836
CA04M001-R	CA04M001_10^5_1d_1_RNA_ExpSam	RNA	GEO	GSM921837
CA04M001-R	CA04M001_10^5_1d_2_RNA_ExpSam	RNA	GEO	GSM921838
CA04M001-R	CA04M001_10^5_1d_3_RNA_ExpSam	RNA	GEO	GSM921839
CA04M001-R	CA04M001_10^5_1d_4_RNA_ExpSam	RNA	GEO	GSM921840
CA04M001-R	CA04M001_10^5_2d_1_RNA_ExpSam	RNA	GEO	GSM921841
CA04M001-R	CA04M001_10^5_2d_2_RNA_ExpSam	RNA	GEO	GSM921842
CA04M001-R	CA04M001_10^5_2d_3_RNA_ExpSam	RNA	GEO	GSM921843
CA04M001-R	CA04M001_10^5_2d_4_RNA_ExpSam	RNA	GEO	GSM921844
CA04M001-R	CA04M001_10^5_4d_1_RNA_ExpSam	RNA	GEO	GSM921845
CA04M001-R	CA04M001_10^5_4d_2_RNA_ExpSam	RNA	GEO	GSM921846
CA04M001-R	CA04M001_10^5_4d_3_RNA_ExpSam	RNA	GEO	GSM921847
CA04M001-R	CA04M001_10^5_4d_4_RNA_ExpSam	RNA	GEO	GSM921848
CA04M001-R	CA04M001_10^5_7d_1_RNA_ExpSam	RNA	GEO	GSM921849
CA04M001-R	CA04M001_10^5_7d_2_RNA_ExpSam	RNA	GEO	GSM921850
CA04M001-R	CA04M001_10^5_7d_3_RNA_ExpSam	RNA	GEO	GSM921851
CA04M001-R	CA04M001_10^5_7d_4_RNA_ExpSam	RNA	GEO	GSM921852
CA04M001-R	CA04M001_10^5_7d_5_RNA_ExpSam	RNA	GEO	GSM921853
CA04M001-R	CA04M001_10^6_1d_1_RNA_ExpSam	RNA	GEO	GSM921854
CA04M001-R	CA04M001_10^6_1d_3_RNA_ExpSam	RNA	GEO	GSM921855
CA04M001-R	CA04M001_10^6_1d_4_RNA_ExpSam	RNA	GEO	GSM921856
CA04M001-R	CA04M001_10^6_2d_1_RNA_ExpSam	RNA	GEO	GSM921857
CA04M001-R	CA04M001_10^6_2d_2_RNA_ExpSam	RNA	GEO	GSM921858
CA04M001-R	CA04M001_10^6_2d_3_RNA_ExpSam	RNA	GEO	GSM921859
CA04M001-R	CA04M001_10^6_2d_4_RNA_ExpSam	RNA	GEO	GSM921860
CA04M001-R	CA04M001_10^6_4d_1_RNA_ExpSam	RNA	GEO	GSM921861
CA04M001-R	CA04M001_10^6_4d_2_RNA_ExpSam	RNA	GEO	GSM921862
CA04M001-R	CA04M001_10^6_4d_3_RNA_ExpSam	RNA	GEO	GSM921863
CA04M001-R	CA04M001_10^6_7d_1_RNA_ExpSam	RNA	GEO	GSM921864
CA04M001-R	CA04M001_10^6_7d_2_RNA_ExpSam	RNA	GEO	GSM921865
CA04M001-R	CA04M001_10^6_7d_3_RNA_ExpSam	RNA	GEO	GSM921866
CA04M001-R	CA04M001_10^6_7d_4_RNA_ExpSam	RNA	GEO	GSM921867
CA04M001-R	CA04M001_10^6_7d_5_RNA_ExpSam	RNA	GEO	GSM921868
CA04M001-R	CA04M001_mock_1d_1_RNA_ExpSam	RNA	GEO	GSM921869
CA04M001-R	CA04M001_mock_1d_2_RNA_ExpSam	RNA	GEO	GSM921870
CA04M001-R	CA04M001_mock_2d_1_RNA_ExpSam	RNA	GEO	GSM921871
CA04M001-R	CA04M001_mock_2d_2_RNA_ExpSam	RNA	GEO	GSM921872
CA04M001-R	CA04M001_mock_4d_1_RNA_ExpSam	RNA	GEO	GSM921873
CA04M001-R	CA04M001_mock_4d_2_RNA_ExpSam	RNA	GEO	GSM921874
CA04M001-R	CA04M001_mock_4d_3_RNA_ExpSam	RNA	GEO	GSM921875
CA04M001-R	CA04M001_mock_7d_1_RNA_ExpSam	RNA	GEO	GSM921876
CA04M001-R	CA04M001_mock_7d_2_RNA_ExpSam	RNA	GEO	GSM921877
CA04M001-R	CA04M001_mock_7d_3_RNA_ExpSam	RNA	GEO	GSM921878
ECL001-R	ECL001_EMC_0h_1_array	RNA	GEO	GSM1096530
ECL001-R	ECL001_EMC_0h_2_array	RNA	GEO	GSM1096515
ECL001-R	ECL001_EMC_0h_3_array	RNA	GEO	GSM1096512
ECL001-R	ECL001_EMC_12h_2_array	RNA	GEO	GSM1096529
ECL001-R	ECL001_EMC_12h_3_array	RNA	GEO	GSM1096526
ECL001-R	ECL001_EMC_18h_1_array	RNA	GEO	GSM1096540
ECL001-R	ECL001_EMC_18h_2_array	RNA	GEO	GSM1096536
ECL001-R	ECL001_EMC_18h_3_array	RNA	GEO	GSM1096532
ECL001-R	ECL001_EMC_24h_1_array	RNA	GEO	GSM1096528
ECL001-R	ECL001_EMC_24h_2_array	RNA	GEO	GSM1096513
ECL001-R	ECL001_EMC_24h_3_array	RNA	GEO	GSM1096519
ECL001-R	ECL001_EMC_3h_1_array	RNA	GEO	GSM1096521
ECL001-R	ECL001_EMC_3h_2_array	RNA	GEO	GSM1096517
ECL001-R	ECL001_EMC_3h_3_array	RNA	GEO	GSM1096542
ECL001-R	ECL001_EMC_7h_1_array	RNA	GEO	GSM1096541
ECL001-R	ECL001_EMC_7h_2_array	RNA	GEO	GSM1096518
ECL001-R	ECL001_EMC_7h_3_array	RNA	GEO	GSM1096535
ECL001-R	ECL001_Mock_0h_1_array	RNA	GEO	GSM1096531
ECL001-R	ECL001_Mock_0h_2_array	RNA	GEO	GSM1096514
ECL001-R	ECL001_Mock_0h_3_array	RNA	GEO	GSM1096534
ECL001-R	ECL001_Mock_12h_1_array	RNA	GEO	GSM1096511
ECL001-R	ECL001_Mock_12h_2_array	RNA	GEO	GSM1096533
ECL001-R	ECL001_Mock_12h_3_array	RNA	GEO	GSM1096522
ECL001-R	ECL001_Mock_24h_1_array	RNA	GEO	GSM1096525
ECL001-R	ECL001_Mock_24h_2_array	RNA	GEO	GSM1096524
ECL001-R	ECL001_Mock_24h_3_array	RNA	GEO	GSM1096520
ECL001-R	ECL001_Mock_3h_1_array	RNA	GEO	GSM1096538
ECL001-R	ECL001_Mock_3h_2_array	RNA	GEO	GSM1096516
ECL001-R	ECL001_Mock_3h_3_array	RNA	GEO	GSM1096539
ECL001-R	ECL001_Mock_7h_1_array	RNA	GEO	GSM1096537
ECL001-R	ECL001_Mock_7h_2_array	RNA	GEO	GSM1096523
ECL001-R	ECL001_Mock_7h_3_array	RNA	GEO	GSM1096527
ICL004-R	ICL004_Mock_0H_1_RNA_ExpSam	RNA	GEO	GSM697564
ICL004-R	ICL004_Mock_0H_2_RNA_ExpSam	RNA	GEO	GSM697565
ICL004-R	ICL004_Mock_0H_3_RNA_ExpSam	RNA	GEO	GSM697566
ICL004-R	ICL004_Mock_12H_1_RNA_ExpSam	RNA	GEO	GSM697573
ICL004-R	ICL004_Mock_12H_2_RNA_ExpSam	RNA	GEO	GSM697574
ICL004-R	ICL004_Mock_12H_3_RNA_ExpSam	RNA	GEO	GSM697575
ICL004-R	ICL004_Mock_18H_1_RNA_ExpSam	RNA	GEO	GSM697576
ICL004-R	ICL004_Mock_18H_2_RNA_ExpSam	RNA	GEO	GSM697577
ICL004-R	ICL004_Mock_18H_3_RNA_ExpSam	RNA	GEO	GSM697578
ICL004-R	ICL004_Mock_24H_1_RNA_ExpSam	RNA	GEO	GSM697579
ICL004-R	ICL004_Mock_24H_2_RNA_ExpSam	RNA	GEO	GSM697580
ICL004-R	ICL004_Mock_24H_3_RNA_ExpSam	RNA	GEO	GSM697581
ICL004-R	ICL004_Mock_3H_1_RNA_ExpSam	RNA	GEO	GSM697567
ICL004-R	ICL004_Mock_3H_2_RNA_ExpSam	RNA	GEO	GSM697568
ICL004-R	ICL004_Mock_3H_3_RNA_ExpSam	RNA	GEO	GSM697569
ICL004-R	ICL004_Mock_7H_1_RNA_ExpSam	RNA	GEO	GSM697570
ICL004-R	ICL004_Mock_7H_2_RNA_ExpSam	RNA	GEO	GSM697571
ICL004-R	ICL004_Mock_7H_3_RNA_ExpSam	RNA	GEO	GSM697572
ICL004-R	ICL004_VN1203_0H_1_RNA_ExpSam	RNA	GEO	GSM697582
ICL004-R	ICL004_VN1203_0H_2_RNA_ExpSam	RNA	GEO	GSM697583
ICL004-R	ICL004_VN1203_0H_3_RNA_ExpSam	RNA	GEO	GSM697584
ICL004-R	ICL004_VN1203_12H_1_RNA_ExpSam	RNA	GEO	GSM697591
ICL004-R	ICL004_VN1203_12H_2_RNA_ExpSam	RNA	GEO	GSM697592
ICL004-R	ICL004_VN1203_12H_3_RNA_ExpSam	RNA	GEO	GSM697593
ICL004-R	ICL004_VN1203_18H_1_RNA_ExpSam	RNA	GEO	GSM697594
ICL004-R	ICL004_VN1203_18H_2_RNA_ExpSam	RNA	GEO	GSM697595
ICL004-R	ICL004_VN1203_18H_3_RNA_ExpSam	RNA	GEO	GSM697596
ICL004-R	ICL004_VN1203_24H_1_RNA_ExpSam	RNA	GEO	GSM697597
ICL004-R	ICL004_VN1203_24H_2_RNA_ExpSam	RNA	GEO	GSM697598
ICL004-R	ICL004_VN1203_24H_3_RNA_ExpSam	RNA	GEO	GSM697599
ICL004-R	ICL004_VN1203_3H_1_RNA_ExpSam	RNA	GEO	GSM697585
ICL004-R	ICL004_VN1203_3H_2_RNA_ExpSam	RNA	GEO	GSM697586
ICL004-R	ICL004_VN1203_3H_3_RNA_ExpSam	RNA	GEO	GSM697587
ICL004-R	ICL004_VN1203_7H_1_RNA_ExpSam	RNA	GEO	GSM697588
ICL004-R	ICL004_VN1203_7H_2_RNA_ExpSam	RNA	GEO	GSM697589
ICL004-R	ICL004_VN1203_7H_3_RNA_ExpSam	RNA	GEO	GSM697590
ICL006-R	ICL006_CA04_0h_1_RNA_ExpSam	RNA	GEO	GSM921945
ICL006-R	ICL006_CA04_0h_2_RNA_ExpSam	RNA	GEO	GSM921944
ICL006-R	ICL006_CA04_0h_3_RNA_ExpSam	RNA	GEO	GSM921943
ICL006-R	ICL006_CA04_12h_1_RNA_ExpSam	RNA	GEO	GSM921942
ICL006-R	ICL006_CA04_12h_2_RNA_ExpSam	RNA	GEO	GSM921941
ICL006-R	ICL006_CA04_12h_3_RNA_ExpSam	RNA	GEO	GSM921940
ICL006-R	ICL006_CA04_18h_1_RNA_ExpSam	RNA	GEO	GSM921939
ICL006-R	ICL006_CA04_18h_2_RNA_ExpSam	RNA	GEO	GSM921938
ICL006-R	ICL006_CA04_18h_3_RNA_ExpSam	RNA	GEO	GSM921937
ICL006-R	ICL006_CA04_24h_1_RNA_ExpSam	RNA	GEO	GSM921936
ICL006-R	ICL006_CA04_24h_2_RNA_ExpSam	RNA	GEO	GSM921935
ICL006-R	ICL006_CA04_24h_3_RNA_ExpSam	RNA	GEO	GSM921934
ICL006-R	ICL006_CA04_30h_1_RNA_ExpSam	RNA	GEO	GSM921933
ICL006-R	ICL006_CA04_30h_2_RNA_ExpSam	RNA	GEO	GSM921932
ICL006-R	ICL006_CA04_30h_3_RNA_ExpSam	RNA	GEO	GSM921931
ICL006-R	ICL006_CA04_36h_1_RNA_ExpSam	RNA	GEO	GSM921930
ICL006-R	ICL006_CA04_36h_2_RNA_ExpSam	RNA	GEO	GSM921929
ICL006-R	ICL006_CA04_36h_3_RNA_ExpSam	RNA	GEO	GSM921928
ICL006-R	ICL006_CA04_3h_1_RNA_ExpSam	RNA	GEO	GSM921927
ICL006-R	ICL006_CA04_3h_2_RNA_ExpSam	RNA	GEO	GSM921926
ICL006-R	ICL006_CA04_3h_3_RNA_ExpSam	RNA	GEO	GSM921925
ICL006-R	ICL006_CA04_48h_1_RNA_ExpSam	RNA	GEO	GSM921924
ICL006-R	ICL006_CA04_48h_2_RNA_ExpSam	RNA	GEO	GSM921923
ICL006-R	ICL006_CA04_48h_3_RNA_ExpSam	RNA	GEO	GSM921922
ICL006-R	ICL006_CA04_7h_1_RNA_ExpSam	RNA	GEO	GSM921921
ICL006-R	ICL006_CA04_7h_2_RNA_ExpSam	RNA	GEO	GSM921920
ICL006-R	ICL006_CA04_7h_3_RNA_ExpSam	RNA	GEO	GSM921919
ICL006-R	ICL006_mock_0h_1_RNA_ExpSam	RNA	GEO	GSM921918
ICL006-R	ICL006_mock_0h_2_RNA_ExpSam	RNA	GEO	GSM921917
ICL006-R	ICL006_mock_0h_3_RNA_ExpSam	RNA	GEO	GSM921916
ICL006-R	ICL006_mock_12h_1_RNA_ExpSam	RNA	GEO	GSM921915
ICL006-R	ICL006_mock_12h_2_RNA_ExpSam	RNA	GEO	GSM921914
ICL006-R	ICL006_mock_12h_3_RNA_ExpSam	RNA	GEO	GSM921913
ICL006-R	ICL006_mock_18h_1_RNA_ExpSam	RNA	GEO	GSM921912
ICL006-R	ICL006_mock_18h_2_RNA_ExpSam	RNA	GEO	GSM921911
ICL006-R	ICL006_mock_18h_3_RNA_ExpSam	RNA	GEO	GSM921910
ICL006-R	ICL006_mock_24h_1_RNA_ExpSam	RNA	GEO	GSM921909
ICL006-R	ICL006_mock_24h_2_RNA_ExpSam	RNA	GEO	GSM921908
ICL006-R	ICL006_mock_24h_3_RNA_ExpSam	RNA	GEO	GSM921907
ICL006-R	ICL006_mock_30h_1_RNA_ExpSam	RNA	GEO	GSM921906
ICL006-R	ICL006_mock_30h_2_RNA_ExpSam	RNA	GEO	GSM921905
ICL006-R	ICL006_mock_30h_3_RNA_ExpSam	RNA	GEO	GSM921904
ICL006-R	ICL006_mock_36h_1_RNA_ExpSam	RNA	GEO	GSM921903
ICL006-R	ICL006_mock_36h_2_RNA_ExpSam	RNA	GEO	GSM921902
ICL006-R	ICL006_mock_36h_3_RNA_ExpSam	RNA	GEO	GSM921901
ICL006-R	ICL006_mock_3h_1_RNA_ExpSam	RNA	GEO	GSM921900
ICL006-R	ICL006_mock_3h_2_RNA_ExpSam	RNA	GEO	GSM921899
ICL006-R	ICL006_mock_3h_3_RNA_ExpSam	RNA	GEO	GSM921898
ICL006-R	ICL006_mock_48h_1_RNA_ExpSam	RNA	GEO	GSM921897
ICL006-R	ICL006_mock_48h_2_RNA_ExpSam	RNA	GEO	GSM921896
ICL006-R	ICL006_mock_48h_3_RNA_ExpSam	RNA	GEO	GSM921895
ICL006-R	ICL006_mock_7h_1_RNA_ExpSam	RNA	GEO	GSM921894
ICL006-R	ICL006_mock_7h_2_RNA_ExpSam	RNA	GEO	GSM921893
ICL006-R	ICL006_mock_7h_3_RNA_ExpSam	RNA	GEO	GSM921892
ICL010-R	ICL010_Cal_0h_1_array	RNA	GEO	GSM1003148
ICL010-R	ICL010_Cal_0h_2_array	RNA	GEO	GSM1003149
ICL010-R	ICL010_Cal_0h_3_array	RNA	GEO	GSM1003150
ICL010-R	ICL010_Cal_12h_1_array	RNA	GEO	GSM1003151
ICL010-R	ICL010_Cal_12h_2_array	RNA	GEO	GSM1003152
ICL010-R	ICL010_Cal_12h_3_array	RNA	GEO	GSM1003153
ICL010-R	ICL010_Cal_24h_1_array	RNA	GEO	GSM1003154
ICL010-R	ICL010_Cal_24h_2_array	RNA	GEO	GSM1003155
ICL010-R	ICL010_Cal_24h_3_array	RNA	GEO	GSM1003156
ICL010-R	ICL010_Cal_48h_1_array	RNA	GEO	GSM1003157
ICL010-R	ICL010_Cal_48h_2_array	RNA	GEO	GSM1003158
ICL010-R	ICL010_Cal_48h_3_array	RNA	GEO	GSM1003159
ICL010-R	ICL010_mock_0h_1_array	RNA	GEO	GSM1003160
ICL010-R	ICL010_mock_0h_2_array	RNA	GEO	GSM1003161
ICL010-R	ICL010_mock_0h_3_array	RNA	GEO	GSM1003162
ICL010-R	ICL010_mock_12h_1_array	RNA	GEO	GSM1003163
ICL010-R	ICL010_mock_12h_2_array	RNA	GEO	GSM1003164
ICL010-R	ICL010_mock_12h_3_array	RNA	GEO	GSM1003165
ICL010-R	ICL010_mock_18h_1_array	RNA	GEO	GSM1003166
ICL010-R	ICL010_mock_18h_2_array	RNA	GEO	GSM1003167
ICL010-R	ICL010_mock_18h_3_array	RNA	GEO	GSM1003168
ICL010-R	ICL010_mock_24h_1_array	RNA	GEO	GSM1003169
ICL010-R	ICL010_mock_24h_2_array	RNA	GEO	GSM1003170
ICL010-R	ICL010_mock_24h_3_array	RNA	GEO	GSM1003171
ICL010-R	ICL010_mock_30h_1_array	RNA	GEO	GSM1003172
ICL010-R	ICL010_mock_30h_2_array	RNA	GEO	GSM1003173
ICL010-R	ICL010_mock_30h_3_array	RNA	GEO	GSM1003174
ICL010-R	ICL010_mock_36h_1_array	RNA	GEO	GSM1003175
ICL010-R	ICL010_mock_36h_2_array	RNA	GEO	GSM1003176
ICL010-R	ICL010_mock_36h_3_array	RNA	GEO	GSM1003177
ICL010-R	ICL010_mock_3h_1_array	RNA	GEO	GSM1003178
ICL010-R	ICL010_mock_3h_2_array	RNA	GEO	GSM1003179
ICL010-R	ICL010_mock_3h_3_array	RNA	GEO	GSM1003180
ICL010-R	ICL010_mock_48h_1_array	RNA	GEO	GSM1003181
ICL010-R	ICL010_mock_48h_2_array	RNA	GEO	GSM1003182
ICL010-R	ICL010_mock_48h_3_array	RNA	GEO	GSM1003183
ICL010-R	ICL010_mock_7h_1_array	RNA	GEO	GSM1003184
ICL010-R	ICL010_mock_7h_2_array	RNA	GEO	GSM1003185
ICL010-R	ICL010_mock_7h_3_array	RNA	GEO	GSM1003186
ICL010-R	ICL010_NL_0h_1_array	RNA	GEO	GSM1003187
ICL010-R	ICL010_NL_0h_2_array	RNA	GEO	GSM1003188
ICL010-R	ICL010_NL_0h_3_array	RNA	GEO	GSM1003189
ICL010-R	ICL010_NL_12h_1_array	RNA	GEO	GSM1003190
ICL010-R	ICL010_NL_12h_2_array	RNA	GEO	GSM1003191
ICL010-R	ICL010_NL_12h_3_array	RNA	GEO	GSM1003192
ICL010-R	ICL010_NL_18h_1_array	RNA	GEO	GSM1003193
ICL010-R	ICL010_NL_18h_2_array	RNA	GEO	GSM1003194
ICL010-R	ICL010_NL_18h_3_array	RNA	GEO	GSM1003195
ICL010-R	ICL010_NL_24h_1_array	RNA	GEO	GSM1003196
ICL010-R	ICL010_NL_24h_2_array	RNA	GEO	GSM1003197
ICL010-R	ICL010_NL_24h_3_array	RNA	GEO	GSM1003198
ICL010-R	ICL010_NL_30h_1_array	RNA	GEO	GSM1003199
ICL010-R	ICL010_NL_30h_2_array	RNA	GEO	GSM1003200
ICL010-R	ICL010_NL_30h_3_array	RNA	GEO	GSM1003201
ICL010-R	ICL010_NL_36h_1_array	RNA	GEO	GSM1003202
ICL010-R	ICL010_NL_36h_2_array	RNA	GEO	GSM1003203
ICL010-R	ICL010_NL_36h_3_array	RNA	GEO	GSM1003204
ICL010-R	ICL010_NL_3h_1_array	RNA	GEO	GSM1003205
ICL010-R	ICL010_NL_3h_2_array	RNA	GEO	GSM1003206
ICL010-R	ICL010_NL_3h_3_array	RNA	GEO	GSM1003207
ICL010-R	ICL010_NL_48h_1_array	RNA	GEO	GSM1003208
ICL010-R	ICL010_NL_48h_2_array	RNA	GEO	GSM1003209
ICL010-R	ICL010_NL_48h_3_array	RNA	GEO	GSM1003210
ICL010-R	ICL010_NL_7h_1_array	RNA	GEO	GSM1003211
ICL010-R	ICL010_NL_7h_2_array	RNA	GEO	GSM1003212
ICL010-R	ICL010_NL_7h_3_array	RNA	GEO	GSM1003213
ICL011-R	ICL011_mock_0h_1_array	RNA	GEO	GSM1058227
ICL011-R	ICL011_mock_0h_2_array	RNA	GEO	GSM1058200
ICL011-R	ICL011_mock_0h_3_array	RNA	GEO	GSM1058202
ICL011-R	ICL011_mock_12h_1_array	RNA	GEO	GSM1058190
ICL011-R	ICL011_mock_12h_2_array	RNA	GEO	GSM1058217
ICL011-R	ICL011_mock_12h_3_array	RNA	GEO	GSM1058234
ICL011-R	ICL011_mock_18h_1_array	RNA	GEO	GSM1058207
ICL011-R	ICL011_mock_18h_2_array	RNA	GEO	GSM1058220
ICL011-R	ICL011_mock_18h_3_array	RNA	GEO	GSM1058193
ICL011-R	ICL011_mock_24h_1_array	RNA	GEO	GSM1058243
ICL011-R	ICL011_mock_24h_2_array	RNA	GEO	GSM1058203
ICL011-R	ICL011_mock_24h_3_array	RNA	GEO	GSM1058236
ICL011-R	ICL011_mock_3h_1_array	RNA	GEO	GSM1058232
ICL011-R	ICL011_mock_3h_2_array	RNA	GEO	GSM1058206
ICL011-R	ICL011_mock_3h_3_array	RNA	GEO	GSM1058228
ICL011-R	ICL011_mock_7h_1_array	RNA	GEO	GSM1058226
ICL011-R	ICL011_mock_7h_2_array	RNA	GEO	GSM1058197
ICL011-R	ICL011_mock_7h_3_array	RNA	GEO	GSM1058222
ICL011-R	ICL011_VN-PB1-F2-del_0h_1_array	RNA	GEO	GSM1058219
ICL011-R	ICL011_VN-PB1-F2-del_0h_2_array	RNA	GEO	GSM1058210
ICL011-R	ICL011_VN-PB1-F2-del_0h_3_array	RNA	GEO	GSM1058216
ICL011-R	ICL011_VN-PB1-F2-del_12h_1_array	RNA	GEO	GSM1058208
ICL011-R	ICL011_VN-PB1-F2-del_12h_2_array	RNA	GEO	GSM1058241
ICL011-R	ICL011_VN-PB1-F2-del_12h_3_array	RNA	GEO	GSM1058198
ICL011-R	ICL011_VN-PB1-F2-del_18h_1_array	RNA	GEO	GSM1058215
ICL011-R	ICL011_VN-PB1-F2-del_18h_2_array	RNA	GEO	GSM1058201
ICL011-R	ICL011_VN-PB1-F2-del_18h_3_array	RNA	GEO	GSM1058246
ICL011-R	ICL011_VN-PB1-F2-del_24h_1_array	RNA	GEO	GSM1058240
ICL011-R	ICL011_VN-PB1-F2-del_24h_2_array	RNA	GEO	GSM1058230
ICL011-R	ICL011_VN-PB1-F2-del_24h_3_array	RNA	GEO	GSM1058209
ICL011-R	ICL011_VN-PB1-F2-del_3h_1_array	RNA	GEO	GSM1058229
ICL011-R	ICL011_VN-PB1-F2-del_3h_2_array	RNA	GEO	GSM1058245
ICL011-R	ICL011_VN-PB1-F2-del_3h_3_array	RNA	GEO	GSM1058213
ICL011-R	ICL011_VN-PB1-F2-del_7h_1_array	RNA	GEO	GSM1058242
ICL011-R	ICL011_VN-PB1-F2-del_7h_2_array	RNA	GEO	GSM1058199
ICL011-R	ICL011_VN-PB1-F2-del_7h_3_array	RNA	GEO	GSM1058225
ICL011-R	ICL011_VN-PB2-627E_0h_1_array	RNA	GEO	GSM1058189
ICL011-R	ICL011_VN-PB2-627E_0h_2_array	RNA	GEO	GSM1058223
ICL011-R	ICL011_VN-PB2-627E_0h_3_array	RNA	GEO	GSM1058237
ICL011-R	ICL011_VN-PB2-627E_12h_1_array	RNA	GEO	GSM1058211
ICL011-R	ICL011_VN-PB2-627E_12h_2_array	RNA	GEO	GSM1058191
ICL011-R	ICL011_VN-PB2-627E_12h_3_array	RNA	GEO	GSM1058187
ICL011-R	ICL011_VN-PB2-627E_18h_1_array	RNA	GEO	GSM1058224
ICL011-R	ICL011_VN-PB2-627E_18h_2_array	RNA	GEO	GSM1058188
ICL011-R	ICL011_VN-PB2-627E_18h_3_array	RNA	GEO	GSM1058212
ICL011-R	ICL011_VN-PB2-627E_24h_1_array	RNA	GEO	GSM1058218
ICL011-R	ICL011_VN-PB2-627E_24h_2_array	RNA	GEO	GSM1058233
ICL011-R	ICL011_VN-PB2-627E_24h_3_array	RNA	GEO	GSM1058214
ICL011-R	ICL011_VN-PB2-627E_3h_1_array	RNA	GEO	GSM1058195
ICL011-R	ICL011_VN-PB2-627E_3h_2_array	RNA	GEO	GSM1058205
ICL011-R	ICL011_VN-PB2-627E_3h_3_array	RNA	GEO	GSM1058221
ICL011-R	ICL011_VN-PB2-627E_7h_1_array	RNA	GEO	GSM1058196
ICL011-R	ICL011_VN-PB2-627E_7h_2_array	RNA	GEO	GSM1058231
ICL011-R	ICL011_VN-PB2-627E_7h_3_array	RNA	GEO	GSM1058204
ICL011-R	ICL011_VN1203_24h_1_array	RNA	GEO	GSM1058244
ICL011-R	ICL011_VN1203_24h_2_array	RNA	GEO	GSM1058235
ICL011-R	ICL011_VN1203_24h_3_array	RNA	GEO	GSM1058192
ICL011-R	ICL011_VN1203_7h_1_array	RNA	GEO	GSM1058194
ICL011-R	ICL011_VN1203_7h_2_array	RNA	GEO	GSM1058238
ICL011-R	ICL011_VN1203_7h_3_array	RNA	GEO	GSM1058239
ICL012-R	ICL012_mock_0h_2_array	RNA	GEO	GSM1058271
ICL012-R	ICL012_mock_0h_3_array	RNA	GEO	GSM1058285
ICL012-R	ICL012_mock_12h_1_array	RNA	GEO	GSM1058274
ICL012-R	ICL012_mock_12h_2_array	RNA	GEO	GSM1058269
ICL012-R	ICL012_mock_12h_3_array	RNA	GEO	GSM1058251
ICL012-R	ICL012_mock_18h_1_array	RNA	GEO	GSM1058255
ICL012-R	ICL012_mock_18h_2_array	RNA	GEO	GSM1058257
ICL012-R	ICL012_mock_18h_3_array	RNA	GEO	GSM1058260
ICL012-R	ICL012_mock_24h_1_array	RNA	GEO	GSM1058256
ICL012-R	ICL012_mock_24h_2_array	RNA	GEO	GSM1058283
ICL012-R	ICL012_mock_24h_3_array	RNA	GEO	GSM1058266
ICL012-R	ICL012_mock_3h_1_array	RNA	GEO	GSM1058263
ICL012-R	ICL012_mock_3h_2_array	RNA	GEO	GSM1058253
ICL012-R	ICL012_mock_3h_3_array	RNA	GEO	GSM1058259
ICL012-R	ICL012_mock_7h_1_array	RNA	GEO	GSM1058279
ICL012-R	ICL012_mock_7h_2_array	RNA	GEO	GSM1058262
ICL012-R	ICL012_mock_7h_3_array	RNA	GEO	GSM1058254
ICL012-R	ICL012_NS1trunc_0h_1_array	RNA	GEO	GSM1058280
ICL012-R	ICL012_NS1trunc_0h_2_array	RNA	GEO	GSM1058258
ICL012-R	ICL012_NS1trunc_0h_3_array	RNA	GEO	GSM1058273
ICL012-R	ICL012_NS1trunc_12h_1_array	RNA	GEO	GSM1058284
ICL012-R	ICL012_NS1trunc_12h_2_array	RNA	GEO	GSM1058265
ICL012-R	ICL012_NS1trunc_12h_3_array	RNA	GEO	GSM1058261
ICL012-R	ICL012_NS1trunc_18h_1_array	RNA	GEO	GSM1058282
ICL012-R	ICL012_NS1trunc_18h_2_array	RNA	GEO	GSM1058286
ICL012-R	ICL012_NS1trunc_18h_3_array	RNA	GEO	GSM1058278
ICL012-R	ICL012_NS1trunc_24h_1_array	RNA	GEO	GSM1058275
ICL012-R	ICL012_NS1trunc_24h_2_array	RNA	GEO	GSM1058270
ICL012-R	ICL012_NS1trunc_24h_3_array	RNA	GEO	GSM1058267
ICL012-R	ICL012_NS1trunc_3h_1_array	RNA	GEO	GSM1058268
ICL012-R	ICL012_NS1trunc_3h_2_array	RNA	GEO	GSM1058272
ICL012-R	ICL012_NS1trunc_3h_3_array	RNA	GEO	GSM1058248
ICL012-R	ICL012_NS1trunc_7h_1_array	RNA	GEO	GSM1058276
ICL012-R	ICL012_NS1trunc_7h_2_array	RNA	GEO	GSM1058264
ICL012-R	ICL012_NS1trunc_7h_3_array	RNA	GEO	GSM1058249
ICL012-R	ICL012_VN1203_24h_1_array	RNA	GEO	GSM1058252
ICL012-R	ICL012_VN1203_24h_2_array	RNA	GEO	GSM1058250
ICL012-R	ICL012_VN1203_24h_3_array	RNA	GEO	GSM1058277
ICL012-R	ICL012_VN1203_7h_1_array	RNA	GEO	GSM1058281
ICL012-R	ICL012_VN1203_7h_2_array	RNA	GEO	GSM1058247
ICL012-R	ICL012_VN1203_7h_3_array	RNA	GEO	GSM1058287
IM001-R	IM001_Mock_D1_1_RNA_ExpSam	RNA	GEO	GSM822968
IM001-R	IM001_Mock_D1_2_RNA_ExpSam	RNA	GEO	GSM822969
IM001-R	IM001_Mock_D1_3_RNA_ExpSam	RNA	GEO	GSM822970
IM001-R	IM001_Mock_D2_1_RNA_ExpSam	RNA	GEO	GSM822971
IM001-R	IM001_Mock_D2_2_RNA_ExpSam	RNA	GEO	GSM822972
IM001-R	IM001_Mock_D2_3_RNA_ExpSam	RNA	GEO	GSM822973
IM001-R	IM001_Mock_D4_1_RNA_ExpSam	RNA	GEO	GSM822974
IM001-R	IM001_Mock_D4_2_RNA_ExpSam	RNA	GEO	GSM822975
IM001-R	IM001_Mock_D4_3_RNA_ExpSam	RNA	GEO	GSM822976
IM001-R	IM001_Mock_D7_1_RNA_ExpSam	RNA	GEO	GSM822977
IM001-R	IM001_Mock_D7_2_RNA_ExpSam	RNA	GEO	GSM822978
IM001-R	IM001_Mock_D7_3_RNA_ExpSam	RNA	GEO	GSM822979
IM001-R	IM001_VN1203_10^2pfu_D1_1_RNA_ExpSam	RNA	GEO	GSM822980
IM001-R	IM001_VN1203_10^2pfu_D1_2_RNA_ExpSam	RNA	GEO	GSM822981
IM001-R	IM001_VN1203_10^2pfu_D1_3_RNA_ExpSam	RNA	GEO	GSM822982
IM001-R	IM001_VN1203_10^2pfu_D1_4_RNA_ExpSam	RNA	GEO	GSM822983
IM001-R	IM001_VN1203_10^2pfu_D1_5_RNA_ExpSam	RNA	GEO	GSM822984
IM001-R	IM001_VN1203_10^2pfu_D2_1_RNA_ExpSam	RNA	GEO	GSM822985
IM001-R	IM001_VN1203_10^2pfu_D2_2_RNA_ExpSam	RNA	GEO	GSM822986
IM001-R	IM001_VN1203_10^2pfu_D2_3_RNA_ExpSam	RNA	GEO	GSM822987
IM001-R	IM001_VN1203_10^2pfu_D2_4_RNA_ExpSam	RNA	GEO	GSM822988
IM001-R	IM001_VN1203_10^2pfu_D2_5_RNA_ExpSam	RNA	GEO	GSM822989
IM001-R	IM001_VN1203_10^2pfu_D4_1_RNA_ExpSam	RNA	GEO	GSM822990
IM001-R	IM001_VN1203_10^2pfu_D4_2_RNA_ExpSam	RNA	GEO	GSM822991
IM001-R	IM001_VN1203_10^2pfu_D4_3_RNA_ExpSam	RNA	GEO	GSM822992
IM001-R	IM001_VN1203_10^2pfu_D4_4_RNA_ExpSam	RNA	GEO	GSM822993
IM001-R	IM001_VN1203_10^2pfu_D4_5_RNA_ExpSam	RNA	GEO	GSM822994
IM001-R	IM001_VN1203_10^2pfu_D7_1_RNA_ExpSam	RNA	GEO	GSM822995
IM001-R	IM001_VN1203_10^2pfu_D7_2_RNA_ExpSam	RNA	GEO	GSM822996
IM001-R	IM001_VN1203_10^2pfu_D7_3_RNA_ExpSam	RNA	GEO	GSM822997
IM001-R	IM001_VN1203_10^2pfu_D7_4_RNA_ExpSam	RNA	GEO	GSM822998
IM001-R	IM001_VN1203_10^3pfu_D1_1_RNA_ExpSam	RNA	GEO	GSM822999
IM001-R	IM001_VN1203_10^3pfu_D1_2_RNA_ExpSam	RNA	GEO	GSM823000
IM001-R	IM001_VN1203_10^3pfu_D1_3_RNA_ExpSam	RNA	GEO	GSM823001
IM001-R	IM001_VN1203_10^3pfu_D1_4_RNA_ExpSam	RNA	GEO	GSM823002
IM001-R	IM001_VN1203_10^3pfu_D1_5_RNA_ExpSam	RNA	GEO	GSM823003
IM001-R	IM001_VN1203_10^3pfu_D2_1_RNA_ExpSam	RNA	GEO	GSM823004
IM001-R	IM001_VN1203_10^3pfu_D2_2_RNA_ExpSam	RNA	GEO	GSM823005
IM001-R	IM001_VN1203_10^3pfu_D2_3_RNA_ExpSam	RNA	GEO	GSM823006
IM001-R	IM001_VN1203_10^3pfu_D2_4_RNA_ExpSam	RNA	GEO	GSM823007
IM001-R	IM001_VN1203_10^3pfu_D2_5_RNA_ExpSam	RNA	GEO	GSM823008
IM001-R	IM001_VN1203_10^3pfu_D4_1_RNA_ExpSam	RNA	GEO	GSM823009
IM001-R	IM001_VN1203_10^3pfu_D4_2_RNA_ExpSam	RNA	GEO	GSM823010
IM001-R	IM001_VN1203_10^3pfu_D4_3_RNA_ExpSam	RNA	GEO	GSM823011
IM001-R	IM001_VN1203_10^3pfu_D4_4_RNA_ExpSam	RNA	GEO	GSM823012
IM001-R	IM001_VN1203_10^3pfu_D4_5_RNA_ExpSam	RNA	GEO	GSM823013
IM001-R	IM001_VN1203_10^3pfu_D7_1_RNA_ExpSam	RNA	GEO	GSM823014
IM001-R	IM001_VN1203_10^3pfu_D7_2_RNA_ExpSam	RNA	GEO	GSM823015
IM001-R	IM001_VN1203_10^3pfu_D7_3_RNA_ExpSam	RNA	GEO	GSM823016
IM001-R	IM001_VN1203_10^4pfu_D1_1_RNA_ExpSam	RNA	GEO	GSM823017
IM001-R	IM001_VN1203_10^4pfu_D1_2_RNA_ExpSam	RNA	GEO	GSM823018
IM001-R	IM001_VN1203_10^4pfu_D1_3_RNA_ExpSam	RNA	GEO	GSM823019
IM001-R	IM001_VN1203_10^4pfu_D1_4_RNA_ExpSam	RNA	GEO	GSM823020
IM001-R	IM001_VN1203_10^4pfu_D1_5_RNA_ExpSam	RNA	GEO	GSM823021
IM001-R	IM001_VN1203_10^4pfu_D2_1_RNA_ExpSam	RNA	GEO	GSM823022
IM001-R	IM001_VN1203_10^4pfu_D2_2_RNA_ExpSam	RNA	GEO	GSM823023
IM001-R	IM001_VN1203_10^4pfu_D2_3_RNA_ExpSam	RNA	GEO	GSM823024
IM001-R	IM001_VN1203_10^4pfu_D2_4_RNA_ExpSam	RNA	GEO	GSM823025
IM001-R	IM001_VN1203_10^4pfu_D2_5_RNA_ExpSam	RNA	GEO	GSM823026
IM001-R	IM001_VN1203_10^4pfu_D4_1_RNA_ExpSam	RNA	GEO	GSM823027
IM001-R	IM001_VN1203_10^4pfu_D4_2_RNA_ExpSam	RNA	GEO	GSM823028
IM001-R	IM001_VN1203_10^4pfu_D4_3_RNA_ExpSam	RNA	GEO	GSM823029
IM001-R	IM001_VN1203_10^4pfu_D4_4_RNA_ExpSam	RNA	GEO	GSM823030
IM001-R	IM001_VN1203_10^4pfu_D4_5_RNA_ExpSam	RNA	GEO	GSM823031
IM002-R	IM002_Brisbane_10^6pfu_D1_2_array	RNA	GEO	GSM888961
IM002-R	IM002_Brisbane_10^6pfu_D1_3_array	RNA	GEO	GSM888973
IM002-R	IM002_Brisbane_10^6pfu_D3_2_array	RNA	GEO	GSM888958
IM002-R	IM002_Brisbane_10^6pfu_D3_3_array	RNA	GEO	GSM888965
IM002-R	IM002_Brisbane_10^6pfu_D5_1_array	RNA	GEO	GSM888966
IM002-R	IM002_Brisbane_10^6pfu_D5_2_array	RNA	GEO	GSM888968
IM002-R	IM002_Brisbane_10^6pfu_D5_3_array	RNA	GEO	GSM888975
IM002-R	IM002_Mex_10^6pfu_D1_1_array	RNA	GEO	GSM888960
IM002-R	IM002_Mex_10^6pfu_D1_2_array	RNA	GEO	GSM888982
IM002-R	IM002_Mex_10^6pfu_D1_3_array	RNA	GEO	GSM888978
IM002-R	IM002_Mex_10^6pfu_D3_1_array	RNA	GEO	GSM888992
IM002-R	IM002_Mex_10^6pfu_D3_2_array	RNA	GEO	GSM888989
IM002-R	IM002_Mex_10^6pfu_D3_3_array	RNA	GEO	GSM888988
IM002-R	IM002_Mex_10^6pfu_D5_1_array	RNA	GEO	GSM888972
IM002-R	IM002_Mex_10^6pfu_D5_2_array	RNA	GEO	GSM888964
IM002-R	IM002_Mex_10^6pfu_D5_3_array	RNA	GEO	GSM888983
IM002-R	IM002_Mock_D1_1_array	RNA	GEO	GSM888979
IM002-R	IM002_Mock_D1_2_array	RNA	GEO	GSM888976
IM002-R	IM002_Mock_D3_1_array	RNA	GEO	GSM888956
IM002-R	IM002_Mock_D3_2_array	RNA	GEO	GSM888981
IM002-R	IM002_Mock_D5_2_array	RNA	GEO	GSM888991
IM002-R	IM002_NJ_10^6pfu_D1_1_array	RNA	GEO	GSM888962
IM002-R	IM002_NJ_10^6pfu_D1_3_array	RNA	GEO	GSM888990
IM002-R	IM002_NJ_10^6pfu_D3_1_array	RNA	GEO	GSM888984
IM002-R	IM002_NJ_10^6pfu_D3_2_array	RNA	GEO	GSM888959
IM002-R	IM002_NJ_10^6pfu_D3_3_array	RNA	GEO	GSM888969
IM002-R	IM002_NJ_10^6pfu_D5_2_array	RNA	GEO	GSM888967
IM002-R	IM002_NJ_10^6pfu_D5_3_array	RNA	GEO	GSM888971
IM002-R	IM002_r1918_10^6pfu_D1_1_array	RNA	GEO	GSM888974
IM002-R	IM002_r1918_10^6pfu_D1_2_array	RNA	GEO	GSM888986
IM002-R	IM002_r1918_10^6pfu_D1_3_array	RNA	GEO	GSM888957
IM002-R	IM002_r1918_10^6pfu_D3_1_array	RNA	GEO	GSM888985
IM002-R	IM002_r1918_10^6pfu_D3_2_array	RNA	GEO	GSM888980
IM002-R	IM002_r1918_10^6pfu_D3_3_array	RNA	GEO	GSM888955
IM002-R	IM002_r1918_10^6pfu_D5_1_array	RNA	GEO	GSM888963
IM002-R	IM002_r1918_10^6pfu_D5_2_array	RNA	GEO	GSM888970
IM002-R	IM002_r1918_10^6pfu_D5_3_array	RNA	GEO	GSM888977
IM004-R	IM004_HAavirulent_10^4pfu_1d_1_RNA_ExpSam	RNA	GEO	GSM921978
IM004-R	IM004_HAavirulent_10^4pfu_1d_2_RNA_ExpSam	RNA	GEO	GSM921977
IM004-R	IM004_HAavirulent_10^4pfu_1d_3_RNA_ExpSam	RNA	GEO	GSM921976
IM004-R	IM004_HAavirulent_10^4pfu_1d_4_RNA_ExpSam	RNA	GEO	GSM921975
IM004-R	IM004_HAavirulent_10^4pfu_1d_5_RNA_ExpSam	RNA	GEO	GSM921974
IM004-R	IM004_HAavirulent_10^4pfu_2d_1_RNA_ExpSam	RNA	GEO	GSM921973
IM004-R	IM004_HAavirulent_10^4pfu_2d_2_RNA_ExpSam	RNA	GEO	GSM921972
IM004-R	IM004_HAavirulent_10^4pfu_2d_3_RNA_ExpSam	RNA	GEO	GSM921971
IM004-R	IM004_HAavirulent_10^4pfu_2d_4_RNA_ExpSam	RNA	GEO	GSM921970
IM004-R	IM004_HAavirulent_10^4pfu_2d_5_RNA_ExpSam	RNA	GEO	GSM921969
IM004-R	IM004_HAavirulent_10^4pfu_4d_1_RNA_ExpSam	RNA	GEO	GSM921968
IM004-R	IM004_HAavirulent_10^4pfu_4d_2_RNA_ExpSam	RNA	GEO	GSM921967
IM004-R	IM004_HAavirulent_10^4pfu_4d_3_RNA_ExpSam	RNA	GEO	GSM921966
IM004-R	IM004_HAavirulent_10^4pfu_4d_4_RNA_ExpSam	RNA	GEO	GSM921965
IM004-R	IM004_HAavirulent_10^4pfu_4d_5_RNA_ExpSam	RNA	GEO	GSM921964
IM004-R	IM004_HAavirulent_10^4pfu_7d_1_RNA_ExpSam	RNA	GEO	GSM921963
IM004-R	IM004_HAavirulent_10^4pfu_7d_2_RNA_ExpSam	RNA	GEO	GSM921962
IM004-R	IM004_HAavirulent_10^4pfu_7d_3_RNA_ExpSam	RNA	GEO	GSM921961
IM004-R	IM004_HAavirulent_10^4pfu_7d_4_RNA_ExpSam	RNA	GEO	GSM921960
IM004-R	IM004_HAavirulent_10^4pfu_7d_5_RNA_ExpSam	RNA	GEO	GSM921959
IM004-R	IM004_mock_1d_1_RNA_ExpSam	RNA	GEO	GSM921958
IM004-R	IM004_mock_1d_2_RNA_ExpSam	RNA	GEO	GSM921957
IM004-R	IM004_mock_1d_3_RNA_ExpSam	RNA	GEO	GSM921956
IM004-R	IM004_mock_2d_1_RNA_ExpSam	RNA	GEO	GSM921955
IM004-R	IM004_mock_2d_2_RNA_ExpSam	RNA	GEO	GSM921954
IM004-R	IM004_mock_2d_3_RNA_ExpSam	RNA	GEO	GSM921953
IM004-R	IM004_mock_4d_1_RNA_ExpSam	RNA	GEO	GSM921952
IM004-R	IM004_mock_4d_2_RNA_ExpSam	RNA	GEO	GSM921951
IM004-R	IM004_mock_4d_3_RNA_ExpSam	RNA	GEO	GSM921950
IM004-R	IM004_mock_7d_1_RNA_ExpSam	RNA	GEO	GSM921949
IM004-R	IM004_mock_7d_2_RNA_ExpSam	RNA	GEO	GSM921948
IM004-R	IM004_mock_7d_3_RNA_ExpSam	RNA	GEO	GSM921947
IM005-R	IM005_Mock_1d_1_array	RNA	GEO	GSM1060268
IM005-R	IM005_Mock_1d_2_array	RNA	GEO	GSM1060285
IM005-R	IM005_Mock_1d_3_array	RNA	GEO	GSM1060278
IM005-R	IM005_Mock_2d_1_array	RNA	GEO	GSM1060271
IM005-R	IM005_Mock_2d_2_array	RNA	GEO	GSM1060261
IM005-R	IM005_Mock_2d_3_array	RNA	GEO	GSM1060267
IM005-R	IM005_Mock_4d_1_array	RNA	GEO	GSM1060281
IM005-R	IM005_Mock_4d_2_array	RNA	GEO	GSM1060275
IM005-R	IM005_Mock_4d_3_array	RNA	GEO	GSM1060273
IM005-R	IM005_Mock_7d_1_array	RNA	GEO	GSM1060260
IM005-R	IM005_Mock_7d_2_array	RNA	GEO	GSM1060287
IM005-R	IM005_Mock_7d_3_array	RNA	GEO	GSM1060265
IM005-R	IM005_PB2_627E_10^4pfu_1d_1_array	RNA	GEO	GSM1060288
IM005-R	IM005_PB2_627E_10^4pfu_1d_2_array	RNA	GEO	GSM1060263
IM005-R	IM005_PB2_627E_10^4pfu_1d_3_array	RNA	GEO	GSM1060269
IM005-R	IM005_PB2_627E_10^4pfu_1d_4_array	RNA	GEO	GSM1060274
IM005-R	IM005_PB2_627E_10^4pfu_1d_5_array	RNA	GEO	GSM1060258
IM005-R	IM005_PB2_627E_10^4pfu_2d_1_array	RNA	GEO	GSM1060282
IM005-R	IM005_PB2_627E_10^4pfu_2d_2_array	RNA	GEO	GSM1060259
IM005-R	IM005_PB2_627E_10^4pfu_2d_3_array	RNA	GEO	GSM1060276
IM005-R	IM005_PB2_627E_10^4pfu_2d_4_array	RNA	GEO	GSM1060272
IM005-R	IM005_PB2_627E_10^4pfu_2d_5_array	RNA	GEO	GSM1060280
IM005-R	IM005_PB2_627E_10^4pfu_4d_1_array	RNA	GEO	GSM1060266
IM005-R	IM005_PB2_627E_10^4pfu_4d_2_array	RNA	GEO	GSM1060262
IM005-R	IM005_PB2_627E_10^4pfu_4d_3_array	RNA	GEO	GSM1060257
IM005-R	IM005_PB2_627E_10^4pfu_4d_4_array	RNA	GEO	GSM1060286
IM005-R	IM005_PB2_627E_10^4pfu_4d_5_array	RNA	GEO	GSM1060284
IM005-R	IM005_PB2_627E_10^4pfu_7d_1_array	RNA	GEO	GSM1060283
IM005-R	IM005_PB2_627E_10^4pfu_7d_2_array	RNA	GEO	GSM1060277
IM005-R	IM005_PB2_627E_10^4pfu_7d_3_array	RNA	GEO	GSM1060270
IM005-R	IM005_PB2_627E_10^4pfu_7d_4_array	RNA	GEO	GSM1060279
IM005-R	IM005_PB2_627E_10^4pfu_7d_5_array	RNA	GEO	GSM1060264
IM006A-R	IM006A_Mock_1d_1_array	RNA	GEO	GSM1060316
IM006A-R	IM006A_Mock_1d_2_array	RNA	GEO	GSM1060297
IM006A-R	IM006A_Mock_1d_3_array	RNA	GEO	GSM1060304
IM006A-R	IM006A_Mock_2d_1_array	RNA	GEO	GSM1060307
IM006A-R	IM006A_Mock_2d_2_array	RNA	GEO	GSM1060289
IM006A-R	IM006A_Mock_2d_3_array	RNA	GEO	GSM1060303
IM006A-R	IM006A_Mock_4d_1_array	RNA	GEO	GSM1060295
IM006A-R	IM006A_Mock_4d_2_array	RNA	GEO	GSM1060313
IM006A-R	IM006A_Mock_4d_3_array	RNA	GEO	GSM1060319
IM006A-R	IM006A_Mock_7d_1_array	RNA	GEO	GSM1060296
IM006A-R	IM006A_Mock_7d_2_array	RNA	GEO	GSM1060306
IM006A-R	IM006A_Mock_7d_3_array	RNA	GEO	GSM1060301
IM006A-R	IM006A_PB1_F2del_10^3pfu_1d_1_array	RNA	GEO	GSM1060299
IM006A-R	IM006A_PB1_F2del_10^3pfu_1d_2_array	RNA	GEO	GSM1060308
IM006A-R	IM006A_PB1_F2del_10^3pfu_1d_3_array	RNA	GEO	GSM1060291
IM006A-R	IM006A_PB1_F2del_10^3pfu_1d_4_array	RNA	GEO	GSM1060293
IM006A-R	IM006A_PB1_F2del_10^3pfu_1d_5_array	RNA	GEO	GSM1060310
IM006A-R	IM006A_PB1_F2del_10^3pfu_2d_1_array	RNA	GEO	GSM1060318
IM006A-R	IM006A_PB1_F2del_10^3pfu_2d_2_array	RNA	GEO	GSM1060312
IM006A-R	IM006A_PB1_F2del_10^3pfu_2d_3_array	RNA	GEO	GSM1060298
IM006A-R	IM006A_PB1_F2del_10^3pfu_2d_4_array	RNA	GEO	GSM1060294
IM006A-R	IM006A_PB1_F2del_10^3pfu_2d_5_array	RNA	GEO	GSM1060315
IM006A-R	IM006A_PB1_F2del_10^3pfu_4d_1_array	RNA	GEO	GSM1060300
IM006A-R	IM006A_PB1_F2del_10^3pfu_4d_2_array	RNA	GEO	GSM1060302
IM006A-R	IM006A_PB1_F2del_10^3pfu_4d_3_array	RNA	GEO	GSM1060314
IM006A-R	IM006A_PB1_F2del_10^3pfu_4d_4_array	RNA	GEO	GSM1060309
IM006A-R	IM006A_PB1_F2del_10^3pfu_4d_5_array	RNA	GEO	GSM1060290
IM006A-R	IM006A_PB1_F2del_10^3pfu_7d_1_array	RNA	GEO	GSM1060311
IM006A-R	IM006A_PB1_F2del_10^3pfu_7d_2_array	RNA	GEO	GSM1060305
IM006A-R	IM006A_PB1_F2del_10^3pfu_7d_3_array	RNA	GEO	GSM1060292
IM006A-R	IM006A_PB1_F2del_10^3pfu_7d_5_array	RNA	GEO	GSM1060317
IM006B-R	IM006B_Mock_1d_1_array	RNA	GEO	GSM1085373
IM006B-R	IM006B_Mock_1d_2_array	RNA	GEO	GSM1085385
IM006B-R	IM006B_Mock_1d_3_array	RNA	GEO	GSM1085396
IM006B-R	IM006B_Mock_2d_1_array	RNA	GEO	GSM1085387
IM006B-R	IM006B_Mock_2d_2_array	RNA	GEO	GSM1085379
IM006B-R	IM006B_Mock_2d_3_array	RNA	GEO	GSM1085389
IM006B-R	IM006B_Mock_4d_1_array	RNA	GEO	GSM1085384
IM006B-R	IM006B_Mock_4d_2_array	RNA	GEO	GSM1085376
IM006B-R	IM006B_Mock_4d_3_array	RNA	GEO	GSM1085391
IM006B-R	IM006B_Mock_7d_1_array	RNA	GEO	GSM1085393
IM006B-R	IM006B_Mock_7d_2_array	RNA	GEO	GSM1085382
IM006B-R	IM006B_PB1_F2del_10^4pfu_1d_1_array	RNA	GEO	GSM1085383
IM006B-R	IM006B_PB1_F2del_10^4pfu_1d_2_array	RNA	GEO	GSM1085397
IM006B-R	IM006B_PB1_F2del_10^4pfu_1d_3_array	RNA	GEO	GSM1085378
IM006B-R	IM006B_PB1_F2del_10^4pfu_1d_4_array	RNA	GEO	GSM1085380
IM006B-R	IM006B_PB1_F2del_10^4pfu_1d_5_array	RNA	GEO	GSM1085386
IM006B-R	IM006B_PB1_F2del_10^4pfu_2d_1_array	RNA	GEO	GSM1085392
IM006B-R	IM006B_PB1_F2del_10^4pfu_2d_2_array	RNA	GEO	GSM1085377
IM006B-R	IM006B_PB1_F2del_10^4pfu_2d_3_array	RNA	GEO	GSM1085374
IM006B-R	IM006B_PB1_F2del_10^4pfu_2d_4_array	RNA	GEO	GSM1085388
IM006B-R	IM006B_PB1_F2del_10^4pfu_2d_5_array	RNA	GEO	GSM1085381
IM006B-R	IM006B_PB1_F2del_10^4pfu_4d_1_array	RNA	GEO	GSM1085398
IM006B-R	IM006B_PB1_F2del_10^4pfu_4d_2_array	RNA	GEO	GSM1085395
IM006B-R	IM006B_PB1_F2del_10^4pfu_4d_3_array	RNA	GEO	GSM1085375
IM006B-R	IM006B_PB1_F2del_10^4pfu_4d_4_array	RNA	GEO	GSM1085394
IM006B-R	IM006B_PB1_F2del_10^4pfu_4d_5_array	RNA	GEO	GSM1085390
IM006B-R	IM006B_PB1_F2del_10^4pfu_7d_1_array	RNA	GEO	GSM1085372
IM007-R	IM007_Mock_1d_1_array	RNA	GEO	GSM1085555
IM007-R	IM007_Mock_1d_2_array	RNA	GEO	GSM1085571
IM007-R	IM007_Mock_1d_3_array	RNA	GEO	GSM1085545
IM007-R	IM007_Mock_2d_1_array	RNA	GEO	GSM1085564
IM007-R	IM007_Mock_2d_2_array	RNA	GEO	GSM1085558
IM007-R	IM007_Mock_2d_3_array	RNA	GEO	GSM1085547
IM007-R	IM007_Mock_4d_1_array	RNA	GEO	GSM1085590
IM007-R	IM007_Mock_4d_2_array	RNA	GEO	GSM1085551
IM007-R	IM007_Mock_4d_3_array	RNA	GEO	GSM1085562
IM007-R	IM007_Mock_7d_1_array	RNA	GEO	GSM1085567
IM007-R	IM007_Mock_7d_2_array	RNA	GEO	GSM1085539
IM007-R	IM007_Mock_7d_3_array	RNA	GEO	GSM1085542
IM007-R	IM007_NS1trunc124_10^3pfu_1d_1_array	RNA	GEO	GSM1085575
IM007-R	IM007_NS1trunc124_10^3pfu_1d_2_array	RNA	GEO	GSM1085568
IM007-R	IM007_NS1trunc124_10^3pfu_1d_3_array	RNA	GEO	GSM1085577
IM007-R	IM007_NS1trunc124_10^3pfu_1d_4_array	RNA	GEO	GSM1085559
IM007-R	IM007_NS1trunc124_10^3pfu_1d_5_array	RNA	GEO	GSM1085579
IM007-R	IM007_NS1trunc124_10^3pfu_2d_1_array	RNA	GEO	GSM1085580
IM007-R	IM007_NS1trunc124_10^3pfu_2d_2_array	RNA	GEO	GSM1085581
IM007-R	IM007_NS1trunc124_10^3pfu_2d_3_array	RNA	GEO	GSM1085572
IM007-R	IM007_NS1trunc124_10^3pfu_2d_4_array	RNA	GEO	GSM1085560
IM007-R	IM007_NS1trunc124_10^3pfu_2d_5_array	RNA	GEO	GSM1085552
IM007-R	IM007_NS1trunc124_10^3pfu_4d_1_array	RNA	GEO	GSM1085543
IM007-R	IM007_NS1trunc124_10^3pfu_4d_2_array	RNA	GEO	GSM1085553
IM007-R	IM007_NS1trunc124_10^3pfu_4d_3_array	RNA	GEO	GSM1085582
IM007-R	IM007_NS1trunc124_10^3pfu_4d_4_array	RNA	GEO	GSM1085583
IM007-R	IM007_NS1trunc124_10^3pfu_4d_5_array	RNA	GEO	GSM1085584
IM007-R	IM007_NS1trunc124_10^3pfu_7d_1_array	RNA	GEO	GSM1085574
IM007-R	IM007_NS1trunc124_10^3pfu_7d_2_array	RNA	GEO	GSM1085548
IM007-R	IM007_NS1trunc124_10^3pfu_7d_3_array	RNA	GEO	GSM1085566
IM007-R	IM007_NS1trunc124_10^3pfu_7d_4_array	RNA	GEO	GSM1085546
IM007-R	IM007_NS1trunc124_10^3pfu_7d_5_array	RNA	GEO	GSM1085563
IM007-R	IM007_NS1trunc124_10^4pfu_1d_1_array	RNA	GEO	GSM1085578
IM007-R	IM007_NS1trunc124_10^4pfu_1d_2_array	RNA	GEO	GSM1085550
IM007-R	IM007_NS1trunc124_10^4pfu_1d_3_array	RNA	GEO	GSM1085540
IM007-R	IM007_NS1trunc124_10^4pfu_1d_4_array	RNA	GEO	GSM1085557
IM007-R	IM007_NS1trunc124_10^4pfu_1d_5_array	RNA	GEO	GSM1085570
IM007-R	IM007_NS1trunc124_10^4pfu_2d_1_array	RNA	GEO	GSM1085549
IM007-R	IM007_NS1trunc124_10^4pfu_2d_2_array	RNA	GEO	GSM1085561
IM007-R	IM007_NS1trunc124_10^4pfu_2d_3_array	RNA	GEO	GSM1085554
IM007-R	IM007_NS1trunc124_10^4pfu_2d_4_array	RNA	GEO	GSM1085585
IM007-R	IM007_NS1trunc124_10^4pfu_2d_5_array	RNA	GEO	GSM1085586
IM007-R	IM007_NS1trunc124_10^4pfu_4d_1_array	RNA	GEO	GSM1085544
IM007-R	IM007_NS1trunc124_10^4pfu_4d_2_array	RNA	GEO	GSM1085556
IM007-R	IM007_NS1trunc124_10^4pfu_4d_3_array	RNA	GEO	GSM1085569
IM007-R	IM007_NS1trunc124_10^4pfu_4d_4_array	RNA	GEO	GSM1085541
IM007-R	IM007_NS1trunc124_10^4pfu_4d_5_array	RNA	GEO	GSM1085573
IM007-R	IM007_NS1trunc124_10^4pfu_7d_1_array	RNA	GEO	GSM1085565
IM007-R	IM007_NS1trunc124_10^4pfu_7d_2_array	RNA	GEO	GSM1085587
IM007-R	IM007_NS1trunc124_10^4pfu_7d_3_array	RNA	GEO	GSM1085588
IM007-R	IM007_NS1trunc124_10^4pfu_7d_4_array	RNA	GEO	GSM1085589
IM007-R	IM007_NS1trunc124_10^4pfu_7d_5_array	RNA	GEO	GSM1085576
IM009-R	IM009_CA04_10^6pfu_D1_1_array	RNA	GEO	GSM888953
IM009-R	IM009_CA04_10^6pfu_D1_2_array	RNA	GEO	GSM888936
IM009-R	IM009_CA04_10^6pfu_D1_3_array	RNA	GEO	GSM888950
IM009-R	IM009_CA04_10^6pfu_D3_1_array	RNA	GEO	GSM888947
IM009-R	IM009_CA04_10^6pfu_D3_2_array	RNA	GEO	GSM888940
IM009-R	IM009_CA04_10^6pfu_D3_3_array	RNA	GEO	GSM888942
IM009-R	IM009_CA04_10^6pfu_D5_1_array	RNA	GEO	GSM888937
IM009-R	IM009_CA04_10^6pfu_D5_2_array	RNA	GEO	GSM888929
IM009-R	IM009_CA04_10^6pfu_D5_3_array	RNA	GEO	GSM888931
IM009-R	IM009_MA-CA04_10^6pfu_D1_1_array	RNA	GEO	GSM888952
IM009-R	IM009_MA-CA04_10^6pfu_D1_2_array	RNA	GEO	GSM888943
IM009-R	IM009_MA-CA04_10^6pfu_D1_3_array	RNA	GEO	GSM888935
IM009-R	IM009_MA-CA04_10^6pfu_D3_1_array	RNA	GEO	GSM888946
IM009-R	IM009_MA-CA04_10^6pfu_D3_2_array	RNA	GEO	GSM888930
IM009-R	IM009_MA-CA04_10^6pfu_D3_3_array	RNA	GEO	GSM888945
IM009-R	IM009_MA-CA04_10^6pfu_D5_1_array	RNA	GEO	GSM888954
IM009-R	IM009_MA-CA04_10^6pfu_D5_2_array	RNA	GEO	GSM888939
IM009-R	IM009_MA-CA04_10^6pfu_D5_3_array	RNA	GEO	GSM888934
IM009-R	IM009_Mock_D1_1_array	RNA	GEO	GSM888948
IM009-R	IM009_Mock_D1_2_array	RNA	GEO	GSM888941
IM009-R	IM009_Mock_D1_3_array	RNA	GEO	GSM888933
IM009-R	IM009_Mock_D3_1_array	RNA	GEO	GSM888932
IM009-R	IM009_Mock_D3_2_array	RNA	GEO	GSM888951
IM009-R	IM009_Mock_D3_3_array	RNA	GEO	GSM888938
IM009-R	IM009_Mock_D5_1_array	RNA	GEO	GSM888944
IM009-R	IM009_Mock_D5_2_array	RNA	GEO	GSM888949
IM010-R	IM010_B6_Mock_2d_1_array	RNA	GEO	GSM1001726
IM010-R	IM010_B6_Mock_2d_2_array	RNA	GEO	GSM1001727
IM010-R	IM010_B6_Mock_2d_3_array	RNA	GEO	GSM1001728
IM010-R	IM010_B6_VN1203_2d_1_array	RNA	GEO	GSM1001729
IM010-R	IM010_B6_VN1203_2d_2_array	RNA	GEO	GSM1001730
IM010-R	IM010_B6_VN1203_2d_3_array	RNA	GEO	GSM1001731
IM010-R	IM010_IDO1_Mock_2d_1_array	RNA	GEO	GSM1001704
IM010-R	IM010_IDO1_Mock_2d_2_array	RNA	GEO	GSM1001705
IM010-R	IM010_IDO1_Mock_2d_3_array	RNA	GEO	GSM1001706
IM010-R	IM010_IDO1_Mock_6d_1_array	RNA	GEO	GSM1001707
IM010-R	IM010_IDO1_Mock_6d_2_array	RNA	GEO	GSM1001708
IM010-R	IM010_IDO1_Mock_6d_3_array	RNA	GEO	GSM1001709
IM010-R	IM010_IDO1_VN1203_2d_1_array	RNA	GEO	GSM1001710
IM010-R	IM010_IDO1_VN1203_2d_2_array	RNA	GEO	GSM1001711
IM010-R	IM010_IDO1_VN1203_2d_3_array	RNA	GEO	GSM1001712
IM010-R	IM010_IDO1_VN1203_6d_1_array	RNA	GEO	GSM1001713
IM010-R	IM010_IDO1_VN1203_6d_2_array	RNA	GEO	GSM1001714
IM010-R	IM010_Tnfrsf1b_Mock_2d_1_array	RNA	GEO	GSM1001715
IM010-R	IM010_Tnfrsf1b_Mock_2d_2_array	RNA	GEO	GSM1001716
IM010-R	IM010_Tnfrsf1b_Mock_2d_3_array	RNA	GEO	GSM1001717
IM010-R	IM010_Tnfrsf1b_Mock_6d_1_array	RNA	GEO	GSM1001718
IM010-R	IM010_Tnfrsf1b_Mock_6d_2_array	RNA	GEO	GSM1001719
IM010-R	IM010_Tnfrsf1b_Mock_6d_3_array	RNA	GEO	GSM1001720
IM010-R	IM010_Tnfrsf1b_VN1203_2d_1_array	RNA	GEO	GSM1001721
IM010-R	IM010_Tnfrsf1b_VN1203_2d_2_array	RNA	GEO	GSM1001722
IM010-R	IM010_Tnfrsf1b_VN1203_2d_3_array	RNA	GEO	GSM1001723
IM010-R	IM010_Tnfrsf1b_VN1203_6d_1_array	RNA	GEO	GSM1001724
IM010-R	IM010_Tnfrsf1b_VN1203_6d_2_array	RNA	GEO	GSM1001725
SBRI_AA_E1	AA_Mock_001	RNA	GEO	GSM914274
SBRI_AA_E1	AA_Mock_002	RNA	GEO	GSM914275
SBRI_AA_E1	AA_Mock_003	RNA	GEO	GSM914276
SBRI_AA_E1	AA_Mock_005	RNA	GEO	GSM914277
SBRI_AA_E1	AA_PR8_006	RNA	GEO	GSM914259
SBRI_AA_E1	AA_PR8_007	RNA	GEO	GSM914260
SBRI_AA_E1	AA_PR8_008	RNA	GEO	GSM914261
SBRI_AA_E1	AA_PR8_009	RNA	GEO	GSM914262
SBRI_AA_E1	AA_PR8_010	RNA	GEO	GSM914263
SBRI_AA_E1	AA_VN6+2_016	RNA	GEO	GSM914264
SBRI_AA_E1	AA_VN6+2_017	RNA	GEO	GSM914265
SBRI_AA_E1	AA_VN6+2_018	RNA	GEO	GSM914266
SBRI_AA_E1	AA_VN6+2_019	RNA	GEO	GSM914267
SBRI_AA_E1	AA_VN6+2_020	RNA	GEO	GSM914268
SBRI_AA_E1	AA_X31_011	RNA	GEO	GSM914269
SBRI_AA_E1	AA_X31_012	RNA	GEO	GSM914270
SBRI_AA_E1	AA_X31_013	RNA	GEO	GSM914271
SBRI_AA_E1	AA_X31_014	RNA	GEO	GSM914272
SBRI_AA_E1	AA_X31_015	RNA	GEO	GSM914273
SCL005-R	SCL005_DORF6_0H_1_RNA_ExpSam	RNA	GEO	GSM823243
SCL005-R	SCL005_DORF6_0H_2_RNA_ExpSam	RNA	GEO	GSM823244
SCL005-R	SCL005_DORF6_0H_3_RNA_ExpSam	RNA	GEO	GSM823245
SCL005-R	SCL005_DORF6_12H_1_RNA_ExpSam	RNA	GEO	GSM823252
SCL005-R	SCL005_DORF6_12H_2_RNA_ExpSam	RNA	GEO	GSM823253
SCL005-R	SCL005_DORF6_12H_3_RNA_ExpSam	RNA	GEO	GSM823254
SCL005-R	SCL005_DORF6_24H_1_RNA_ExpSam	RNA	GEO	GSM823255
SCL005-R	SCL005_DORF6_24H_2_RNA_ExpSam	RNA	GEO	GSM823256
SCL005-R	SCL005_DORF6_24H_3_RNA_ExpSam	RNA	GEO	GSM823257
SCL005-R	SCL005_DORF6_30H_1_RNA_ExpSam	RNA	GEO	GSM823258
SCL005-R	SCL005_DORF6_30H_2_RNA_ExpSam	RNA	GEO	GSM823259
SCL005-R	SCL005_DORF6_30H_3_RNA_ExpSam	RNA	GEO	GSM823260
SCL005-R	SCL005_DORF6_36H_1_RNA_ExpSam	RNA	GEO	GSM823261
SCL005-R	SCL005_DORF6_36H_2_RNA_ExpSam	RNA	GEO	GSM823262
SCL005-R	SCL005_DORF6_36H_3_RNA_ExpSam	RNA	GEO	GSM823263
SCL005-R	SCL005_DORF6_3H_1_RNA_ExpSam	RNA	GEO	GSM823246
SCL005-R	SCL005_DORF6_3H_2_RNA_ExpSam	RNA	GEO	GSM823247
SCL005-R	SCL005_DORF6_3H_3_RNA_ExpSam	RNA	GEO	GSM823248
SCL005-R	SCL005_DORF6_48H_1_RNA_ExpSam	RNA	GEO	GSM823264
SCL005-R	SCL005_DORF6_48H_2_RNA_ExpSam	RNA	GEO	GSM823265
SCL005-R	SCL005_DORF6_48H_3_RNA_ExpSam	RNA	GEO	GSM823266
SCL005-R	SCL005_DORF6_54H_1_RNA_ExpSam	RNA	GEO	GSM823267
SCL005-R	SCL005_DORF6_54H_2_RNA_ExpSam	RNA	GEO	GSM823268
SCL005-R	SCL005_DORF6_54H_3_RNA_ExpSam	RNA	GEO	GSM823269
SCL005-R	SCL005_DORF6_60H_1_RNA_ExpSam	RNA	GEO	GSM823270
SCL005-R	SCL005_DORF6_60H_2_RNA_ExpSam	RNA	GEO	GSM823271
SCL005-R	SCL005_DORF6_60H_3_RNA_ExpSam	RNA	GEO	GSM823272
SCL005-R	SCL005_DORF6_72H_1_RNA_ExpSam	RNA	GEO	GSM823273
SCL005-R	SCL005_DORF6_72H_2_RNA_ExpSam	RNA	GEO	GSM823274
SCL005-R	SCL005_DORF6_72H_3_RNA_ExpSam	RNA	GEO	GSM823275
SCL005-R	SCL005_DORF6_7H_1_RNA_ExpSam	RNA	GEO	GSM823249
SCL005-R	SCL005_DORF6_7H_2_RNA_ExpSam	RNA	GEO	GSM823250
SCL005-R	SCL005_DORF6_7H_3_RNA_ExpSam	RNA	GEO	GSM823251
SCL005-R	SCL005_mock_0H_1_RNA_ExpSam	RNA	GEO	GSM823177
SCL005-R	SCL005_mock_0H_2_RNA_ExpSam	RNA	GEO	GSM823178
SCL005-R	SCL005_mock_0H_3_RNA_ExpSam	RNA	GEO	GSM823179
SCL005-R	SCL005_mock_12H_1_RNA_ExpSam	RNA	GEO	GSM823186
SCL005-R	SCL005_mock_12H_2_RNA_ExpSam	RNA	GEO	GSM823187
SCL005-R	SCL005_mock_12H_3_RNA_ExpSam	RNA	GEO	GSM823188
SCL005-R	SCL005_mock_24H_1_RNA_ExpSam	RNA	GEO	GSM823189
SCL005-R	SCL005_mock_24H_2_RNA_ExpSam	RNA	GEO	GSM823190
SCL005-R	SCL005_mock_24H_3_RNA_ExpSam	RNA	GEO	GSM823191
SCL005-R	SCL005_mock_30H_1_RNA_ExpSam	RNA	GEO	GSM823192
SCL005-R	SCL005_mock_30H_2_RNA_ExpSam	RNA	GEO	GSM823193
SCL005-R	SCL005_mock_30H_3_RNA_ExpSam	RNA	GEO	GSM823194
SCL005-R	SCL005_mock_36H_1_RNA_ExpSam	RNA	GEO	GSM823195
SCL005-R	SCL005_mock_36H_2_RNA_ExpSam	RNA	GEO	GSM823196
SCL005-R	SCL005_mock_36H_3_RNA_ExpSam	RNA	GEO	GSM823197
SCL005-R	SCL005_mock_3H_1_RNA_ExpSam	RNA	GEO	GSM823180
SCL005-R	SCL005_mock_3H_2_RNA_ExpSam	RNA	GEO	GSM823181
SCL005-R	SCL005_mock_3H_3_RNA_ExpSam	RNA	GEO	GSM823182
SCL005-R	SCL005_mock_48H_1_RNA_ExpSam	RNA	GEO	GSM823198
SCL005-R	SCL005_mock_48H_2_RNA_ExpSam	RNA	GEO	GSM823199
SCL005-R	SCL005_mock_48H_3_RNA_ExpSam	RNA	GEO	GSM823200
SCL005-R	SCL005_mock_54H_1_RNA_ExpSam	RNA	GEO	GSM823201
SCL005-R	SCL005_mock_54H_2_RNA_ExpSam	RNA	GEO	GSM823202
SCL005-R	SCL005_mock_54H_3_RNA_ExpSam	RNA	GEO	GSM823203
SCL005-R	SCL005_mock_60H_1_RNA_ExpSam	RNA	GEO	GSM823204
SCL005-R	SCL005_mock_60H_2_RNA_ExpSam	RNA	GEO	GSM823205
SCL005-R	SCL005_mock_60H_3_RNA_ExpSam	RNA	GEO	GSM823206
SCL005-R	SCL005_mock_72H_1_RNA_ExpSam	RNA	GEO	GSM823207
SCL005-R	SCL005_mock_72H_2_RNA_ExpSam	RNA	GEO	GSM823208
SCL005-R	SCL005_mock_72H_3_RNA_ExpSam	RNA	GEO	GSM823209
SCL005-R	SCL005_mock_7H_1_RNA_ExpSam	RNA	GEO	GSM823183
SCL005-R	SCL005_mock_7H_2_RNA_ExpSam	RNA	GEO	GSM823184
SCL005-R	SCL005_mock_7H_3_RNA_ExpSam	RNA	GEO	GSM823185
SCL005-R	SCL005_WT_0H_1_RNA_ExpSam	RNA	GEO	GSM823210
SCL005-R	SCL005_WT_0H_2_RNA_ExpSam	RNA	GEO	GSM823211
SCL005-R	SCL005_WT_0H_3_RNA_ExpSam	RNA	GEO	GSM823212
SCL005-R	SCL005_WT_12H_1_RNA_ExpSam	RNA	GEO	GSM823219
SCL005-R	SCL005_WT_12H_2_RNA_ExpSam	RNA	GEO	GSM823220
SCL005-R	SCL005_WT_12H_3_RNA_ExpSam	RNA	GEO	GSM823221
SCL005-R	SCL005_WT_24H_1_RNA_ExpSam	RNA	GEO	GSM823222
SCL005-R	SCL005_WT_24H_2_RNA_ExpSam	RNA	GEO	GSM823223
SCL005-R	SCL005_WT_24H_3_RNA_ExpSam	RNA	GEO	GSM823224
SCL005-R	SCL005_WT_30H_1_RNA_ExpSam	RNA	GEO	GSM823225
SCL005-R	SCL005_WT_30H_2_RNA_ExpSam	RNA	GEO	GSM823226
SCL005-R	SCL005_WT_30H_3_RNA_ExpSam	RNA	GEO	GSM823227
SCL005-R	SCL005_WT_36H_1_RNA_ExpSam	RNA	GEO	GSM823228
SCL005-R	SCL005_WT_36H_2_RNA_ExpSam	RNA	GEO	GSM823229
SCL005-R	SCL005_WT_36H_3_RNA_ExpSam	RNA	GEO	GSM823230
SCL005-R	SCL005_WT_3H_1_RNA_ExpSam	RNA	GEO	GSM823213
SCL005-R	SCL005_WT_3H_2_RNA_ExpSam	RNA	GEO	GSM823214
SCL005-R	SCL005_WT_3H_3_RNA_ExpSam	RNA	GEO	GSM823215
SCL005-R	SCL005_WT_48H_1_RNA_ExpSam	RNA	GEO	GSM823231
SCL005-R	SCL005_WT_48H_2_RNA_ExpSam	RNA	GEO	GSM823232
SCL005-R	SCL005_WT_48H_3_RNA_ExpSam	RNA	GEO	GSM823233
SCL005-R	SCL005_WT_54H_1_RNA_ExpSam	RNA	GEO	GSM823234
SCL005-R	SCL005_WT_54H_2_RNA_ExpSam	RNA	GEO	GSM823235
SCL005-R	SCL005_WT_54H_3_RNA_ExpSam	RNA	GEO	GSM823236
SCL005-R	SCL005_WT_60H_1_RNA_ExpSam	RNA	GEO	GSM823237
SCL005-R	SCL005_WT_60H_2_RNA_ExpSam	RNA	GEO	GSM823238
SCL005-R	SCL005_WT_60H_3_RNA_ExpSam	RNA	GEO	GSM823239
SCL005-R	SCL005_WT_72H_1_RNA_ExpSam	RNA	GEO	GSM823240
SCL005-R	SCL005_WT_72H_2_RNA_ExpSam	RNA	GEO	GSM823241
SCL005-R	SCL005_WT_72H_3_RNA_ExpSam	RNA	GEO	GSM823242
SCL005-R	SCL005_WT_7H_1_RNA_ExpSam	RNA	GEO	GSM823216
SCL005-R	SCL005_WT_7H_2_RNA_ExpSam	RNA	GEO	GSM823217
SCL005-R	SCL005_WT_7H_3_RNA_ExpSam	RNA	GEO	GSM823218
SCL006-R	SCL006_BatSRBD_0h_1_RNA_ExpSam	RNA	GEO	GSM928559
SCL006-R	SCL006_BatSRBD_0h_2_RNA_ExpSam	RNA	GEO	GSM928560
SCL006-R	SCL006_BatSRBD_0h_3_RNA_ExpSam	RNA	GEO	GSM928561
SCL006-R	SCL006_BatSRBD_12h_1_RNA_ExpSam	RNA	GEO	GSM928562
SCL006-R	SCL006_BatSRBD_12h_2_RNA_ExpSam	RNA	GEO	GSM928563
SCL006-R	SCL006_BatSRBD_12h_3_RNA_ExpSam	RNA	GEO	GSM928564
SCL006-R	SCL006_BatSRBD_24h_1_RNA_ExpSam	RNA	GEO	GSM928565
SCL006-R	SCL006_BatSRBD_24h_2_RNA_ExpSam	RNA	GEO	GSM928566
SCL006-R	SCL006_BatSRBD_24h_3_RNA_ExpSam	RNA	GEO	GSM928567
SCL006-R	SCL006_BatSRBD_30h_1_RNA_ExpSam	RNA	GEO	GSM928568
SCL006-R	SCL006_BatSRBD_30h_2_RNA_ExpSam	RNA	GEO	GSM928569
SCL006-R	SCL006_BatSRBD_30h_3_RNA_ExpSam	RNA	GEO	GSM928570
SCL006-R	SCL006_BatSRBD_36h_1_RNA_ExpSam	RNA	GEO	GSM928571
SCL006-R	SCL006_BatSRBD_36h_2_RNA_ExpSam	RNA	GEO	GSM928572
SCL006-R	SCL006_BatSRBD_36h_3_RNA_ExpSam	RNA	GEO	GSM928573
SCL006-R	SCL006_BatSRBD_48h_1_B_RNA_ExpSam	RNA	GEO	GSM928574
SCL006-R	SCL006_BatSRBD_48h_2_RNA_ExpSam	RNA	GEO	GSM928575
SCL006-R	SCL006_BatSRBD_48h_3_RNA_ExpSam	RNA	GEO	GSM928576
SCL006-R	SCL006_BatSRBD_54h_1_B_RNA_ExpSam	RNA	GEO	GSM928577
SCL006-R	SCL006_BatSRBD_54h_2_RNA_ExpSam	RNA	GEO	GSM928578
SCL006-R	SCL006_BatSRBD_54h_3_RNA_ExpSam	RNA	GEO	GSM928579
SCL006-R	SCL006_BatSRBD_60h_1_RNA_ExpSam	RNA	GEO	GSM928580
SCL006-R	SCL006_BatSRBD_60h_2_RNA_ExpSam	RNA	GEO	GSM928581
SCL006-R	SCL006_BatSRBD_60h_3_RNA_ExpSam	RNA	GEO	GSM928582
SCL006-R	SCL006_BatSRBD_72h_1_RNA_ExpSam	RNA	GEO	GSM928583
SCL006-R	SCL006_BatSRBD_72h_2_RNA_ExpSam	RNA	GEO	GSM928584
SCL006-R	SCL006_BatSRBD_72h_3_RNA_ExpSam	RNA	GEO	GSM928585
SCL006-R	SCL006_icSARSCoV_0h_1_RNA_ExpSam	RNA	GEO	GSM928586
SCL006-R	SCL006_icSARSCoV_0h_2_RNA_ExpSam	RNA	GEO	GSM928587
SCL006-R	SCL006_icSARSCoV_0h_3_RNA_ExpSam	RNA	GEO	GSM928588
SCL006-R	SCL006_icSARSCoV_12h_1_RNA_ExpSam	RNA	GEO	GSM928592
SCL006-R	SCL006_icSARSCoV_12h_2_RNA_ExpSam	RNA	GEO	GSM928593
SCL006-R	SCL006_icSARSCoV_12h_3_B_RNA_ExpSam	RNA	GEO	GSM928594
SCL006-R	SCL006_icSARSCoV_24h_1_RNA_ExpSam	RNA	GEO	GSM928595
SCL006-R	SCL006_icSARSCoV_24h_2_RNA_ExpSam	RNA	GEO	GSM928596
SCL006-R	SCL006_icSARSCoV_24h_3_RNA_ExpSam	RNA	GEO	GSM928597
SCL006-R	SCL006_icSARSCoV_30h_1_RNA_ExpSam	RNA	GEO	GSM928598
SCL006-R	SCL006_icSARSCoV_30h_2_RNA_ExpSam	RNA	GEO	GSM928599
SCL006-R	SCL006_icSARSCoV_30h_3_RNA_ExpSam	RNA	GEO	GSM928600
SCL006-R	SCL006_icSARSCoV_36h_1_RNA_ExpSam	RNA	GEO	GSM928601
SCL006-R	SCL006_icSARSCoV_36h_2_RNA_ExpSam	RNA	GEO	GSM928602
SCL006-R	SCL006_icSARSCoV_36h_3_RNA_ExpSam	RNA	GEO	GSM928603
SCL006-R	SCL006_icSARSCoV_48h_1_RNA_ExpSam	RNA	GEO	GSM928604
SCL006-R	SCL006_icSARSCoV_48h_2_RNA_ExpSam	RNA	GEO	GSM928605
SCL006-R	SCL006_icSARSCoV_48h_3_RNA_ExpSam	RNA	GEO	GSM928606
SCL006-R	SCL006_icSARSCoV_54h_1_RNA_ExpSam	RNA	GEO	GSM928607
SCL006-R	SCL006_icSARSCoV_54h_2_RNA_ExpSam	RNA	GEO	GSM928608
SCL006-R	SCL006_icSARSCoV_54h_3_RNA_ExpSam	RNA	GEO	GSM928609
SCL006-R	SCL006_icSARSCoV_60h_1_RNA_ExpSam	RNA	GEO	GSM928610
SCL006-R	SCL006_icSARSCoV_60h_2_RNA_ExpSam	RNA	GEO	GSM928611
SCL006-R	SCL006_icSARSCoV_60h_3_B_RNA_ExpSam	RNA	GEO	GSM928612
SCL006-R	SCL006_icSARSCoV_72h_1_RNA_ExpSam	RNA	GEO	GSM928613
SCL006-R	SCL006_icSARSCoV_72h_2_RNA_ExpSam	RNA	GEO	GSM928614
SCL006-R	SCL006_icSARSCoV_72h_3_RNA_ExpSam	RNA	GEO	GSM928615
SCL006-R	SCL006_icSARSCoV_7h_1_RNA_ExpSam	RNA	GEO	GSM928589
SCL006-R	SCL006_icSARSCoV_7h_2_RNA_ExpSam	RNA	GEO	GSM928590
SCL006-R	SCL006_icSARSCoV_7h_3_RNA_ExpSam	RNA	GEO	GSM928591
SCL006-R	SCL006_mock_0h_1_RNA_ExpSam	RNA	GEO	GSM928529
SCL006-R	SCL006_mock_0h_2_RNA_ExpSam	RNA	GEO	GSM928530
SCL006-R	SCL006_mock_0h_3_RNA_ExpSam	RNA	GEO	GSM928531
SCL006-R	SCL006_mock_12h_1_RNA_ExpSam	RNA	GEO	GSM928535
SCL006-R	SCL006_mock_12h_2_RNA_ExpSam	RNA	GEO	GSM928536
SCL006-R	SCL006_mock_12h_3_RNA_ExpSam	RNA	GEO	GSM928537
SCL006-R	SCL006_mock_24h_1_RNA_ExpSam	RNA	GEO	GSM928538
SCL006-R	SCL006_mock_24h_2_RNA_ExpSam	RNA	GEO	GSM928539
SCL006-R	SCL006_mock_24h_3_RNA_ExpSam	RNA	GEO	GSM928540
SCL006-R	SCL006_mock_30h_1_RNA_ExpSam	RNA	GEO	GSM928541
SCL006-R	SCL006_mock_30h_2_RNA_ExpSam	RNA	GEO	GSM928542
SCL006-R	SCL006_mock_30h_3_RNA_ExpSam	RNA	GEO	GSM928543
SCL006-R	SCL006_mock_36h_1_RNA_ExpSam	RNA	GEO	GSM928544
SCL006-R	SCL006_mock_36h_2_RNA_ExpSam	RNA	GEO	GSM928545
SCL006-R	SCL006_mock_36h_3_RNA_ExpSam	RNA	GEO	GSM928546
SCL006-R	SCL006_mock_48h_1_RNA_ExpSam	RNA	GEO	GSM928547
SCL006-R	SCL006_mock_48h_2_RNA_ExpSam	RNA	GEO	GSM928548
SCL006-R	SCL006_mock_48h_3_RNA_ExpSam	RNA	GEO	GSM928549
SCL006-R	SCL006_mock_54h_1_RNA_ExpSam	RNA	GEO	GSM928550
SCL006-R	SCL006_mock_54h_2_RNA_ExpSam	RNA	GEO	GSM928551
SCL006-R	SCL006_mock_54h_3_RNA_ExpSam	RNA	GEO	GSM928552
SCL006-R	SCL006_mock_60h_1_RNA_ExpSam	RNA	GEO	GSM928553
SCL006-R	SCL006_mock_60h_2_RNA_ExpSam	RNA	GEO	GSM928554
SCL006-R	SCL006_mock_60h_3_RNA_ExpSam	RNA	GEO	GSM928555
SCL006-R	SCL006_mock_72h_1_RNA_ExpSam	RNA	GEO	GSM928556
SCL006-R	SCL006_mock_72h_2_RNA_ExpSam	RNA	GEO	GSM928557
SCL006-R	SCL006_mock_72h_3_RNA_ExpSam	RNA	GEO	GSM928558
SCL006-R	SCL006_mock_7h_1_RNA_ExpSam	RNA	GEO	GSM928532
SCL006-R	SCL006_mock_7h_2_RNA_ExpSam	RNA	GEO	GSM928533
SCL006-R	SCL006_mock_7h_3_RNA_ExpSam	RNA	GEO	GSM928534
SHAE002-R	SHAE002_BAT_0h_1_array	RNA	GEO	GSM1163233
SHAE002-R	SHAE002_BAT_0h_2_array	RNA	GEO	GSM1163234
SHAE002-R	SHAE002_BAT_0h_3_array	RNA	GEO	GSM1163235
SHAE002-R	SHAE002_BAT_0h_4_array	RNA	GEO	GSM1163236
SHAE002-R	SHAE002_BAT_12h_1_array	RNA	GEO	GSM1163237
SHAE002-R	SHAE002_BAT_12h_2_array	RNA	GEO	GSM1163238
SHAE002-R	SHAE002_BAT_12h_3_array	RNA	GEO	GSM1163239
SHAE002-R	SHAE002_BAT_12h_4_array	RNA	GEO	GSM1163240
SHAE002-R	SHAE002_BAT_24h_1_array	RNA	GEO	GSM1163241
SHAE002-R	SHAE002_BAT_24h_2_array	RNA	GEO	GSM1163242
SHAE002-R	SHAE002_BAT_24h_3_array	RNA	GEO	GSM1163243
SHAE002-R	SHAE002_BAT_24h_4_array	RNA	GEO	GSM1163244
SHAE002-R	SHAE002_BAT_36h_1_array	RNA	GEO	GSM1163245
SHAE002-R	SHAE002_BAT_36h_2_array	RNA	GEO	GSM1163246
SHAE002-R	SHAE002_BAT_36h_3_array	RNA	GEO	GSM1163247
SHAE002-R	SHAE002_BAT_36h_4_array	RNA	GEO	GSM1163248
SHAE002-R	SHAE002_BAT_48h_1_array	RNA	GEO	GSM1163249
SHAE002-R	SHAE002_BAT_48h_3_array	RNA	GEO	GSM1163250
SHAE002-R	SHAE002_BAT_48h_4_array	RNA	GEO	GSM1163251
SHAE002-R	SHAE002_BAT_60h_1_array	RNA	GEO	GSM1163252
SHAE002-R	SHAE002_BAT_60h_2_array	RNA	GEO	GSM1163390
SHAE002-R	SHAE002_BAT_60h_3_array	RNA	GEO	GSM1163253
SHAE002-R	SHAE002_BAT_60h_4_array	RNA	GEO	GSM1163254
SHAE002-R	SHAE002_BAT_72h_1_array	RNA	GEO	GSM1163255
SHAE002-R	SHAE002_BAT_72h_2_array	RNA	GEO	GSM1163256
SHAE002-R	SHAE002_BAT_72h_3_array	RNA	GEO	GSM1163257
SHAE002-R	SHAE002_BAT_72h_4_array	RNA	GEO	GSM1163258
SHAE002-R	SHAE002_BAT_84h_1_array	RNA	GEO	GSM1163392
SHAE002-R	SHAE002_BAT_84h_2_array	RNA	GEO	GSM1163259
SHAE002-R	SHAE002_BAT_84h_3_array	RNA	GEO	GSM1163260
SHAE002-R	SHAE002_BAT_84h_4_array	RNA	GEO	GSM1163261
SHAE002-R	SHAE002_BAT_96h_1_array	RNA	GEO	GSM1163262
SHAE002-R	SHAE002_BAT_96h_2_array	RNA	GEO	GSM1163263
SHAE002-R	SHAE002_BAT_96h_3_array	RNA	GEO	GSM1163264
SHAE002-R	SHAE002_BAT_96h_4_array	RNA	GEO	GSM1163265
SHAE002-R	SHAE002_dORF6_0h_1_array	RNA	GEO	GSM1163322
SHAE002-R	SHAE002_dORF6_0h_2_array	RNA	GEO	GSM1163323
SHAE002-R	SHAE002_dORF6_0h_3_array	RNA	GEO	GSM1163324
SHAE002-R	SHAE002_dORF6_0h_4_array	RNA	GEO	GSM1163325
SHAE002-R	SHAE002_dORF6_12h_1_array	RNA	GEO	GSM1163326
SHAE002-R	SHAE002_dORF6_12h_2_array	RNA	GEO	GSM1163327
SHAE002-R	SHAE002_dORF6_12h_3_array	RNA	GEO	GSM1163328
SHAE002-R	SHAE002_dORF6_12h_4_array	RNA	GEO	GSM1163329
SHAE002-R	SHAE002_dORF6_24h_1_array	RNA	GEO	GSM1163330
SHAE002-R	SHAE002_dORF6_24h_2_array	RNA	GEO	GSM1163331
SHAE002-R	SHAE002_dORF6_24h_4_array	RNA	GEO	GSM1163332
SHAE002-R	SHAE002_dORF6_36h_1_array	RNA	GEO	GSM1163333
SHAE002-R	SHAE002_dORF6_36h_2_array	RNA	GEO	GSM1163334
SHAE002-R	SHAE002_dORF6_36h_3_array	RNA	GEO	GSM1163335
SHAE002-R	SHAE002_dORF6_36h_4_array	RNA	GEO	GSM1163336
SHAE002-R	SHAE002_dORF6_48h_1_array	RNA	GEO	GSM1163337
SHAE002-R	SHAE002_dORF6_48h_2_array	RNA	GEO	GSM1163338
SHAE002-R	SHAE002_dORF6_48h_3_array	RNA	GEO	GSM1163339
SHAE002-R	SHAE002_dORF6_48h_4_array	RNA	GEO	GSM1163340
SHAE002-R	SHAE002_dORF6_60h_1_array	RNA	GEO	GSM1163341
SHAE002-R	SHAE002_dORF6_60h_2_array	RNA	GEO	GSM1163342
SHAE002-R	SHAE002_dORF6_60h_3_array	RNA	GEO	GSM1163343
SHAE002-R	SHAE002_dORF6_60h_4_array	RNA	GEO	GSM1163344
SHAE002-R	SHAE002_dORF6_72h_1_array	RNA	GEO	GSM1163345
SHAE002-R	SHAE002_dORF6_72h_2_array	RNA	GEO	GSM1163346
SHAE002-R	SHAE002_dORF6_72h_3_array	RNA	GEO	GSM1163347
SHAE002-R	SHAE002_dORF6_72h_4_array	RNA	GEO	GSM1163348
SHAE002-R	SHAE002_dORF6_84h_1_array	RNA	GEO	GSM1163349
SHAE002-R	SHAE002_dORF6_84h_2_array	RNA	GEO	GSM1163350
SHAE002-R	SHAE002_dORF6_84h_3_array	RNA	GEO	GSM1163351
SHAE002-R	SHAE002_dORF6_84h_4_array	RNA	GEO	GSM1163352
SHAE002-R	SHAE002_dORF6_96h_1_array	RNA	GEO	GSM1163353
SHAE002-R	SHAE002_dORF6_96h_2_array	RNA	GEO	GSM1163354
SHAE002-R	SHAE002_dORF6_96h_3_array	RNA	GEO	GSM1163355
SHAE002-R	SHAE002_dORF6_96h_4_array	RNA	GEO	GSM1163356
SHAE002-R	SHAE002_H1N1_0h_1_array	RNA	GEO	GSM1163266
SHAE002-R	SHAE002_H1N1_0h_2_array	RNA	GEO	GSM1163267
SHAE002-R	SHAE002_H1N1_0h_3_array	RNA	GEO	GSM1163268
SHAE002-R	SHAE002_H1N1_12h_1_array	RNA	GEO	GSM1163269
SHAE002-R	SHAE002_H1N1_12h_2_array	RNA	GEO	GSM1163270
SHAE002-R	SHAE002_H1N1_12h_3_array	RNA	GEO	GSM1163271
SHAE002-R	SHAE002_H1N1_18h_1_array	RNA	GEO	GSM1163272
SHAE002-R	SHAE002_H1N1_18h_2_array	RNA	GEO	GSM1163273
SHAE002-R	SHAE002_H1N1_18h_3_array	RNA	GEO	GSM1163395
SHAE002-R	SHAE002_H1N1_24h_1_array	RNA	GEO	GSM1163274
SHAE002-R	SHAE002_H1N1_24h_2_array	RNA	GEO	GSM1163275
SHAE002-R	SHAE002_H1N1_24h_3_array	RNA	GEO	GSM1163276
SHAE002-R	SHAE002_H1N1_36h_1_array	RNA	GEO	GSM1163277
SHAE002-R	SHAE002_H1N1_36h_2_array	RNA	GEO	GSM1163278
SHAE002-R	SHAE002_H1N1_36h_3_array	RNA	GEO	GSM1163394
SHAE002-R	SHAE002_H1N1_48h_1_array	RNA	GEO	GSM1163279
SHAE002-R	SHAE002_H1N1_48h_2_array	RNA	GEO	GSM1163280
SHAE002-R	SHAE002_H1N1_48h_3_array	RNA	GEO	GSM1163281
SHAE002-R	SHAE002_H1N1_48h_4_array	RNA	GEO	GSM1163282
SHAE002-R	SHAE002_H1N1_6h_1_array	RNA	GEO	GSM1163283
SHAE002-R	SHAE002_H1N1_6h_2_array	RNA	GEO	GSM1163284
SHAE002-R	SHAE002_H1N1_6h_3_array	RNA	GEO	GSM1163285
SHAE002-R	SHAE002_Mock_0h_1_array	RNA	GEO	GSM1163286
SHAE002-R	SHAE002_Mock_0h_2_array	RNA	GEO	GSM1163287
SHAE002-R	SHAE002_Mock_0h_3_array	RNA	GEO	GSM1163288
SHAE002-R	SHAE002_Mock_12h_1_array	RNA	GEO	GSM1163289
SHAE002-R	SHAE002_Mock_12h_2_array	RNA	GEO	GSM1163290
SHAE002-R	SHAE002_Mock_12h_3_array	RNA	GEO	GSM1163291
SHAE002-R	SHAE002_Mock_18h_1_array	RNA	GEO	GSM1163292
SHAE002-R	SHAE002_Mock_18h_2_array	RNA	GEO	GSM1163293
SHAE002-R	SHAE002_Mock_18h_3_array	RNA	GEO	GSM1163294
SHAE002-R	SHAE002_Mock_24h_1_array	RNA	GEO	GSM1163295
SHAE002-R	SHAE002_Mock_24h_2_array	RNA	GEO	GSM1163389
SHAE002-R	SHAE002_Mock_24h_3_array	RNA	GEO	GSM1163296
SHAE002-R	SHAE002_Mock_36h_1_array	RNA	GEO	GSM1163297
SHAE002-R	SHAE002_Mock_36h_2_array	RNA	GEO	GSM1163298
SHAE002-R	SHAE002_Mock_36h_3_array	RNA	GEO	GSM1163299
SHAE002-R	SHAE002_Mock_48h_1_array	RNA	GEO	GSM1163300
SHAE002-R	SHAE002_Mock_48h_3_array	RNA	GEO	GSM1163301
SHAE002-R	SHAE002_Mock_48h_4_array	RNA	GEO	GSM1163302
SHAE002-R	SHAE002_Mock_60h_1_array	RNA	GEO	GSM1163303
SHAE002-R	SHAE002_Mock_60h_2_array	RNA	GEO	GSM1163304
SHAE002-R	SHAE002_Mock_60h_3_array	RNA	GEO	GSM1163305
SHAE002-R	SHAE002_Mock_60h_4_array	RNA	GEO	GSM1163306
SHAE002-R	SHAE002_Mock_6h_1_array	RNA	GEO	GSM1163307
SHAE002-R	SHAE002_Mock_6h_2_array	RNA	GEO	GSM1163308
SHAE002-R	SHAE002_Mock_6h_3_array	RNA	GEO	GSM1163309
SHAE002-R	SHAE002_Mock_72h_1_array	RNA	GEO	GSM1163310
SHAE002-R	SHAE002_Mock_72h_2_array	RNA	GEO	GSM1163311
SHAE002-R	SHAE002_Mock_72h_3_array	RNA	GEO	GSM1163312
SHAE002-R	SHAE002_Mock_72h_4_array	RNA	GEO	GSM1163313
SHAE002-R	SHAE002_Mock_84h_1_array	RNA	GEO	GSM1163314
SHAE002-R	SHAE002_Mock_84h_2_array	RNA	GEO	GSM1163315
SHAE002-R	SHAE002_Mock_84h_3_array	RNA	GEO	GSM1163316
SHAE002-R	SHAE002_Mock_84h_4_array	RNA	GEO	GSM1163317
SHAE002-R	SHAE002_Mock_96h_1_array	RNA	GEO	GSM1163318
SHAE002-R	SHAE002_Mock_96h_2_array	RNA	GEO	GSM1163319
SHAE002-R	SHAE002_Mock_96h_3_array	RNA	GEO	GSM1163320
SHAE002-R	SHAE002_Mock_96h_4_array	RNA	GEO	GSM1163321
SHAE002-R	SHAE002_SARS_0h_1_array	RNA	GEO	GSM1163357
SHAE002-R	SHAE002_SARS_0h_2_array	RNA	GEO	GSM1163358
SHAE002-R	SHAE002_SARS_0h_3_array	RNA	GEO	GSM1163359
SHAE002-R	SHAE002_SARS_0h_4_array	RNA	GEO	GSM1163360
SHAE002-R	SHAE002_SARS_12h_1_array	RNA	GEO	GSM1163361
SHAE002-R	SHAE002_SARS_12h_2_array	RNA	GEO	GSM1163362
SHAE002-R	SHAE002_SARS_12h_3_array	RNA	GEO	GSM1163391
SHAE002-R	SHAE002_SARS_12h_4_array	RNA	GEO	GSM1163363
SHAE002-R	SHAE002_SARS_24h_1_array	RNA	GEO	GSM1163364
SHAE002-R	SHAE002_SARS_24h_2_array	RNA	GEO	GSM1163365
SHAE002-R	SHAE002_SARS_24h_3_array	RNA	GEO	GSM1163366
SHAE002-R	SHAE002_SARS_24h_4_array	RNA	GEO	GSM1163367
SHAE002-R	SHAE002_SARS_36h_1_array	RNA	GEO	GSM1163368
SHAE002-R	SHAE002_SARS_36h_2_array	RNA	GEO	GSM1163369
SHAE002-R	SHAE002_SARS_48h_1_array	RNA	GEO	GSM1163370
SHAE002-R	SHAE002_SARS_48h_2_array	RNA	GEO	GSM1163371
SHAE002-R	SHAE002_SARS_48h_3_array	RNA	GEO	GSM1163372
SHAE002-R	SHAE002_SARS_48h_4_array	RNA	GEO	GSM1163373
SHAE002-R	SHAE002_SARS_60h_1_array	RNA	GEO	GSM1163374
SHAE002-R	SHAE002_SARS_60h_2_array	RNA	GEO	GSM1163375
SHAE002-R	SHAE002_SARS_60h_3_array	RNA	GEO	GSM1163393
SHAE002-R	SHAE002_SARS_60h_4_array	RNA	GEO	GSM1163376
SHAE002-R	SHAE002_SARS_72h_1_array	RNA	GEO	GSM1163377
SHAE002-R	SHAE002_SARS_72h_2_array	RNA	GEO	GSM1163378
SHAE002-R	SHAE002_SARS_72h_3_array	RNA	GEO	GSM1163379
SHAE002-R	SHAE002_SARS_72h_4_array	RNA	GEO	GSM1163380
SHAE002-R	SHAE002_SARS_84h_1_array	RNA	GEO	GSM1163381
SHAE002-R	SHAE002_SARS_84h_2_array	RNA	GEO	GSM1163382
SHAE002-R	SHAE002_SARS_84h_3_array	RNA	GEO	GSM1163383
SHAE002-R	SHAE002_SARS_84h_4_array	RNA	GEO	GSM1163384
SHAE002-R	SHAE002_SARS_96h_1_array	RNA	GEO	GSM1163385
SHAE002-R	SHAE002_SARS_96h_2_array	RNA	GEO	GSM1163386
SHAE002-R	SHAE002_SARS_96h_3_array	RNA	GEO	GSM1163387
SHAE002-R	SHAE002_SARS_96h_4_array	RNA	GEO	GSM1163388
SHAE003-R	SHAE003_BAT_0h_1_array	RNA	GEO	GSM1163396
SHAE003-R	SHAE003_BAT_0h_2_array	RNA	GEO	GSM1163397
SHAE003-R	SHAE003_BAT_0h_3_array	RNA	GEO	GSM1163398
SHAE003-R	SHAE003_BAT_0h_4_array	RNA	GEO	GSM1163399
SHAE003-R	SHAE003_BAT_24h_3_array	RNA	GEO	GSM1163400
SHAE003-R	SHAE003_BAT_24h_4_array	RNA	GEO	GSM1163401
SHAE003-R	SHAE003_BAT_48h_1_array	RNA	GEO	GSM1163402
SHAE003-R	SHAE003_BAT_48h_2_array	RNA	GEO	GSM1163403
SHAE003-R	SHAE003_BAT_48h_3_array	RNA	GEO	GSM1163404
SHAE003-R	SHAE003_BAT_48h_4_array	RNA	GEO	GSM1163405
SHAE003-R	SHAE003_BAT_60h_1_array	RNA	GEO	GSM1163406
SHAE003-R	SHAE003_BAT_60h_2_array	RNA	GEO	GSM1163407
SHAE003-R	SHAE003_BAT_60h_3_array	RNA	GEO	GSM1163408
SHAE003-R	SHAE003_BAT_60h_4_array	RNA	GEO	GSM1163409
SHAE003-R	SHAE003_BAT_72h_1_array	RNA	GEO	GSM1163410
SHAE003-R	SHAE003_BAT_72h_2_array	RNA	GEO	GSM1163411
SHAE003-R	SHAE003_BAT_72h_3_array	RNA	GEO	GSM1163412
SHAE003-R	SHAE003_BAT_72h_4_array	RNA	GEO	GSM1163413
SHAE003-R	SHAE003_BAT_84h_1_array	RNA	GEO	GSM1163414
SHAE003-R	SHAE003_BAT_84h_2_array	RNA	GEO	GSM1163415
SHAE003-R	SHAE003_BAT_84h_3_array	RNA	GEO	GSM1163416
SHAE003-R	SHAE003_BAT_84h_4_array	RNA	GEO	GSM1163417
SHAE003-R	SHAE003_BAT_96h_1_array	RNA	GEO	GSM1163418
SHAE003-R	SHAE003_BAT_96h_2_array	RNA	GEO	GSM1163419
SHAE003-R	SHAE003_BAT_96h_3_array	RNA	GEO	GSM1163420
SHAE003-R	SHAE003_BAT_96h_4_array	RNA	GEO	GSM1163421
SHAE003-R	SHAE003_dORF6_0h_1_array	RNA	GEO	GSM1163510
SHAE003-R	SHAE003_dORF6_0h_2_array	RNA	GEO	GSM1163511
SHAE003-R	SHAE003_dORF6_0h_3_array	RNA	GEO	GSM1163512
SHAE003-R	SHAE003_dORF6_0h_4_array	RNA	GEO	GSM1163513
SHAE003-R	SHAE003_dORF6_24h_1_array	RNA	GEO	GSM1163514
SHAE003-R	SHAE003_dORF6_24h_2_array	RNA	GEO	GSM1163515
SHAE003-R	SHAE003_dORF6_24h_3_array	RNA	GEO	GSM1163516
SHAE003-R	SHAE003_dORF6_48h_1_array	RNA	GEO	GSM1163517
SHAE003-R	SHAE003_dORF6_48h_2_array	RNA	GEO	GSM1163518
SHAE003-R	SHAE003_dORF6_48h_3_array	RNA	GEO	GSM1163519
SHAE003-R	SHAE003_dORF6_48h_4_array	RNA	GEO	GSM1163520
SHAE003-R	SHAE003_dORF6_60h_1_array	RNA	GEO	GSM1163521
SHAE003-R	SHAE003_dORF6_60h_2_array	RNA	GEO	GSM1163522
SHAE003-R	SHAE003_dORF6_60h_3_array	RNA	GEO	GSM1163523
SHAE003-R	SHAE003_dORF6_60h_4_array	RNA	GEO	GSM1163524
SHAE003-R	SHAE003_dORF6_72h_1_array	RNA	GEO	GSM1163525
SHAE003-R	SHAE003_dORF6_72h_2_array	RNA	GEO	GSM1163526
SHAE003-R	SHAE003_dORF6_72h_3_array	RNA	GEO	GSM1163527
SHAE003-R	SHAE003_dORF6_72h_4_array	RNA	GEO	GSM1163528
SHAE003-R	SHAE003_dORF6_84h_1_array	RNA	GEO	GSM1163529
SHAE003-R	SHAE003_dORF6_84h_2_array	RNA	GEO	GSM1163530
SHAE003-R	SHAE003_dORF6_84h_3_array	RNA	GEO	GSM1163531
SHAE003-R	SHAE003_dORF6_84h_4_array	RNA	GEO	GSM1163532
SHAE003-R	SHAE003_dORF6_96h_1_array	RNA	GEO	GSM1163533
SHAE003-R	SHAE003_dORF6_96h_2_array	RNA	GEO	GSM1163534
SHAE003-R	SHAE003_dORF6_96h_3_array	RNA	GEO	GSM1163535
SHAE003-R	SHAE003_dORF6_96h_4_array	RNA	GEO	GSM1163536
SHAE003-R	SHAE003_H1N1_0h_1_array	RNA	GEO	GSM1163422
SHAE003-R	SHAE003_H1N1_0h_2_array	RNA	GEO	GSM1163423
SHAE003-R	SHAE003_H1N1_0h_3_array	RNA	GEO	GSM1163424
SHAE003-R	SHAE003_H1N1_12h_1_array	RNA	GEO	GSM1163430
SHAE003-R	SHAE003_H1N1_12h_2_array	RNA	GEO	GSM1163431
SHAE003-R	SHAE003_H1N1_12h_3_array	RNA	GEO	GSM1163432
SHAE003-R	SHAE003_H1N1_18h_1_array	RNA	GEO	GSM1163434
SHAE003-R	SHAE003_H1N1_18h_2_array	RNA	GEO	GSM1163435
SHAE003-R	SHAE003_H1N1_18h_3_array	RNA	GEO	GSM1163436
SHAE003-R	SHAE003_H1N1_24h_1_array	RNA	GEO	GSM1163438
SHAE003-R	SHAE003_H1N1_24h_2_array	RNA	GEO	GSM1163439
SHAE003-R	SHAE003_H1N1_24h_3_array	RNA	GEO	GSM1163440
SHAE003-R	SHAE003_H1N1_36h_1_array	RNA	GEO	GSM1163442
SHAE003-R	SHAE003_H1N1_36h_2_array	RNA	GEO	GSM1163443
SHAE003-R	SHAE003_H1N1_36h_3_array	RNA	GEO	GSM1163444
SHAE003-R	SHAE003_H1N1_48h_1_array	RNA	GEO	GSM1163446
SHAE003-R	SHAE003_H1N1_48h_2_array	RNA	GEO	GSM1163447
SHAE003-R	SHAE003_H1N1_48h_3_array	RNA	GEO	GSM1163448
SHAE003-R	SHAE003_H1N1_48h_4_array	RNA	GEO	GSM1163449
SHAE003-R	SHAE003_H1N1_6h_1_array	RNA	GEO	GSM1163426
SHAE003-R	SHAE003_H1N1_6h_2_array	RNA	GEO	GSM1163427
SHAE003-R	SHAE003_H1N1_6h_3_array	RNA	GEO	GSM1163428
SHAE003-R	SHAE003_Mock_0h_1_array	RNA	GEO	GSM1163478
SHAE003-R	SHAE003_Mock_0h_2_array	RNA	GEO	GSM1163479
SHAE003-R	SHAE003_Mock_0h_3_array	RNA	GEO	GSM1163480
SHAE003-R	SHAE003_Mock_12h_1_array	RNA	GEO	GSM1163484
SHAE003-R	SHAE003_Mock_12h_2_array	RNA	GEO	GSM1163485
SHAE003-R	SHAE003_Mock_12h_3_array	RNA	GEO	GSM1163486
SHAE003-R	SHAE003_Mock_18h_1_array	RNA	GEO	GSM1163487
SHAE003-R	SHAE003_Mock_18h_3_array	RNA	GEO	GSM1163488
SHAE003-R	SHAE003_Mock_24h_1_array	RNA	GEO	GSM1163489
SHAE003-R	SHAE003_Mock_24h_2_array	RNA	GEO	GSM1163490
SHAE003-R	SHAE003_Mock_24h_3_array	RNA	GEO	GSM1163491
SHAE003-R	SHAE003_Mock_36h_1_array	RNA	GEO	GSM1163492
SHAE003-R	SHAE003_Mock_36h_2_array	RNA	GEO	GSM1163493
SHAE003-R	SHAE003_Mock_36h_3_array	RNA	GEO	GSM1163494
SHAE003-R	SHAE003_Mock_48h_1_array	RNA	GEO	GSM1163495
SHAE003-R	SHAE003_Mock_48h_2_array	RNA	GEO	GSM1163496
SHAE003-R	SHAE003_Mock_48h_3_array	RNA	GEO	GSM1163497
SHAE003-R	SHAE003_Mock_60h_1_array	RNA	GEO	GSM1163498
SHAE003-R	SHAE003_Mock_60h_2_array	RNA	GEO	GSM1163499
SHAE003-R	SHAE003_Mock_60h_3_array	RNA	GEO	GSM1163500
SHAE003-R	SHAE003_Mock_6h_1_array	RNA	GEO	GSM1163481
SHAE003-R	SHAE003_Mock_6h_2_array	RNA	GEO	GSM1163482
SHAE003-R	SHAE003_Mock_6h_3_array	RNA	GEO	GSM1163483
SHAE003-R	SHAE003_Mock_72h_1_array	RNA	GEO	GSM1163501
SHAE003-R	SHAE003_Mock_72h_2_array	RNA	GEO	GSM1163502
SHAE003-R	SHAE003_Mock_72h_3_array	RNA	GEO	GSM1163503
SHAE003-R	SHAE003_Mock_84h_1_array	RNA	GEO	GSM1163504
SHAE003-R	SHAE003_Mock_84h_2_array	RNA	GEO	GSM1163505
SHAE003-R	SHAE003_Mock_84h_3_array	RNA	GEO	GSM1163506
SHAE003-R	SHAE003_Mock_96h_1_array	RNA	GEO	GSM1163507
SHAE003-R	SHAE003_Mock_96h_2_array	RNA	GEO	GSM1163508
SHAE003-R	SHAE003_Mock_96h_3_array	RNA	GEO	GSM1163509
SHAE003-R	SHAE003_SARS_0h_1_array	RNA	GEO	GSM1163450
SHAE003-R	SHAE003_SARS_0h_2_array	RNA	GEO	GSM1163451
SHAE003-R	SHAE003_SARS_0h_3_array	RNA	GEO	GSM1163452
SHAE003-R	SHAE003_SARS_0h_4_array	RNA	GEO	GSM1163453
SHAE003-R	SHAE003_SARS_24h_1_array	RNA	GEO	GSM1163454
SHAE003-R	SHAE003_SARS_24h_2_array	RNA	GEO	GSM1163455
SHAE003-R	SHAE003_SARS_24h_3_array	RNA	GEO	GSM1163456
SHAE003-R	SHAE003_SARS_24h_4_array	RNA	GEO	GSM1163457
SHAE003-R	SHAE003_SARS_48h_1_array	RNA	GEO	GSM1163458
SHAE003-R	SHAE003_SARS_48h_2_array	RNA	GEO	GSM1163459
SHAE003-R	SHAE003_SARS_48h_3_array	RNA	GEO	GSM1163460
SHAE003-R	SHAE003_SARS_60h_1_array	RNA	GEO	GSM1163462
SHAE003-R	SHAE003_SARS_60h_2_array	RNA	GEO	GSM1163463
SHAE003-R	SHAE003_SARS_60h_3_array	RNA	GEO	GSM1163464
SHAE003-R	SHAE003_SARS_60h_4_array	RNA	GEO	GSM1163465
SHAE003-R	SHAE003_SARS_72h_1_array	RNA	GEO	GSM1163466
SHAE003-R	SHAE003_SARS_72h_2_array	RNA	GEO	GSM1163467
SHAE003-R	SHAE003_SARS_72h_3_array	RNA	GEO	GSM1163468
SHAE003-R	SHAE003_SARS_72h_4_array	RNA	GEO	GSM1163469
SHAE003-R	SHAE003_SARS_84h_1_array	RNA	GEO	GSM1163470
SHAE003-R	SHAE003_SARS_84h_2_array	RNA	GEO	GSM1163471
SHAE003-R	SHAE003_SARS_84h_3_array	RNA	GEO	GSM1163472
SHAE003-R	SHAE003_SARS_84h_4_array	RNA	GEO	GSM1163473
SHAE003-R	SHAE003_SARS_96h_1_array	RNA	GEO	GSM1163474
SHAE003-R	SHAE003_SARS_96h_2_array	RNA	GEO	GSM1163475
SHAE003-R	SHAE003_SARS_96h_3_array	RNA	GEO	GSM1163476
SHAE003-R	SHAE003_SARS_96h_4_array	RNA	GEO	GSM1163477
SHAE004-R	SHAE004_BAT_0h_1_array	RNA	GEO	GSM1163537
SHAE004-R	SHAE004_BAT_0h_2_array	RNA	GEO	GSM1163538
SHAE004-R	SHAE004_BAT_0h_3_array	RNA	GEO	GSM1163539
SHAE004-R	SHAE004_BAT_12h_1_array	RNA	GEO	GSM1163540
SHAE004-R	SHAE004_BAT_12h_2_array	RNA	GEO	GSM1163541
SHAE004-R	SHAE004_BAT_12h_3_array	RNA	GEO	GSM1163542
SHAE004-R	SHAE004_BAT_24h_1_array	RNA	GEO	GSM1163543
SHAE004-R	SHAE004_BAT_24h_2_array	RNA	GEO	GSM1163544
SHAE004-R	SHAE004_BAT_36h_1_array	RNA	GEO	GSM1163545
SHAE004-R	SHAE004_BAT_36h_2_array	RNA	GEO	GSM1163546
SHAE004-R	SHAE004_BAT_36h_3_array	RNA	GEO	GSM1163547
SHAE004-R	SHAE004_BAT_48h_1_array	RNA	GEO	GSM1163548
SHAE004-R	SHAE004_BAT_48h_2_array	RNA	GEO	GSM1163549
SHAE004-R	SHAE004_BAT_48h_3_array	RNA	GEO	GSM1163550
SHAE004-R	SHAE004_BAT_60h_1_array	RNA	GEO	GSM1163551
SHAE004-R	SHAE004_BAT_60h_2_array	RNA	GEO	GSM1163552
SHAE004-R	SHAE004_BAT_60h_3_array	RNA	GEO	GSM1163553
SHAE004-R	SHAE004_BAT_72h_1_array	RNA	GEO	GSM1163554
SHAE004-R	SHAE004_BAT_72h_2_array	RNA	GEO	GSM1163555
SHAE004-R	SHAE004_BAT_72h_3_array	RNA	GEO	GSM1163556
SHAE004-R	SHAE004_BAT_84h_1_array	RNA	GEO	GSM1163557
SHAE004-R	SHAE004_BAT_84h_2_array	RNA	GEO	GSM1163558
SHAE004-R	SHAE004_BAT_84h_3_array	RNA	GEO	GSM1163559
SHAE004-R	SHAE004_BAT_96h_1_array	RNA	GEO	GSM1163560
SHAE004-R	SHAE004_BAT_96h_2_array	RNA	GEO	GSM1163561
SHAE004-R	SHAE004_BAT_96h_3_array	RNA	GEO	GSM1163562
SHAE004-R	SHAE004_dORF6_0h_1_array	RNA	GEO	GSM1163563
SHAE004-R	SHAE004_dORF6_0h_2_array	RNA	GEO	GSM1163564
SHAE004-R	SHAE004_dORF6_0h_3_array	RNA	GEO	GSM1163565
SHAE004-R	SHAE004_dORF6_12h_1_array	RNA	GEO	GSM1163566
SHAE004-R	SHAE004_dORF6_12h_2_array	RNA	GEO	GSM1163567
SHAE004-R	SHAE004_dORF6_12h_3_array	RNA	GEO	GSM1163568
SHAE004-R	SHAE004_dORF6_24h_1_array	RNA	GEO	GSM1163569
SHAE004-R	SHAE004_dORF6_24h_2_array	RNA	GEO	GSM1163570
SHAE004-R	SHAE004_dORF6_24h_3_array	RNA	GEO	GSM1163571
SHAE004-R	SHAE004_dORF6_36h_1_array	RNA	GEO	GSM1163572
SHAE004-R	SHAE004_dORF6_36h_2_array	RNA	GEO	GSM1163573
SHAE004-R	SHAE004_dORF6_36h_3_array	RNA	GEO	GSM1163574
SHAE004-R	SHAE004_dORF6_48h_1_array	RNA	GEO	GSM1163575
SHAE004-R	SHAE004_dORF6_48h_2_array	RNA	GEO	GSM1163576
SHAE004-R	SHAE004_dORF6_48h_3_array	RNA	GEO	GSM1163577
SHAE004-R	SHAE004_dORF6_60h_1_array	RNA	GEO	GSM1163578
SHAE004-R	SHAE004_dORF6_60h_2_array	RNA	GEO	GSM1163579
SHAE004-R	SHAE004_dORF6_60h_3_array	RNA	GEO	GSM1163580
SHAE004-R	SHAE004_dORF6_72h_1_array	RNA	GEO	GSM1163581
SHAE004-R	SHAE004_dORF6_72h_2_array	RNA	GEO	GSM1163582
SHAE004-R	SHAE004_dORF6_72h_3_array	RNA	GEO	GSM1163583
SHAE004-R	SHAE004_dORF6_84h_1_array	RNA	GEO	GSM1163584
SHAE004-R	SHAE004_dORF6_84h_2_array	RNA	GEO	GSM1163585
SHAE004-R	SHAE004_dORF6_84h_3_array	RNA	GEO	GSM1163586
SHAE004-R	SHAE004_dORF6_96h_1_array	RNA	GEO	GSM1163587
SHAE004-R	SHAE004_dORF6_96h_2_array	RNA	GEO	GSM1163588
SHAE004-R	SHAE004_dORF6_96h_3_array	RNA	GEO	GSM1163589
SHAE004-R	SHAE004_H1N1_0h_1_array	RNA	GEO	GSM1163590
SHAE004-R	SHAE004_H1N1_0h_2_array	RNA	GEO	GSM1163591
SHAE004-R	SHAE004_H1N1_0h_3_array	RNA	GEO	GSM1163592
SHAE004-R	SHAE004_H1N1_12h_1_array	RNA	GEO	GSM1163596
SHAE004-R	SHAE004_H1N1_12h_2_array	RNA	GEO	GSM1163597
SHAE004-R	SHAE004_H1N1_12h_3_array	RNA	GEO	GSM1163598
SHAE004-R	SHAE004_H1N1_18h_1_array	RNA	GEO	GSM1163599
SHAE004-R	SHAE004_H1N1_18h_2_array	RNA	GEO	GSM1163600
SHAE004-R	SHAE004_H1N1_18h_3_array	RNA	GEO	GSM1163601
SHAE004-R	SHAE004_H1N1_24h_1_array	RNA	GEO	GSM1163602
SHAE004-R	SHAE004_H1N1_24h_2_array	RNA	GEO	GSM1163603
SHAE004-R	SHAE004_H1N1_24h_3_array	RNA	GEO	GSM1163604
SHAE004-R	SHAE004_H1N1_36h_1_array	RNA	GEO	GSM1163605
SHAE004-R	SHAE004_H1N1_36h_2_array	RNA	GEO	GSM1163606
SHAE004-R	SHAE004_H1N1_36h_3_array	RNA	GEO	GSM1163607
SHAE004-R	SHAE004_H1N1_48h_1_array	RNA	GEO	GSM1163608
SHAE004-R	SHAE004_H1N1_48h_2_array	RNA	GEO	GSM1163609
SHAE004-R	SHAE004_H1N1_48h_3_array	RNA	GEO	GSM1163610
SHAE004-R	SHAE004_H1N1_6h_1_array	RNA	GEO	GSM1163593
SHAE004-R	SHAE004_H1N1_6h_2_array	RNA	GEO	GSM1163594
SHAE004-R	SHAE004_H1N1_6h_3_array	RNA	GEO	GSM1163595
SHAE004-R	SHAE004_Mock_0h_1_array	RNA	GEO	GSM1163638
SHAE004-R	SHAE004_Mock_0h_2_array	RNA	GEO	GSM1163639
SHAE004-R	SHAE004_Mock_0h_3_array	RNA	GEO	GSM1163640
SHAE004-R	SHAE004_Mock_12h_1_array	RNA	GEO	GSM1163644
SHAE004-R	SHAE004_Mock_12h_2_array	RNA	GEO	GSM1163645
SHAE004-R	SHAE004_Mock_12h_3_array	RNA	GEO	GSM1163646
SHAE004-R	SHAE004_Mock_18h_1_array	RNA	GEO	GSM1163647
SHAE004-R	SHAE004_Mock_18h_2_array	RNA	GEO	GSM1163648
SHAE004-R	SHAE004_Mock_18h_3_array	RNA	GEO	GSM1163649
SHAE004-R	SHAE004_Mock_24h_1_array	RNA	GEO	GSM1163650
SHAE004-R	SHAE004_Mock_24h_2_array	RNA	GEO	GSM1163651
SHAE004-R	SHAE004_Mock_24h_3_array	RNA	GEO	GSM1163652
SHAE004-R	SHAE004_Mock_36h_1_array	RNA	GEO	GSM1163653
SHAE004-R	SHAE004_Mock_36h_2_array	RNA	GEO	GSM1163654
SHAE004-R	SHAE004_Mock_36h_3_array	RNA	GEO	GSM1163655
SHAE004-R	SHAE004_Mock_48h_1_array	RNA	GEO	GSM1163656
SHAE004-R	SHAE004_Mock_48h_2_array	RNA	GEO	GSM1163657
SHAE004-R	SHAE004_Mock_48h_3_array	RNA	GEO	GSM1163658
SHAE004-R	SHAE004_Mock_60h_1_array	RNA	GEO	GSM1163659
SHAE004-R	SHAE004_Mock_60h_2_array	RNA	GEO	GSM1163660
SHAE004-R	SHAE004_Mock_60h_3_array	RNA	GEO	GSM1163661
SHAE004-R	SHAE004_Mock_6h_1_array	RNA	GEO	GSM1163641
SHAE004-R	SHAE004_Mock_6h_2_array	RNA	GEO	GSM1163642
SHAE004-R	SHAE004_Mock_6h_3_array	RNA	GEO	GSM1163643
SHAE004-R	SHAE004_Mock_72h_1_array	RNA	GEO	GSM1163662
SHAE004-R	SHAE004_Mock_72h_2_array	RNA	GEO	GSM1163663
SHAE004-R	SHAE004_Mock_72h_3_array	RNA	GEO	GSM1163664
SHAE004-R	SHAE004_Mock_84h_1_array	RNA	GEO	GSM1163665
SHAE004-R	SHAE004_Mock_84h_2_array	RNA	GEO	GSM1163666
SHAE004-R	SHAE004_Mock_84h_3_array	RNA	GEO	GSM1163667
SHAE004-R	SHAE004_Mock_96h_1_array	RNA	GEO	GSM1163668
SHAE004-R	SHAE004_Mock_96h_2_array	RNA	GEO	GSM1163669
SHAE004-R	SHAE004_Mock_96h_3_array	RNA	GEO	GSM1163670
SHAE004-R	SHAE004_SARS_0h_1_array	RNA	GEO	GSM1163611
SHAE004-R	SHAE004_SARS_0h_2_array	RNA	GEO	GSM1163612
SHAE004-R	SHAE004_SARS_0h_3_array	RNA	GEO	GSM1163613
SHAE004-R	SHAE004_SARS_12h_1_array	RNA	GEO	GSM1163614
SHAE004-R	SHAE004_SARS_12h_2_array	RNA	GEO	GSM1163615
SHAE004-R	SHAE004_SARS_12h_3_array	RNA	GEO	GSM1163616
SHAE004-R	SHAE004_SARS_24h_1_array	RNA	GEO	GSM1163617
SHAE004-R	SHAE004_SARS_24h_2_array	RNA	GEO	GSM1163618
SHAE004-R	SHAE004_SARS_24h_3_array	RNA	GEO	GSM1163619
SHAE004-R	SHAE004_SARS_36h_1_array	RNA	GEO	GSM1163620
SHAE004-R	SHAE004_SARS_36h_2_array	RNA	GEO	GSM1163621
SHAE004-R	SHAE004_SARS_36h_3_array	RNA	GEO	GSM1163622
SHAE004-R	SHAE004_SARS_48h_1_array	RNA	GEO	GSM1163623
SHAE004-R	SHAE004_SARS_48h_2_array	RNA	GEO	GSM1163624
SHAE004-R	SHAE004_SARS_48h_3_array	RNA	GEO	GSM1163625
SHAE004-R	SHAE004_SARS_60h_1_array	RNA	GEO	GSM1163626
SHAE004-R	SHAE004_SARS_60h_2_array	RNA	GEO	GSM1163627
SHAE004-R	SHAE004_SARS_60h_3_array	RNA	GEO	GSM1163628
SHAE004-R	SHAE004_SARS_72h_1_array	RNA	GEO	GSM1163629
SHAE004-R	SHAE004_SARS_72h_2_array	RNA	GEO	GSM1163630
SHAE004-R	SHAE004_SARS_72h_3_array	RNA	GEO	GSM1163631
SHAE004-R	SHAE004_SARS_84h_1_array	RNA	GEO	GSM1163632
SHAE004-R	SHAE004_SARS_84h_2_array	RNA	GEO	GSM1163633
SHAE004-R	SHAE004_SARS_84h_3_array	RNA	GEO	GSM1163634
SHAE004-R	SHAE004_SARS_96h_1_array	RNA	GEO	GSM1163635
SHAE004-R	SHAE004_SARS_96h_2_array	RNA	GEO	GSM1163636
SHAE004-R	SHAE004_SARS_96h_3_array	RNA	GEO	GSM1163637
SM001-R	SM001_Mock_D1_1_RNA_ExpSam	RNA	GEO	GSM823085
SM001-R	SM001_Mock_D1_2_RNA_ExpSam	RNA	GEO	GSM823086
SM001-R	SM001_Mock_D1_3_RNA_ExpSam	RNA	GEO	GSM823087
SM001-R	SM001_Mock_D2_1_RNA_ExpSam	RNA	GEO	GSM823088
SM001-R	SM001_Mock_D2_2_RNA_ExpSam	RNA	GEO	GSM823089
SM001-R	SM001_Mock_D2_3_RNA_ExpSam	RNA	GEO	GSM823090
SM001-R	SM001_Mock_D4_1_RNA_ExpSam	RNA	GEO	GSM823091
SM001-R	SM001_Mock_D4_2_RNA_ExpSam	RNA	GEO	GSM823092
SM001-R	SM001_Mock_D4_3_RNA_ExpSam	RNA	GEO	GSM823093
SM001-R	SM001_Mock_D7_1_RNA_ExpSam	RNA	GEO	GSM823094
SM001-R	SM001_Mock_D7_2_RNA_ExpSam	RNA	GEO	GSM823095
SM001-R	SM001_Mock_D7_3_RNA_ExpSam	RNA	GEO	GSM823096
SM001-R	SM001_SARS_10^2pfu_D1_1_RNA_ExpSam	RNA	GEO	GSM823097
SM001-R	SM001_SARS_10^2pfu_D1_2_RNA_ExpSam	RNA	GEO	GSM823098
SM001-R	SM001_SARS_10^2pfu_D1_3_RNA_ExpSam	RNA	GEO	GSM823099
SM001-R	SM001_SARS_10^2pfu_D1_4_RNA_ExpSam	RNA	GEO	GSM823100
SM001-R	SM001_SARS_10^2pfu_D1_5_RNA_ExpSam	RNA	GEO	GSM823101
SM001-R	SM001_SARS_10^2pfu_D2_1_RNA_ExpSam	RNA	GEO	GSM823102
SM001-R	SM001_SARS_10^2pfu_D2_2_RNA_ExpSam	RNA	GEO	GSM823103
SM001-R	SM001_SARS_10^2pfu_D2_3_RNA_ExpSam	RNA	GEO	GSM823104
SM001-R	SM001_SARS_10^2pfu_D2_4_RNA_ExpSam	RNA	GEO	GSM823105
SM001-R	SM001_SARS_10^2pfu_D2_5_RNA_ExpSam	RNA	GEO	GSM823106
SM001-R	SM001_SARS_10^2pfu_D4_1_RNA_ExpSam	RNA	GEO	GSM823107
SM001-R	SM001_SARS_10^2pfu_D4_2_RNA_ExpSam	RNA	GEO	GSM823108
SM001-R	SM001_SARS_10^2pfu_D4_3_RNA_ExpSam	RNA	GEO	GSM823109
SM001-R	SM001_SARS_10^2pfu_D4_4_RNA_ExpSam	RNA	GEO	GSM823110
SM001-R	SM001_SARS_10^2pfu_D4_5_RNA_ExpSam	RNA	GEO	GSM823111
SM001-R	SM001_SARS_10^2pfu_D7_1_RNA_ExpSam	RNA	GEO	GSM823112
SM001-R	SM001_SARS_10^2pfu_D7_2_RNA_ExpSam	RNA	GEO	GSM823113
SM001-R	SM001_SARS_10^2pfu_D7_3_RNA_ExpSam	RNA	GEO	GSM823114
SM001-R	SM001_SARS_10^2pfu_D7_4_RNA_ExpSam	RNA	GEO	GSM823115
SM001-R	SM001_SARS_10^2pfu_D7_5_RNA_ExpSam	RNA	GEO	GSM823116
SM001-R	SM001_SARS_10^3pfu_D1_1_RNA_ExpSam	RNA	GEO	GSM823117
SM001-R	SM001_SARS_10^3pfu_D1_2_RNA_ExpSam	RNA	GEO	GSM823118
SM001-R	SM001_SARS_10^3pfu_D1_3_RNA_ExpSam	RNA	GEO	GSM823119
SM001-R	SM001_SARS_10^3pfu_D1_4_RNA_ExpSam	RNA	GEO	GSM823120
SM001-R	SM001_SARS_10^3pfu_D1_5_RNA_ExpSam	RNA	GEO	GSM823121
SM001-R	SM001_SARS_10^3pfu_D2_1_RNA_ExpSam	RNA	GEO	GSM823122
SM001-R	SM001_SARS_10^3pfu_D2_2_RNA_ExpSam	RNA	GEO	GSM823123
SM001-R	SM001_SARS_10^3pfu_D2_3_RNA_ExpSam	RNA	GEO	GSM823124
SM001-R	SM001_SARS_10^3pfu_D2_4_RNA_ExpSam	RNA	GEO	GSM823125
SM001-R	SM001_SARS_10^3pfu_D2_5_RNA_ExpSam	RNA	GEO	GSM823126
SM001-R	SM001_SARS_10^3pfu_D4_1_RNA_ExpSam	RNA	GEO	GSM823127
SM001-R	SM001_SARS_10^3pfu_D4_2_RNA_ExpSam	RNA	GEO	GSM823128
SM001-R	SM001_SARS_10^3pfu_D4_3_RNA_ExpSam	RNA	GEO	GSM823129
SM001-R	SM001_SARS_10^3pfu_D4_4_RNA_ExpSam	RNA	GEO	GSM823130
SM001-R	SM001_SARS_10^3pfu_D4_5_RNA_ExpSam	RNA	GEO	GSM823131
SM001-R	SM001_SARS_10^3pfu_D7_1_RNA_ExpSam	RNA	GEO	GSM823132
SM001-R	SM001_SARS_10^3pfu_D7_2_RNA_ExpSam	RNA	GEO	GSM823133
SM001-R	SM001_SARS_10^3pfu_D7_3_RNA_ExpSam	RNA	GEO	GSM823134
SM001-R	SM001_SARS_10^3pfu_D7_4_RNA_ExpSam	RNA	GEO	GSM823135
SM001-R	SM001_SARS_10^3pfu_D7_5_RNA_ExpSam	RNA	GEO	GSM823136
SM001-R	SM001_SARS_10^4pfu_D1_1_RNA_ExpSam	RNA	GEO	GSM823137
SM001-R	SM001_SARS_10^4pfu_D1_2_RNA_ExpSam	RNA	GEO	GSM823138
SM001-R	SM001_SARS_10^4pfu_D1_3_RNA_ExpSam	RNA	GEO	GSM823139
SM001-R	SM001_SARS_10^4pfu_D1_4_RNA_ExpSam	RNA	GEO	GSM823140
SM001-R	SM001_SARS_10^4pfu_D1_5_RNA_ExpSam	RNA	GEO	GSM823141
SM001-R	SM001_SARS_10^4pfu_D2_1_RNA_ExpSam	RNA	GEO	GSM823142
SM001-R	SM001_SARS_10^4pfu_D2_2_RNA_ExpSam	RNA	GEO	GSM823143
SM001-R	SM001_SARS_10^4pfu_D2_3_RNA_ExpSam	RNA	GEO	GSM823144
SM001-R	SM001_SARS_10^4pfu_D2_4_RNA_ExpSam	RNA	GEO	GSM823145
SM001-R	SM001_SARS_10^4pfu_D2_5_RNA_ExpSam	RNA	GEO	GSM823146
SM001-R	SM001_SARS_10^4pfu_D4_1_RNA_ExpSam	RNA	GEO	GSM823147
SM001-R	SM001_SARS_10^4pfu_D4_2_RNA_ExpSam	RNA	GEO	GSM823148
SM001-R	SM001_SARS_10^4pfu_D4_3_RNA_ExpSam	RNA	GEO	GSM823149
SM001-R	SM001_SARS_10^4pfu_D4_4_RNA_ExpSam	RNA	GEO	GSM823150
SM001-R	SM001_SARS_10^4pfu_D4_5_RNA_ExpSam	RNA	GEO	GSM823151
SM001-R	SM001_SARS_10^4pfu_D7_1_RNA_ExpSam	RNA	GEO	GSM823152
SM001-R	SM001_SARS_10^4pfu_D7_2_RNA_ExpSam	RNA	GEO	GSM823153
SM001-R	SM001_SARS_10^4pfu_D7_3_RNA_ExpSam	RNA	GEO	GSM823154
SM001-R	SM001_SARS_10^4pfu_D7_4_RNA_ExpSam	RNA	GEO	GSM823155
SM001-R	SM001_SARS_10^4pfu_D7_5_RNA_ExpSam	RNA	GEO	GSM823156
SM001-R	SM001_SARS_10^5pfu_D1_1_RNA_ExpSam	RNA	GEO	GSM823157
SM001-R	SM001_SARS_10^5pfu_D1_2_RNA_ExpSam	RNA	GEO	GSM823158
SM001-R	SM001_SARS_10^5pfu_D1_3_RNA_ExpSam	RNA	GEO	GSM823159
SM001-R	SM001_SARS_10^5pfu_D1_4_RNA_ExpSam	RNA	GEO	GSM823160
SM001-R	SM001_SARS_10^5pfu_D1_5_RNA_ExpSam	RNA	GEO	GSM823161
SM001-R	SM001_SARS_10^5pfu_D2_1_RNA_ExpSam	RNA	GEO	GSM823162
SM001-R	SM001_SARS_10^5pfu_D2_2_RNA_ExpSam	RNA	GEO	GSM823163
SM001-R	SM001_SARS_10^5pfu_D2_3_RNA_ExpSam	RNA	GEO	GSM823164
SM001-R	SM001_SARS_10^5pfu_D2_4_RNA_ExpSam	RNA	GEO	GSM823165
SM001-R	SM001_SARS_10^5pfu_D2_5_RNA_ExpSam	RNA	GEO	GSM823166
SM001-R	SM001_SARS_10^5pfu_D4_1_RNA_ExpSam	RNA	GEO	GSM823167
SM001-R	SM001_SARS_10^5pfu_D4_2_RNA_ExpSam	RNA	GEO	GSM823168
SM001-R	SM001_SARS_10^5pfu_D4_3_RNA_ExpSam	RNA	GEO	GSM823169
SM001-R	SM001_SARS_10^5pfu_D4_4_RNA_ExpSam	RNA	GEO	GSM823170
SM001-R	SM001_SARS_10^5pfu_D4_5_RNA_ExpSam	RNA	GEO	GSM823171
SM001-R	SM001_SARS_10^5pfu_D7_1_RNA_ExpSam	RNA	GEO	GSM823172
SM001-R	SM001_SARS_10^5pfu_D7_2_RNA_ExpSam	RNA	GEO	GSM823173
SM001-R	SM001_SARS_10^5pfu_D7_3_RNA_ExpSam	RNA	GEO	GSM823174
SM001-R	SM001_SARS_10^5pfu_D7_4_RNA_ExpSam	RNA	GEO	GSM823175
SM001-R	SM001_SARS_10^5pfu_D7_5_RNA_ExpSam	RNA	GEO	GSM823176
SM003-R	SM003_10^4_MA15_d1_1_array	RNA	GEO	GSM1211755
SM003-R	SM003_10^4_MA15_d1_2_array	RNA	GEO	GSM1211756
SM003-R	SM003_10^4_MA15_d1_3_array	RNA	GEO	GSM1211757
SM003-R	SM003_10^4_MA15_d1_4_array	RNA	GEO	GSM1211758
SM003-R	SM003_10^4_MA15_d2_1_array	RNA	GEO	GSM1211759
SM003-R	SM003_10^4_MA15_d2_2_array	RNA	GEO	GSM1211760
SM003-R	SM003_10^4_MA15_d2_3_array	RNA	GEO	GSM1211761
SM003-R	SM003_10^4_MA15_d2_4_array	RNA	GEO	GSM1211762
SM003-R	SM003_10^4_MA15_d4_1_array	RNA	GEO	GSM1211763
SM003-R	SM003_10^4_MA15_d4_2_array	RNA	GEO	GSM1211764
SM003-R	SM003_10^4_MA15_d4_3_array	RNA	GEO	GSM1211765
SM003-R	SM003_10^4_MA15_d4_4_array	RNA	GEO	GSM1211766
SM003-R	SM003_10^4_MA15_d7_1_array	RNA	GEO	GSM1211767
SM003-R	SM003_10^4_MA15_d7_2_array	RNA	GEO	GSM1211768
SM003-R	SM003_10^4_MA15_d7_3_array	RNA	GEO	GSM1211769
SM003-R	SM003_10^5_MA15_d1_1_array	RNA	GEO	GSM1211771
SM003-R	SM003_10^5_MA15_d1_3_array	RNA	GEO	GSM1211773
SM003-R	SM003_10^5_MA15_d1_4_array	RNA	GEO	GSM1211774
SM003-R	SM003_10^5_MA15_d2_2_array	RNA	GEO	GSM1211776
SM003-R	SM003_10^5_MA15_d2_3_array	RNA	GEO	GSM1211777
SM003-R	SM003_10^5_MA15_d2_4_array	RNA	GEO	GSM1211778
SM003-R	SM003_10^5_MA15_d4_1_array	RNA	GEO	GSM1211779
SM003-R	SM003_10^5_MA15_d4_2_array	RNA	GEO	GSM1211780
SM003-R	SM003_10^5_MA15_d4_3_array	RNA	GEO	GSM1211781
SM003-R	SM003_10^5_MA15_d4_4_array	RNA	GEO	GSM1211782
SM003-R	SM003_10^5_MA15_d4_5_array	RNA	GEO	GSM1211783
SM003-R	SM003_10^5_MA15_d7_1_array	RNA	GEO	GSM1211784
SM003-R	SM003_10^5_MA15_d7_2_array	RNA	GEO	GSM1211785
SM003-R	SM003_10^5_MA15_d7_3_array	RNA	GEO	GSM1211786
SM003-R	SM003_BAT_d1_1_array	RNA	GEO	GSM1211787
SM003-R	SM003_BAT_d1_2_array	RNA	GEO	GSM1211788
SM003-R	SM003_BAT_d1_3_array	RNA	GEO	GSM1211789
SM003-R	SM003_BAT_d1_4_array	RNA	GEO	GSM1211790
SM003-R	SM003_BAT_d1_5_array	RNA	GEO	GSM1211791
SM003-R	SM003_BAT_d2_1_array	RNA	GEO	GSM1211792
SM003-R	SM003_BAT_d2_2_array	RNA	GEO	GSM1211793
SM003-R	SM003_BAT_d2_3_array	RNA	GEO	GSM1211794
SM003-R	SM003_BAT_d2_4_array	RNA	GEO	GSM1211795
SM003-R	SM003_BAT_d2_5_array	RNA	GEO	GSM1211796
SM003-R	SM003_BAT_d4_1_array	RNA	GEO	GSM1211797
SM003-R	SM003_BAT_d4_2_array	RNA	GEO	GSM1211798
SM003-R	SM003_BAT_d4_3_array	RNA	GEO	GSM1211799
SM003-R	SM003_BAT_d4_4_array	RNA	GEO	GSM1211800
SM003-R	SM003_BAT_d4_5_array	RNA	GEO	GSM1211801
SM003-R	SM003_BAT_d7_1_array	RNA	GEO	GSM1211802
SM003-R	SM003_BAT_d7_3_array	RNA	GEO	GSM1211803
SM003-R	SM003_BAT_d7_4_array	RNA	GEO	GSM1211804
SM003-R	SM003_BAT_d7_5_array	RNA	GEO	GSM1211805
SM003-R	SM003_Mock_d1_1_array	RNA	GEO	GSM1211825
SM003-R	SM003_Mock_d1_2_array	RNA	GEO	GSM1211826
SM003-R	SM003_Mock_d1_3_array	RNA	GEO	GSM1211827
SM003-R	SM003_Mock_d1_4_array	RNA	GEO	GSM1211828
SM003-R	SM003_Mock_d2_1_array	RNA	GEO	GSM1211829
SM003-R	SM003_Mock_d2_2_array	RNA	GEO	GSM1211830
SM003-R	SM003_Mock_d2_3_array	RNA	GEO	GSM1211831
SM003-R	SM003_Mock_d2_4_array	RNA	GEO	GSM1211832
SM003-R	SM003_Mock_d4_1_array	RNA	GEO	GSM1211833
SM003-R	SM003_Mock_d4_2_array	RNA	GEO	GSM1211834
SM003-R	SM003_Mock_d4_3_array	RNA	GEO	GSM1211835
SM003-R	SM003_Mock_d4_4_array	RNA	GEO	GSM1211836
SM003-R	SM003_Mock_d7_1_array	RNA	GEO	GSM1211837
SM003-R	SM003_Mock_d7_2_array	RNA	GEO	GSM1211838
SM003-R	SM003_Mock_d7_3_array	RNA	GEO	GSM1211839
SM003-R	SM003_Mock_d7_4_array	RNA	GEO	GSM1211840
SM003-R	SM003_SARS_d1_1_array	RNA	GEO	GSM1211806
SM003-R	SM003_SARS_d1_2_array	RNA	GEO	GSM1211807
SM003-R	SM003_SARS_d1_3_array	RNA	GEO	GSM1211808
SM003-R	SM003_SARS_d1_4_array	RNA	GEO	GSM1211809
SM003-R	SM003_SARS_d2_1_array	RNA	GEO	GSM1211810
SM003-R	SM003_SARS_d2_2_array	RNA	GEO	GSM1211811
SM003-R	SM003_SARS_d2_3_array	RNA	GEO	GSM1211812
SM003-R	SM003_SARS_d2_4_array	RNA	GEO	GSM1211813
SM003-R	SM003_SARS_d2_5_array	RNA	GEO	GSM1211814
SM003-R	SM003_SARS_d4_1_array	RNA	GEO	GSM1211815
SM003-R	SM003_SARS_d4_2_array	RNA	GEO	GSM1211816
SM003-R	SM003_SARS_d4_3_array	RNA	GEO	GSM1211817
SM003-R	SM003_SARS_d4_4_array	RNA	GEO	GSM1211818
SM003-R	SM003_SARS_d4_5_array	RNA	GEO	GSM1211819
SM003-R	SM003_SARS_d7_1_array	RNA	GEO	GSM1211820
SM003-R	SM003_SARS_d7_2_array	RNA	GEO	GSM1211821
SM003-R	SM003_SARS_d7_3_array	RNA	GEO	GSM1211822
SM003-R	SM003_SARS_d7_4_array	RNA	GEO	GSM1211823
SM003-R	SM003_SARS_d7_5_array	RNA	GEO	GSM1211824
SM004-R	SM004_B6_MA15_d4_1_array	RNA	GEO	GSM1244476
SM004-R	SM004_B6_MA15_d4_2_array	RNA	GEO	GSM1244477
SM004-R	SM004_B6_MA15_d4_3_array	RNA	GEO	GSM1244478
SM004-R	SM004_B6_MA15_d4_4_array	RNA	GEO	GSM1244479
SM004-R	SM004_B6_MA15_d7_1_array	RNA	GEO	GSM1244480
SM004-R	SM004_B6_MA15_d7_3_array	RNA	GEO	GSM1244448
SM004-R	SM004_B6_MA15_d7_4_array	RNA	GEO	GSM1244449
SM004-R	SM004_B6_Mock_d4_1_array	RNA	GEO	GSM1244450
SM004-R	SM004_B6_Mock_d4_2_array	RNA	GEO	GSM1244451
SM004-R	SM004_B6_Mock_d4_3_array	RNA	GEO	GSM1244452
SM004-R	SM004_B6_Mock_d4_4_array	RNA	GEO	GSM1244453
SM004-R	SM004_B6_Mock_d7_1_array	RNA	GEO	GSM1244454
SM004-R	SM004_B6_Mock_d7_2_array	RNA	GEO	GSM1244455
SM004-R	SM004_B6_Mock_d7_3_array	RNA	GEO	GSM1244456
SM004-R	SM004_B6_Mock_d7_4_array	RNA	GEO	GSM1244457
SM004-R	SM004_PAI1_MA15_d4_1_array	RNA	GEO	GSM1244465
SM004-R	SM004_PAI1_MA15_d4_2_array	RNA	GEO	GSM1244466
SM004-R	SM004_PAI1_MA15_d4_3_array	RNA	GEO	GSM1244467
SM004-R	SM004_PAI1_MA15_d4_4_array	RNA	GEO	GSM1244468
SM004-R	SM004_PAI1_MA15_d7_1_array	RNA	GEO	GSM1244469
SM004-R	SM004_PAI1_MA15_d7_2_array	RNA	GEO	GSM1244470
SM004-R	SM004_PAI1_MA15_d7_3_array	RNA	GEO	GSM1244471
SM004-R	SM004_PAI1_Mock_d4_1_array	RNA	GEO	GSM1244472
SM004-R	SM004_PAI1_Mock_d4_2_array	RNA	GEO	GSM1244473
SM004-R	SM004_PAI1_Mock_d7_1_array	RNA	GEO	GSM1244474
SM004-R	SM004_PAI1_Mock_d7_2_array	RNA	GEO	GSM1244475
SM004-R	SM004_TIMP1_MA15_d4_1_array	RNA	GEO	GSM1244443
SM004-R	SM004_TIMP1_MA15_d4_2_array	RNA	GEO	GSM1244444
SM004-R	SM004_TIMP1_MA15_d4_3_array	RNA	GEO	GSM1244445
SM004-R	SM004_TIMP1_MA15_d4_4_array	RNA	GEO	GSM1244446
SM004-R	SM004_TIMP1_MA15_d7_1_array	RNA	GEO	GSM1244447
SM004-R	SM004_TIMP1_MA15_d7_2_array	RNA	GEO	GSM1244458
SM004-R	SM004_TIMP1_MA15_d7_3_array	RNA	GEO	GSM1244459
SM004-R	SM004_TIMP1_MA15_d7_4_array	RNA	GEO	GSM1244460
SM004-R	SM004_TIMP1_Mock_d4_1_array	RNA	GEO	GSM1244461
SM004-R	SM004_TIMP1_Mock_d4_2_array	RNA	GEO	GSM1244462
SM004-R	SM004_TIMP1_Mock_d7_1_array	RNA	GEO	GSM1244463
SM004-R	SM004_TIMP1_Mock_d7_2_array	RNA	GEO	GSM1244464
SM007-R	SM007_B6_MA15_d2_1_array	RNA	GEO	GSM1231651
SM007-R	SM007_B6_MA15_d2_2_array	RNA	GEO	GSM1231652
SM007-R	SM007_B6_MA15_d2_3_array	RNA	GEO	GSM1231653
SM007-R	SM007_B6_MA15_d4_1_array	RNA	GEO	GSM1231654
SM007-R	SM007_B6_MA15_d4_2_array	RNA	GEO	GSM1231655
SM007-R	SM007_B6_MA15_d7_1_array	RNA	GEO	GSM1231656
SM007-R	SM007_B6_MA15_d7_2_array	RNA	GEO	GSM1231657
SM007-R	SM007_B6_MA15_d7_3_array	RNA	GEO	GSM1231658
SM007-R	SM007_B6_Mock_d2_1_array	RNA	GEO	GSM1231659
SM007-R	SM007_B6_Mock_d2_2_array	RNA	GEO	GSM1231660
SM007-R	SM007_B6_Mock_d2_3_array	RNA	GEO	GSM1231661
SM007-R	SM007_B6_Mock_d4_1_array	RNA	GEO	GSM1231662
SM007-R	SM007_B6_Mock_d4_2_array	RNA	GEO	GSM1231663
SM007-R	SM007_B6_Mock_d4_3_array	RNA	GEO	GSM1231664
SM007-R	SM007_B6_Mock_d7_1_array	RNA	GEO	GSM1231665
SM007-R	SM007_B6_Mock_d7_2_array	RNA	GEO	GSM1231666
SM007-R	SM007_B6_Mock_d7_3_array	RNA	GEO	GSM1231667
SM007-R	SM007_CXCR3_MA15_d2_1_array	RNA	GEO	GSM1231668
SM007-R	SM007_CXCR3_MA15_d2_2_array	RNA	GEO	GSM1231669
SM007-R	SM007_CXCR3_MA15_d2_3_array	RNA	GEO	GSM1231670
SM007-R	SM007_CXCR3_MA15_d4_2_array	RNA	GEO	GSM1231671
SM007-R	SM007_CXCR3_MA15_d4_3_array	RNA	GEO	GSM1231672
SM007-R	SM007_CXCR3_MA15_d7_1_array	RNA	GEO	GSM1231673
SM007-R	SM007_CXCR3_MA15_d7_2_array	RNA	GEO	GSM1231674
SM007-R	SM007_CXCR3_MA15_d7_3_array	RNA	GEO	GSM1231675
SM007-R	SM007_CXCR3_MA15_d7_4_array	RNA	GEO	GSM1231676
SM007-R	SM007_CXCR3_Mock_d2_2_array	RNA	GEO	GSM1231677
SM007-R	SM007_CXCR3_Mock_d2_3_array	RNA	GEO	GSM1231678
SM007-R	SM007_CXCR3_Mock_d4_1_array	RNA	GEO	GSM1231679
SM007-R	SM007_CXCR3_Mock_d4_2_array	RNA	GEO	GSM1231680
SM007-R	SM007_CXCR3_Mock_d7_1_array	RNA	GEO	GSM1231681
SM007-R	SM007_CXCR3_Mock_d7_2_array	RNA	GEO	GSM1231682
SM007-R	SM007_CXCR3_Mock_d7_3_array	RNA	GEO	GSM1231683
SM009-R	SM009_B6_MA15_d4_2_array	RNA	GEO	GSM1244490
SM009-R	SM009_B6_MA15_d4_3_array	RNA	GEO	GSM1244491
SM009-R	SM009_B6_MA15_d4_4_array	RNA	GEO	GSM1244492
SM009-R	SM009_B6_MA15_d7_2_array	RNA	GEO	GSM1244493
SM009-R	SM009_B6_MA15_d7_4_array	RNA	GEO	GSM1244494
SM009-R	SM009_B6_Mock_d4_1_array	RNA	GEO	GSM1244495
SM009-R	SM009_B6_Mock_d4_2_array	RNA	GEO	GSM1244496
SM009-R	SM009_B6_Mock_d7_1_array	RNA	GEO	GSM1244497
SM009-R	SM009_B6_Mock_d7_2_array	RNA	GEO	GSM1244498
SM009-R	SM009_PLAT_MA15_d4_3_array	RNA	GEO	GSM1244481
SM009-R	SM009_PLAT_MA15_d4_4_array	RNA	GEO	GSM1244482
SM009-R	SM009_PLAT_MA15_d7_1_array	RNA	GEO	GSM1244483
SM009-R	SM009_PLAT_MA15_d7_2_array	RNA	GEO	GSM1244484
SM009-R	SM009_PLAT_MA15_d7_4_array	RNA	GEO	GSM1244485
SM009-R	SM009_PLAT_Mock_d4_1_array	RNA	GEO	GSM1244486
SM009-R	SM009_PLAT_Mock_d4_2_array	RNA	GEO	GSM1244487
SM009-R	SM009_PLAT_Mock_d7_1_array	RNA	GEO	GSM1244488
SM009-R	SM009_PLAT_Mock_d7_2_array	RNA	GEO	GSM1244489
SM012-R	SM012_dORF6_10^5pfu_1d_1_array	RNA	GEO	GSM1196066
SM012-R	SM012_dORF6_10^5pfu_1d_3_array	RNA	GEO	GSM1196067
SM012-R	SM012_dORF6_10^5pfu_1d_5_array	RNA	GEO	GSM1196068
SM012-R	SM012_dORF6_10^5pfu_2d_1_array	RNA	GEO	GSM1196069
SM012-R	SM012_dORF6_10^5pfu_2d_3_array	RNA	GEO	GSM1196070
SM012-R	SM012_dORF6_10^5pfu_2d_4_array	RNA	GEO	GSM1196071
SM012-R	SM012_dORF6_10^5pfu_4d_1_array	RNA	GEO	GSM1196072
SM012-R	SM012_dORF6_10^5pfu_4d_2_array	RNA	GEO	GSM1196073
SM012-R	SM012_dORF6_10^5pfu_4d_3_array	RNA	GEO	GSM1196074
SM012-R	SM012_dORF6_10^5pfu_7d_1_array	RNA	GEO	GSM1196075
SM012-R	SM012_dORF6_10^5pfu_7d_2_array	RNA	GEO	GSM1196076
SM012-R	SM012_dORF6_10^5pfu_7d_3_array	RNA	GEO	GSM1196077
SM012-R	SM012_MA15_10^5pfu_1d_2_array	RNA	GEO	GSM1196078
SM012-R	SM012_MA15_10^5pfu_1d_3_array	RNA	GEO	GSM1196079
SM012-R	SM012_MA15_10^5pfu_1d_4_array	RNA	GEO	GSM1196080
SM012-R	SM012_MA15_10^5pfu_2d_1_array	RNA	GEO	GSM1196081
SM012-R	SM012_MA15_10^5pfu_2d_3_array	RNA	GEO	GSM1196082
SM012-R	SM012_MA15_10^5pfu_2d_5_array	RNA	GEO	GSM1196083
SM012-R	SM012_MA15_10^5pfu_4d_1_array	RNA	GEO	GSM1196084
SM012-R	SM012_MA15_10^5pfu_4d_2_array	RNA	GEO	GSM1196085
SM012-R	SM012_MA15_10^5pfu_4d_3_array	RNA	GEO	GSM1196086
SM012-R	SM012_MA15_10^5pfu_7d_1_array	RNA	GEO	GSM1196087
SM012-R	SM012_MA15_10^5pfu_7d_2_array	RNA	GEO	GSM1196088
SM012-R	SM012_MA15_10^5pfu_7d_3_array	RNA	GEO	GSM1196089
SM012-R	SM012_Mock_1d_1_array	RNA	GEO	GSM1196090
SM012-R	SM012_Mock_1d_4_array	RNA	GEO	GSM1196091
SM012-R	SM012_Mock_1d_5_array	RNA	GEO	GSM1196092
SM012-R	SM012_Mock_2d_2_array	RNA	GEO	GSM1196093
SM012-R	SM012_Mock_2d_3_array	RNA	GEO	GSM1196094
SM012-R	SM012_Mock_2d_4_array	RNA	GEO	GSM1196095
SM012-R	SM012_Mock_4d_1_array	RNA	GEO	GSM1196096
SM012-R	SM012_Mock_4d_2_array	RNA	GEO	GSM1196097
SM012-R	SM012_Mock_4d_3_array	RNA	GEO	GSM1196098
SM012-R	SM012_Mock_7d_1_array	RNA	GEO	GSM1196099
SM012-R	SM012_Mock_7d_3_array	RNA	GEO	GSM1196100
SM014-R	SM014_MA15_10^5pfu_1d_1_array	RNA	GEO	GSM1196101
SM014-R	SM014_MA15_10^5pfu_1d_2_array	RNA	GEO	GSM1196102
SM014-R	SM014_MA15_10^5pfu_1d_3_array	RNA	GEO	GSM1196103
SM014-R	SM014_MA15_10^5pfu_1d_4_array	RNA	GEO	GSM1196104
SM014-R	SM014_MA15_10^5pfu_2d_1_array	RNA	GEO	GSM1196105
SM014-R	SM014_MA15_10^5pfu_2d_2_array	RNA	GEO	GSM1196106
SM014-R	SM014_MA15_10^5pfu_2d_3_array	RNA	GEO	GSM1196107
SM014-R	SM014_MA15_10^5pfu_2d_4_array	RNA	GEO	GSM1196108
SM014-R	SM014_MA15_10^5pfu_4d_1_array	RNA	GEO	GSM1196109
SM014-R	SM014_MA15_10^5pfu_4d_2_array	RNA	GEO	GSM1196110
SM014-R	SM014_MA15_10^5pfu_4d_3_array	RNA	GEO	GSM1196111
SM014-R	SM014_MA15_10^5pfu_4d_4_array	RNA	GEO	GSM1196112
SM014-R	SM014_MA15_10^5pfu_7d_1_array	RNA	GEO	GSM1196113
SM014-R	SM014_MA15_10^5pfu_7d_2_array	RNA	GEO	GSM1196114
SM014-R	SM014_MA15_10^5pfu_7d_4_array	RNA	GEO	GSM1196115
SM014-R	SM014_Mock_1d_2_array	RNA	GEO	GSM1196116
SM014-R	SM014_Mock_1d_3_array	RNA	GEO	GSM1196117
SM014-R	SM014_Mock_2d_1_array	RNA	GEO	GSM1196118
SM014-R	SM014_Mock_2d_2_array	RNA	GEO	GSM1196119
SM014-R	SM014_Mock_2d_3_array	RNA	GEO	GSM1196120
SM014-R	SM014_Mock_4d_1_array	RNA	GEO	GSM1196121
SM014-R	SM014_Mock_4d_2_array	RNA	GEO	GSM1196122
SM014-R	SM014_Mock_4d_3_array	RNA	GEO	GSM1196123
SM014-R	SM014_Mock_7d_1_array	RNA	GEO	GSM1196124
SM014-R	SM014_Mock_7d_2_array	RNA	GEO	GSM1196125
SM014-R	SM014_Mock_7d_3_array	RNA	GEO	GSM1196126
SM014-R	SM014_nsp16_10^5pfu_1d_2_array	RNA	GEO	GSM1196127
SM014-R	SM014_nsp16_10^5pfu_1d_3_array	RNA	GEO	GSM1196128
SM014-R	SM014_nsp16_10^5pfu_1d_4_array	RNA	GEO	GSM1196129
SM014-R	SM014_nsp16_10^5pfu_2d_1_array	RNA	GEO	GSM1196130
SM014-R	SM014_nsp16_10^5pfu_2d_2_array	RNA	GEO	GSM1196131
SM014-R	SM014_nsp16_10^5pfu_2d_3_array	RNA	GEO	GSM1196132
SM014-R	SM014_nsp16_10^5pfu_2d_4_array	RNA	GEO	GSM1196133
SM014-R	SM014_nsp16_10^5pfu_4d_1_array	RNA	GEO	GSM1196134
SM014-R	SM014_nsp16_10^5pfu_4d_3_array	RNA	GEO	GSM1196135
SM014-R	SM014_nsp16_10^5pfu_4d_4_array	RNA	GEO	GSM1196136
SM014-R	SM014_nsp16_10^5pfu_7d_1_array	RNA	GEO	GSM1196137
SM014-R	SM014_nsp16_10^5pfu_7d_2_array	RNA	GEO	GSM1196138
SM014-R	SM014_nsp16_10^5pfu_7d_3_array	RNA	GEO	GSM1196139
SM014-R	SM014_nsp16_10^5pfu_7d_4_array	RNA	GEO	GSM1196140
SM015-R	SM015_B6_MA15_d4_2_array	RNA	GEO	GSM1003080
SM015-R	SM015_B6_MA15_d7_1_array	RNA	GEO	GSM1003081
SM015-R	SM015_B6_MA15_d7_2_array	RNA	GEO	GSM1003082
SM015-R	SM015_B6_MA15_d7_3_array	RNA	GEO	GSM1003083
SM015-R	SM015_B6_Mock_d4_1_array	RNA	GEO	GSM1003084
SM015-R	SM015_B6_Mock_d4_2_array	RNA	GEO	GSM1003085
SM015-R	SM015_B6_Mock_d7_1_array	RNA	GEO	GSM1003086
SM015-R	SM015_B6_Mock_d7_2_array	RNA	GEO	GSM1003087
SM015-R	SM015_B6_Mock_d7_3_array	RNA	GEO	GSM1003088
SM015-R	SM015_Tnfrsf1a/1b_MA15_d4_1_array	RNA	GEO	GSM1003089
SM015-R	SM015_Tnfrsf1a/1b_MA15_d4_2_array	RNA	GEO	GSM1003090
SM015-R	SM015_Tnfrsf1a/1b_MA15_d7_1_array	RNA	GEO	GSM1003091
SM015-R	SM015_Tnfrsf1a/1b_MA15_d7_2_array	RNA	GEO	GSM1003092
SM015-R	SM015_Tnfrsf1a/1b_MA15_d7_3_array	RNA	GEO	GSM1003093
SM015-R	SM015_Tnfrsf1a/1b_Mock_d4_1_array	RNA	GEO	GSM1003094
SM015-R	SM015_Tnfrsf1a/1b_Mock_d4_2_array	RNA	GEO	GSM1003095
SM015-R	SM015_Tnfrsf1a/1b_Mock_d7_1_array	RNA	GEO	GSM1003096
SM015-R	SM015_Tnfrsf1a/1b_Mock_d7_2_array	RNA	GEO	GSM1003097
SM015-R	SM015_Tnfrsf1a/1b_Mock_d7_3_array	RNA	GEO	GSM1003098
SM019-R	SM019_B6_MA15_d4_1_array	RNA	GEO	GSM1002567
SM019-R	SM019_B6_MA15_d4_2_array	RNA	GEO	GSM1002568
SM019-R	SM019_B6_MA15_d4_3_array	RNA	GEO	GSM1002569
SM019-R	SM019_B6_MA15_d7_1_array	RNA	GEO	GSM1002570
SM019-R	SM019_B6_MA15_d7_2_array	RNA	GEO	GSM1002571
SM019-R	SM019_B6_MA15_d7_3_array	RNA	GEO	GSM1002572
SM019-R	SM019_B6_Mock_d4_1_array	RNA	GEO	GSM1002573
SM019-R	SM019_B6_Mock_d4_2_array	RNA	GEO	GSM1002574
SM019-R	SM019_B6_Mock_d4_3_array	RNA	GEO	GSM1002575
SM019-R	SM019_B6_Mock_d7_1_array	RNA	GEO	GSM1002576
SM019-R	SM019_B6_Mock_d7_2_array	RNA	GEO	GSM1002577
SM019-R	SM019_B6_Mock_d7_3_array	RNA	GEO	GSM1002578
SM019-R	SM019_Tnfrsf1b_MA15_d4_1_array	RNA	GEO	GSM1002579
SM019-R	SM019_Tnfrsf1b_MA15_d4_2_array	RNA	GEO	GSM1002580
SM019-R	SM019_Tnfrsf1b_MA15_d4_3_array	RNA	GEO	GSM1002581
SM019-R	SM019_Tnfrsf1b_MA15_d7_2_array	RNA	GEO	GSM1002582
SM019-R	SM019_Tnfrsf1b_MA15_d7_3_array	RNA	GEO	GSM1002583
SM019-R	SM019_Tnfrsf1b_Mock_d4_1_array	RNA	GEO	GSM1002584
SM019-R	SM019_Tnfrsf1b_Mock_d4_2_array	RNA	GEO	GSM1002585
SM019-R	SM019_Tnfrsf1b_Mock_d4_3_array	RNA	GEO	GSM1002586
SM019-R	SM019_Tnfrsf1b_Mock_d7_2_array	RNA	GEO	GSM1002587
SM019-R	SM019_Tnfrsf1b_Mock_d7_3_array	RNA	GEO	GSM1002588
SM020-R	SM020_B6_MA15_d4_1_array	RNA	GEO	GSM1002603
SM020-R	SM020_B6_MA15_d4_2_array	RNA	GEO	GSM1002604
SM020-R	SM020_B6_MA15_d7_1_array	RNA	GEO	GSM1002605
SM020-R	SM020_B6_MA15_d7_2_array	RNA	GEO	GSM1002606
SM020-R	SM020_B6_MA15_d7_3_array	RNA	GEO	GSM1002607
SM020-R	SM020_B6_Mock_d4_1_array	RNA	GEO	GSM1002608
SM020-R	SM020_B6_Mock_d4_2_array	RNA	GEO	GSM1002609
SM020-R	SM020_B6_Mock_d7_1_array	RNA	GEO	GSM1002610
SM020-R	SM020_B6_Mock_d7_2_array	RNA	GEO	GSM1002611
SM020-R	SM020_B6_Mock_d7_3_array	RNA	GEO	GSM1002612
SM020-R	SM020_ppp1r14c_MA15_d4_1_array	RNA	GEO	GSM1002613
SM020-R	SM020_ppp1r14c_MA15_d4_2_array	RNA	GEO	GSM1002614
SM020-R	SM020_ppp1r14c_MA15_d7_2_array	RNA	GEO	GSM1002615
SM020-R	SM020_ppp1r14c_MA15_d7_3_array	RNA	GEO	GSM1002616
SM020-R	SM020_ppp1r14c_Mock_d4_1_array	RNA	GEO	GSM1002617
SM020-R	SM020_ppp1r14c_Mock_d4_2_array	RNA	GEO	GSM1002618
SM020-R	SM020_ppp1r14c_Mock_d7_1_array	RNA	GEO	GSM1002619
SM020-R	SM020_ppp1r14c_Mock_d7_2_array	RNA	GEO	GSM1002620
SM020-R	SM020_ppp1r14c_Mock_d7_3_array	RNA	GEO	GSM1002621

**Table 3:** Experiment samples from proteomic assays

**Experiment ID**	**Experiment Sample ID**	**Biological Sample Type**	**Public Repository Name**
CA04M001-P	CA04M001_CA04_10^4_1d_1_proteomics	Protein	PeptideAtlas
CA04M001-P	CA04M001_CA04_10^4_1d_2_proteomics	Protein	PeptideAtlas
CA04M001-P	CA04M001_CA04_10^4_1d_3_proteomics	Protein	PeptideAtlas
CA04M001-P	CA04M001_CA04_10^4_1d_4_proteomics	Protein	PeptideAtlas
CA04M001-P	CA04M001_CA04_10^4_2d_1_proteomics	Protein	PeptideAtlas
CA04M001-P	CA04M001_CA04_10^4_2d_2_proteomics	Protein	PeptideAtlas
CA04M001-P	CA04M001_CA04_10^4_2d_3_proteomics	Protein	PeptideAtlas
CA04M001-P	CA04M001_CA04_10^4_2d_4_proteomics	Protein	PeptideAtlas
CA04M001-P	CA04M001_CA04_10^4_4d_1_proteomics	Protein	PeptideAtlas
CA04M001-P	CA04M001_CA04_10^4_4d_2_proteomics	Protein	PeptideAtlas
CA04M001-P	CA04M001_CA04_10^4_4d_3_proteomics	Protein	PeptideAtlas
CA04M001-P	CA04M001_CA04_10^4_7d_1_proteomics	Protein	PeptideAtlas
CA04M001-P	CA04M001_CA04_10^4_7d_2_proteomics	Protein	PeptideAtlas
CA04M001-P	CA04M001_CA04_10^4_7d_3_proteomics	Protein	PeptideAtlas
CA04M001-P	CA04M001_CA04_10^5_1d_1_proteomics	Protein	PeptideAtlas
CA04M001-P	CA04M001_CA04_10^5_1d_2_proteomics	Protein	PeptideAtlas
CA04M001-P	CA04M001_CA04_10^5_1d_3_proteomics	Protein	PeptideAtlas
CA04M001-P	CA04M001_CA04_10^5_1d_4_proteomics	Protein	PeptideAtlas
CA04M001-P	CA04M001_CA04_10^5_2d_1_proteomics	Protein	PeptideAtlas
CA04M001-P	CA04M001_CA04_10^5_2d_2_proteomics	Protein	PeptideAtlas
CA04M001-P	CA04M001_CA04_10^5_2d_3_proteomics	Protein	PeptideAtlas
CA04M001-P	CA04M001_CA04_10^5_2d_4_proteomics	Protein	PeptideAtlas
CA04M001-P	CA04M001_CA04_10^5_4d_1_proteomics	Protein	PeptideAtlas
CA04M001-P	CA04M001_CA04_10^5_4d_2_proteomics	Protein	PeptideAtlas
CA04M001-P	CA04M001_CA04_10^5_4d_3_proteomics	Protein	PeptideAtlas
CA04M001-P	CA04M001_CA04_10^5_4d_4_proteomics	Protein	PeptideAtlas
CA04M001-P	CA04M001_CA04_10^5_7d_1_proteomics	Protein	PeptideAtlas
CA04M001-P	CA04M001_CA04_10^5_7d_2_proteomics	Protein	PeptideAtlas
CA04M001-P	CA04M001_CA04_10^5_7d_3_proteomics	Protein	PeptideAtlas
CA04M001-P	CA04M001_CA04_10^5_7d_4_proteomics	Protein	PeptideAtlas
CA04M001-P	CA04M001_CA04_10^5_7d_5_proteomics	Protein	PeptideAtlas
CA04M001-P	CA04M001_Mock_1d_1_proteomics	Protein	PeptideAtlas
CA04M001-P	CA04M001_Mock_1d_2_proteomics	Protein	PeptideAtlas
CA04M001-P	CA04M001_Mock_2d_1_proteomics	Protein	PeptideAtlas
CA04M001-P	CA04M001_Mock_4d_1_proteomics	Protein	PeptideAtlas
CA04M001-P	CA04M001_Mock_4d_2_proteomics	Protein	PeptideAtlas
CA04M001-P	CA04M001_Mock_4d_3_proteomics	Protein	PeptideAtlas
CA04M001-P	CA04M001_Mock_7d_1_proteomics	Protein	PeptideAtlas
CA04M001-P	CA04M001_Mock_7d_2_proteomics	Protein	PeptideAtlas
CA04M001-P	CA04M001_Mock_7d_3_proteomics	Protein	PeptideAtlas
ICL004-P	ICL004_Mock_0h_1_proteomics	Protein	PeptideAtlas
ICL004-P	ICL004_Mock_0h_2_proteomics	Protein	PeptideAtlas
ICL004-P	ICL004_Mock_0h_3_proteomics	Protein	PeptideAtlas
ICL004-P	ICL004_Mock_12h_1_proteomics	Protein	PeptideAtlas
ICL004-P	ICL004_Mock_12h_2_proteomics	Protein	PeptideAtlas
ICL004-P	ICL004_Mock_12h_3_proteomics	Protein	PeptideAtlas
ICL004-P	ICL004_Mock_18h_1_proteomics	Protein	PeptideAtlas
ICL004-P	ICL004_Mock_18h_2_proteomics	Protein	PeptideAtlas
ICL004-P	ICL004_Mock_18h_3_proteomics	Protein	PeptideAtlas
ICL004-P	ICL004_Mock_24h_1_proteomics	Protein	PeptideAtlas
ICL004-P	ICL004_Mock_24h_3_proteomics	Protein	PeptideAtlas
ICL004-P	ICL004_Mock_3h_1_proteomics	Protein	PeptideAtlas
ICL004-P	ICL004_Mock_7h_1_proteomics	Protein	PeptideAtlas
ICL004-P	ICL004_Mock_7h_2_proteomics	Protein	PeptideAtlas
ICL004-P	ICL004_Mock_7h_3_proteomics	Protein	PeptideAtlas
ICL004-P	ICL004_VN1203_0h_1_proteomics	Protein	PeptideAtlas
ICL004-P	ICL004_VN1203_0h_2_proteomics	Protein	PeptideAtlas
ICL004-P	ICL004_VN1203_12h_2_proteomics	Protein	PeptideAtlas
ICL004-P	ICL004_VN1203_12h_3_proteomics	Protein	PeptideAtlas
ICL004-P	ICL004_VN1203_18h_1_proteomics	Protein	PeptideAtlas
ICL004-P	ICL004_VN1203_18h_2_proteomics	Protein	PeptideAtlas
ICL004-P	ICL004_VN1203_18h_3_proteomics	Protein	PeptideAtlas
ICL004-P	ICL004_VN1203_24h_1_proteomics	Protein	PeptideAtlas
ICL004-P	ICL004_VN1203_24h_2_proteomics	Protein	PeptideAtlas
ICL004-P	ICL004_VN1203_3h_1_proteomics	Protein	PeptideAtlas
ICL004-P	ICL004_VN1203_3h_2_proteomics	Protein	PeptideAtlas
ICL004-P	ICL004_VN1203_7h_1_proteomics	Protein	PeptideAtlas
ICL004-P	ICL004_VN1203_7h_2_proteomics	Protein	PeptideAtlas
ICL004-P	ICL004_VN1203_7h_3_proteomics	Protein	PeptideAtlas
ICL006-P	ICL006_CA04_0h_1_proteomics	Protein	PeptideAtlas
ICL006-P	ICL006_CA04_0h_2_proteomics	Protein	PeptideAtlas
ICL006-P	ICL006_CA04_0h_3_proteomics	Protein	PeptideAtlas
ICL006-P	ICL006_CA04_12h_1_proteomics	Protein	PeptideAtlas
ICL006-P	ICL006_CA04_12h_2_proteomics	Protein	PeptideAtlas
ICL006-P	ICL006_CA04_12h_3_proteomics	Protein	PeptideAtlas
ICL006-P	ICL006_CA04_18h_1_proteomics	Protein	PeptideAtlas
ICL006-P	ICL006_CA04_18h_2_proteomics	Protein	PeptideAtlas
ICL006-P	ICL006_CA04_18h_3_proteomics	Protein	PeptideAtlas
ICL006-P	ICL006_CA04_24h_1_proteomics	Protein	PeptideAtlas
ICL006-P	ICL006_CA04_24h_2_proteomics	Protein	PeptideAtlas
ICL006-P	ICL006_CA04_24h_3_proteomics	Protein	PeptideAtlas
ICL006-P	ICL006_CA04_30h_1_proteomics	Protein	PeptideAtlas
ICL006-P	ICL006_CA04_30h_2_proteomics	Protein	PeptideAtlas
ICL006-P	ICL006_CA04_30h_3_proteomics	Protein	PeptideAtlas
ICL006-P	ICL006_CA04_36h_1_proteomics	Protein	PeptideAtlas
ICL006-P	ICL006_CA04_36h_2_proteomics	Protein	PeptideAtlas
ICL006-P	ICL006_CA04_36h_3_proteomics	Protein	PeptideAtlas
ICL006-P	ICL006_CA04_3h_1_proteomics	Protein	PeptideAtlas
ICL006-P	ICL006_CA04_3h_2_proteomics	Protein	PeptideAtlas
ICL006-P	ICL006_CA04_3h_3_proteomics	Protein	PeptideAtlas
ICL006-P	ICL006_CA04_48h_1_proteomics	Protein	PeptideAtlas
ICL006-P	ICL006_CA04_48h_2_proteomics	Protein	PeptideAtlas
ICL006-P	ICL006_CA04_48h_3_proteomics	Protein	PeptideAtlas
ICL006-P	ICL006_CA04_7h_1_proteomics	Protein	PeptideAtlas
ICL006-P	ICL006_CA04_7h_2_proteomics	Protein	PeptideAtlas
ICL006-P	ICL006_CA04_7h_3_proteomics	Protein	PeptideAtlas
ICL006-P	ICL006_Mock_0h_1_proteomics	Protein	PeptideAtlas
ICL006-P	ICL006_Mock_0h_2_proteomics	Protein	PeptideAtlas
ICL006-P	ICL006_Mock_0h_3_proteomics	Protein	PeptideAtlas
ICL006-P	ICL006_Mock_12h_1_proteomics	Protein	PeptideAtlas
ICL006-P	ICL006_Mock_12h_2_proteomics	Protein	PeptideAtlas
ICL006-P	ICL006_Mock_12h_3_proteomics	Protein	PeptideAtlas
ICL006-P	ICL006_Mock_18h_1_proteomics	Protein	PeptideAtlas
ICL006-P	ICL006_Mock_18h_2_proteomics	Protein	PeptideAtlas
ICL006-P	ICL006_Mock_18h_3_proteomics	Protein	PeptideAtlas
ICL006-P	ICL006_Mock_24h_1_proteomics	Protein	PeptideAtlas
ICL006-P	ICL006_Mock_24h_2_proteomics	Protein	PeptideAtlas
ICL006-P	ICL006_Mock_24h_3_proteomics	Protein	PeptideAtlas
ICL006-P	ICL006_Mock_30h_1_proteomics	Protein	PeptideAtlas
ICL006-P	ICL006_Mock_30h_2_proteomics	Protein	PeptideAtlas
ICL006-P	ICL006_Mock_30h_3_proteomics	Protein	PeptideAtlas
ICL006-P	ICL006_Mock_36h_1_proteomics	Protein	PeptideAtlas
ICL006-P	ICL006_Mock_36h_2_proteomics	Protein	PeptideAtlas
ICL006-P	ICL006_Mock_36h_3_proteomics	Protein	PeptideAtlas
ICL006-P	ICL006_Mock_3h_1_proteomics	Protein	PeptideAtlas
ICL006-P	ICL006_Mock_3h_2_proteomics	Protein	PeptideAtlas
ICL006-P	ICL006_Mock_3h_3_proteomics	Protein	PeptideAtlas
ICL006-P	ICL006_Mock_48h_1_proteomics	Protein	PeptideAtlas
ICL006-P	ICL006_Mock_48h_2_proteomics	Protein	PeptideAtlas
ICL006-P	ICL006_Mock_48h_3_proteomics	Protein	PeptideAtlas
ICL006-P	ICL006_Mock_7h_1_proteomics	Protein	PeptideAtlas
ICL006-P	ICL006_Mock_7h_2_proteomics	Protein	PeptideAtlas
ICL006-P	ICL006_Mock_7h_3_proteomics	Protein	PeptideAtlas
ICL010-P	ICL010_mock_0h_1_Proteomics	Protein	PeptideAtlas
ICL010-P	ICL010_mock_0h_2_Proteomics	Protein	PeptideAtlas
ICL010-P	ICL010_mock_0h_3_Proteomics	Protein	PeptideAtlas
ICL010-P	ICL010_mock_12h_1_Proteomics	Protein	PeptideAtlas
ICL010-P	ICL010_mock_12h_2_Proteomics	Protein	PeptideAtlas
ICL010-P	ICL010_mock_12h_3_Proteomics	Protein	PeptideAtlas
ICL010-P	ICL010_mock_18h_1_Proteomics	Protein	PeptideAtlas
ICL010-P	ICL010_mock_18h_2_Proteomics	Protein	PeptideAtlas
ICL010-P	ICL010_mock_18h_3_Proteomics	Protein	PeptideAtlas
ICL010-P	ICL010_mock_24h_1_Proteomics	Protein	PeptideAtlas
ICL010-P	ICL010_mock_24h_2_Proteomics	Protein	PeptideAtlas
ICL010-P	ICL010_mock_24h_3_Proteomics	Protein	PeptideAtlas
ICL010-P	ICL010_mock_30h_1_Proteomics	Protein	PeptideAtlas
ICL010-P	ICL010_mock_30h_2_Proteomics	Protein	PeptideAtlas
ICL010-P	ICL010_mock_30h_3_Proteomics	Protein	PeptideAtlas
ICL010-P	ICL010_mock_36h_1_Proteomics	Protein	PeptideAtlas
ICL010-P	ICL010_mock_36h_2_Proteomics	Protein	PeptideAtlas
ICL010-P	ICL010_mock_36h_3_Proteomics	Protein	PeptideAtlas
ICL010-P	ICL010_mock_3h_1_Proteomics	Protein	PeptideAtlas
ICL010-P	ICL010_mock_3h_2_Proteomics	Protein	PeptideAtlas
ICL010-P	ICL010_mock_3h_3_Proteomics	Protein	PeptideAtlas
ICL010-P	ICL010_mock_48h_1_Proteomics	Protein	PeptideAtlas
ICL010-P	ICL010_mock_48h_2_Proteomics	Protein	PeptideAtlas
ICL010-P	ICL010_mock_48h_3_Proteomics	Protein	PeptideAtlas
ICL010-P	ICL010_mock_7h_1_Proteomics	Protein	PeptideAtlas
ICL010-P	ICL010_mock_7h_2_Proteomics	Protein	PeptideAtlas
ICL010-P	ICL010_mock_7h_3_Proteomics	Protein	PeptideAtlas
ICL010-P	ICL010_NL_0h_1_Proteomics	Protein	PeptideAtlas
ICL010-P	ICL010_NL_0h_2_Proteomics	Protein	PeptideAtlas
ICL010-P	ICL010_NL_0h_3_Proteomics	Protein	PeptideAtlas
ICL010-P	ICL010_NL_12h_1_Proteomics	Protein	PeptideAtlas
ICL010-P	ICL010_NL_12h_2_Proteomics	Protein	PeptideAtlas
ICL010-P	ICL010_NL_12h_3_Proteomics	Protein	PeptideAtlas
ICL010-P	ICL010_NL_18h_1_Proteomics	Protein	PeptideAtlas
ICL010-P	ICL010_NL_18h_2_Proteomics	Protein	PeptideAtlas
ICL010-P	ICL010_NL_18h_3_Proteomics	Protein	PeptideAtlas
ICL010-P	ICL010_NL_24h_1_Proteomics	Protein	PeptideAtlas
ICL010-P	ICL010_NL_24h_2_Proteomics	Protein	PeptideAtlas
ICL010-P	ICL010_NL_24h_3_Proteomics	Protein	PeptideAtlas
ICL010-P	ICL010_NL_30h_1_Proteomics	Protein	PeptideAtlas
ICL010-P	ICL010_NL_30h_2_Proteomics	Protein	PeptideAtlas
ICL010-P	ICL010_NL_30h_3_Proteomics	Protein	PeptideAtlas
ICL010-P	ICL010_NL_36h_1_Proteomics	Protein	PeptideAtlas
ICL010-P	ICL010_NL_36h_2_Proteomics	Protein	PeptideAtlas
ICL010-P	ICL010_NL_36h_3_Proteomics	Protein	PeptideAtlas
ICL010-P	ICL010_NL_3h_1_Proteomics	Protein	PeptideAtlas
ICL010-P	ICL010_NL_3h_2_Proteomics	Protein	PeptideAtlas
ICL010-P	ICL010_NL_3h_3_Proteomics	Protein	PeptideAtlas
ICL010-P	ICL010_NL_48h_1_Proteomics	Protein	PeptideAtlas
ICL010-P	ICL010_NL_48h_2_Proteomics	Protein	PeptideAtlas
ICL010-P	ICL010_NL_48h_3_Proteomics	Protein	PeptideAtlas
ICL010-P	ICL010_NL_7h_1_Proteomics	Protein	PeptideAtlas
ICL010-P	ICL010_NL_7h_2_Proteomics	Protein	PeptideAtlas
ICL010-P	ICL010_NL_7h_3_Proteomics	Protein	PeptideAtlas
ICL011-P	ICL011_mock_0h_1_proteomics	Protein	PeptideAtlas
ICL011-P	ICL011_mock_0h_2_proteomics	Protein	PeptideAtlas
ICL011-P	ICL011_mock_0h_3_proteomics	Protein	PeptideAtlas
ICL011-P	ICL011_mock_12h_1_proteomics	Protein	PeptideAtlas
ICL011-P	ICL011_mock_12h_2_proteomics	Protein	PeptideAtlas
ICL011-P	ICL011_mock_12h_3_proteomics	Protein	PeptideAtlas
ICL011-P	ICL011_mock_18h_1_proteomics	Protein	PeptideAtlas
ICL011-P	ICL011_mock_18h_2_proteomics	Protein	PeptideAtlas
ICL011-P	ICL011_mock_18h_3_proteomics	Protein	PeptideAtlas
ICL011-P	ICL011_mock_24h_1_proteomics	Protein	PeptideAtlas
ICL011-P	ICL011_mock_24h_2_proteomics	Protein	PeptideAtlas
ICL011-P	ICL011_mock_24h_3_proteomics	Protein	PeptideAtlas
ICL011-P	ICL011_mock_3h_1_proteomics	Protein	PeptideAtlas
ICL011-P	ICL011_mock_3h_2_proteomics	Protein	PeptideAtlas
ICL011-P	ICL011_mock_3h_3_proteomics	Protein	PeptideAtlas
ICL011-P	ICL011_mock_7h_1_proteomics	Protein	PeptideAtlas
ICL011-P	ICL011_mock_7h_2_proteomics	Protein	PeptideAtlas
ICL011-P	ICL011_mock_7h_3_proteomics	Protein	PeptideAtlas
ICL011-P	ICL011_VN-PB1-F2-del_0h_1_proteomics	Protein	PeptideAtlas
ICL011-P	ICL011_VN-PB1-F2-del_0h_2_proteomics	Protein	PeptideAtlas
ICL011-P	ICL011_VN-PB1-F2-del_0h_3_proteomics	Protein	PeptideAtlas
ICL011-P	ICL011_VN-PB1-F2-del_12h_1_proteomics	Protein	PeptideAtlas
ICL011-P	ICL011_VN-PB1-F2-del_12h_2_proteomics	Protein	PeptideAtlas
ICL011-P	ICL011_VN-PB1-F2-del_12h_3_proteomics	Protein	PeptideAtlas
ICL011-P	ICL011_VN-PB1-F2-del_18h_1_proteomics	Protein	PeptideAtlas
ICL011-P	ICL011_VN-PB1-F2-del_18h_2_proteomics	Protein	PeptideAtlas
ICL011-P	ICL011_VN-PB1-F2-del_18h_3_proteomics	Protein	PeptideAtlas
ICL011-P	ICL011_VN-PB1-F2-del_24h_1_proteomics	Protein	PeptideAtlas
ICL011-P	ICL011_VN-PB1-F2-del_24h_2_proteomics	Protein	PeptideAtlas
ICL011-P	ICL011_VN-PB1-F2-del_24h_3_proteomics	Protein	PeptideAtlas
ICL011-P	ICL011_VN-PB1-F2-del_3h_1_proteomics	Protein	PeptideAtlas
ICL011-P	ICL011_VN-PB1-F2-del_3h_2_proteomics	Protein	PeptideAtlas
ICL011-P	ICL011_VN-PB1-F2-del_3h_3_proteomics	Protein	PeptideAtlas
ICL011-P	ICL011_VN-PB1-F2-del_7h_1_proteomics	Protein	PeptideAtlas
ICL011-P	ICL011_VN-PB1-F2-del_7h_2_proteomics	Protein	PeptideAtlas
ICL011-P	ICL011_VN-PB1-F2-del_7h_3_proteomics	Protein	PeptideAtlas
ICL011-P	ICL011_VN-PB2-627E_0h_1_proteomics	Protein	PeptideAtlas
ICL011-P	ICL011_VN-PB2-627E_0h_2_proteomics	Protein	PeptideAtlas
ICL011-P	ICL011_VN-PB2-627E_0h_3_proteomics	Protein	PeptideAtlas
ICL011-P	ICL011_VN-PB2-627E_12h_1_proteomics	Protein	PeptideAtlas
ICL011-P	ICL011_VN-PB2-627E_12h_2_proteomics	Protein	PeptideAtlas
ICL011-P	ICL011_VN-PB2-627E_12h_3_proteomics	Protein	PeptideAtlas
ICL011-P	ICL011_VN-PB2-627E_18h_1_proteomics	Protein	PeptideAtlas
ICL011-P	ICL011_VN-PB2-627E_18h_2_proteomics	Protein	PeptideAtlas
ICL011-P	ICL011_VN-PB2-627E_18h_3_proteomics	Protein	PeptideAtlas
ICL011-P	ICL011_VN-PB2-627E_24h_1_proteomics	Protein	PeptideAtlas
ICL011-P	ICL011_VN-PB2-627E_24h_2_proteomics	Protein	PeptideAtlas
ICL011-P	ICL011_VN-PB2-627E_24h_3_proteomics	Protein	PeptideAtlas
ICL011-P	ICL011_VN-PB2-627E_3h_1_proteomics	Protein	PeptideAtlas
ICL011-P	ICL011_VN-PB2-627E_3h_2_proteomics	Protein	PeptideAtlas
ICL011-P	ICL011_VN-PB2-627E_3h_3_proteomics	Protein	PeptideAtlas
ICL011-P	ICL011_VN-PB2-627E_7h_1_proteomics	Protein	PeptideAtlas
ICL011-P	ICL011_VN-PB2-627E_7h_2_proteomics	Protein	PeptideAtlas
ICL011-P	ICL011_VN-PB2-627E_7h_3_proteomics	Protein	PeptideAtlas
ICL011-P	ICL011_VN1203_24h_1_proteomics	Protein	PeptideAtlas
ICL011-P	ICL011_VN1203_24h_2_proteomics	Protein	PeptideAtlas
ICL011-P	ICL011_VN1203_24h_3_proteomics	Protein	PeptideAtlas
ICL011-P	ICL011_VN1203_7h_1_proteomics	Protein	PeptideAtlas
ICL011-P	ICL011_VN1203_7h_2_proteomics	Protein	PeptideAtlas
ICL011-P	ICL011_VN1203_7h_3_proteomics	Protein	PeptideAtlas
ICL012-P	ICL012_mock_0h_1_proteomics	Protein	PeptideAtlas
ICL012-P	ICL012_mock_0h_2_proteomics	Protein	PeptideAtlas
ICL012-P	ICL012_mock_0h_3_proteomics	Protein	PeptideAtlas
ICL012-P	ICL012_mock_12h_1_proteomics	Protein	PeptideAtlas
ICL012-P	ICL012_mock_12h_2_proteomics	Protein	PeptideAtlas
ICL012-P	ICL012_mock_12h_3_proteomics	Protein	PeptideAtlas
ICL012-P	ICL012_mock_18h_1_proteomics	Protein	PeptideAtlas
ICL012-P	ICL012_mock_18h_2_proteomics	Protein	PeptideAtlas
ICL012-P	ICL012_mock_18h_3_proteomics	Protein	PeptideAtlas
ICL012-P	ICL012_mock_24h_1_proteomics	Protein	PeptideAtlas
ICL012-P	ICL012_mock_24h_2_proteomics	Protein	PeptideAtlas
ICL012-P	ICL012_mock_24h_3_proteomics	Protein	PeptideAtlas
ICL012-P	ICL012_mock_3h_1_proteomics	Protein	PeptideAtlas
ICL012-P	ICL012_mock_3h_2_proteomics	Protein	PeptideAtlas
ICL012-P	ICL012_mock_3h_3_proteomics	Protein	PeptideAtlas
ICL012-P	ICL012_mock_7h_1_proteomics	Protein	PeptideAtlas
ICL012-P	ICL012_mock_7h_2_proteomics	Protein	PeptideAtlas
ICL012-P	ICL012_mock_7h_3_proteomics	Protein	PeptideAtlas
ICL012-P	ICL012_NS1trunc_0h_1_proteomics	Protein	PeptideAtlas
ICL012-P	ICL012_NS1trunc_0h_2_proteomics	Protein	PeptideAtlas
ICL012-P	ICL012_NS1trunc_0h_3_proteomics	Protein	PeptideAtlas
ICL012-P	ICL012_NS1trunc_12h_1_proteomics	Protein	PeptideAtlas
ICL012-P	ICL012_NS1trunc_12h_2_proteomics	Protein	PeptideAtlas
ICL012-P	ICL012_NS1trunc_12h_3_proteomics	Protein	PeptideAtlas
ICL012-P	ICL012_NS1trunc_18h_1_proteomics	Protein	PeptideAtlas
ICL012-P	ICL012_NS1trunc_18h_2_proteomics	Protein	PeptideAtlas
ICL012-P	ICL012_NS1trunc_18h_3_proteomics	Protein	PeptideAtlas
ICL012-P	ICL012_NS1trunc_24h_1_proteomics	Protein	PeptideAtlas
ICL012-P	ICL012_NS1trunc_24h_2_proteomics	Protein	PeptideAtlas
ICL012-P	ICL012_NS1trunc_24h_3_proteomics	Protein	PeptideAtlas
ICL012-P	ICL012_NS1trunc_3h_1_proteomics	Protein	PeptideAtlas
ICL012-P	ICL012_NS1trunc_3h_2_proteomics	Protein	PeptideAtlas
ICL012-P	ICL012_NS1trunc_3h_3_proteomics	Protein	PeptideAtlas
ICL012-P	ICL012_NS1trunc_7h_1_proteomics	Protein	PeptideAtlas
ICL012-P	ICL012_NS1trunc_7h_2_proteomics	Protein	PeptideAtlas
ICL012-P	ICL012_NS1trunc_7h_3_proteomics	Protein	PeptideAtlas
ICL012-P	ICL012_VN1203_24h_1_proteomics	Protein	PeptideAtlas
ICL012-P	ICL012_VN1203_24h_2_proteomics	Protein	PeptideAtlas
ICL012-P	ICL012_VN1203_24h_3_proteomics	Protein	PeptideAtlas
ICL012-P	ICL012_VN1203_7h_1_proteomics	Protein	PeptideAtlas
ICL012-P	ICL012_VN1203_7h_2_proteomics	Protein	PeptideAtlas
ICL012-P	ICL012_VN1203_7h_3_proteomics	Protein	PeptideAtlas
IM001-P	IM001_Mock_1d_1_proteomics	Protein	PeptideAtlas
IM001-P	IM001_Mock_1d_2_proteomics	Protein	PeptideAtlas
IM001-P	IM001_Mock_1d_3_proteomics	Protein	PeptideAtlas
IM001-P	IM001_Mock_2d_1_proteomics	Protein	PeptideAtlas
IM001-P	IM001_Mock_2d_2_proteomics	Protein	PeptideAtlas
IM001-P	IM001_Mock_2d_3_proteomics	Protein	PeptideAtlas
IM001-P	IM001_Mock_4d_1_proteomics	Protein	PeptideAtlas
IM001-P	IM001_Mock_4d_2_proteomics	Protein	PeptideAtlas
IM001-P	IM001_Mock_4d_3_proteomics	Protein	PeptideAtlas
IM001-P	IM001_Mock_7d_1_proteomics	Protein	PeptideAtlas
IM001-P	IM001_Mock_7d_2_proteomics	Protein	PeptideAtlas
IM001-P	IM001_Mock_7d_3_proteomics	Protein	PeptideAtlas
IM001-P	IM001_VN1203_10^2_1d_1_proteomics	Protein	PeptideAtlas
IM001-P	IM001_VN1203_10^2_1d_2_proteomics	Protein	PeptideAtlas
IM001-P	IM001_VN1203_10^2_1d_3_proteomics	Protein	PeptideAtlas
IM001-P	IM001_VN1203_10^2_1d_4_proteomics	Protein	PeptideAtlas
IM001-P	IM001_VN1203_10^2_1d_5_proteomics	Protein	PeptideAtlas
IM001-P	IM001_VN1203_10^2_2d_1_proteomics	Protein	PeptideAtlas
IM001-P	IM001_VN1203_10^2_2d_2_proteomics	Protein	PeptideAtlas
IM001-P	IM001_VN1203_10^2_2d_3_proteomics	Protein	PeptideAtlas
IM001-P	IM001_VN1203_10^2_2d_4_proteomics	Protein	PeptideAtlas
IM001-P	IM001_VN1203_10^2_2d_5_proteomics	Protein	PeptideAtlas
IM001-P	IM001_VN1203_10^2_4d_1_proteomics	Protein	PeptideAtlas
IM001-P	IM001_VN1203_10^2_4d_2_proteomics	Protein	PeptideAtlas
IM001-P	IM001_VN1203_10^2_4d_3_proteomics	Protein	PeptideAtlas
IM001-P	IM001_VN1203_10^2_4d_4_proteomics	Protein	PeptideAtlas
IM001-P	IM001_VN1203_10^2_4d_5_proteomics	Protein	PeptideAtlas
IM001-P	IM001_VN1203_10^2_7d_1_proteomics	Protein	PeptideAtlas
IM001-P	IM001_VN1203_10^2_7d_2_proteomics	Protein	PeptideAtlas
IM001-P	IM001_VN1203_10^2_7d_3_proteomics	Protein	PeptideAtlas
IM001-P	IM001_VN1203_10^2_7d_4_proteomics	Protein	PeptideAtlas
IM001-P	IM001_VN1203_10^3_1d_1_proteomics	Protein	PeptideAtlas
IM001-P	IM001_VN1203_10^3_1d_2_proteomics	Protein	PeptideAtlas
IM001-P	IM001_VN1203_10^3_1d_3_proteomics	Protein	PeptideAtlas
IM001-P	IM001_VN1203_10^3_1d_4_proteomics	Protein	PeptideAtlas
IM001-P	IM001_VN1203_10^3_1d_5_proteomics	Protein	PeptideAtlas
IM001-P	IM001_VN1203_10^3_2d_1_proteomics	Protein	PeptideAtlas
IM001-P	IM001_VN1203_10^3_2d_2_proteomics	Protein	PeptideAtlas
IM001-P	IM001_VN1203_10^3_2d_3_proteomics	Protein	PeptideAtlas
IM001-P	IM001_VN1203_10^3_2d_4_proteomics	Protein	PeptideAtlas
IM001-P	IM001_VN1203_10^3_2d_5_proteomics	Protein	PeptideAtlas
IM001-P	IM001_VN1203_10^3_4d_1_proteomics	Protein	PeptideAtlas
IM001-P	IM001_VN1203_10^3_4d_2_proteomics	Protein	PeptideAtlas
IM001-P	IM001_VN1203_10^3_4d_3_proteomics	Protein	PeptideAtlas
IM001-P	IM001_VN1203_10^3_4d_4_proteomics	Protein	PeptideAtlas
IM001-P	IM001_VN1203_10^3_4d_5_proteomics	Protein	PeptideAtlas
IM001-P	IM001_VN1203_10^3_7d_1_proteomics	Protein	PeptideAtlas
IM001-P	IM001_VN1203_10^3_7d_2_proteomics	Protein	PeptideAtlas
IM001-P	IM001_VN1203_10^3_7d_3_proteomics	Protein	PeptideAtlas
IM001-P	IM001_VN1203_10^4_1d_1_proteomics	Protein	PeptideAtlas
IM001-P	IM001_VN1203_10^4_1d_2_proteomics	Protein	PeptideAtlas
IM001-P	IM001_VN1203_10^4_1d_3_proteomics	Protein	PeptideAtlas
IM001-P	IM001_VN1203_10^4_1d_4_proteomics	Protein	PeptideAtlas
IM001-P	IM001_VN1203_10^4_1d_5_proteomics	Protein	PeptideAtlas
IM001-P	IM001_VN1203_10^4_2d_1_proteomics	Protein	PeptideAtlas
IM001-P	IM001_VN1203_10^4_2d_2_proteomics	Protein	PeptideAtlas
IM001-P	IM001_VN1203_10^4_2d_3_proteomics	Protein	PeptideAtlas
IM001-P	IM001_VN1203_10^4_2d_4_proteomics	Protein	PeptideAtlas
IM001-P	IM001_VN1203_10^4_2d_5_proteomics	Protein	PeptideAtlas
IM001-P	IM001_VN1203_10^4_4d_1_proteomics	Protein	PeptideAtlas
IM001-P	IM001_VN1203_10^4_4d_2_proteomics	Protein	PeptideAtlas
IM001-P	IM001_VN1203_10^4_4d_3_proteomics	Protein	PeptideAtlas
IM001-P	IM001_VN1203_10^4_4d_4_proteomics	Protein	PeptideAtlas
IM001-P	IM001_VN1203_10^4_4d_5_proteomics	Protein	PeptideAtlas
IM004-P	IM004_HAavir_10^4_1d_1_proteomics	Protein	PeptideAtlas
IM004-P	IM004_HAavir_10^4_1d_2_proteomics	Protein	PeptideAtlas
IM004-P	IM004_HAavir_10^4_1d_3_proteomics	Protein	PeptideAtlas
IM004-P	IM004_HAavir_10^4_1d_4_proteomics	Protein	PeptideAtlas
IM004-P	IM004_HAavir_10^4_1d_5_proteomics	Protein	PeptideAtlas
IM004-P	IM004_HAavir_10^4_2d_1_proteomics	Protein	PeptideAtlas
IM004-P	IM004_HAavir_10^4_2d_2_proteomics	Protein	PeptideAtlas
IM004-P	IM004_HAavir_10^4_2d_3_proteomics	Protein	PeptideAtlas
IM004-P	IM004_HAavir_10^4_2d_4_proteomics	Protein	PeptideAtlas
IM004-P	IM004_HAavir_10^4_2d_5_proteomics	Protein	PeptideAtlas
IM004-P	IM004_HAavir_10^4_4d_1_proteomics	Protein	PeptideAtlas
IM004-P	IM004_HAavir_10^4_4d_2_proteomics	Protein	PeptideAtlas
IM004-P	IM004_HAavir_10^4_4d_3_proteomics	Protein	PeptideAtlas
IM004-P	IM004_HAavir_10^4_4d_4_proteomics	Protein	PeptideAtlas
IM004-P	IM004_HAavir_10^4_4d_5_proteomics	Protein	PeptideAtlas
IM004-P	IM004_HAavir_10^4_7d_1_proteomics	Protein	PeptideAtlas
IM004-P	IM004_HAavir_10^4_7d_2_proteomics	Protein	PeptideAtlas
IM004-P	IM004_HAavir_10^4_7d_3_proteomics	Protein	PeptideAtlas
IM004-P	IM004_HAavir_10^4_7d_4_proteomics	Protein	PeptideAtlas
IM004-P	IM004_HAavir_10^4_7d_5_proteomics	Protein	PeptideAtlas
IM004-P	IM004_Mock_1d_1_proteomics	Protein	PeptideAtlas
IM004-P	IM004_Mock_1d_2_proteomics	Protein	PeptideAtlas
IM004-P	IM004_Mock_1d_3_proteomics	Protein	PeptideAtlas
IM004-P	IM004_Mock_2d_1_proteomics	Protein	PeptideAtlas
IM004-P	IM004_Mock_2d_2_proteomics	Protein	PeptideAtlas
IM004-P	IM004_Mock_2d_3_proteomics	Protein	PeptideAtlas
IM004-P	IM004_Mock_4d_1_proteomics	Protein	PeptideAtlas
IM004-P	IM004_Mock_4d_2_proteomics	Protein	PeptideAtlas
IM004-P	IM004_Mock_4d_3_proteomics	Protein	PeptideAtlas
IM004-P	IM004_Mock_7d_1_proteomics	Protein	PeptideAtlas
IM004-P	IM004_Mock_7d_2_proteomics	Protein	PeptideAtlas
IM004-P	IM004_Mock_7d_3_proteomics	Protein	PeptideAtlas
IM005-P	IM005_Mock_1d_1_proteomics	Protein	PeptideAtlas
IM005-P	IM005_Mock_1d_2_proteomics	Protein	PeptideAtlas
IM005-P	IM005_Mock_1d_3_proteomics	Protein	PeptideAtlas
IM005-P	IM005_Mock_2d_1_proteomics	Protein	PeptideAtlas
IM005-P	IM005_Mock_2d_2_proteomics	Protein	PeptideAtlas
IM005-P	IM005_Mock_2d_3_proteomics	Protein	PeptideAtlas
IM005-P	IM005_Mock_4d_1_proteomics	Protein	PeptideAtlas
IM005-P	IM005_Mock_4d_2_proteomics	Protein	PeptideAtlas
IM005-P	IM005_Mock_4d_3_proteomics	Protein	PeptideAtlas
IM005-P	IM005_Mock_7d_1_proteomics	Protein	PeptideAtlas
IM005-P	IM005_Mock_7d_2_proteomics	Protein	PeptideAtlas
IM005-P	IM005_Mock_7d_3_proteomics	Protein	PeptideAtlas
IM005-P	IM005_PB2_627E_10^4pfu_1d_1_proteomics	Protein	PeptideAtlas
IM005-P	IM005_PB2_627E_10^4pfu_1d_2_proteomics	Protein	PeptideAtlas
IM005-P	IM005_PB2_627E_10^4pfu_1d_3_proteomics	Protein	PeptideAtlas
IM005-P	IM005_PB2_627E_10^4pfu_1d_4_proteomics	Protein	PeptideAtlas
IM005-P	IM005_PB2_627E_10^4pfu_1d_5_proteomics	Protein	PeptideAtlas
IM005-P	IM005_PB2_627E_10^4pfu_2d_1_proteomics	Protein	PeptideAtlas
IM005-P	IM005_PB2_627E_10^4pfu_2d_2_proteomics	Protein	PeptideAtlas
IM005-P	IM005_PB2_627E_10^4pfu_2d_3_proteomics	Protein	PeptideAtlas
IM005-P	IM005_PB2_627E_10^4pfu_2d_4_proteomics	Protein	PeptideAtlas
IM005-P	IM005_PB2_627E_10^4pfu_2d_5_proteomics	Protein	PeptideAtlas
IM005-P	IM005_PB2_627E_10^4pfu_4d_1_proteomics	Protein	PeptideAtlas
IM005-P	IM005_PB2_627E_10^4pfu_4d_2_proteomics	Protein	PeptideAtlas
IM005-P	IM005_PB2_627E_10^4pfu_4d_3_proteomics	Protein	PeptideAtlas
IM005-P	IM005_PB2_627E_10^4pfu_4d_4_proteomics	Protein	PeptideAtlas
IM005-P	IM005_PB2_627E_10^4pfu_4d_5_proteomics	Protein	PeptideAtlas
IM005-P	IM005_PB2_627E_10^4pfu_7d_1_proteomics	Protein	PeptideAtlas
IM005-P	IM005_PB2_627E_10^4pfu_7d_2_proteomics	Protein	PeptideAtlas
IM005-P	IM005_PB2_627E_10^4pfu_7d_3_proteomics	Protein	PeptideAtlas
IM005-P	IM005_PB2_627E_10^4pfu_7d_4_proteomics	Protein	PeptideAtlas
IM005-P	IM005_PB2_627E_10^4pfu_7d_5_proteomics	Protein	PeptideAtlas
IM006A-P	IM006A_Mock_1d_1_proteomics	Protein	PeptideAtlas
IM006A-P	IM006A_Mock_1d_2_proteomics	Protein	PeptideAtlas
IM006A-P	IM006A_Mock_1d_3_proteomics	Protein	PeptideAtlas
IM006A-P	IM006A_Mock_2d_1_proteomics	Protein	PeptideAtlas
IM006A-P	IM006A_Mock_2d_2_proteomics	Protein	PeptideAtlas
IM006A-P	IM006A_Mock_2d_3_proteomics	Protein	PeptideAtlas
IM006A-P	IM006A_Mock_4d_1_proteomics	Protein	PeptideAtlas
IM006A-P	IM006A_Mock_4d_2_proteomics	Protein	PeptideAtlas
IM006A-P	IM006A_Mock_4d_3_proteomics	Protein	PeptideAtlas
IM006A-P	IM006A_Mock_7d_1_proteomics	Protein	PeptideAtlas
IM006A-P	IM006A_Mock_7d_2_proteomics	Protein	PeptideAtlas
IM006A-P	IM006A_Mock_7d_3_proteomics	Protein	PeptideAtlas
IM006A-P	IM006A_PB1_F2del_10^3pfu_1d_1_proteomics	Protein	PeptideAtlas
IM006A-P	IM006A_PB1_F2del_10^3pfu_1d_2_proteomics	Protein	PeptideAtlas
IM006A-P	IM006A_PB1_F2del_10^3pfu_1d_3_proteomics	Protein	PeptideAtlas
IM006A-P	IM006A_PB1_F2del_10^3pfu_1d_4_proteomics	Protein	PeptideAtlas
IM006A-P	IM006A_PB1_F2del_10^3pfu_1d_5_proteomics	Protein	PeptideAtlas
IM006A-P	IM006A_PB1_F2del_10^3pfu_2d_1_proteomics	Protein	PeptideAtlas
IM006A-P	IM006A_PB1_F2del_10^3pfu_2d_2_proteomics	Protein	PeptideAtlas
IM006A-P	IM006A_PB1_F2del_10^3pfu_2d_3_proteomics	Protein	PeptideAtlas
IM006A-P	IM006A_PB1_F2del_10^3pfu_2d_4_proteomics	Protein	PeptideAtlas
IM006A-P	IM006A_PB1_F2del_10^3pfu_2d_5_proteomics	Protein	PeptideAtlas
IM006A-P	IM006A_PB1_F2del_10^3pfu_4d_1_proteomics	Protein	PeptideAtlas
IM006A-P	IM006A_PB1_F2del_10^3pfu_4d_2_proteomics	Protein	PeptideAtlas
IM006A-P	IM006A_PB1_F2del_10^3pfu_4d_3_proteomics	Protein	PeptideAtlas
IM006A-P	IM006A_PB1_F2del_10^3pfu_4d_4_proteomics	Protein	PeptideAtlas
IM006A-P	IM006A_PB1_F2del_10^3pfu_4d_5_proteomics	Protein	PeptideAtlas
IM006A-P	IM006A_PB1_F2del_10^3pfu_7d_1_proteomics	Protein	PeptideAtlas
IM006A-P	IM006A_PB1_F2del_10^3pfu_7d_2_proteomics	Protein	PeptideAtlas
IM006A-P	IM006A_PB1_F2del_10^3pfu_7d_3_proteomics	Protein	PeptideAtlas
IM006A-P	IM006A_PB1_F2del_10^3pfu_7d_4_proteomics	Protein	PeptideAtlas
IM006A-P	IM006A_PB1_F2del_10^3pfu_7d_5_proteomics	Protein	PeptideAtlas
IM006B-P	IM006B_Mock_1d_1_proteomics	Protein	PeptideAtlas
IM006B-P	IM006B_Mock_1d_2_proteomics	Protein	PeptideAtlas
IM006B-P	IM006B_Mock_1d_3_proteomics	Protein	PeptideAtlas
IM006B-P	IM006B_Mock_2d_1_proteomics	Protein	PeptideAtlas
IM006B-P	IM006B_Mock_2d_2_proteomics	Protein	PeptideAtlas
IM006B-P	IM006B_Mock_2d_3_proteomics	Protein	PeptideAtlas
IM006B-P	IM006B_Mock_4d_1_proteomics	Protein	PeptideAtlas
IM006B-P	IM006B_Mock_4d_2_proteomics	Protein	PeptideAtlas
IM006B-P	IM006B_Mock_4d_3_proteomics	Protein	PeptideAtlas
IM006B-P	IM006B_PB1_F2del_10^4pfu_1d_1_proteomics	Protein	PeptideAtlas
IM006B-P	IM006B_PB1_F2del_10^4pfu_1d_2_proteomics	Protein	PeptideAtlas
IM006B-P	IM006B_PB1_F2del_10^4pfu_1d_3_proteomics	Protein	PeptideAtlas
IM006B-P	IM006B_PB1_F2del_10^4pfu_1d_4_proteomics	Protein	PeptideAtlas
IM006B-P	IM006B_PB1_F2del_10^4pfu_1d_5_proteomics	Protein	PeptideAtlas
IM006B-P	IM006B_PB1_F2del_10^4pfu_2d_1_proteomics	Protein	PeptideAtlas
IM006B-P	IM006B_PB1_F2del_10^4pfu_2d_2_proteomics	Protein	PeptideAtlas
IM006B-P	IM006B_PB1_F2del_10^4pfu_2d_3_proteomics	Protein	PeptideAtlas
IM006B-P	IM006B_PB1_F2del_10^4pfu_2d_4_proteomics	Protein	PeptideAtlas
IM006B-P	IM006B_PB1_F2del_10^4pfu_2d_5_proteomics	Protein	PeptideAtlas
IM006B-P	IM006B_PB1_F2del_10^4pfu_4d_1_proteomics	Protein	PeptideAtlas
IM006B-P	IM006B_PB1_F2del_10^4pfu_4d_2_proteomics	Protein	PeptideAtlas
IM006B-P	IM006B_PB1_F2del_10^4pfu_4d_3_proteomics	Protein	PeptideAtlas
IM006B-P	IM006B_PB1_F2del_10^4pfu_4d_4_proteomics	Protein	PeptideAtlas
IM006B-P	IM006B_PB1_F2del_10^4pfu_4d_5_proteomics	Protein	PeptideAtlas
IM007-P	IM007_Mock_1d_1_proteomics	Protein	PeptideAtlas
IM007-P	IM007_Mock_1d_2_proteomics	Protein	PeptideAtlas
IM007-P	IM007_Mock_1d_3_proteomics	Protein	PeptideAtlas
IM007-P	IM007_Mock_2d_1_proteomics	Protein	PeptideAtlas
IM007-P	IM007_Mock_2d_2_proteomics	Protein	PeptideAtlas
IM007-P	IM007_Mock_2d_3_proteomics	Protein	PeptideAtlas
IM007-P	IM007_Mock_4d_1_proteomics	Protein	PeptideAtlas
IM007-P	IM007_Mock_4d_2_proteomics	Protein	PeptideAtlas
IM007-P	IM007_Mock_4d_3_proteomics	Protein	PeptideAtlas
IM007-P	IM007_Mock_7d_1_proteomics	Protein	PeptideAtlas
IM007-P	IM007_Mock_7d_2_proteomics	Protein	PeptideAtlas
IM007-P	IM007_Mock_7d_3_proteomics	Protein	PeptideAtlas
IM007-P	IM007_NS1trunc124_10^3pfu_1d_1_proteomics	Protein	PeptideAtlas
IM007-P	IM007_NS1trunc124_10^3pfu_1d_2_proteomics	Protein	PeptideAtlas
IM007-P	IM007_NS1trunc124_10^3pfu_1d_3_proteomics	Protein	PeptideAtlas
IM007-P	IM007_NS1trunc124_10^3pfu_1d_4_proteomics	Protein	PeptideAtlas
IM007-P	IM007_NS1trunc124_10^3pfu_1d_5_proteomics	Protein	PeptideAtlas
IM007-P	IM007_NS1trunc124_10^3pfu_2d_1_proteomics	Protein	PeptideAtlas
IM007-P	IM007_NS1trunc124_10^3pfu_2d_2_proteomics	Protein	PeptideAtlas
IM007-P	IM007_NS1trunc124_10^3pfu_2d_3_proteomics	Protein	PeptideAtlas
IM007-P	IM007_NS1trunc124_10^3pfu_2d_4_proteomics	Protein	PeptideAtlas
IM007-P	IM007_NS1trunc124_10^3pfu_2d_5_proteomics	Protein	PeptideAtlas
IM007-P	IM007_NS1trunc124_10^3pfu_4d_1_proteomics	Protein	PeptideAtlas
IM007-P	IM007_NS1trunc124_10^3pfu_4d_2_proteomics	Protein	PeptideAtlas
IM007-P	IM007_NS1trunc124_10^3pfu_4d_3_proteomics	Protein	PeptideAtlas
IM007-P	IM007_NS1trunc124_10^3pfu_4d_4_proteomics	Protein	PeptideAtlas
IM007-P	IM007_NS1trunc124_10^3pfu_4d_5_proteomics	Protein	PeptideAtlas
IM007-P	IM007_NS1trunc124_10^3pfu_7d_1_proteomics	Protein	PeptideAtlas
IM007-P	IM007_NS1trunc124_10^3pfu_7d_2_proteomics	Protein	PeptideAtlas
IM007-P	IM007_NS1trunc124_10^3pfu_7d_3_proteomics	Protein	PeptideAtlas
IM007-P	IM007_NS1trunc124_10^3pfu_7d_4_proteomics	Protein	PeptideAtlas
IM007-P	IM007_NS1trunc124_10^3pfu_7d_5_proteomics	Protein	PeptideAtlas
IM007-P	IM007_NS1trunc124_10^4pfu_1d_1_proteomics	Protein	PeptideAtlas
IM007-P	IM007_NS1trunc124_10^4pfu_1d_2_proteomics	Protein	PeptideAtlas
IM007-P	IM007_NS1trunc124_10^4pfu_1d_3_proteomics	Protein	PeptideAtlas
IM007-P	IM007_NS1trunc124_10^4pfu_1d_4_proteomics	Protein	PeptideAtlas
IM007-P	IM007_NS1trunc124_10^4pfu_1d_5_proteomics	Protein	PeptideAtlas
IM007-P	IM007_NS1trunc124_10^4pfu_2d_1_proteomics	Protein	PeptideAtlas
IM007-P	IM007_NS1trunc124_10^4pfu_2d_2_proteomics	Protein	PeptideAtlas
IM007-P	IM007_NS1trunc124_10^4pfu_2d_3_proteomics	Protein	PeptideAtlas
IM007-P	IM007_NS1trunc124_10^4pfu_2d_4_proteomics	Protein	PeptideAtlas
IM007-P	IM007_NS1trunc124_10^4pfu_2d_5_proteomics	Protein	PeptideAtlas
IM007-P	IM007_NS1trunc124_10^4pfu_4d_1_proteomics	Protein	PeptideAtlas
IM007-P	IM007_NS1trunc124_10^4pfu_4d_2_proteomics	Protein	PeptideAtlas
IM007-P	IM007_NS1trunc124_10^4pfu_4d_3_proteomics	Protein	PeptideAtlas
IM007-P	IM007_NS1trunc124_10^4pfu_4d_4_proteomics	Protein	PeptideAtlas
IM007-P	IM007_NS1trunc124_10^4pfu_4d_5_proteomics	Protein	PeptideAtlas
IM007-P	IM007_NS1trunc124_10^4pfu_7d_1_proteomics	Protein	PeptideAtlas
IM007-P	IM007_NS1trunc124_10^4pfu_7d_2_proteomics	Protein	PeptideAtlas
IM007-P	IM007_NS1trunc124_10^4pfu_7d_3_proteomics	Protein	PeptideAtlas
IM007-P	IM007_NS1trunc124_10^4pfu_7d_4_proteomics	Protein	PeptideAtlas
IM007-P	IM007_NS1trunc124_10^4pfu_7d_5_proteomics	Protein	PeptideAtlas
SCL005-P	SCL005_dORF6_0h_1_proteomics	Protein	PeptideAtlas
SCL005-P	SCL005_dORF6_0h_2_proteomics	Protein	PeptideAtlas
SCL005-P	SCL005_dORF6_0h_3_proteomics	Protein	PeptideAtlas
SCL005-P	SCL005_dORF6_12h_1_proteomics	Protein	PeptideAtlas
SCL005-P	SCL005_dORF6_12h_2_proteomics	Protein	PeptideAtlas
SCL005-P	SCL005_dORF6_12h_3_proteomics	Protein	PeptideAtlas
SCL005-P	SCL005_dORF6_24h_1_proteomics	Protein	PeptideAtlas
SCL005-P	SCL005_dORF6_24h_2_proteomics	Protein	PeptideAtlas
SCL005-P	SCL005_dORF6_24h_3_proteomics	Protein	PeptideAtlas
SCL005-P	SCL005_dORF6_30h_1_proteomics	Protein	PeptideAtlas
SCL005-P	SCL005_dORF6_30h_2_proteomics	Protein	PeptideAtlas
SCL005-P	SCL005_dORF6_30h_3_proteomics	Protein	PeptideAtlas
SCL005-P	SCL005_dORF6_36h_1_proteomics	Protein	PeptideAtlas
SCL005-P	SCL005_dORF6_36h_2_proteomics	Protein	PeptideAtlas
SCL005-P	SCL005_dORF6_36h_3_proteomics	Protein	PeptideAtlas
SCL005-P	SCL005_dORF6_3h_1_proteomics	Protein	PeptideAtlas
SCL005-P	SCL005_dORF6_3h_2_proteomics	Protein	PeptideAtlas
SCL005-P	SCL005_dORF6_3h_3_proteomics	Protein	PeptideAtlas
SCL005-P	SCL005_dORF6_48h_1_proteomics	Protein	PeptideAtlas
SCL005-P	SCL005_dORF6_48h_2_proteomics	Protein	PeptideAtlas
SCL005-P	SCL005_dORF6_48h_3_proteomics	Protein	PeptideAtlas
SCL005-P	SCL005_dORF6_54h_1_proteomics	Protein	PeptideAtlas
SCL005-P	SCL005_dORF6_54h_2_proteomics	Protein	PeptideAtlas
SCL005-P	SCL005_dORF6_60h_1_proteomics	Protein	PeptideAtlas
SCL005-P	SCL005_dORF6_60h_2_proteomics	Protein	PeptideAtlas
SCL005-P	SCL005_dORF6_60h_3_proteomics	Protein	PeptideAtlas
SCL005-P	SCL005_dORF6_72h_1_proteomics	Protein	PeptideAtlas
SCL005-P	SCL005_dORF6_72h_2_proteomics	Protein	PeptideAtlas
SCL005-P	SCL005_dORF6_72h_3_proteomics	Protein	PeptideAtlas
SCL005-P	SCL005_dORF6_7h_1_proteomics	Protein	PeptideAtlas
SCL005-P	SCL005_dORF6_7h_2_proteomics	Protein	PeptideAtlas
SCL005-P	SCL005_dORF6_7h_3_proteomics	Protein	PeptideAtlas
SCL005-P	SCL005_icSARS_0h_1_proteomics	Protein	PeptideAtlas
SCL005-P	SCL005_icSARS_0h_2_proteomics	Protein	PeptideAtlas
SCL005-P	SCL005_icSARS_0h_3_proteomics	Protein	PeptideAtlas
SCL005-P	SCL005_icSARS_12h_1_proteomics	Protein	PeptideAtlas
SCL005-P	SCL005_icSARS_12h_2_proteomics	Protein	PeptideAtlas
SCL005-P	SCL005_icSARS_24h_1_proteomics	Protein	PeptideAtlas
SCL005-P	SCL005_icSARS_24h_2_proteomics	Protein	PeptideAtlas
SCL005-P	SCL005_icSARS_24h_3_proteomics	Protein	PeptideAtlas
SCL005-P	SCL005_icSARS_30h_1_proteomics	Protein	PeptideAtlas
SCL005-P	SCL005_icSARS_30h_2_proteomics	Protein	PeptideAtlas
SCL005-P	SCL005_icSARS_30h_3_proteomics	Protein	PeptideAtlas
SCL005-P	SCL005_icSARS_36h_1_proteomics	Protein	PeptideAtlas
SCL005-P	SCL005_icSARS_36h_2_proteomics	Protein	PeptideAtlas
SCL005-P	SCL005_icSARS_36h_3_proteomics	Protein	PeptideAtlas
SCL005-P	SCL005_icSARS_3h_1_proteomics	Protein	PeptideAtlas
SCL005-P	SCL005_icSARS_3h_2_proteomics	Protein	PeptideAtlas
SCL005-P	SCL005_icSARS_3h_3_proteomics	Protein	PeptideAtlas
SCL005-P	SCL005_icSARS_48h_1_proteomics	Protein	PeptideAtlas
SCL005-P	SCL005_icSARS_48h_2_proteomics	Protein	PeptideAtlas
SCL005-P	SCL005_icSARS_48h_3_proteomics	Protein	PeptideAtlas
SCL005-P	SCL005_icSARS_54h_1_proteomics	Protein	PeptideAtlas
SCL005-P	SCL005_icSARS_54h_2_proteomics	Protein	PeptideAtlas
SCL005-P	SCL005_icSARS_54h_3_proteomics	Protein	PeptideAtlas
SCL005-P	SCL005_icSARS_60h_1_proteomics	Protein	PeptideAtlas
SCL005-P	SCL005_icSARS_60h_2_proteomics	Protein	PeptideAtlas
SCL005-P	SCL005_icSARS_60h_3_proteomics	Protein	PeptideAtlas
SCL005-P	SCL005_icSARS_72h_1_proteomics	Protein	PeptideAtlas
SCL005-P	SCL005_icSARS_72h_2_proteomics	Protein	PeptideAtlas
SCL005-P	SCL005_icSARS_72h_3_proteomics	Protein	PeptideAtlas
SCL005-P	SCL005_icSARS_7h_1_proteomics	Protein	PeptideAtlas
SCL005-P	SCL005_icSARS_7h_2_proteomics	Protein	PeptideAtlas
SCL005-P	SCL005_Mock_0h_1_proteomics	Protein	PeptideAtlas
SCL005-P	SCL005_Mock_0h_2_proteomics	Protein	PeptideAtlas
SCL005-P	SCL005_Mock_0h_3_proteomics	Protein	PeptideAtlas
SCL005-P	SCL005_Mock_12h_1_proteomics	Protein	PeptideAtlas
SCL005-P	SCL005_Mock_12h_2_proteomics	Protein	PeptideAtlas
SCL005-P	SCL005_Mock_12h_3_proteomics	Protein	PeptideAtlas
SCL005-P	SCL005_Mock_24h_1_proteomics	Protein	PeptideAtlas
SCL005-P	SCL005_Mock_24h_2_proteomics	Protein	PeptideAtlas
SCL005-P	SCL005_Mock_24h_3_proteomics	Protein	PeptideAtlas
SCL005-P	SCL005_Mock_30h_1_proteomics	Protein	PeptideAtlas
SCL005-P	SCL005_Mock_30h_2_proteomics	Protein	PeptideAtlas
SCL005-P	SCL005_Mock_30h_3_proteomics	Protein	PeptideAtlas
SCL005-P	SCL005_Mock_36h_1_proteomics	Protein	PeptideAtlas
SCL005-P	SCL005_Mock_36h_2_proteomics	Protein	PeptideAtlas
SCL005-P	SCL005_Mock_36h_3_proteomics	Protein	PeptideAtlas
SCL005-P	SCL005_Mock_3h_1_proteomics	Protein	PeptideAtlas
SCL005-P	SCL005_Mock_3h_2_proteomics	Protein	PeptideAtlas
SCL005-P	SCL005_Mock_3h_3_proteomics	Protein	PeptideAtlas
SCL005-P	SCL005_Mock_48h_1_proteomics	Protein	PeptideAtlas
SCL005-P	SCL005_Mock_48h_2_proteomics	Protein	PeptideAtlas
SCL005-P	SCL005_Mock_48h_3_proteomics	Protein	PeptideAtlas
SCL005-P	SCL005_Mock_54h_1_proteomics	Protein	PeptideAtlas
SCL005-P	SCL005_Mock_54h_2_proteomics	Protein	PeptideAtlas
SCL005-P	SCL005_Mock_54h_3_proteomics	Protein	PeptideAtlas
SCL005-P	SCL005_Mock_60h_1_proteomics	Protein	PeptideAtlas
SCL005-P	SCL005_Mock_60h_2_proteomics	Protein	PeptideAtlas
SCL005-P	SCL005_Mock_60h_3_proteomics	Protein	PeptideAtlas
SCL005-P	SCL005_Mock_72h_1_proteomics	Protein	PeptideAtlas
SCL005-P	SCL005_Mock_72h_2_proteomics	Protein	PeptideAtlas
SCL005-P	SCL005_Mock_72h_3_proteomics	Protein	PeptideAtlas
SCL005-P	SCL005_Mock_7h_1_proteomics	Protein	PeptideAtlas
SCL005-P	SCL005_Mock_7h_2_proteomics	Protein	PeptideAtlas
SCL006-P	SCL006_BatSRBD_0h_1_proteomics	Protein	PeptideAtlas
SCL006-P	SCL006_BatSRBD_0h_2_proteomics	Protein	PeptideAtlas
SCL006-P	SCL006_BatSRBD_0h_3_proteomics	Protein	PeptideAtlas
SCL006-P	SCL006_BatSRBD_12h_1_proteomics	Protein	PeptideAtlas
SCL006-P	SCL006_BatSRBD_12h_2_proteomics	Protein	PeptideAtlas
SCL006-P	SCL006_BatSRBD_12h_3_proteomics	Protein	PeptideAtlas
SCL006-P	SCL006_BatSRBD_24h_1_proteomics	Protein	PeptideAtlas
SCL006-P	SCL006_BatSRBD_24h_2_proteomics	Protein	PeptideAtlas
SCL006-P	SCL006_BatSRBD_24h_3_proteomics	Protein	PeptideAtlas
SCL006-P	SCL006_BatSRBD_30h_2_proteomics	Protein	PeptideAtlas
SCL006-P	SCL006_BatSRBD_30h_3_proteomics	Protein	PeptideAtlas
SCL006-P	SCL006_BatSRBD_36h_1_proteomics	Protein	PeptideAtlas
SCL006-P	SCL006_BatSRBD_36h_2_proteomics	Protein	PeptideAtlas
SCL006-P	SCL006_BatSRBD_36h_3_proteomics	Protein	PeptideAtlas
SCL006-P	SCL006_BatSRBD_3h_1_proteomics	Protein	PeptideAtlas
SCL006-P	SCL006_BatSRBD_3h_2_proteomics	Protein	PeptideAtlas
SCL006-P	SCL006_BatSRBD_3h_3_proteomics	Protein	PeptideAtlas
SCL006-P	SCL006_BatSRBD_48h_2_proteomics	Protein	PeptideAtlas
SCL006-P	SCL006_BatSRBD_48h_3_proteomics	Protein	PeptideAtlas
SCL006-P	SCL006_BatSRBD_54h_1_proteomics	Protein	PeptideAtlas
SCL006-P	SCL006_BatSRBD_54h_2_proteomics	Protein	PeptideAtlas
SCL006-P	SCL006_BatSRBD_60h_1_proteomics	Protein	PeptideAtlas
SCL006-P	SCL006_BatSRBD_60h_2_proteomics	Protein	PeptideAtlas
SCL006-P	SCL006_BatSRBD_60h_3_proteomics	Protein	PeptideAtlas
SCL006-P	SCL006_BatSRBD_72h_1_proteomics	Protein	PeptideAtlas
SCL006-P	SCL006_BatSRBD_72h_2_proteomics	Protein	PeptideAtlas
SCL006-P	SCL006_BatSRBD_72h_3_proteomics	Protein	PeptideAtlas
SCL006-P	SCL006_BatSRBD_7h_1_proteomics	Protein	PeptideAtlas
SCL006-P	SCL006_BatSRBD_7h_2_proteomics	Protein	PeptideAtlas
SCL006-P	SCL006_BatSRBD_7h_3_proteomics	Protein	PeptideAtlas
SCL006-P	SCL006_icSARS_0h_1_proteomics	Protein	PeptideAtlas
SCL006-P	SCL006_icSARS_0h_2_proteomics	Protein	PeptideAtlas
SCL006-P	SCL006_icSARS_0h_3_proteomics	Protein	PeptideAtlas
SCL006-P	SCL006_icSARS_12h_1_proteomics	Protein	PeptideAtlas
SCL006-P	SCL006_icSARS_12h_2_proteomics	Protein	PeptideAtlas
SCL006-P	SCL006_icSARS_12h_3_proteomics	Protein	PeptideAtlas
SCL006-P	SCL006_icSARS_24h_1_proteomics	Protein	PeptideAtlas
SCL006-P	SCL006_icSARS_24h_2_proteomics	Protein	PeptideAtlas
SCL006-P	SCL006_icSARS_24h_3_proteomics	Protein	PeptideAtlas
SCL006-P	SCL006_icSARS_30h_2_proteomics	Protein	PeptideAtlas
SCL006-P	SCL006_icSARS_30h_3_proteomics	Protein	PeptideAtlas
SCL006-P	SCL006_icSARS_36h_1_proteomics	Protein	PeptideAtlas
SCL006-P	SCL006_icSARS_36h_2_proteomics	Protein	PeptideAtlas
SCL006-P	SCL006_icSARS_36h_3_proteomics	Protein	PeptideAtlas
SCL006-P	SCL006_icSARS_48h_1_proteomics	Protein	PeptideAtlas
SCL006-P	SCL006_icSARS_48h_2_proteomics	Protein	PeptideAtlas
SCL006-P	SCL006_icSARS_48h_3_proteomics	Protein	PeptideAtlas
SCL006-P	SCL006_icSARS_54h_1_proteomics	Protein	PeptideAtlas
SCL006-P	SCL006_icSARS_54h_2_proteomics	Protein	PeptideAtlas
SCL006-P	SCL006_icSARS_54h_3_proteomics	Protein	PeptideAtlas
SCL006-P	SCL006_icSARS_60h_1_proteomics	Protein	PeptideAtlas
SCL006-P	SCL006_icSARS_60h_2_proteomics	Protein	PeptideAtlas
SCL006-P	SCL006_icSARS_60h_3_proteomics	Protein	PeptideAtlas
SCL006-P	SCL006_icSARS_72h_1_proteomics	Protein	PeptideAtlas
SCL006-P	SCL006_icSARS_72h_2_proteomics	Protein	PeptideAtlas
SCL006-P	SCL006_icSARS_72h_3_proteomics	Protein	PeptideAtlas
SCL006-P	SCL006_icSARS_7h_1_proteomics	Protein	PeptideAtlas
SCL006-P	SCL006_icSARS_7h_2_proteomics	Protein	PeptideAtlas
SCL006-P	SCL006_icSARS_7h_3_proteomics	Protein	PeptideAtlas
SCL006-P	SCL006_Mock_0h_2_proteomics	Protein	PeptideAtlas
SCL006-P	SCL006_Mock_0h_3_proteomics	Protein	PeptideAtlas
SCL006-P	SCL006_Mock_12h_1_proteomics	Protein	PeptideAtlas
SCL006-P	SCL006_Mock_12h_2_proteomics	Protein	PeptideAtlas
SCL006-P	SCL006_Mock_12h_3_proteomics	Protein	PeptideAtlas
SCL006-P	SCL006_Mock_24h_1_proteomics	Protein	PeptideAtlas
SCL006-P	SCL006_Mock_24h_2_proteomics	Protein	PeptideAtlas
SCL006-P	SCL006_Mock_24h_3_proteomics	Protein	PeptideAtlas
SCL006-P	SCL006_Mock_30h_1_proteomics	Protein	PeptideAtlas
SCL006-P	SCL006_Mock_30h_2_proteomics	Protein	PeptideAtlas
SCL006-P	SCL006_Mock_30h_3_proteomics	Protein	PeptideAtlas
SCL006-P	SCL006_Mock_36h_1_proteomics	Protein	PeptideAtlas
SCL006-P	SCL006_Mock_36h_2_proteomics	Protein	PeptideAtlas
SCL006-P	SCL006_Mock_36h_3_proteomics	Protein	PeptideAtlas
SCL006-P	SCL006_Mock_3h_1_proteomics	Protein	PeptideAtlas
SCL006-P	SCL006_Mock_3h_2_proteomics	Protein	PeptideAtlas
SCL006-P	SCL006_Mock_3h_3_proteomics	Protein	PeptideAtlas
SCL006-P	SCL006_Mock_48h_1_proteomics	Protein	PeptideAtlas
SCL006-P	SCL006_Mock_48h_2_proteomics	Protein	PeptideAtlas
SCL006-P	SCL006_Mock_48h_3_proteomics	Protein	PeptideAtlas
SCL006-P	SCL006_Mock_54h_1_proteomics	Protein	PeptideAtlas
SCL006-P	SCL006_Mock_54h_2_proteomics	Protein	PeptideAtlas
SCL006-P	SCL006_Mock_54h_3_proteomics	Protein	PeptideAtlas
SCL006-P	SCL006_Mock_60h_1_proteomics	Protein	PeptideAtlas
SCL006-P	SCL006_Mock_60h_2_proteomics	Protein	PeptideAtlas
SCL006-P	SCL006_Mock_72h_1_proteomics	Protein	PeptideAtlas
SCL006-P	SCL006_Mock_72h_3_proteomics	Protein	PeptideAtlas
SCL006-P	SCL006_Mock_7h_1_proteomics	Protein	PeptideAtlas
SCL006-P	SCL006_Mock_7h_2_proteomics	Protein	PeptideAtlas
SCL006-P	SCL006_Mock_7h_3_proteomics	Protein	PeptideAtlas
SM001-P	SM001_Mock_1d_1_proteomics	Protein	PeptideAtlas
SM001-P	SM001_Mock_1d_2_proteomics	Protein	PeptideAtlas
SM001-P	SM001_Mock_1d_3_proteomics	Protein	PeptideAtlas
SM001-P	SM001_Mock_2d_1_proteomics	Protein	PeptideAtlas
SM001-P	SM001_Mock_2d_2_proteomics	Protein	PeptideAtlas
SM001-P	SM001_Mock_2d_3_proteomics	Protein	PeptideAtlas
SM001-P	SM001_Mock_4d_1_proteomics	Protein	PeptideAtlas
SM001-P	SM001_Mock_4d_2_proteomics	Protein	PeptideAtlas
SM001-P	SM001_Mock_4d_3_proteomics	Protein	PeptideAtlas
SM001-P	SM001_Mock_7d_1_proteomics	Protein	PeptideAtlas
SM001-P	SM001_Mock_7d_2_proteomics	Protein	PeptideAtlas
SM001-P	SM001_Mock_7d_3_proteomics	Protein	PeptideAtlas
SM001-P	SM001_SARS_10^2_1d_1_proteomics	Protein	PeptideAtlas
SM001-P	SM001_SARS_10^2_1d_2_proteomics	Protein	PeptideAtlas
SM001-P	SM001_SARS_10^2_1d_3_proteomics	Protein	PeptideAtlas
SM001-P	SM001_SARS_10^2_1d_4_proteomics	Protein	PeptideAtlas
SM001-P	SM001_SARS_10^2_1d_5_proteomics	Protein	PeptideAtlas
SM001-P	SM001_SARS_10^2_2d_1_proteomics	Protein	PeptideAtlas
SM001-P	SM001_SARS_10^2_2d_2_proteomics	Protein	PeptideAtlas
SM001-P	SM001_SARS_10^2_2d_3_proteomics	Protein	PeptideAtlas
SM001-P	SM001_SARS_10^2_2d_4_proteomics	Protein	PeptideAtlas
SM001-P	SM001_SARS_10^2_2d_5_proteomics	Protein	PeptideAtlas
SM001-P	SM001_SARS_10^2_4d_1_proteomics	Protein	PeptideAtlas
SM001-P	SM001_SARS_10^2_4d_2_proteomics	Protein	PeptideAtlas
SM001-P	SM001_SARS_10^2_4d_3_proteomics	Protein	PeptideAtlas
SM001-P	SM001_SARS_10^2_4d_4_proteomics	Protein	PeptideAtlas
SM001-P	SM001_SARS_10^2_4d_5_proteomics	Protein	PeptideAtlas
SM001-P	SM001_SARS_10^2_7d_1_proteomics	Protein	PeptideAtlas
SM001-P	SM001_SARS_10^2_7d_2_proteomics	Protein	PeptideAtlas
SM001-P	SM001_SARS_10^2_7d_3_proteomics	Protein	PeptideAtlas
SM001-P	SM001_SARS_10^2_7d_4_proteomics	Protein	PeptideAtlas
SM001-P	SM001_SARS_10^2_7d_5_proteomics	Protein	PeptideAtlas
SM001-P	SM001_SARS_10^3_1d_1_proteomics	Protein	PeptideAtlas
SM001-P	SM001_SARS_10^3_1d_2_proteomics	Protein	PeptideAtlas
SM001-P	SM001_SARS_10^3_1d_3_proteomics	Protein	PeptideAtlas
SM001-P	SM001_SARS_10^3_1d_4_proteomics	Protein	PeptideAtlas
SM001-P	SM001_SARS_10^3_1d_5_proteomics	Protein	PeptideAtlas
SM001-P	SM001_SARS_10^3_2d_1_proteomics	Protein	PeptideAtlas
SM001-P	SM001_SARS_10^3_2d_2_proteomics	Protein	PeptideAtlas
SM001-P	SM001_SARS_10^3_2d_3_proteomics	Protein	PeptideAtlas
SM001-P	SM001_SARS_10^3_2d_4_proteomics	Protein	PeptideAtlas
SM001-P	SM001_SARS_10^3_2d_5_proteomics	Protein	PeptideAtlas
SM001-P	SM001_SARS_10^3_4d_1_proteomics	Protein	PeptideAtlas
SM001-P	SM001_SARS_10^3_4d_2_proteomics	Protein	PeptideAtlas
SM001-P	SM001_SARS_10^3_4d_3_proteomics	Protein	PeptideAtlas
SM001-P	SM001_SARS_10^3_4d_4_proteomics	Protein	PeptideAtlas
SM001-P	SM001_SARS_10^3_4d_5_proteomics	Protein	PeptideAtlas
SM001-P	SM001_SARS_10^3_7d_1_proteomics	Protein	PeptideAtlas
SM001-P	SM001_SARS_10^3_7d_2_proteomics	Protein	PeptideAtlas
SM001-P	SM001_SARS_10^3_7d_3_proteomics	Protein	PeptideAtlas
SM001-P	SM001_SARS_10^3_7d_4_proteomics	Protein	PeptideAtlas
SM001-P	SM001_SARS_10^3_7d_5_proteomics	Protein	PeptideAtlas
SM001-P	SM001_SARS_10^4_1d_1_proteomics	Protein	PeptideAtlas
SM001-P	SM001_SARS_10^4_1d_2_proteomics	Protein	PeptideAtlas
SM001-P	SM001_SARS_10^4_1d_3_proteomics	Protein	PeptideAtlas
SM001-P	SM001_SARS_10^4_1d_4_proteomics	Protein	PeptideAtlas
SM001-P	SM001_SARS_10^4_1d_5_proteomics	Protein	PeptideAtlas
SM001-P	SM001_SARS_10^4_2d_1_proteomics	Protein	PeptideAtlas
SM001-P	SM001_SARS_10^4_2d_2_proteomics	Protein	PeptideAtlas
SM001-P	SM001_SARS_10^4_2d_3_proteomics	Protein	PeptideAtlas
SM001-P	SM001_SARS_10^4_2d_4_proteomics	Protein	PeptideAtlas
SM001-P	SM001_SARS_10^4_2d_5_proteomics	Protein	PeptideAtlas
SM001-P	SM001_SARS_10^4_4d_1_proteomics	Protein	PeptideAtlas
SM001-P	SM001_SARS_10^4_4d_2_proteomics	Protein	PeptideAtlas
SM001-P	SM001_SARS_10^4_4d_3_proteomics	Protein	PeptideAtlas
SM001-P	SM001_SARS_10^4_4d_4_proteomics	Protein	PeptideAtlas
SM001-P	SM001_SARS_10^4_4d_5_proteomics	Protein	PeptideAtlas
SM001-P	SM001_SARS_10^4_7d_1_proteomics	Protein	PeptideAtlas
SM001-P	SM001_SARS_10^4_7d_2_proteomics	Protein	PeptideAtlas
SM001-P	SM001_SARS_10^4_7d_3_proteomics	Protein	PeptideAtlas
SM001-P	SM001_SARS_10^4_7d_4_proteomics	Protein	PeptideAtlas
SM001-P	SM001_SARS_10^4_7d_5_proteomics	Protein	PeptideAtlas
SM001-P	SM001_SARS_10^5_1d_1_proteomics	Protein	PeptideAtlas
SM001-P	SM001_SARS_10^5_1d_2_proteomics	Protein	PeptideAtlas
SM001-P	SM001_SARS_10^5_1d_3_proteomics	Protein	PeptideAtlas
SM001-P	SM001_SARS_10^5_1d_4_proteomics	Protein	PeptideAtlas
SM001-P	SM001_SARS_10^5_1d_5_proteomics	Protein	PeptideAtlas
SM001-P	SM001_SARS_10^5_2d_1_proteomics	Protein	PeptideAtlas
SM001-P	SM001_SARS_10^5_2d_2_proteomics	Protein	PeptideAtlas
SM001-P	SM001_SARS_10^5_2d_3_proteomics	Protein	PeptideAtlas
SM001-P	SM001_SARS_10^5_2d_4_proteomics	Protein	PeptideAtlas
SM001-P	SM001_SARS_10^5_2d_5_proteomics	Protein	PeptideAtlas
SM001-P	SM001_SARS_10^5_4d_1_proteomics	Protein	PeptideAtlas
SM001-P	SM001_SARS_10^5_4d_2_proteomics	Protein	PeptideAtlas
SM001-P	SM001_SARS_10^5_4d_3_proteomics	Protein	PeptideAtlas
SM001-P	SM001_SARS_10^5_4d_4_proteomics	Protein	PeptideAtlas
SM001-P	SM001_SARS_10^5_4d_5_proteomics	Protein	PeptideAtlas
SM001-P	SM001_SARS_10^5_7d_1_proteomics	Protein	PeptideAtlas
SM001-P	SM001_SARS_10^5_7d_2_proteomics	Protein	PeptideAtlas
SM001-P	SM001_SARS_10^5_7d_3_proteomics	Protein	PeptideAtlas
SM001-P	SM001_SARS_10^5_7d_4_proteomics	Protein	PeptideAtlas
SM001-P	SM001_SARS_10^5_7d_5_proteomics	Protein	PeptideAtlas

**Table 4:** Sample tracking from the subject to the experiment sample level

STUDY ID	Subject ID	Species Name	Biological Sample ID	Sample Type	Experiment ID	Experiment Sample ID	Biological Sample Type	Experiment ID	Experiment Sample ID	Biological Sample Type
CA04M001	CA04M001_10^3_1d_2	Mus musculus	CA04M001_10^3_1d_2_lung	Tissue	CA04M001-R	CA04M001_10^3_1d_2_RNA_ExpSam	RNA			
CA04M001	CA04M001_10^3_1d_3	Mus musculus	CA04M001_10^3_1d_3_lung	Tissue	CA04M001-R	CA04M001_10^3_1d_3_RNA_ExpSam	RNA			
CA04M001	CA04M001_10^3_1d_4	Mus musculus	CA04M001_10^3_1d_4_lung	Tissue	CA04M001-R	CA04M001_10^3_1d_4_RNA_ExpSam	RNA			
CA04M001	CA04M001_10^3_2d_1	Mus musculus	CA04M001_10^3_2d_1_lung	Tissue	CA04M001-R	CA04M001_10^3_2d_1_RNA_ExpSam	RNA			
CA04M001	CA04M001_10^3_2d_2	Mus musculus	CA04M001_10^3_2d_2_lung	Tissue	CA04M001-R	CA04M001_10^3_2d_2_RNA_ExpSam	RNA			
CA04M001	CA04M001_10^3_2d_3	Mus musculus	CA04M001_10^3_2d_3_lung	Tissue	CA04M001-R	CA04M001_10^3_2d_3_RNA_ExpSam	RNA			
CA04M001	CA04M001_10^3_4d_1	Mus musculus	CA04M001_10^3_4d_1_lung	Tissue	CA04M001-R	CA04M001_10^3_4d_1_RNA_ExpSam	RNA			
CA04M001	CA04M001_10^3_4d_2	Mus musculus	CA04M001_10^3_4d_2_lung	Tissue	CA04M001-R	CA04M001_10^3_4d_2_RNA_ExpSam	RNA			
CA04M001	CA04M001_10^3_4d_3	Mus musculus	CA04M001_10^3_4d_3_lung	Tissue	CA04M001-R	CA04M001_10^3_4d_3_RNA_ExpSam	RNA			
CA04M001	CA04M001_10^3_4d_4	Mus musculus	CA04M001_10^3_4d_4_lung	Tissue	CA04M001-R	CA04M001_10^3_4d_4_RNA_ExpSam	RNA			
CA04M001	CA04M001_10^3_7d_2	Mus musculus	CA04M001_10^3_7d_2_lung	Tissue	CA04M001-R	CA04M001_10^3_7d_2_RNA_ExpSam	RNA			
CA04M001	CA04M001_10^3_7d_3	Mus musculus	CA04M001_10^3_7d_3_lung	Tissue	CA04M001-R	CA04M001_10^3_7d_3_RNA_ExpSam	RNA			
CA04M001	CA04M001_10^3_7d_4	Mus musculus	CA04M001_10^3_7d_4_lung	Tissue	CA04M001-R	CA04M001_10^3_7d_4_RNA_ExpSam	RNA			
CA04M001	CA04M001_10^4_1d_1	Mus musculus	CA04M001_10^4_1d_1_lung	Tissue	CA04M001-R	CA04M001_10^4_1d_1_RNA_ExpSam	RNA	CA04M001-P	CA04M001_CA04_10^4_1d_1_proteomics	Protein
CA04M001	CA04M001_10^4_1d_2	Mus musculus	CA04M001_10^4_1d_2_lung	Tissue	CA04M001-R	CA04M001_10^4_1d_2_RNA_ExpSam	RNA	CA04M001-P	CA04M001_CA04_10^4_1d_2_proteomics	Protein
CA04M001	CA04M001_10^4_1d_3	Mus musculus	CA04M001_10^4_1d_3_lung	Tissue	CA04M001-R	CA04M001_10^4_1d_3_RNA_ExpSam	RNA	CA04M001-P	CA04M001_CA04_10^4_1d_3_proteomics	Protein
CA04M001	CA04M001_10^4_1d_4	Mus musculus	CA04M001_10^4_1d_4_lung	Tissue	CA04M001-R	CA04M001_10^4_1d_4_RNA_ExpSam	RNA	CA04M001-P	CA04M001_CA04_10^4_1d_4_proteomics	Protein
CA04M001	CA04M001_10^4_2d_1	Mus musculus	CA04M001_10^4_2d_1_lung	Tissue	CA04M001-R	CA04M001_10^4_2d_1_RNA_ExpSam	RNA	CA04M001-P	CA04M001_CA04_10^4_2d_1_proteomics	Protein
CA04M001	CA04M001_10^4_2d_2	Mus musculus	CA04M001_10^4_2d_2_lung	Tissue	CA04M001-R	CA04M001_10^4_2d_2_RNA_ExpSam	RNA	CA04M001-P	CA04M001_CA04_10^4_2d_2_proteomics	Protein
CA04M001	CA04M001_10^4_2d_3	Mus musculus	CA04M001_10^4_2d_3_lung	Tissue	CA04M001-R	CA04M001_10^4_2d_3_RNA_ExpSam	RNA	CA04M001-P	CA04M001_CA04_10^4_2d_3_proteomics	Protein
CA04M001	CA04M001_10^4_2d_4	Mus musculus	CA04M001_10^4_2d_4_lung	Tissue	CA04M001-R	CA04M001_10^4_2d_4_RNA_ExpSam	RNA	CA04M001-P	CA04M001_CA04_10^4_2d_4_proteomics	Protein
CA04M001	CA04M001_10^4_4d_1	Mus musculus	CA04M001_10^4_4d_1_lung	Tissue	CA04M001-R	CA04M001_10^4_4d_1_RNA_ExpSam	RNA	CA04M001-P	CA04M001_CA04_10^4_4d_1_proteomics	Protein
CA04M001	CA04M001_10^4_4d_2	Mus musculus	CA04M001_10^4_4d_2_lung	Tissue	CA04M001-R	CA04M001_10^4_4d_2_RNA_ExpSam	RNA	CA04M001-P	CA04M001_CA04_10^4_4d_2_proteomics	Protein
CA04M001	CA04M001_10^4_4d_3	Mus musculus	CA04M001_10^4_4d_3_lung	Tissue	CA04M001-R	CA04M001_10^4_4d_3_RNA_ExpSam	RNA	CA04M001-P	CA04M001_CA04_10^4_4d_3_proteomics	Protein
CA04M001	CA04M001_10^4_7d_1	Mus musculus	CA04M001_10^4_7d_1_lung	Tissue	CA04M001-R	CA04M001_10^4_7d_1_RNA_ExpSam	RNA	CA04M001-P	CA04M001_CA04_10^4_7d_1_proteomics	Protein
CA04M001	CA04M001_10^4_7d_2	Mus musculus	CA04M001_10^4_7d_2_lung	Tissue	CA04M001-R	CA04M001_10^4_7d_2_RNA_ExpSam	RNA	CA04M001-P	CA04M001_CA04_10^4_7d_2_proteomics	Protein
CA04M001	CA04M001_10^4_7d_3	Mus musculus	CA04M001_10^4_7d_3_lung	Tissue	CA04M001-R	CA04M001_10^4_7d_3_RNA_ExpSam	RNA	CA04M001-P	CA04M001_CA04_10^4_7d_3_proteomics	Protein
CA04M001	CA04M001_10^5_1d_1	Mus musculus	CA04M001_10^5_1d_1_lung	Tissue	CA04M001-R	CA04M001_10^5_1d_1_RNA_ExpSam	RNA	CA04M001-P	CA04M001_CA04_10^5_1d_1_proteomics	Protein
CA04M001	CA04M001_10^5_1d_2	Mus musculus	CA04M001_10^5_1d_2_lung	Tissue	CA04M001-R	CA04M001_10^5_1d_2_RNA_ExpSam	RNA	CA04M001-P	CA04M001_CA04_10^5_1d_2_proteomics	Protein
CA04M001	CA04M001_10^5_1d_3	Mus musculus	CA04M001_10^5_1d_3_lung	Tissue	CA04M001-R	CA04M001_10^5_1d_3_RNA_ExpSam	RNA	CA04M001-P	CA04M001_CA04_10^5_1d_3_proteomics	Protein
CA04M001	CA04M001_10^5_1d_4	Mus musculus	CA04M001_10^5_1d_4_lung	Tissue	CA04M001-R	CA04M001_10^5_1d_4_RNA_ExpSam	RNA	CA04M001-P	CA04M001_CA04_10^5_1d_4_proteomics	Protein
CA04M001	CA04M001_10^5_2d_1	Mus musculus	CA04M001_10^5_2d_1_lung	Tissue	CA04M001-R	CA04M001_10^5_2d_1_RNA_ExpSam	RNA	CA04M001-P	CA04M001_CA04_10^5_2d_1_proteomics	Protein
CA04M001	CA04M001_10^5_2d_2	Mus musculus	CA04M001_10^5_2d_2_lung	Tissue	CA04M001-R	CA04M001_10^5_2d_2_RNA_ExpSam	RNA	CA04M001-P	CA04M001_CA04_10^5_2d_2_proteomics	Protein
CA04M001	CA04M001_10^5_2d_3	Mus musculus	CA04M001_10^5_2d_3_lung	Tissue	CA04M001-R	CA04M001_10^5_2d_3_RNA_ExpSam	RNA	CA04M001-P	CA04M001_CA04_10^5_2d_3_proteomics	Protein
CA04M001	CA04M001_10^5_2d_4	Mus musculus	CA04M001_10^5_2d_4_lung	Tissue	CA04M001-R	CA04M001_10^5_2d_4_RNA_ExpSam	RNA	CA04M001-P	CA04M001_CA04_10^5_2d_4_proteomics	Protein
CA04M001	CA04M001_10^5_4d_1	Mus musculus	CA04M001_10^5_4d_1_lung	Tissue	CA04M001-R	CA04M001_10^5_4d_1_RNA_ExpSam	RNA	CA04M001-P	CA04M001_CA04_10^5_4d_1_proteomics	Protein
CA04M001	CA04M001_10^5_4d_2	Mus musculus	CA04M001_10^5_4d_2_lung	Tissue	CA04M001-R	CA04M001_10^5_4d_2_RNA_ExpSam	RNA	CA04M001-P	CA04M001_CA04_10^5_4d_2_proteomics	Protein
CA04M001	CA04M001_10^5_4d_3	Mus musculus	CA04M001_10^5_4d_3_lung	Tissue	CA04M001-R	CA04M001_10^5_4d_3_RNA_ExpSam	RNA	CA04M001-P	CA04M001_CA04_10^5_4d_3_proteomics	Protein
CA04M001	CA04M001_10^5_4d_4	Mus musculus	CA04M001_10^5_4d_4_lung	Tissue	CA04M001-R	CA04M001_10^5_4d_4_RNA_ExpSam	RNA	CA04M001-P	CA04M001_CA04_10^5_4d_4_proteomics	Protein
CA04M001	CA04M001_10^5_7d_1	Mus musculus	CA04M001_10^5_7d_1_lung	Tissue	CA04M001-R	CA04M001_10^5_7d_1_RNA_ExpSam	RNA	CA04M001-P	CA04M001_CA04_10^5_7d_1_proteomics	Protein
CA04M001	CA04M001_10^5_7d_2	Mus musculus	CA04M001_10^5_7d_2_lung	Tissue	CA04M001-R	CA04M001_10^5_7d_2_RNA_ExpSam	RNA	CA04M001-P	CA04M001_CA04_10^5_7d_2_proteomics	Protein
CA04M001	CA04M001_10^5_7d_3	Mus musculus	CA04M001_10^5_7d_3_lung	Tissue	CA04M001-R	CA04M001_10^5_7d_3_RNA_ExpSam	RNA	CA04M001-P	CA04M001_CA04_10^5_7d_3_proteomics	Protein
CA04M001	CA04M001_10^5_7d_4	Mus musculus	CA04M001_10^5_7d_4_lung	Tissue	CA04M001-R	CA04M001_10^5_7d_4_RNA_ExpSam	RNA	CA04M001-P	CA04M001_CA04_10^5_7d_4_proteomics	Protein
CA04M001	CA04M001_10^5_7d_5	Mus musculus	CA04M001_10^5_7d_5_lung	Tissue	CA04M001-R	CA04M001_10^5_7d_5_RNA_ExpSam	RNA	CA04M001-P	CA04M001_CA04_10^5_7d_5_proteomics	Protein
CA04M001	CA04M001_10^6_1d_1	Mus musculus	CA04M001_10^6_1d_1_lung	Tissue	CA04M001-R	CA04M001_10^6_1d_1_RNA_ExpSam	RNA			
CA04M001	CA04M001_10^6_1d_3	Mus musculus	CA04M001_10^6_1d_3_lung	Tissue	CA04M001-R	CA04M001_10^6_1d_3_RNA_ExpSam	RNA			
CA04M001	CA04M001_10^6_1d_4	Mus musculus	CA04M001_10^6_1d_4_lung	Tissue	CA04M001-R	CA04M001_10^6_1d_4_RNA_ExpSam	RNA			
CA04M001	CA04M001_10^6_2d_1	Mus musculus	CA04M001_10^6_2d_1_lung	Tissue	CA04M001-R	CA04M001_10^6_2d_1_RNA_ExpSam	RNA			
CA04M001	CA04M001_10^6_2d_2	Mus musculus	CA04M001_10^6_2d_2_lung	Tissue	CA04M001-R	CA04M001_10^6_2d_2_RNA_ExpSam	RNA			
CA04M001	CA04M001_10^6_2d_3	Mus musculus	CA04M001_10^6_2d_3_lung	Tissue	CA04M001-R	CA04M001_10^6_2d_3_RNA_ExpSam	RNA			
CA04M001	CA04M001_10^6_2d_4	Mus musculus	CA04M001_10^6_2d_4_lung	Tissue	CA04M001-R	CA04M001_10^6_2d_4_RNA_ExpSam	RNA			
CA04M001	CA04M001_10^6_4d_1	Mus musculus	CA04M001_10^6_4d_1_lung	Tissue	CA04M001-R	CA04M001_10^6_4d_1_RNA_ExpSam	RNA			
CA04M001	CA04M001_10^6_4d_2	Mus musculus	CA04M001_10^6_4d_2_lung	Tissue	CA04M001-R	CA04M001_10^6_4d_2_RNA_ExpSam	RNA			
CA04M001	CA04M001_10^6_4d_3	Mus musculus	CA04M001_10^6_4d_3_lung	Tissue	CA04M001-R	CA04M001_10^6_4d_3_RNA_ExpSam	RNA			
CA04M001	CA04M001_10^6_7d_1	Mus musculus	CA04M001_10^6_7d_1_lung	Tissue	CA04M001-R	CA04M001_10^6_7d_1_RNA_ExpSam	RNA			
CA04M001	CA04M001_10^6_7d_2	Mus musculus	CA04M001_10^6_7d_2_lung	Tissue	CA04M001-R	CA04M001_10^6_7d_2_RNA_ExpSam	RNA			
CA04M001	CA04M001_10^6_7d_3	Mus musculus	CA04M001_10^6_7d_3_lung	Tissue	CA04M001-R	CA04M001_10^6_7d_3_RNA_ExpSam	RNA			
CA04M001	CA04M001_10^6_7d_4	Mus musculus	CA04M001_10^6_7d_4_lung	Tissue	CA04M001-R	CA04M001_10^6_7d_4_RNA_ExpSam	RNA			
CA04M001	CA04M001_10^6_7d_5	Mus musculus	CA04M001_10^6_7d_5_lung	Tissue	CA04M001-R	CA04M001_10^6_7d_5_RNA_ExpSam	RNA			
CA04M001	CA04M001_mock_1d_1	Mus musculus	CA04M001_mock_1d_1_lung	Tissue	CA04M001-R	CA04M001_mock_1d_1_RNA_ExpSam	RNA	CA04M001-P	CA04M001_Mock_1d_1_proteomics	Protein
CA04M001	CA04M001_mock_1d_2	Mus musculus	CA04M001_mock_1d_2_lung	Tissue	CA04M001-R	CA04M001_mock_1d_2_RNA_ExpSam	RNA	CA04M001-P	CA04M001_Mock_1d_2_proteomics	Protein
CA04M001	CA04M001_mock_2d_1	Mus musculus	CA04M001_mock_2d_1_lung	Tissue	CA04M001-R	CA04M001_mock_2d_1_RNA_ExpSam	RNA	CA04M001-P	CA04M001_Mock_2d_1_proteomics	Protein
CA04M001	CA04M001_mock_2d_2	Mus musculus	CA04M001_mock_2d_2_lung	Tissue	CA04M001-R	CA04M001_mock_2d_2_RNA_ExpSam	RNA			
CA04M001	CA04M001_mock_4d_1	Mus musculus	CA04M001_mock_4d_1_lung	Tissue	CA04M001-R	CA04M001_mock_4d_1_RNA_ExpSam	RNA	CA04M001-P	CA04M001_Mock_4d_1_proteomics	Protein
CA04M001	CA04M001_mock_4d_2	Mus musculus	CA04M001_mock_4d_2_lung	Tissue	CA04M001-R	CA04M001_mock_4d_2_RNA_ExpSam	RNA	CA04M001-P	CA04M001_Mock_4d_2_proteomics	Protein
CA04M001	CA04M001_mock_4d_3	Mus musculus	CA04M001_mock_4d_3_lung	Tissue	CA04M001-R	CA04M001_mock_4d_3_RNA_ExpSam	RNA	CA04M001-P	CA04M001_Mock_4d_3_proteomics	Protein
CA04M001	CA04M001_mock_7d_1	Mus musculus	CA04M001_mock_7d_1_lung	Tissue	CA04M001-R	CA04M001_mock_7d_1_RNA_ExpSam	RNA	CA04M001-P	CA04M001_Mock_7d_1_proteomics	Protein
CA04M001	CA04M001_mock_7d_2	Mus musculus	CA04M001_mock_7d_2_lung	Tissue	CA04M001-R	CA04M001_mock_7d_2_RNA_ExpSam	RNA	CA04M001-P	CA04M001_Mock_7d_2_proteomics	Protein
CA04M001	CA04M001_mock_7d_3	Mus musculus	CA04M001_mock_7d_3_lung	Tissue	CA04M001-R	CA04M001_mock_7d_3_RNA_ExpSam	RNA	CA04M001-P	CA04M001_Mock_7d_3_proteomics	Protein
ECL001	ECL001 2B-4 Cells	Homo sapiens	ECL001_Mock_0hr_1_Cells	Cells	ECL001-R	ECL001_Mock_0h_1_array	RNA			
ECL001	ECL001 2B-4 Cells	Homo sapiens	ECL001_Mock_0hr_2_Cells	Cells	ECL001-R	ECL001_Mock_0h_2_array	RNA			
ECL001	ECL001 2B-4 Cells	Homo sapiens	ECL001_Mock_0hr_3_Cells	Cells	ECL001-R	ECL001_Mock_0h_3_array	RNA			
ECL001	ECL001 2B-4 Cells	Homo sapiens	ECL001_Mock_3hr_1_Cells	Cells	ECL001-R	ECL001_Mock_3h_1_array	RNA			
ECL001	ECL001 2B-4 Cells	Homo sapiens	ECL001_Mock_3hr_2_Cells	Cells	ECL001-R	ECL001_Mock_3h_2_array	RNA			
ECL001	ECL001 2B-4 Cells	Homo sapiens	ECL001_Mock_3hr_3_Cells	Cells	ECL001-R	ECL001_Mock_3h_3_array	RNA			
ECL001	ECL001 2B-4 Cells	Homo sapiens	ECL001_Mock_7hr_1_Cells	Cells	ECL001-R	ECL001_Mock_7h_1_array	RNA			
ECL001	ECL001 2B-4 Cells	Homo sapiens	ECL001_Mock_7hr_2_Cells	Cells	ECL001-R	ECL001_Mock_7h_2_array	RNA			
ECL001	ECL001 2B-4 Cells	Homo sapiens	ECL001_Mock_7hr_3_Cells	Cells	ECL001-R	ECL001_Mock_7h_3_array	RNA			
ECL001	ECL001 2B-4 Cells	Homo sapiens	ECL001_Mock_12hr_1_Cells	Cells	ECL001-R	ECL001_Mock_12h_1_array	RNA			
ECL001	ECL001 2B-4 Cells	Homo sapiens	ECL001_Mock_12hr_2_Cells	Cells	ECL001-R	ECL001_Mock_12h_2_array	RNA			
ECL001	ECL001 2B-4 Cells	Homo sapiens	ECL001_Mock_12hr_3_Cells	Cells	ECL001-R	ECL001_Mock_12h_3_array	RNA			
ECL001	ECL001 2B-4 Cells	Homo sapiens	ECL001_Mock_18hr_1_Cells	Cells	ECL001-R		RNA			
ECL001	ECL001 2B-4 Cells	Homo sapiens	ECL001_Mock_18hr_2_Cells	Cells	ECL001-R		RNA			
ECL001	ECL001 2B-4 Cells	Homo sapiens	ECL001_Mock_18hr_3_Cells	Cells	ECL001-R		RNA			
ECL001	ECL001 2B-4 Cells	Homo sapiens	ECL001_Mock_24hr_1_Cells	Cells	ECL001-R	ECL001_Mock_24h_1_array	RNA			
ECL001	ECL001 2B-4 Cells	Homo sapiens	ECL001_Mock_24hr_2_Cells	Cells	ECL001-R	ECL001_Mock_24h_2_array	RNA			
ECL001	ECL001 2B-4 Cells	Homo sapiens	ECL001_Mock_24hr_3_Cells	Cells	ECL001-R	ECL001_Mock_24h_3_array	RNA			
ECL001	ECL001 2B-4 Cells	Homo sapiens	ECL001_EMC_0hr_1_Cells	Cells	ECL001-R	ECL001_EMC_0h_1_array	RNA			
ECL001	ECL001 2B-4 Cells	Homo sapiens	ECL001_EMC_0hr_2_Cells	Cells	ECL001-R	ECL001_EMC_0h_2_array	RNA			
ECL001	ECL001 2B-4 Cells	Homo sapiens	ECL001_EMC_0hr_3_Cells	Cells	ECL001-R	ECL001_EMC_0h_3_array	RNA			
ECL001	ECL001 2B-4 Cells	Homo sapiens	ECL001_EMC_3hr_1_Cells	Cells	ECL001-R	ECL001_EMC_3h_1_array	RNA			
ECL001	ECL001 2B-4 Cells	Homo sapiens	ECL001_EMC_3hr_2_Cells	Cells	ECL001-R	ECL001_EMC_3h_2_array	RNA			
ECL001	ECL001 2B-4 Cells	Homo sapiens	ECL001_EMC_3hr_3_Cells	Cells	ECL001-R	ECL001_EMC_3h_3_array	RNA			
ECL001	ECL001 2B-4 Cells	Homo sapiens	ECL001_EMC_7hr_1_Cells	Cells	ECL001-R	ECL001_EMC_7h_1_array	RNA			
ECL001	ECL001 2B-4 Cells	Homo sapiens	ECL001_EMC_7hr_2_Cells	Cells	ECL001-R	ECL001_EMC_7h_2_array	RNA			
ECL001	ECL001 2B-4 Cells	Homo sapiens	ECL001_EMC_7hr_3_Cells	Cells	ECL001-R	ECL001_EMC_7h_3_array	RNA			
ECL001	ECL001 2B-4 Cells	Homo sapiens	ECL001_EMC_12hr_1_Cells	Cells	ECL001-R		RNA			
ECL001	ECL001 2B-4 Cells	Homo sapiens	ECL001_EMC_12hr_2_Cells	Cells	ECL001-R	ECL001_EMC_12h_2_array	RNA			
ECL001	ECL001 2B-4 Cells	Homo sapiens	ECL001_EMC_12hr_3_Cells	Cells	ECL001-R	ECL001_EMC_12h_3_array	RNA			
ECL001	ECL001 2B-4 Cells	Homo sapiens	ECL001_EMC_18hr_1_Cells	Cells	ECL001-R	ECL001_EMC_18h_1_array	RNA			
ECL001	ECL001 2B-4 Cells	Homo sapiens	ECL001_EMC_18hr_2_Cells	Cells	ECL001-R	ECL001_EMC_18h_2_array	RNA			
ECL001	ECL001 2B-4 Cells	Homo sapiens	ECL001_EMC_18hr_3_Cells	Cells	ECL001-R	ECL001_EMC_18h_3_array	RNA			
ECL001	ECL001 2B-4 Cells	Homo sapiens	ECL001_EMC_24hr_1_Cells	Cells	ECL001-R	ECL001_EMC_24h_1_array	RNA			
ECL001	ECL001 2B-4 Cells	Homo sapiens	ECL001_EMC_24hr_2_Cells	Cells	ECL001-R	ECL001_EMC_24h_2_array	RNA			
ECL001	ECL001 2B-4 Cells	Homo sapiens	ECL001_EMC_24hr_3_Cells	Cells	ECL001-R	ECL001_EMC_24h_3_array	RNA			
ICL004	ICL004 Calu-3 Cells	Homo sapiens	ICL004_Mock_0H_1_cell	Cells	ICL004-R	ICL004_Mock_0H_1_RNA_ExpSam	RNA	ICL004-P	ICL004_Mock_0h_1_proteomics	Protein
ICL004	ICL004 Calu-3 Cells	Homo sapiens	ICL004_Mock_0H_2_cell	Cells	ICL004-R	ICL004_Mock_0H_2_RNA_ExpSam	RNA	ICL004-P	ICL004_Mock_0h_2_proteomics	Protein
ICL004	ICL004 Calu-3 Cells	Homo sapiens	ICL004_Mock_0H_3_cell	Cells	ICL004-R	ICL004_Mock_0H_3_RNA_ExpSam	RNA	ICL004-P	ICL004_Mock_0h_3_proteomics	Protein
ICL004	ICL004 Calu-3 Cells	Homo sapiens	ICL004_Mock_12H_1_cell	Cells	ICL004-R	ICL004_Mock_12H_1_RNA_ExpSam	RNA	ICL004-P	ICL004_Mock_12h_1_proteomics	Protein
ICL004	ICL004 Calu-3 Cells	Homo sapiens	ICL004_Mock_12H_2_cell	Cells	ICL004-R	ICL004_Mock_12H_2_RNA_ExpSam	RNA	ICL004-P	ICL004_Mock_12h_2_proteomics	Protein
ICL004	ICL004 Calu-3 Cells	Homo sapiens	ICL004_Mock_12H_3_cell	Cells	ICL004-R	ICL004_Mock_12H_3_RNA_ExpSam	RNA	ICL004-P	ICL004_Mock_12h_3_proteomics	Protein
ICL004	ICL004 Calu-3 Cells	Homo sapiens	ICL004_Mock_18H_1_cell	Cells	ICL004-R	ICL004_Mock_18H_1_RNA_ExpSam	RNA	ICL004-P	ICL004_Mock_18h_1_proteomics	Protein
ICL004	ICL004 Calu-3 Cells	Homo sapiens	ICL004_Mock_18H_2_cell	Cells	ICL004-R	ICL004_Mock_18H_2_RNA_ExpSam	RNA	ICL004-P	ICL004_Mock_18h_2_proteomics	Protein
ICL004	ICL004 Calu-3 Cells	Homo sapiens	ICL004_Mock_18H_3_cell	Cells	ICL004-R	ICL004_Mock_18H_3_RNA_ExpSam	RNA	ICL004-P	ICL004_Mock_18h_3_proteomics	Protein
ICL004	ICL004 Calu-3 Cells	Homo sapiens	ICL004_Mock_24H_1_cell	Cells	ICL004-R	ICL004_Mock_24H_1_RNA_ExpSam	RNA	ICL004-P	ICL004_Mock_24h_1_proteomics	Protein
ICL004	ICL004 Calu-3 Cells	Homo sapiens	ICL004_Mock_24H_2_cell	Cells	ICL004-R	ICL004_Mock_24H_2_RNA_ExpSam	RNA			
ICL004	ICL004 Calu-3 Cells	Homo sapiens	ICL004_Mock_24H_3_cell	Cells	ICL004-R	ICL004_Mock_24H_3_RNA_ExpSam	RNA	ICL004-P	ICL004_Mock_24h_3_proteomics	Protein
ICL004	ICL004 Calu-3 Cells	Homo sapiens	ICL004_Mock_3H_1_cell	Cells	ICL004-R	ICL004_Mock_3H_1_RNA_ExpSam	RNA	ICL004-P	ICL004_Mock_3h_1_proteomics	Protein
ICL004	ICL004 Calu-3 Cells	Homo sapiens	ICL004_Mock_3H_2_cell	Cells	ICL004-R	ICL004_Mock_3H_2_RNA_ExpSam	RNA			
ICL004	ICL004 Calu-3 Cells	Homo sapiens	ICL004_Mock_3H_3_cell	Cells	ICL004-R	ICL004_Mock_3H_3_RNA_ExpSam	RNA			
ICL004	ICL004 Calu-3 Cells	Homo sapiens	ICL004_Mock_7H_1_cell	Cells	ICL004-R	ICL004_Mock_7H_1_RNA_ExpSam	RNA	ICL004-P	ICL004_Mock_7h_1_proteomics	Protein
ICL004	ICL004 Calu-3 Cells	Homo sapiens	ICL004_Mock_7H_2_cell	Cells	ICL004-R	ICL004_Mock_7H_2_RNA_ExpSam	RNA	ICL004-P	ICL004_Mock_7h_2_proteomics	Protein
ICL004	ICL004 Calu-3 Cells	Homo sapiens	ICL004_Mock_7H_3_cell	Cells	ICL004-R	ICL004_Mock_7H_3_RNA_ExpSam	RNA	ICL004-P	ICL004_Mock_7h_3_proteomics	Protein
ICL004	ICL004 Calu-3 Cells	Homo sapiens	ICL004_VN1203_0H_1_cell	Cells	ICL004-R	ICL004_VN1203_0H_1_RNA_ExpSam	RNA	ICL004-P	ICL004_VN1203_0h_1_proteomics	Protein
ICL004	ICL004 Calu-3 Cells	Homo sapiens	ICL004_VN1203_0H_2_cell	Cells	ICL004-R	ICL004_VN1203_0H_2_RNA_ExpSam	RNA	ICL004-P	ICL004_VN1203_0h_2_proteomics	Protein
ICL004	ICL004 Calu-3 Cells	Homo sapiens	ICL004_VN1203_0H_3_cell	Cells	ICL004-R	ICL004_VN1203_0H_3_RNA_ExpSam	RNA			
ICL004	ICL004 Calu-3 Cells	Homo sapiens	ICL004_VN1203_12H_1_cell	Cells	ICL004-R	ICL004_VN1203_12H_1_RNA_ExpSam	RNA			
ICL004	ICL004 Calu-3 Cells	Homo sapiens	ICL004_VN1203_12H_2_cell	Cells	ICL004-R	ICL004_VN1203_12H_2_RNA_ExpSam	RNA	ICL004-P	ICL004_VN1203_12h_2_proteomics	Protein
ICL004	ICL004 Calu-3 Cells	Homo sapiens	ICL004_VN1203_12H_3_cell	Cells	ICL004-R	ICL004_VN1203_12H_3_RNA_ExpSam	RNA	ICL004-P	ICL004_VN1203_12h_3_proteomics	Protein
ICL004	ICL004 Calu-3 Cells	Homo sapiens	ICL004_VN1203_18H_1_cell	Cells	ICL004-R	ICL004_VN1203_18H_1_RNA_ExpSam	RNA	ICL004-P	ICL004_VN1203_18h_1_proteomics	Protein
ICL004	ICL004 Calu-3 Cells	Homo sapiens	ICL004_VN1203_18H_2_cell	Cells	ICL004-R	ICL004_VN1203_18H_2_RNA_ExpSam	RNA	ICL004-P	ICL004_VN1203_18h_2_proteomics	Protein
ICL004	ICL004 Calu-3 Cells	Homo sapiens	ICL004_VN1203_18H_3_cell	Cells	ICL004-R	ICL004_VN1203_18H_3_RNA_ExpSam	RNA	ICL004-P	ICL004_VN1203_18h_3_proteomics	Protein
ICL004	ICL004 Calu-3 Cells	Homo sapiens	ICL004_VN1203_24H_1_cell	Cells	ICL004-R	ICL004_VN1203_24H_1_RNA_ExpSam	RNA	ICL004-P	ICL004_VN1203_24h_1_proteomics	Protein
ICL004	ICL004 Calu-3 Cells	Homo sapiens	ICL004_VN1203_24H_2_cell	Cells	ICL004-R	ICL004_VN1203_24H_2_RNA_ExpSam	RNA	ICL004-P	ICL004_VN1203_24h_2_proteomics	Protein
ICL004	ICL004 Calu-3 Cells	Homo sapiens	ICL004_VN1203_24H_3_cell	Cells	ICL004-R	ICL004_VN1203_24H_3_RNA_ExpSam	RNA			
ICL004	ICL004 Calu-3 Cells	Homo sapiens	ICL004_VN1203_3H_1_cell	Cells	ICL004-R	ICL004_VN1203_3H_1_RNA_ExpSam	RNA	ICL004-P	ICL004_VN1203_3h_1_proteomics	Protein
ICL004	ICL004 Calu-3 Cells	Homo sapiens	ICL004_VN1203_3H_2_cell	Cells	ICL004-R	ICL004_VN1203_3H_2_RNA_ExpSam	RNA	ICL004-P	ICL004_VN1203_3h_2_proteomics	Protein
ICL004	ICL004 Calu-3 Cells	Homo sapiens	ICL004_VN1203_3H_3_cell	Cells	ICL004-R	ICL004_VN1203_3H_3_RNA_ExpSam	RNA			
ICL004	ICL004 Calu-3 Cells	Homo sapiens	ICL004_VN1203_7H_1_cell	Cells	ICL004-R	ICL004_VN1203_7H_1_RNA_ExpSam	RNA	ICL004-P	ICL004_VN1203_7h_1_proteomics	Protein
ICL004	ICL004 Calu-3 Cells	Homo sapiens	ICL004_VN1203_7H_2_cell	Cells	ICL004-R	ICL004_VN1203_7H_2_RNA_ExpSam	RNA	ICL004-P	ICL004_VN1203_7h_2_proteomics	Protein
ICL004	ICL004 Calu-3 Cells	Homo sapiens	ICL004_VN1203_7H_3_cell	Cells	ICL004-R	ICL004_VN1203_7H_3_RNA_ExpSam	RNA	ICL004-P	ICL004_VN1203_7h_3_proteomics	Protein
ICL006	ICL006 Calu-3 Cells	Homo sapiens	ICL006_CA04_0h_1_cell	Cells	ICL006-R	ICL006_CA04_0h_1_RNA_ExpSam	RNA	ICL006-P	ICL006_CA04_0h_1_proteomics	Protein
ICL006	ICL006 Calu-3 Cells	Homo sapiens	ICL006_CA04_0h_2_cell	Cells	ICL006-R	ICL006_CA04_0h_2_RNA_ExpSam	RNA	ICL006-P	ICL006_CA04_0h_2_proteomics	Protein
ICL006	ICL006 Calu-3 Cells	Homo sapiens	ICL006_CA04_0h_3_cell	Cells	ICL006-R	ICL006_CA04_0h_3_RNA_ExpSam	RNA	ICL006-P	ICL006_CA04_0h_3_proteomics	Protein
ICL006	ICL006 Calu-3 Cells	Homo sapiens	ICL006_CA04_12h_1_cell	Cells	ICL006-R	ICL006_CA04_12h_1_RNA_ExpSam	RNA	ICL006-P	ICL006_CA04_12h_1_proteomics	Protein
ICL006	ICL006 Calu-3 Cells	Homo sapiens	ICL006_CA04_12h_2_cell	Cells	ICL006-R	ICL006_CA04_12h_2_RNA_ExpSam	RNA	ICL006-P	ICL006_CA04_12h_2_proteomics	Protein
ICL006	ICL006 Calu-3 Cells	Homo sapiens	ICL006_CA04_12h_3_cell	Cells	ICL006-R	ICL006_CA04_12h_3_RNA_ExpSam	RNA	ICL006-P	ICL006_CA04_12h_3_proteomics	Protein
ICL006	ICL006 Calu-3 Cells	Homo sapiens	ICL006_CA04_18h_1_cell	Cells	ICL006-R	ICL006_CA04_18h_1_RNA_ExpSam	RNA	ICL006-P	ICL006_CA04_18h_1_proteomics	Protein
ICL006	ICL006 Calu-3 Cells	Homo sapiens	ICL006_CA04_18h_2_cell	Cells	ICL006-R	ICL006_CA04_18h_2_RNA_ExpSam	RNA	ICL006-P	ICL006_CA04_18h_2_proteomics	Protein
ICL006	ICL006 Calu-3 Cells	Homo sapiens	ICL006_CA04_18h_3_cell	Cells	ICL006-R	ICL006_CA04_18h_3_RNA_ExpSam	RNA	ICL006-P	ICL006_CA04_18h_3_proteomics	Protein
ICL006	ICL006 Calu-3 Cells	Homo sapiens	ICL006_CA04_24h_1_cell	Cells	ICL006-R	ICL006_CA04_24h_1_RNA_ExpSam	RNA	ICL006-P	ICL006_CA04_24h_1_proteomics	Protein
ICL006	ICL006 Calu-3 Cells	Homo sapiens	ICL006_CA04_24h_2_cell	Cells	ICL006-R	ICL006_CA04_24h_2_RNA_ExpSam	RNA	ICL006-P	ICL006_CA04_24h_2_proteomics	Protein
ICL006	ICL006 Calu-3 Cells	Homo sapiens	ICL006_CA04_24h_3_cell	Cells	ICL006-R	ICL006_CA04_24h_3_RNA_ExpSam	RNA	ICL006-P	ICL006_CA04_24h_3_proteomics	Protein
ICL006	ICL006 Calu-3 Cells	Homo sapiens	ICL006_CA04_30h_1_cell	Cells	ICL006-R	ICL006_CA04_30h_1_RNA_ExpSam	RNA	ICL006-P	ICL006_CA04_30h_1_proteomics	Protein
ICL006	ICL006 Calu-3 Cells	Homo sapiens	ICL006_CA04_30h_2_cell	Cells	ICL006-R	ICL006_CA04_30h_2_RNA_ExpSam	RNA	ICL006-P	ICL006_CA04_30h_2_proteomics	Protein
ICL006	ICL006 Calu-3 Cells	Homo sapiens	ICL006_CA04_30h_3_cell	Cells	ICL006-R	ICL006_CA04_30h_3_RNA_ExpSam	RNA	ICL006-P	ICL006_CA04_30h_3_proteomics	Protein
ICL006	ICL006 Calu-3 Cells	Homo sapiens	ICL006_CA04_36h_1_cell	Cells	ICL006-R	ICL006_CA04_36h_1_RNA_ExpSam	RNA	ICL006-P	ICL006_CA04_36h_1_proteomics	Protein
ICL006	ICL006 Calu-3 Cells	Homo sapiens	ICL006_CA04_36h_2_cell	Cells	ICL006-R	ICL006_CA04_36h_2_RNA_ExpSam	RNA	ICL006-P	ICL006_CA04_36h_2_proteomics	Protein
ICL006	ICL006 Calu-3 Cells	Homo sapiens	ICL006_CA04_36h_3_cell	Cells	ICL006-R	ICL006_CA04_36h_3_RNA_ExpSam	RNA	ICL006-P	ICL006_CA04_36h_3_proteomics	Protein
ICL006	ICL006 Calu-3 Cells	Homo sapiens	ICL006_CA04_3h_1_cell	Cells	ICL006-R	ICL006_CA04_3h_1_RNA_ExpSam	RNA	ICL006-P	ICL006_CA04_3h_1_proteomics	Protein
ICL006	ICL006 Calu-3 Cells	Homo sapiens	ICL006_CA04_3h_2_cell	Cells	ICL006-R	ICL006_CA04_3h_2_RNA_ExpSam	RNA	ICL006-P	ICL006_CA04_3h_2_proteomics	Protein
ICL006	ICL006 Calu-3 Cells	Homo sapiens	ICL006_CA04_3h_3_cell	Cells	ICL006-R	ICL006_CA04_3h_3_RNA_ExpSam	RNA	ICL006-P	ICL006_CA04_3h_3_proteomics	Protein
ICL006	ICL006 Calu-3 Cells	Homo sapiens	ICL006_CA04_48h_1_cell	Cells	ICL006-R	ICL006_CA04_48h_1_RNA_ExpSam	RNA	ICL006-P	ICL006_CA04_48h_1_proteomics	Protein
ICL006	ICL006 Calu-3 Cells	Homo sapiens	ICL006_CA04_48h_2_cell	Cells	ICL006-R	ICL006_CA04_48h_2_RNA_ExpSam	RNA	ICL006-P	ICL006_CA04_48h_2_proteomics	Protein
ICL006	ICL006 Calu-3 Cells	Homo sapiens	ICL006_CA04_48h_3_cell	Cells	ICL006-R	ICL006_CA04_48h_3_RNA_ExpSam	RNA	ICL006-P	ICL006_CA04_48h_3_proteomics	Protein
ICL006	ICL006 Calu-3 Cells	Homo sapiens	ICL006_CA04_7h_1_cell	Cells	ICL006-R	ICL006_CA04_7h_1_RNA_ExpSam	RNA	ICL006-P	ICL006_CA04_7h_1_proteomics	Protein
ICL006	ICL006 Calu-3 Cells	Homo sapiens	ICL006_CA04_7h_2_cell	Cells	ICL006-R	ICL006_CA04_7h_2_RNA_ExpSam	RNA	ICL006-P	ICL006_CA04_7h_2_proteomics	Protein
ICL006	ICL006 Calu-3 Cells	Homo sapiens	ICL006_CA04_7h_3_cell	Cells	ICL006-R	ICL006_CA04_7h_3_RNA_ExpSam	RNA	ICL006-P	ICL006_CA04_7h_3_proteomics	Protein
ICL006	ICL006 Calu-3 Cells	Homo sapiens	ICL006_mock_0h_1_cell	Cells	ICL006-R	ICL006_mock_0h_1_RNA_ExpSam	RNA	ICL006-P	ICL006_Mock_0h_1_proteomics	Protein
ICL006	ICL006 Calu-3 Cells	Homo sapiens	ICL006_mock_0h_2_cell	Cells	ICL006-R	ICL006_mock_0h_2_RNA_ExpSam	RNA	ICL006-P	ICL006_Mock_0h_2_proteomics	Protein
ICL006	ICL006 Calu-3 Cells	Homo sapiens	ICL006_mock_0h_3_cell	Cells	ICL006-R	ICL006_mock_0h_3_RNA_ExpSam	RNA	ICL006-P	ICL006_Mock_0h_3_proteomics	Protein
ICL006	ICL006 Calu-3 Cells	Homo sapiens	ICL006_mock_12h_1_cell	Cells	ICL006-R	ICL006_mock_12h_1_RNA_ExpSam	RNA	ICL006-P	ICL006_Mock_12h_1_proteomics	Protein
ICL006	ICL006 Calu-3 Cells	Homo sapiens	ICL006_mock_12h_2_cell	Cells	ICL006-R	ICL006_mock_12h_2_RNA_ExpSam	RNA	ICL006-P	ICL006_Mock_12h_2_proteomics	Protein
ICL006	ICL006 Calu-3 Cells	Homo sapiens	ICL006_mock_12h_3_cell	Cells	ICL006-R	ICL006_mock_12h_3_RNA_ExpSam	RNA	ICL006-P	ICL006_Mock_12h_3_proteomics	Protein
ICL006	ICL006 Calu-3 Cells	Homo sapiens	ICL006_mock_18h_1_cell	Cells	ICL006-R	ICL006_mock_18h_1_RNA_ExpSam	RNA	ICL006-P	ICL006_Mock_18h_1_proteomics	Protein
ICL006	ICL006 Calu-3 Cells	Homo sapiens	ICL006_mock_18h_2_cell	Cells	ICL006-R	ICL006_mock_18h_2_RNA_ExpSam	RNA	ICL006-P	ICL006_Mock_18h_2_proteomics	Protein
ICL006	ICL006 Calu-3 Cells	Homo sapiens	ICL006_mock_18h_3_cell	Cells	ICL006-R	ICL006_mock_18h_3_RNA_ExpSam	RNA	ICL006-P	ICL006_Mock_18h_3_proteomics	Protein
ICL006	ICL006 Calu-3 Cells	Homo sapiens	ICL006_mock_24h_1_cell	Cells	ICL006-R	ICL006_mock_24h_1_RNA_ExpSam	RNA	ICL006-P	ICL006_Mock_24h_1_proteomics	Protein
ICL006	ICL006 Calu-3 Cells	Homo sapiens	ICL006_mock_24h_2_cell	Cells	ICL006-R	ICL006_mock_24h_2_RNA_ExpSam	RNA	ICL006-P	ICL006_Mock_24h_2_proteomics	Protein
ICL006	ICL006 Calu-3 Cells	Homo sapiens	ICL006_mock_24h_3_cell	Cells	ICL006-R	ICL006_mock_24h_3_RNA_ExpSam	RNA	ICL006-P	ICL006_Mock_24h_3_proteomics	Protein
ICL006	ICL006 Calu-3 Cells	Homo sapiens	ICL006_mock_30h_1_cell	Cells	ICL006-R	ICL006_mock_30h_1_RNA_ExpSam	RNA	ICL006-P	ICL006_Mock_30h_1_proteomics	Protein
ICL006	ICL006 Calu-3 Cells	Homo sapiens	ICL006_mock_30h_2_cell	Cells	ICL006-R	ICL006_mock_30h_2_RNA_ExpSam	RNA	ICL006-P	ICL006_Mock_30h_2_proteomics	Protein
ICL006	ICL006 Calu-3 Cells	Homo sapiens	ICL006_mock_30h_3_cell	Cells	ICL006-R	ICL006_mock_30h_3_RNA_ExpSam	RNA	ICL006-P	ICL006_Mock_30h_3_proteomics	Protein
ICL006	ICL006 Calu-3 Cells	Homo sapiens	ICL006_mock_36h_1_cell	Cells	ICL006-R	ICL006_mock_36h_1_RNA_ExpSam	RNA	ICL006-P	ICL006_Mock_36h_1_proteomics	Protein
ICL006	ICL006 Calu-3 Cells	Homo sapiens	ICL006_mock_36h_2_cell	Cells	ICL006-R	ICL006_mock_36h_2_RNA_ExpSam	RNA	ICL006-P	ICL006_Mock_36h_2_proteomics	Protein
ICL006	ICL006 Calu-3 Cells	Homo sapiens	ICL006_mock_36h_3_cell	Cells	ICL006-R	ICL006_mock_36h_3_RNA_ExpSam	RNA	ICL006-P	ICL006_Mock_36h_3_proteomics	Protein
ICL006	ICL006 Calu-3 Cells	Homo sapiens	ICL006_mock_3h_1_cell	Cells	ICL006-R	ICL006_mock_3h_1_RNA_ExpSam	RNA	ICL006-P	ICL006_Mock_3h_1_proteomics	Protein
ICL006	ICL006 Calu-3 Cells	Homo sapiens	ICL006_mock_3h_2_cell	Cells	ICL006-R	ICL006_mock_3h_2_RNA_ExpSam	RNA	ICL006-P	ICL006_Mock_3h_2_proteomics	Protein
ICL006	ICL006 Calu-3 Cells	Homo sapiens	ICL006_mock_3h_3_cell	Cells	ICL006-R	ICL006_mock_3h_3_RNA_ExpSam	RNA	ICL006-P	ICL006_Mock_3h_3_proteomics	Protein
ICL006	ICL006 Calu-3 Cells	Homo sapiens	ICL006_mock_48h_1_cell	Cells	ICL006-R	ICL006_mock_48h_1_RNA_ExpSam	RNA	ICL006-P	ICL006_Mock_48h_1_proteomics	Protein
ICL006	ICL006 Calu-3 Cells	Homo sapiens	ICL006_mock_48h_2_cell	Cells	ICL006-R	ICL006_mock_48h_2_RNA_ExpSam	RNA	ICL006-P	ICL006_Mock_48h_2_proteomics	Protein
ICL006	ICL006 Calu-3 Cells	Homo sapiens	ICL006_mock_48h_3_cell	Cells	ICL006-R	ICL006_mock_48h_3_RNA_ExpSam	RNA	ICL006-P	ICL006_Mock_48h_3_proteomics	Protein
ICL006	ICL006 Calu-3 Cells	Homo sapiens	ICL006_mock_7h_1_cell	Cells	ICL006-R	ICL006_mock_7h_1_RNA_ExpSam	RNA	ICL006-P	ICL006_Mock_7h_1_proteomics	Protein
ICL006	ICL006 Calu-3 Cells	Homo sapiens	ICL006_mock_7h_2_cell	Cells	ICL006-R	ICL006_mock_7h_2_RNA_ExpSam	RNA	ICL006-P	ICL006_Mock_7h_2_proteomics	Protein
ICL006	ICL006 Calu-3 Cells	Homo sapiens	ICL006_mock_7h_3_cell	Cells	ICL006-R	ICL006_mock_7h_3_RNA_ExpSam	RNA	ICL006-P	ICL006_Mock_7h_3_proteomics	Protein
ICL010	ICL010 Calu-3 cells	Homo sapiens	ICL010_NL_0h_1_Cells	Cells	ICL010-R	ICL010_NL_0h_1_array	RNA	ICL010-P	ICL010_NL_0h_1_Proteomics	Protein
ICL010	ICL010 Calu-3 cells	Homo sapiens	ICL010_NL_0h_2_Cells	Cells	ICL010-R	ICL010_NL_0h_2_array	RNA	ICL010-P	ICL010_NL_0h_2_Proteomics	Protein
ICL010	ICL010 Calu-3 cells	Homo sapiens	ICL010_NL_0h_3_Cells	Cells	ICL010-R	ICL010_NL_0h_3_array	RNA	ICL010-P	ICL010_NL_0h_3_Proteomics	Protein
ICL010	ICL010 Calu-3 cells	Homo sapiens	ICL010_NL_3h_1_Cells	Cells	ICL010-R	ICL010_NL_12h_1_array	RNA	ICL010-P	ICL010_NL_12h_1_Proteomics	Protein
ICL010	ICL010 Calu-3 cells	Homo sapiens	ICL010_NL_3h_2_Cells	Cells	ICL010-R	ICL010_NL_12h_2_array	RNA	ICL010-P	ICL010_NL_12h_2_Proteomics	Protein
ICL010	ICL010 Calu-3 cells	Homo sapiens	ICL010_NL_3h_3_Cells	Cells	ICL010-R	ICL010_NL_12h_3_array	RNA	ICL010-P	ICL010_NL_12h_3_Proteomics	Protein
ICL010	ICL010 Calu-3 cells	Homo sapiens	ICL010_NL_7h_1_Cells	Cells	ICL010-R	ICL010_NL_18h_1_array	RNA	ICL010-P	ICL010_NL_18h_1_Proteomics	Protein
ICL010	ICL010 Calu-3 cells	Homo sapiens	ICL010_NL_7h_2_Cells	Cells	ICL010-R	ICL010_NL_18h_2_array	RNA	ICL010-P	ICL010_NL_18h_2_Proteomics	Protein
ICL010	ICL010 Calu-3 cells	Homo sapiens	ICL010_NL_7h_3_Cells	Cells	ICL010-R	ICL010_NL_18h_3_array	RNA	ICL010-P	ICL010_NL_18h_3_Proteomics	Protein
ICL010	ICL010 Calu-3 cells	Homo sapiens	ICL010_NL_12h_1_Cells	Cells	ICL010-R	ICL010_NL_24h_1_array	RNA	ICL010-P	ICL010_NL_24h_1_Proteomics	Protein
ICL010	ICL010 Calu-3 cells	Homo sapiens	ICL010_NL_12h_2_Cells	Cells	ICL010-R	ICL010_NL_24h_2_array	RNA	ICL010-P	ICL010_NL_24h_2_Proteomics	Protein
ICL010	ICL010 Calu-3 cells	Homo sapiens	ICL010_NL_12h_3_Cells	Cells	ICL010-R	ICL010_NL_24h_3_array	RNA	ICL010-P	ICL010_NL_24h_3_Proteomics	Protein
ICL010	ICL010 Calu-3 cells	Homo sapiens	ICL010_NL_18h_1_Cells	Cells	ICL010-R	ICL010_NL_30h_1_array	RNA	ICL010-P	ICL010_NL_30h_1_Proteomics	Protein
ICL010	ICL010 Calu-3 cells	Homo sapiens	ICL010_NL_18h_2_Cells	Cells	ICL010-R	ICL010_NL_30h_2_array	RNA	ICL010-P	ICL010_NL_30h_2_Proteomics	Protein
ICL010	ICL010 Calu-3 cells	Homo sapiens	ICL010_NL_18h_3_Cells	Cells	ICL010-R	ICL010_NL_30h_3_array	RNA	ICL010-P	ICL010_NL_30h_3_Proteomics	Protein
ICL010	ICL010 Calu-3 cells	Homo sapiens	ICL010_NL_24h_1_Cells	Cells	ICL010-R	ICL010_NL_36h_1_array	RNA	ICL010-P	ICL010_NL_36h_1_Proteomics	Protein
ICL010	ICL010 Calu-3 cells	Homo sapiens	ICL010_NL_24h_2_Cells	Cells	ICL010-R	ICL010_NL_36h_2_array	RNA	ICL010-P	ICL010_NL_36h_2_Proteomics	Protein
ICL010	ICL010 Calu-3 cells	Homo sapiens	ICL010_NL_24h_3_Cells	Cells	ICL010-R	ICL010_NL_36h_3_array	RNA	ICL010-P	ICL010_NL_36h_3_Proteomics	Protein
ICL010	ICL010 Calu-3 cells	Homo sapiens	ICL010_NL_30h_1_Cells	Cells	ICL010-R	ICL010_NL_3h_1_array	RNA	ICL010-P	ICL010_NL_3h_1_Proteomics	Protein
ICL010	ICL010 Calu-3 cells	Homo sapiens	ICL010_NL_30h_2_Cells	Cells	ICL010-R	ICL010_NL_3h_2_array	RNA	ICL010-P	ICL010_NL_3h_2_Proteomics	Protein
ICL010	ICL010 Calu-3 cells	Homo sapiens	ICL010_NL_30h_3_Cells	Cells	ICL010-R	ICL010_NL_3h_3_array	RNA	ICL010-P	ICL010_NL_3h_3_Proteomics	Protein
ICL010	ICL010 Calu-3 cells	Homo sapiens	ICL010_NL_36h_1_Cells	Cells	ICL010-R	ICL010_NL_48h_1_array	RNA	ICL010-P	ICL010_NL_48h_1_Proteomics	Protein
ICL010	ICL010 Calu-3 cells	Homo sapiens	ICL010_NL_36h_2_Cells	Cells	ICL010-R	ICL010_NL_48h_2_array	RNA	ICL010-P	ICL010_NL_48h_2_Proteomics	Protein
ICL010	ICL010 Calu-3 cells	Homo sapiens	ICL010_NL_36h_3_Cells	Cells	ICL010-R	ICL010_NL_48h_3_array	RNA	ICL010-P	ICL010_NL_48h_3_Proteomics	Protein
ICL010	ICL010 Calu-3 cells	Homo sapiens	ICL010_NL_48h_1_Cells	Cells	ICL010-R	ICL010_NL_7h_1_array	RNA	ICL010-P	ICL010_NL_7h_1_Proteomics	Protein
ICL010	ICL010 Calu-3 cells	Homo sapiens	ICL010_NL_48h_2_Cells	Cells	ICL010-R	ICL010_NL_7h_2_array	RNA	ICL010-P	ICL010_NL_7h_2_Proteomics	Protein
ICL010	ICL010 Calu-3 cells	Homo sapiens	ICL010_NL_48h_3_Cells	Cells	ICL010-R	ICL010_NL_7h_3_array	RNA	ICL010-P	ICL010_NL_7h_3_Proteomics	Protein
ICL010	ICL010 Calu-3 cells	Homo sapiens	ICL010_mock_0h_1_Cells	Cells	ICL010-R	ICL010_mock_0h_1_array	RNA	ICL010-P	ICL010_mock_0h_1_Proteomics	Protein
ICL010	ICL010 Calu-3 cells	Homo sapiens	ICL010_mock_0h_2_Cells	Cells	ICL010-R	ICL010_mock_0h_2_array	RNA	ICL010-P	ICL010_mock_0h_2_Proteomics	Protein
ICL010	ICL010 Calu-3 cells	Homo sapiens	ICL010_mock_0h_3_Cells	Cells	ICL010-R	ICL010_mock_0h_3_array	RNA	ICL010-P	ICL010_mock_0h_3_Proteomics	Protein
ICL010	ICL010 Calu-3 cells	Homo sapiens	ICL010_mock_3h_1_Cells	Cells	ICL010-R	ICL010_mock_12h_1_array	RNA	ICL010-P	ICL010_mock_12h_1_Proteomics	Protein
ICL010	ICL010 Calu-3 cells	Homo sapiens	ICL010_mock_3h_2_Cells	Cells	ICL010-R	ICL010_mock_12h_2_array	RNA	ICL010-P	ICL010_mock_12h_2_Proteomics	Protein
ICL010	ICL010 Calu-3 cells	Homo sapiens	ICL010_mock_3h_3_Cells	Cells	ICL010-R	ICL010_mock_12h_3_array	RNA	ICL010-P	ICL010_mock_12h_3_Proteomics	Protein
ICL010	ICL010 Calu-3 cells	Homo sapiens	ICL010_mock_7h_1_Cells	Cells	ICL010-R	ICL010_mock_18h_1_array	RNA	ICL010-P	ICL010_mock_18h_1_Proteomics	Protein
ICL010	ICL010 Calu-3 cells	Homo sapiens	ICL010_mock_7h_2_Cells	Cells	ICL010-R	ICL010_mock_18h_2_array	RNA	ICL010-P	ICL010_mock_18h_2_Proteomics	Protein
ICL010	ICL010 Calu-3 cells	Homo sapiens	ICL010_mock_7h_3_Cells	Cells	ICL010-R	ICL010_mock_18h_3_array	RNA	ICL010-P	ICL010_mock_18h_3_Proteomics	Protein
ICL010	ICL010 Calu-3 cells	Homo sapiens	ICL010_mock_12h_1_Cells	Cells	ICL010-R	ICL010_mock_24h_1_array	RNA	ICL010-P	ICL010_mock_24h_1_Proteomics	Protein
ICL010	ICL010 Calu-3 cells	Homo sapiens	ICL010_mock_12h_2_Cells	Cells	ICL010-R	ICL010_mock_24h_2_array	RNA	ICL010-P	ICL010_mock_24h_2_Proteomics	Protein
ICL010	ICL010 Calu-3 cells	Homo sapiens	ICL010_mock_12h_3_Cells	Cells	ICL010-R	ICL010_mock_24h_3_array	RNA	ICL010-P	ICL010_mock_24h_3_Proteomics	Protein
ICL010	ICL010 Calu-3 cells	Homo sapiens	ICL010_mock_18h_1_Cells	Cells	ICL010-R	ICL010_mock_30h_1_array	RNA	ICL010-P	ICL010_mock_30h_1_Proteomics	Protein
ICL010	ICL010 Calu-3 cells	Homo sapiens	ICL010_mock_18h_2_Cells	Cells	ICL010-R	ICL010_mock_30h_2_array	RNA	ICL010-P	ICL010_mock_30h_2_Proteomics	Protein
ICL010	ICL010 Calu-3 cells	Homo sapiens	ICL010_mock_18h_3_Cells	Cells	ICL010-R	ICL010_mock_30h_3_array	RNA	ICL010-P	ICL010_mock_30h_3_Proteomics	Protein
ICL010	ICL010 Calu-3 cells	Homo sapiens	ICL010_mock_24h_1_Cells	Cells	ICL010-R	ICL010_mock_36h_1_array	RNA	ICL010-P	ICL010_mock_36h_1_Proteomics	Protein
ICL010	ICL010 Calu-3 cells	Homo sapiens	ICL010_mock_24h_2_Cells	Cells	ICL010-R	ICL010_mock_36h_2_array	RNA	ICL010-P	ICL010_mock_36h_2_Proteomics	Protein
ICL010	ICL010 Calu-3 cells	Homo sapiens	ICL010_mock_24h_3_Cells	Cells	ICL010-R	ICL010_mock_36h_3_array	RNA	ICL010-P	ICL010_mock_36h_3_Proteomics	Protein
ICL010	ICL010 Calu-3 cells	Homo sapiens	ICL010_mock_30h_1_Cells	Cells	ICL010-R	ICL010_mock_3h_1_array	RNA	ICL010-P	ICL010_mock_3h_1_Proteomics	Protein
ICL010	ICL010 Calu-3 cells	Homo sapiens	ICL010_mock_30h_2_Cells	Cells	ICL010-R	ICL010_mock_3h_2_array	RNA	ICL010-P	ICL010_mock_3h_2_Proteomics	Protein
ICL010	ICL010 Calu-3 cells	Homo sapiens	ICL010_mock_30h_3_Cells	Cells	ICL010-R	ICL010_mock_3h_3_array	RNA	ICL010-P	ICL010_mock_3h_3_Proteomics	Protein
ICL010	ICL010 Calu-3 cells	Homo sapiens	ICL010_mock_36h_1_Cells	Cells	ICL010-R	ICL010_mock_48h_1_array	RNA	ICL010-P	ICL010_mock_48h_1_Proteomics	Protein
ICL010	ICL010 Calu-3 cells	Homo sapiens	ICL010_mock_36h_2_Cells	Cells	ICL010-R	ICL010_mock_48h_2_array	RNA	ICL010-P	ICL010_mock_48h_2_Proteomics	Protein
ICL010	ICL010 Calu-3 cells	Homo sapiens	ICL010_mock_36h_3_Cells	Cells	ICL010-R	ICL010_mock_48h_3_array	RNA	ICL010-P	ICL010_mock_48h_3_Proteomics	Protein
ICL010	ICL010 Calu-3 cells	Homo sapiens	ICL010_mock_48h_1_Cells	Cells	ICL010-R	ICL010_mock_7h_1_array	RNA	ICL010-P	ICL010_mock_7h_1_Proteomics	Protein
ICL010	ICL010 Calu-3 cells	Homo sapiens	ICL010_mock_48h_2_Cells	Cells	ICL010-R	ICL010_mock_7h_2_array	RNA	ICL010-P	ICL010_mock_7h_2_Proteomics	Protein
ICL010	ICL010 Calu-3 cells	Homo sapiens	ICL010_mock_48h_3_Cells	Cells	ICL010-R	ICL010_mock_7h_3_array	RNA	ICL010-P	ICL010_mock_7h_3_Proteomics	Protein
ICL010	ICL010 Calu-3 cells	Homo sapiens	ICL010_Cal_0h_1_Cells	Cells	ICL010-R	ICL010_Cal_0h_1_array	RNA			
ICL010	ICL010 Calu-3 cells	Homo sapiens	ICL010_Cal_0h_2_Cells	Cells	ICL010-R	ICL010_Cal_0h_2_array	RNA			
ICL010	ICL010 Calu-3 cells	Homo sapiens	ICL010_Cal_0h_3_Cells	Cells	ICL010-R	ICL010_Cal_0h_3_array	RNA			
ICL010	ICL010 Calu-3 cells	Homo sapiens	ICL010_Cal_12h_1_Cells	Cells	ICL010-R	ICL010_Cal_12h_1_array	RNA			
ICL010	ICL010 Calu-3 cells	Homo sapiens	ICL010_Cal_12h_2_Cells	Cells	ICL010-R	ICL010_Cal_12h_2_array	RNA			
ICL010	ICL010 Calu-3 cells	Homo sapiens	ICL010_Cal_12h_3_Cells	Cells	ICL010-R	ICL010_Cal_12h_3_array	RNA			
ICL010	ICL010 Calu-3 cells	Homo sapiens	ICL010_Cal_24h_1_Cells	Cells	ICL010-R	ICL010_Cal_24h_1_array	RNA			
ICL010	ICL010 Calu-3 cells	Homo sapiens	ICL010_Cal_24h_2_Cells	Cells	ICL010-R	ICL010_Cal_24h_2_array	RNA			
ICL010	ICL010 Calu-3 cells	Homo sapiens	ICL010_Cal_24h_3_Cells	Cells	ICL010-R	ICL010_Cal_24h_3_array	RNA			
ICL010	ICL010 Calu-3 cells	Homo sapiens	ICL010_Cal_48h_1_Cells	Cells	ICL010-R	ICL010_Cal_48h_1_array	RNA			
ICL010	ICL010 Calu-3 cells	Homo sapiens	ICL010_Cal_48h_2_Cells	Cells	ICL010-R	ICL010_Cal_48h_2_array	RNA			
ICL010	ICL010 Calu-3 cells	Homo sapiens	ICL010_Cal_48h_3_Cells	Cells	ICL010-R	ICL010_Cal_48h_3_array	RNA			
ICL011	ICL011 Calu-3 Cells	Homo sapiens	ICL011_VN-PB2-627E_0h_1_Cells	Cells	ICL011-R	ICL011_VN-PB2-627E_0h_1_array	RNA	ICL011-P	ICL011_VN-PB2-627E_0h_1_proteomics	Protein
ICL011	ICL011 Calu-3 Cells	Homo sapiens	ICL011_VN-PB2-627E_0h_2_Cells	Cells	ICL011-R	ICL011_VN-PB2-627E_0h_2_array	RNA	ICL011-P	ICL011_VN-PB2-627E_0h_2_proteomics	Protein
ICL011	ICL011 Calu-3 Cells	Homo sapiens	ICL011_VN-PB2-627E_0h_3_Cells	Cells	ICL011-R	ICL011_VN-PB2-627E_0h_3_array	RNA	ICL011-P	ICL011_VN-PB2-627E_0h_3_proteomics	Protein
ICL011	ICL011 Calu-3 Cells	Homo sapiens	ICL011_VN-PB1-F2-del_0h_1_Cells	Cells	ICL011-R	ICL011_VN-PB1-F2-del_0h_1_array	RNA	ICL011-P	ICL011_VN-PB1-F2-del_0h_1_proteomics	Protein
ICL011	ICL011 Calu-3 Cells	Homo sapiens	ICL011_VN-PB1-F2-del_0h_2_Cells	Cells	ICL011-R	ICL011_VN-PB1-F2-del_0h_2_array	RNA	ICL011-P	ICL011_VN-PB1-F2-del_0h_2_proteomics	Protein
ICL011	ICL011 Calu-3 Cells	Homo sapiens	ICL011_VN-PB1-F2-del_0h_3_Cells	Cells	ICL011-R	ICL011_VN-PB1-F2-del_0h_3_array	RNA	ICL011-P	ICL011_VN-PB1-F2-del_0h_3_proteomics	Protein
ICL011	ICL011 Calu-3 Cells	Homo sapiens	ICL011_Mock_0h_1_Cells	Cells	ICL011-R	ICL011_mock_0h_1_array	RNA	ICL011-P	ICL011_mock_0h_1_proteomics	Protein
ICL011	ICL011 Calu-3 Cells	Homo sapiens	ICL011_Mock_0h_2_Cells	Cells	ICL011-R	ICL011_mock_0h_2_array	RNA	ICL011-P	ICL011_mock_0h_2_proteomics	Protein
ICL011	ICL011 Calu-3 Cells	Homo sapiens	ICL011_Mock_0h_3_Cells	Cells	ICL011-R	ICL011_mock_0h_3_array	RNA	ICL011-P	ICL011_mock_0h_3_proteomics	Protein
ICL011	ICL011 Calu-3 Cells	Homo sapiens	ICL011_VN-PB2-627E_3h_1_Cells	Cells	ICL011-R	ICL011_VN-PB2-627E_3h_1_array	RNA	ICL011-P	ICL011_VN-PB2-627E_3h_1_proteomics	Protein
ICL011	ICL011 Calu-3 Cells	Homo sapiens	ICL011_VN-PB2-627E_3h_2_Cells	Cells	ICL011-R	ICL011_VN-PB2-627E_3h_2_array	RNA	ICL011-P	ICL011_VN-PB2-627E_3h_2_proteomics	Protein
ICL011	ICL011 Calu-3 Cells	Homo sapiens	ICL011_VN-PB2-627E_3h_3_Cells	Cells	ICL011-R	ICL011_VN-PB2-627E_3h_3_array	RNA	ICL011-P	ICL011_VN-PB2-627E_3h_3_proteomics	Protein
ICL011	ICL011 Calu-3 Cells	Homo sapiens	ICL011_VN-PB1-F2-del_3h_1_Cells	Cells	ICL011-R	ICL011_VN-PB1-F2-del_3h_1_array	RNA	ICL011-P	ICL011_VN-PB1-F2-del_3h_1_proteomics	Protein
ICL011	ICL011 Calu-3 Cells	Homo sapiens	ICL011_VN-PB1-F2-del_3h_2_Cells	Cells	ICL011-R	ICL011_VN-PB1-F2-del_3h_2_array	RNA	ICL011-P	ICL011_VN-PB1-F2-del_3h_2_proteomics	Protein
ICL011	ICL011 Calu-3 Cells	Homo sapiens	ICL011_VN-PB1-F2-del_3h_3_Cells	Cells	ICL011-R	ICL011_VN-PB1-F2-del_3h_3_array	RNA	ICL011-P	ICL011_VN-PB1-F2-del_3h_3_proteomics	Protein
ICL011	ICL011 Calu-3 Cells	Homo sapiens	ICL011_Mock_3h_1_Cells	Cells	ICL011-R	ICL011_mock_3h_1_array	RNA	ICL011-P	ICL011_mock_3h_1_proteomics	Protein
ICL011	ICL011 Calu-3 Cells	Homo sapiens	ICL011_Mock_3h_2_Cells	Cells	ICL011-R	ICL011_mock_3h_2_array	RNA	ICL011-P	ICL011_mock_3h_2_proteomics	Protein
ICL011	ICL011 Calu-3 Cells	Homo sapiens	ICL011_Mock_3h_3_Cells	Cells	ICL011-R	ICL011_mock_3h_3_array	RNA	ICL011-P	ICL011_mock_3h_3_proteomics	Protein
ICL011	ICL011 Calu-3 Cells	Homo sapiens	ICL011_VN-PB2-627E_7h_1_Cells	Cells	ICL011-R	ICL011_VN-PB2-627E_7h_1_array	RNA	ICL011-P	ICL011_VN-PB2-627E_7h_1_proteomics	Protein
ICL011	ICL011 Calu-3 Cells	Homo sapiens	ICL011_VN-PB2-627E_7h_2_Cells	Cells	ICL011-R	ICL011_VN-PB2-627E_7h_2_array	RNA	ICL011-P	ICL011_VN-PB2-627E_7h_2_proteomics	Protein
ICL011	ICL011 Calu-3 Cells	Homo sapiens	ICL011_VN-PB2-627E_7h_3_Cells	Cells	ICL011-R	ICL011_VN-PB2-627E_7h_3_array	RNA	ICL011-P	ICL011_VN-PB2-627E_7h_3_proteomics	Protein
ICL011	ICL011 Calu-3 Cells	Homo sapiens	ICL011_VN-PB1-F2-del_7h_1_Cells	Cells	ICL011-R	ICL011_VN-PB1-F2-del_7h_1_array	RNA	ICL011-P	ICL011_VN-PB1-F2-del_7h_1_proteomics	Protein
ICL011	ICL011 Calu-3 Cells	Homo sapiens	ICL011_VN-PB1-F2-del_7h_2_Cells	Cells	ICL011-R	ICL011_VN-PB1-F2-del_7h_2_array	RNA	ICL011-P	ICL011_VN-PB1-F2-del_7h_2_proteomics	Protein
ICL011	ICL011 Calu-3 Cells	Homo sapiens	ICL011_VN-PB1-F2-del_7h_3_Cells	Cells	ICL011-R	ICL011_VN-PB1-F2-del_7h_3_array	RNA	ICL011-P	ICL011_VN-PB1-F2-del_7h_3_proteomics	Protein
ICL011	ICL011 Calu-3 Cells	Homo sapiens	ICL011_VN1203_7h_1_Cells	Cells	ICL011-R	ICL011_VN1203_7h_1_array	RNA	ICL011-P	ICL011_VN1203_7h_1_proteomics	Protein
ICL011	ICL011 Calu-3 Cells	Homo sapiens	ICL011_VN1203_7h_2_Cells	Cells	ICL011-R	ICL011_VN1203_7h_2_array	RNA	ICL011-P	ICL011_VN1203_7h_2_proteomics	Protein
ICL011	ICL011 Calu-3 Cells	Homo sapiens	ICL011_VN1203_7h_3_Cells	Cells	ICL011-R	ICL011_VN1203_7h_3_array	RNA	ICL011-P	ICL011_VN1203_7h_3_proteomics	Protein
ICL011	ICL011 Calu-3 Cells	Homo sapiens	ICL011_Mock_7h_1_Cells	Cells	ICL011-R	ICL011_mock_7h_1_array	RNA	ICL011-P	ICL011_mock_7h_1_proteomics	Protein
ICL011	ICL011 Calu-3 Cells	Homo sapiens	ICL011_Mock_7h_2_Cells	Cells	ICL011-R	ICL011_mock_7h_2_array	RNA	ICL011-P	ICL011_mock_7h_2_proteomics	Protein
ICL011	ICL011 Calu-3 Cells	Homo sapiens	ICL011_Mock_7h_3_Cells	Cells	ICL011-R	ICL011_mock_7h_3_array	RNA	ICL011-P	ICL011_mock_7h_3_proteomics	Protein
ICL011	ICL011 Calu-3 Cells	Homo sapiens	ICL011_VN-PB2-627E_12h_1_Cells	Cells	ICL011-R	ICL011_VN-PB2-627E_12h_1_array	RNA	ICL011-P	ICL011_VN-PB2-627E_12h_1_proteomics	Protein
ICL011	ICL011 Calu-3 Cells	Homo sapiens	ICL011_VN-PB2-627E_12h_2_Cells	Cells	ICL011-R	ICL011_VN-PB2-627E_12h_2_array	RNA	ICL011-P	ICL011_VN-PB2-627E_12h_2_proteomics	Protein
ICL011	ICL011 Calu-3 Cells	Homo sapiens	ICL011_VN-PB2-627E_12h_3_Cells	Cells	ICL011-R	ICL011_VN-PB2-627E_12h_3_array	RNA	ICL011-P	ICL011_VN-PB2-627E_12h_3_proteomics	Protein
ICL011	ICL011 Calu-3 Cells	Homo sapiens	ICL011_VN-PB1-F2-del_12h_1_Cells	Cells	ICL011-R	ICL011_VN-PB1-F2-del_12h_1_array	RNA	ICL011-P	ICL011_VN-PB1-F2-del_12h_1_proteomics	Protein
ICL011	ICL011 Calu-3 Cells	Homo sapiens	ICL011_VN-PB1-F2-del_12h_2_Cells	Cells	ICL011-R	ICL011_VN-PB1-F2-del_12h_2_array	RNA	ICL011-P	ICL011_VN-PB1-F2-del_12h_2_proteomics	Protein
ICL011	ICL011 Calu-3 Cells	Homo sapiens	ICL011_VN-PB1-F2-del_12h_3_Cells	Cells	ICL011-R	ICL011_VN-PB1-F2-del_12h_3_array	RNA	ICL011-P	ICL011_VN-PB1-F2-del_12h_3_proteomics	Protein
ICL011	ICL011 Calu-3 Cells	Homo sapiens	ICL011_Mock_12h_1_Cells	Cells	ICL011-R	ICL011_mock_12h_1_array	RNA	ICL011-P	ICL011_mock_12h_1_proteomics	Protein
ICL011	ICL011 Calu-3 Cells	Homo sapiens	ICL011_Mock_12h_2_Cells	Cells	ICL011-R	ICL011_mock_12h_2_array	RNA	ICL011-P	ICL011_mock_12h_2_proteomics	Protein
ICL011	ICL011 Calu-3 Cells	Homo sapiens	ICL011_Mock_12h_3_Cells	Cells	ICL011-R	ICL011_mock_12h_3_array	RNA	ICL011-P	ICL011_mock_12h_3_proteomics	Protein
ICL011	ICL011 Calu-3 Cells	Homo sapiens	ICL011_VN-PB2-627E_18h_1_Cells	Cells	ICL011-R	ICL011_VN-PB2-627E_18h_1_array	RNA	ICL011-P	ICL011_VN-PB2-627E_18h_1_proteomics	Protein
ICL011	ICL011 Calu-3 Cells	Homo sapiens	ICL011_VN-PB2-627E_18h_2_Cells	Cells	ICL011-R	ICL011_VN-PB2-627E_18h_2_array	RNA	ICL011-P	ICL011_VN-PB2-627E_18h_2_proteomics	Protein
ICL011	ICL011 Calu-3 Cells	Homo sapiens	ICL011_VN-PB2-627E_18h_3_Cells	Cells	ICL011-R	ICL011_VN-PB2-627E_18h_3_array	RNA	ICL011-P	ICL011_VN-PB2-627E_18h_3_proteomics	Protein
ICL011	ICL011 Calu-3 Cells	Homo sapiens	ICL011_VN-PB1-F2-del_18h_1_Cells	Cells	ICL011-R	ICL011_VN-PB1-F2-del_18h_1_array	RNA	ICL011-P	ICL011_VN-PB1-F2-del_18h_1_proteomics	Protein
ICL011	ICL011 Calu-3 Cells	Homo sapiens	ICL011_VN-PB1-F2-del_18h_2_Cells	Cells	ICL011-R	ICL011_VN-PB1-F2-del_18h_2_array	RNA	ICL011-P	ICL011_VN-PB1-F2-del_18h_2_proteomics	Protein
ICL011	ICL011 Calu-3 Cells	Homo sapiens	ICL011_VN-PB1-F2-del_18h_3_Cells	Cells	ICL011-R	ICL011_VN-PB1-F2-del_18h_3_array	RNA	ICL011-P	ICL011_VN-PB1-F2-del_18h_3_proteomics	Protein
ICL011	ICL011 Calu-3 Cells	Homo sapiens	ICL011_Mock_18h_1_Cells	Cells	ICL011-R	ICL011_mock_18h_1_array	RNA	ICL011-P	ICL011_mock_18h_1_proteomics	Protein
ICL011	ICL011 Calu-3 Cells	Homo sapiens	ICL011_Mock_18h_2_Cells	Cells	ICL011-R	ICL011_mock_18h_2_array	RNA	ICL011-P	ICL011_mock_18h_2_proteomics	Protein
ICL011	ICL011 Calu-3 Cells	Homo sapiens	ICL011_Mock_18h_3_Cells	Cells	ICL011-R	ICL011_mock_18h_3_array	RNA	ICL011-P	ICL011_mock_18h_3_proteomics	Protein
ICL011	ICL011 Calu-3 Cells	Homo sapiens	ICL011_VN-PB2-627E_24h_1_Cells	Cells	ICL011-R	ICL011_VN-PB2-627E_24h_1_array	RNA	ICL011-P	ICL011_VN-PB2-627E_24h_1_proteomics	Protein
ICL011	ICL011 Calu-3 Cells	Homo sapiens	ICL011_VN-PB2-627E_24h_2_Cells	Cells	ICL011-R	ICL011_VN-PB2-627E_24h_2_array	RNA	ICL011-P	ICL011_VN-PB2-627E_24h_2_proteomics	Protein
ICL011	ICL011 Calu-3 Cells	Homo sapiens	ICL011_VN-PB2-627E_24h_3_Cells	Cells	ICL011-R	ICL011_VN-PB2-627E_24h_3_array	RNA	ICL011-P	ICL011_VN-PB2-627E_24h_3_proteomics	Protein
ICL011	ICL011 Calu-3 Cells	Homo sapiens	ICL011_VN-PB1-F2-del_24h_1_Cells	Cells	ICL011-R	ICL011_VN-PB1-F2-del_24h_1_array	RNA	ICL011-P	ICL011_VN-PB1-F2-del_24h_1_proteomics	Protein
ICL011	ICL011 Calu-3 Cells	Homo sapiens	ICL011_VN-PB1-F2-del_24h_2_Cells	Cells	ICL011-R	ICL011_VN-PB1-F2-del_24h_2_array	RNA	ICL011-P	ICL011_VN-PB1-F2-del_24h_2_proteomics	Protein
ICL011	ICL011 Calu-3 Cells	Homo sapiens	ICL011_VN-PB1-F2-del_24h_3_Cells	Cells	ICL011-R	ICL011_VN-PB1-F2-del_24h_3_array	RNA	ICL011-P	ICL011_VN-PB1-F2-del_24h_3_proteomics	Protein
ICL011	ICL011 Calu-3 Cells	Homo sapiens	ICL011_VN1203_24h_1_Cells	Cells	ICL011-R	ICL011_VN1203_24h_1_array	RNA	ICL011-P	ICL011_VN1203_24h_1_proteomics	Protein
ICL011	ICL011 Calu-3 Cells	Homo sapiens	ICL011_VN1203_24h_2_Cells	Cells	ICL011-R	ICL011_VN1203_24h_2_array	RNA	ICL011-P	ICL011_VN1203_24h_2_proteomics	Protein
ICL011	ICL011 Calu-3 Cells	Homo sapiens	ICL011_VN1203_24h_3_Cells	Cells	ICL011-R	ICL011_VN1203_24h_3_array	RNA	ICL011-P	ICL011_VN1203_24h_3_proteomics	Protein
ICL011	ICL011 Calu-3 Cells	Homo sapiens	ICL011_Mock_24h_1_Cells	Cells	ICL011-R	ICL011_mock_24h_1_array	RNA	ICL011-P	ICL011_mock_24h_1_proteomics	Protein
ICL011	ICL011 Calu-3 Cells	Homo sapiens	ICL011_Mock_24h_2_Cells	Cells	ICL011-R	ICL011_mock_24h_2_array	RNA	ICL011-P	ICL011_mock_24h_2_proteomics	Protein
ICL011	ICL011 Calu-3 Cells	Homo sapiens	ICL011_Mock_24h_3_Cells	Cells	ICL011-R	ICL011_mock_24h_3_array	RNA	ICL011-P	ICL011_mock_24h_3_proteomics	Protein
ICL012	ICL012 Calu-3 Cells	Homo sapiens	ICL012_NS1trunc_0h_1_Cells	Cells	ICL012-R	ICL012_NS1trunc_0h_1_array	RNA	ICL012-P	ICL012_NS1trunc_0h_1_proteomics	Protein
ICL012	ICL012 Calu-3 Cells	Homo sapiens	ICL012_NS1trunc_0h_2_Cells	Cells	ICL012-R	ICL012_NS1trunc_0h_2_array	RNA	ICL012-P	ICL012_NS1trunc_0h_2_proteomics	Protein
ICL012	ICL012 Calu-3 Cells	Homo sapiens	ICL012_NS1trunc_0h_3_Cells	Cells	ICL012-R	ICL012_NS1trunc_0h_3_array	RNA	ICL012-P	ICL012_NS1trunc_0h_3_proteomics	Protein
ICL012	ICL012 Calu-3 Cells	Homo sapiens	ICL012_Mock_0h_1_Cells	Cells	ICL012-R		RNA	ICL012-P	ICL012_mock_0h_1_proteomics	Protein
ICL012	ICL012 Calu-3 Cells	Homo sapiens	ICL012_Mock_0h_2_Cells	Cells	ICL012-R	ICL012_mock_0h_2_array	RNA	ICL012-P	ICL012_mock_0h_2_proteomics	Protein
ICL012	ICL012 Calu-3 Cells	Homo sapiens	ICL012_Mock_0h_3_Cells	Cells	ICL012-R	ICL012_mock_0h_3_array	RNA	ICL012-P	ICL012_mock_0h_3_proteomics	Protein
ICL012	ICL012 Calu-3 Cells	Homo sapiens	ICL012_NS1trunc_3h_1_Cells	Cells	ICL012-R	ICL012_NS1trunc_3h_1_array	RNA	ICL012-P	ICL012_NS1trunc_3h_1_proteomics	Protein
ICL012	ICL012 Calu-3 Cells	Homo sapiens	ICL012_NS1trunc_3h_2_Cells	Cells	ICL012-R	ICL012_NS1trunc_3h_2_array	RNA	ICL012-P	ICL012_NS1trunc_3h_2_proteomics	Protein
ICL012	ICL012 Calu-3 Cells	Homo sapiens	ICL012_NS1trunc_3h_3_Cells	Cells	ICL012-R	ICL012_NS1trunc_3h_3_array	RNA	ICL012-P	ICL012_NS1trunc_3h_3_proteomics	Protein
ICL012	ICL012 Calu-3 Cells	Homo sapiens	ICL012_Mock_3h_1_Cells	Cells	ICL012-R	ICL012_mock_3h_1_array	RNA	ICL012-P	ICL012_mock_3h_1_proteomics	Protein
ICL012	ICL012 Calu-3 Cells	Homo sapiens	ICL012_Mock_3h_2_Cells	Cells	ICL012-R	ICL012_mock_3h_2_array	RNA	ICL012-P	ICL012_mock_3h_2_proteomics	Protein
ICL012	ICL012 Calu-3 Cells	Homo sapiens	ICL012_Mock_3h_3_Cells	Cells	ICL012-R	ICL012_mock_3h_3_array	RNA	ICL012-P	ICL012_mock_3h_3_proteomics	Protein
ICL012	ICL012 Calu-3 Cells	Homo sapiens	ICL012_NS1trunc_7h_1_Cells	Cells	ICL012-R	ICL012_NS1trunc_7h_1_array	RNA	ICL012-P	ICL012_NS1trunc_7h_1_proteomics	Protein
ICL012	ICL012 Calu-3 Cells	Homo sapiens	ICL012_NS1trunc_7h_2_Cells	Cells	ICL012-R	ICL012_NS1trunc_7h_2_array	RNA	ICL012-P	ICL012_NS1trunc_7h_2_proteomics	Protein
ICL012	ICL012 Calu-3 Cells	Homo sapiens	ICL012_NS1trunc_7h_3_Cells	Cells	ICL012-R	ICL012_NS1trunc_7h_3_array	RNA	ICL012-P	ICL012_NS1trunc_7h_3_proteomics	Protein
ICL012	ICL012 Calu-3 Cells	Homo sapiens	ICL012_Mock_7h_1_Cells	Cells	ICL012-R	ICL012_mock_7h_1_array	RNA	ICL012-P	ICL012_mock_7h_1_proteomics	Protein
ICL012	ICL012 Calu-3 Cells	Homo sapiens	ICL012_Mock_7h_2_Cells	Cells	ICL012-R	ICL012_mock_7h_2_array	RNA	ICL012-P	ICL012_mock_7h_2_proteomics	Protein
ICL012	ICL012 Calu-3 Cells	Homo sapiens	ICL012_Mock_7h_3_Cells	Cells	ICL012-R	ICL012_mock_7h_3_array	RNA	ICL012-P	ICL012_mock_7h_3_proteomics	Protein
ICL012	ICL012 Calu-3 Cells	Homo sapiens	ICL012_VN1203_7h_1_Cells	Cells	ICL012-R	ICL012_VN1203_7h_1_array	RNA	ICL012-P	ICL012_VN1203_7h_1_proteomics	Protein
ICL012	ICL012 Calu-3 Cells	Homo sapiens	ICL012_VN1203_7h_2_Cells	Cells	ICL012-R	ICL012_VN1203_7h_2_array	RNA	ICL012-P	ICL012_VN1203_7h_2_proteomics	Protein
ICL012	ICL012 Calu-3 Cells	Homo sapiens	ICL012_VN1203_7h_3_Cells	Cells	ICL012-R	ICL012_VN1203_7h_3_array	RNA	ICL012-P	ICL012_VN1203_7h_3_proteomics	Protein
ICL012	ICL012 Calu-3 Cells	Homo sapiens	ICL012_NS1trunc_12h_1_Cells	Cells	ICL012-R	ICL012_NS1trunc_12h_1_array	RNA	ICL012-P	ICL012_NS1trunc_12h_1_proteomics	Protein
ICL012	ICL012 Calu-3 Cells	Homo sapiens	ICL012_NS1trunc_12h_2_Cells	Cells	ICL012-R	ICL012_NS1trunc_12h_2_array	RNA	ICL012-P	ICL012_NS1trunc_12h_2_proteomics	Protein
ICL012	ICL012 Calu-3 Cells	Homo sapiens	ICL012_NS1trunc_12h_3_Cells	Cells	ICL012-R	ICL012_NS1trunc_12h_3_array	RNA	ICL012-P	ICL012_NS1trunc_12h_3_proteomics	Protein
ICL012	ICL012 Calu-3 Cells	Homo sapiens	ICL012_Mock_12h_1_Cells	Cells	ICL012-R	ICL012_mock_12h_1_array	RNA	ICL012-P	ICL012_mock_12h_1_proteomics	Protein
ICL012	ICL012 Calu-3 Cells	Homo sapiens	ICL012_Mock_12h_2_Cells	Cells	ICL012-R	ICL012_mock_12h_2_array	RNA	ICL012-P	ICL012_mock_12h_2_proteomics	Protein
ICL012	ICL012 Calu-3 Cells	Homo sapiens	ICL012_Mock_12h_3_Cells	Cells	ICL012-R	ICL012_mock_12h_3_array	RNA	ICL012-P	ICL012_mock_12h_3_proteomics	Protein
ICL012	ICL012 Calu-3 Cells	Homo sapiens	ICL012_NS1trunc_18h_1_Cells	Cells	ICL012-R	ICL012_NS1trunc_18h_1_array	RNA	ICL012-P	ICL012_NS1trunc_18h_1_proteomics	Protein
ICL012	ICL012 Calu-3 Cells	Homo sapiens	ICL012_NS1trunc_18h_2_Cells	Cells	ICL012-R	ICL012_NS1trunc_18h_2_array	RNA	ICL012-P	ICL012_NS1trunc_18h_2_proteomics	Protein
ICL012	ICL012 Calu-3 Cells	Homo sapiens	ICL012_NS1trunc_18h_3_Cells	Cells	ICL012-R	ICL012_NS1trunc_18h_3_array	RNA	ICL012-P	ICL012_NS1trunc_18h_3_proteomics	Protein
ICL012	ICL012 Calu-3 Cells	Homo sapiens	ICL012_Mock_18h_1_Cells	Cells	ICL012-R	ICL012_mock_18h_1_array	RNA	ICL012-P	ICL012_mock_18h_1_proteomics	Protein
ICL012	ICL012 Calu-3 Cells	Homo sapiens	ICL012_Mock_18h_2_Cells	Cells	ICL012-R	ICL012_mock_18h_2_array	RNA	ICL012-P	ICL012_mock_18h_2_proteomics	Protein
ICL012	ICL012 Calu-3 Cells	Homo sapiens	ICL012_Mock_18h_3_Cells	Cells	ICL012-R	ICL012_mock_18h_3_array	RNA	ICL012-P	ICL012_mock_18h_3_proteomics	Protein
ICL012	ICL012 Calu-3 Cells	Homo sapiens	ICL012_NS1trunc_24h_1_Cells	Cells	ICL012-R	ICL012_NS1trunc_24h_1_array	RNA	ICL012-P	ICL012_NS1trunc_24h_1_proteomics	Protein
ICL012	ICL012 Calu-3 Cells	Homo sapiens	ICL012_NS1trunc_24h_2_Cells	Cells	ICL012-R	ICL012_NS1trunc_24h_2_array	RNA	ICL012-P	ICL012_NS1trunc_24h_2_proteomics	Protein
ICL012	ICL012 Calu-3 Cells	Homo sapiens	ICL012_NS1trunc_24h_3_Cells	Cells	ICL012-R	ICL012_NS1trunc_24h_3_array	RNA	ICL012-P	ICL012_NS1trunc_24h_3_proteomics	Protein
ICL012	ICL012 Calu-3 Cells	Homo sapiens	ICL012_Mock_24h_1_Cells	Cells	ICL012-R	ICL012_mock_24h_1_array	RNA	ICL012-P	ICL012_mock_24h_1_proteomics	Protein
ICL012	ICL012 Calu-3 Cells	Homo sapiens	ICL012_Mock_24h_2_Cells	Cells	ICL012-R	ICL012_mock_24h_2_array	RNA	ICL012-P	ICL012_mock_24h_2_proteomics	Protein
ICL012	ICL012 Calu-3 Cells	Homo sapiens	ICL012_Mock_24h_3_Cells	Cells	ICL012-R	ICL012_mock_24h_3_array	RNA	ICL012-P	ICL012_mock_24h_3_proteomics	Protein
ICL012	ICL012 Calu-3 Cells	Homo sapiens	ICL012_VN1203_24h_1_Cells	Cells	ICL012-R	ICL012_VN1203_24h_1_array	RNA	ICL012-P	ICL012_VN1203_24h_1_proteomics	Protein
ICL012	ICL012 Calu-3 Cells	Homo sapiens	ICL012_VN1203_24h_2_Cells	Cells	ICL012-R	ICL012_VN1203_24h_2_array	RNA	ICL012-P	ICL012_VN1203_24h_2_proteomics	Protein
ICL012	ICL012 Calu-3 Cells	Homo sapiens	ICL012_VN1203_24h_3_Cells	Cells	ICL012-R	ICL012_VN1203_24h_3_array	RNA	ICL012-P	ICL012_VN1203_24h_3_proteomics	Protein
IM001	IM001_Mock_1d_1	Mus musculus	IM001_Mock_D1_1_lung	Tissue	IM001-R	IM001_Mock_D1_1_RNA_ExpSam	RNA	IM001-P	IM001_Mock_1d_1_proteomics	Protein
IM001	IM001_Mock_1d_2	Mus musculus	IM001_Mock_D1_2_lung	Tissue	IM001-R	IM001_Mock_D1_2_RNA_ExpSam	RNA	IM001-P	IM001_Mock_1d_2_proteomics	Protein
IM001	IM001_Mock_1d_3	Mus musculus	IM001_Mock_D1_3_lung	Tissue	IM001-R	IM001_Mock_D1_3_RNA_ExpSam	RNA	IM001-P	IM001_Mock_1d_3_proteomics	Protein
IM001	IM001_Mock_2d_1	Mus musculus	IM001_Mock_D2_1_lung	Tissue	IM001-R	IM001_Mock_D2_1_RNA_ExpSam	RNA	IM001-P	IM001_Mock_2d_1_proteomics	Protein
IM001	IM001_Mock_2d_2	Mus musculus	IM001_Mock_D2_2_lung	Tissue	IM001-R	IM001_Mock_D2_2_RNA_ExpSam	RNA	IM001-P	IM001_Mock_2d_2_proteomics	Protein
IM001	IM001_Mock_2d_3	Mus musculus	IM001_Mock_D2_3_lung	Tissue	IM001-R	IM001_Mock_D2_3_RNA_ExpSam	RNA	IM001-P	IM001_Mock_2d_3_proteomics	Protein
IM001	IM001_Mock_4d_1	Mus musculus	IM001_Mock_D4_1_lung	Tissue	IM001-R	IM001_Mock_D4_1_RNA_ExpSam	RNA	IM001-P	IM001_Mock_4d_1_proteomics	Protein
IM001	IM001_Mock_4d_2	Mus musculus	IM001_Mock_D4_2_lung	Tissue	IM001-R	IM001_Mock_D4_2_RNA_ExpSam	RNA	IM001-P	IM001_Mock_4d_2_proteomics	Protein
IM001	IM001_Mock_4d_3	Mus musculus	IM001_Mock_D4_3_lung	Tissue	IM001-R	IM001_Mock_D4_3_RNA_ExpSam	RNA	IM001-P	IM001_Mock_4d_3_proteomics	Protein
IM001	IM001_Mock_7d_1	Mus musculus	IM001_Mock_D7_1_lung	Tissue	IM001-R	IM001_Mock_D7_1_RNA_ExpSam	RNA	IM001-P	IM001_Mock_7d_1_proteomics	Protein
IM001	IM001_Mock_7d_2	Mus musculus	IM001_Mock_D7_2_lung	Tissue	IM001-R	IM001_Mock_D7_2_RNA_ExpSam	RNA	IM001-P	IM001_Mock_7d_2_proteomics	Protein
IM001	IM001_Mock_7d_3	Mus musculus	IM001_Mock_D7_3_lung	Tissue	IM001-R	IM001_Mock_D7_3_RNA_ExpSam	RNA	IM001-P	IM001_Mock_7d_3_proteomics	Protein
IM001	IM001_VN1203_10^2pfu_D1_1	Mus musculus	IM001_VN1203_10^2pfu_D1_1_lung	Tissue	IM001-R	IM001_VN1203_10^2pfu_D1_1_RNA_ExpSam	RNA	IM001-P	IM001_VN1203_10^2_1d_1_proteomics	Protein
IM001	IM001_VN1203_10^2pfu_D1_2	Mus musculus	IM001_VN1203_10^2pfu_D1_2_lung	Tissue	IM001-R	IM001_VN1203_10^2pfu_D1_2_RNA_ExpSam	RNA	IM001-P	IM001_VN1203_10^2_1d_2_proteomics	Protein
IM001	IM001_VN1203_10^2pfu_D1_3	Mus musculus	IM001_VN1203_10^2pfu_D1_3_lung	Tissue	IM001-R	IM001_VN1203_10^2pfu_D1_3_RNA_ExpSam	RNA	IM001-P	IM001_VN1203_10^2_1d_3_proteomics	Protein
IM001	IM001_VN1203_10^2pfu_D1_4	Mus musculus	IM001_VN1203_10^2pfu_D1_4_lung	Tissue	IM001-R	IM001_VN1203_10^2pfu_D1_4_RNA_ExpSam	RNA	IM001-P	IM001_VN1203_10^2_1d_4_proteomics	Protein
IM001	IM001_VN1203_10^2pfu_D1_5	Mus musculus	IM001_VN1203_10^2pfu_D1_5_lung	Tissue	IM001-R	IM001_VN1203_10^2pfu_D1_5_RNA_ExpSam	RNA	IM001-P	IM001_VN1203_10^2_1d_5_proteomics	Protein
IM001	IM001_VN1203_10^2pfu_D2_1	Mus musculus	IM001_VN1203_10^2pfu_D2_1_lung	Tissue	IM001-R	IM001_VN1203_10^2pfu_D2_1_RNA_ExpSam	RNA	IM001-P	IM001_VN1203_10^2_2d_1_proteomics	Protein
IM001	IM001_VN1203_10^2pfu_D2_2	Mus musculus	IM001_VN1203_10^2pfu_D2_2_lung	Tissue	IM001-R	IM001_VN1203_10^2pfu_D2_2_RNA_ExpSam	RNA	IM001-P	IM001_VN1203_10^2_2d_2_proteomics	Protein
IM001	IM001_VN1203_10^2pfu_D2_3	Mus musculus	IM001_VN1203_10^2pfu_D2_3_lung	Tissue	IM001-R	IM001_VN1203_10^2pfu_D2_3_RNA_ExpSam	RNA	IM001-P	IM001_VN1203_10^2_2d_3_proteomics	Protein
IM001	IM001_VN1203_10^2pfu_D2_4	Mus musculus	IM001_VN1203_10^2pfu_D2_4_lung	Tissue	IM001-R	IM001_VN1203_10^2pfu_D2_4_RNA_ExpSam	RNA	IM001-P	IM001_VN1203_10^2_2d_4_proteomics	Protein
IM001	IM001_VN1203_10^2pfu_D2_5	Mus musculus	IM001_VN1203_10^2pfu_D2_5_lung	Tissue	IM001-R	IM001_VN1203_10^2pfu_D2_5_RNA_ExpSam	RNA	IM001-P	IM001_VN1203_10^2_2d_5_proteomics	Protein
IM001	IM001_VN1203_10^2pfu_D4_1	Mus musculus	IM001_VN1203_10^2pfu_D4_1_lung	Tissue	IM001-R	IM001_VN1203_10^2pfu_D4_1_RNA_ExpSam	RNA	IM001-P	IM001_VN1203_10^2_4d_1_proteomics	Protein
IM001	IM001_VN1203_10^2pfu_D4_2	Mus musculus	IM001_VN1203_10^2pfu_D4_2_lung	Tissue	IM001-R	IM001_VN1203_10^2pfu_D4_2_RNA_ExpSam	RNA	IM001-P	IM001_VN1203_10^2_4d_2_proteomics	Protein
IM001	IM001_VN1203_10^2pfu_D4_3	Mus musculus	IM001_VN1203_10^2pfu_D4_3_lung	Tissue	IM001-R	IM001_VN1203_10^2pfu_D4_3_RNA_ExpSam	RNA	IM001-P	IM001_VN1203_10^2_4d_3_proteomics	Protein
IM001	IM001_VN1203_10^2pfu_D4_4	Mus musculus	IM001_VN1203_10^2pfu_D4_4_lung	Tissue	IM001-R	IM001_VN1203_10^2pfu_D4_4_RNA_ExpSam	RNA	IM001-P	IM001_VN1203_10^2_4d_4_proteomics	Protein
IM001	IM001_VN1203_10^2pfu_D4_5	Mus musculus	IM001_VN1203_10^2pfu_D4_5_lung	Tissue	IM001-R	IM001_VN1203_10^2pfu_D4_5_RNA_ExpSam	RNA	IM001-P	IM001_VN1203_10^2_4d_5_proteomics	Protein
IM001	IM001_VN1203_10^2pfu_D7_1	Mus musculus	IM001_VN1203_10^2pfu_D7_1_lung	Tissue	IM001-R	IM001_VN1203_10^2pfu_D7_1_RNA_ExpSam	RNA	IM001-P	IM001_VN1203_10^2_7d_1_proteomics	Protein
IM001	IM001_VN1203_10^2pfu_D7_2	Mus musculus	IM001_VN1203_10^2pfu_D7_2_lung	Tissue	IM001-R	IM001_VN1203_10^2pfu_D7_2_RNA_ExpSam	RNA	IM001-P	IM001_VN1203_10^2_7d_2_proteomics	Protein
IM001	IM001_VN1203_10^2pfu_D7_3	Mus musculus	IM001_VN1203_10^2pfu_D7_3_lung	Tissue	IM001-R	IM001_VN1203_10^2pfu_D7_3_RNA_ExpSam	RNA	IM001-P	IM001_VN1203_10^2_7d_3_proteomics	Protein
IM001	IM001_VN1203_10^2pfu_D7_4	Mus musculus	IM001_VN1203_10^2pfu_D7_4_lung	Tissue	IM001-R	IM001_VN1203_10^2pfu_D7_4_RNA_ExpSam	RNA	IM001-P	IM001_VN1203_10^2_7d_4_proteomics	Protein
IM001	IM001_VN1203_10^3pfu_D1_1	Mus musculus	IM001_VN1203_10^3pfu_D1_1_lung	Tissue	IM001-R	IM001_VN1203_10^3pfu_D1_1_RNA_ExpSam	RNA	IM001-P	IM001_VN1203_10^3_1d_1_proteomics	Protein
IM001	IM001_VN1203_10^3pfu_D1_2	Mus musculus	IM001_VN1203_10^3pfu_D1_2_lung	Tissue	IM001-R	IM001_VN1203_10^3pfu_D1_2_RNA_ExpSam	RNA	IM001-P	IM001_VN1203_10^3_1d_2_proteomics	Protein
IM001	IM001_VN1203_10^3pfu_D1_3	Mus musculus	IM001_VN1203_10^3pfu_D1_3_lung	Tissue	IM001-R	IM001_VN1203_10^3pfu_D1_3_RNA_ExpSam	RNA	IM001-P	IM001_VN1203_10^3_1d_3_proteomics	Protein
IM001	IM001_VN1203_10^3pfu_D1_4	Mus musculus	IM001_VN1203_10^3pfu_D1_4_lung	Tissue	IM001-R	IM001_VN1203_10^3pfu_D1_4_RNA_ExpSam	RNA	IM001-P	IM001_VN1203_10^3_1d_4_proteomics	Protein
IM001	IM001_VN1203_10^3pfu_D1_5	Mus musculus	IM001_VN1203_10^3pfu_D1_5_lung	Tissue	IM001-R	IM001_VN1203_10^3pfu_D1_5_RNA_ExpSam	RNA	IM001-P	IM001_VN1203_10^3_1d_5_proteomics	Protein
IM001	IM001_VN1203_10^3pfu_D2_1	Mus musculus	IM001_VN1203_10^3pfu_D2_1_lung	Tissue	IM001-R	IM001_VN1203_10^3pfu_D2_1_RNA_ExpSam	RNA	IM001-P	IM001_VN1203_10^3_2d_1_proteomics	Protein
IM001	IM001_VN1203_10^3pfu_D2_2	Mus musculus	IM001_VN1203_10^3pfu_D2_2_lung	Tissue	IM001-R	IM001_VN1203_10^3pfu_D2_2_RNA_ExpSam	RNA	IM001-P	IM001_VN1203_10^3_2d_2_proteomics	Protein
IM001	IM001_VN1203_10^3pfu_D2_3	Mus musculus	IM001_VN1203_10^3pfu_D2_3_lung	Tissue	IM001-R	IM001_VN1203_10^3pfu_D2_3_RNA_ExpSam	RNA	IM001-P	IM001_VN1203_10^3_2d_3_proteomics	Protein
IM001	IM001_VN1203_10^3pfu_D2_4	Mus musculus	IM001_VN1203_10^3pfu_D2_4_lung	Tissue	IM001-R	IM001_VN1203_10^3pfu_D2_4_RNA_ExpSam	RNA	IM001-P	IM001_VN1203_10^3_2d_4_proteomics	Protein
IM001	IM001_VN1203_10^3pfu_D2_5	Mus musculus	IM001_VN1203_10^3pfu_D2_5_lung	Tissue	IM001-R	IM001_VN1203_10^3pfu_D2_5_RNA_ExpSam	RNA	IM001-P	IM001_VN1203_10^3_2d_5_proteomics	Protein
IM001	IM001_VN1203_10^3pfu_D4_1	Mus musculus	IM001_VN1203_10^3pfu_D4_1_lung	Tissue	IM001-R	IM001_VN1203_10^3pfu_D4_1_RNA_ExpSam	RNA	IM001-P	IM001_VN1203_10^3_4d_1_proteomics	Protein
IM001	IM001_VN1203_10^3pfu_D4_2	Mus musculus	IM001_VN1203_10^3pfu_D4_2_lung	Tissue	IM001-R	IM001_VN1203_10^3pfu_D4_2_RNA_ExpSam	RNA	IM001-P	IM001_VN1203_10^3_4d_2_proteomics	Protein
IM001	IM001_VN1203_10^3pfu_D4_3	Mus musculus	IM001_VN1203_10^3pfu_D4_3_lung	Tissue	IM001-R	IM001_VN1203_10^3pfu_D4_3_RNA_ExpSam	RNA	IM001-P	IM001_VN1203_10^3_4d_3_proteomics	Protein
IM001	IM001_VN1203_10^3pfu_D4_4	Mus musculus	IM001_VN1203_10^3pfu_D4_4_lung	Tissue	IM001-R	IM001_VN1203_10^3pfu_D4_4_RNA_ExpSam	RNA	IM001-P	IM001_VN1203_10^3_4d_4_proteomics	Protein
IM001	IM001_VN1203_10^3pfu_D4_5	Mus musculus	IM001_VN1203_10^3pfu_D4_5_lung	Tissue	IM001-R	IM001_VN1203_10^3pfu_D4_5_RNA_ExpSam	RNA	IM001-P	IM001_VN1203_10^3_4d_5_proteomics	Protein
IM001	IM001_VN1203_10^3pfu_D7_1	Mus musculus	IM001_VN1203_10^3pfu_D7_1_lung	Tissue	IM001-R	IM001_VN1203_10^3pfu_D7_1_RNA_ExpSam	RNA	IM001-P	IM001_VN1203_10^3_7d_1_proteomics	Protein
IM001	IM001_VN1203_10^3pfu_D7_2	Mus musculus	IM001_VN1203_10^3pfu_D7_2_lung	Tissue	IM001-R	IM001_VN1203_10^3pfu_D7_2_RNA_ExpSam	RNA	IM001-P	IM001_VN1203_10^3_7d_2_proteomics	Protein
IM001	IM001_VN1203_10^3pfu_D7_3	Mus musculus	IM001_VN1203_10^3pfu_D7_3_lung	Tissue	IM001-R	IM001_VN1203_10^3pfu_D7_3_RNA_ExpSam	RNA	IM001-P	IM001_VN1203_10^3_7d_3_proteomics	Protein
IM001	IM001_VN1203_10^4pfu_D1_1	Mus musculus	IM001_VN1203_10^4pfu_D1_1_lung	Tissue	IM001-R	IM001_VN1203_10^4pfu_D1_1_RNA_ExpSam	RNA	IM001-P	IM001_VN1203_10^4_1d_1_proteomics	Protein
IM001	IM001_VN1203_10^4pfu_D1_2	Mus musculus	IM001_VN1203_10^4pfu_D1_2_lung	Tissue	IM001-R	IM001_VN1203_10^4pfu_D1_2_RNA_ExpSam	RNA	IM001-P	IM001_VN1203_10^4_1d_2_proteomics	Protein
IM001	IM001_VN1203_10^4pfu_D1_3	Mus musculus	IM001_VN1203_10^4pfu_D1_3_lung	Tissue	IM001-R	IM001_VN1203_10^4pfu_D1_3_RNA_ExpSam	RNA	IM001-P	IM001_VN1203_10^4_1d_3_proteomics	Protein
IM001	IM001_VN1203_10^4pfu_D1_4	Mus musculus	IM001_VN1203_10^4pfu_D1_4_lung	Tissue	IM001-R	IM001_VN1203_10^4pfu_D1_4_RNA_ExpSam	RNA	IM001-P	IM001_VN1203_10^4_1d_4_proteomics	Protein
IM001	IM001_VN1203_10^4pfu_D1_5	Mus musculus	IM001_VN1203_10^4pfu_D1_5_lung	Tissue	IM001-R	IM001_VN1203_10^4pfu_D1_5_RNA_ExpSam	RNA	IM001-P	IM001_VN1203_10^4_1d_5_proteomics	Protein
IM001	IM001_VN1203_10^4pfu_D2_1	Mus musculus	IM001_VN1203_10^4pfu_D2_1_lung	Tissue	IM001-R	IM001_VN1203_10^4pfu_D2_1_RNA_ExpSam	RNA	IM001-P	IM001_VN1203_10^4_2d_1_proteomics	Protein
IM001	IM001_VN1203_10^4pfu_D2_2	Mus musculus	IM001_VN1203_10^4pfu_D2_2_lung	Tissue	IM001-R	IM001_VN1203_10^4pfu_D2_2_RNA_ExpSam	RNA	IM001-P	IM001_VN1203_10^4_2d_2_proteomics	Protein
IM001	IM001_VN1203_10^4pfu_D2_3	Mus musculus	IM001_VN1203_10^4pfu_D2_3_lung	Tissue	IM001-R	IM001_VN1203_10^4pfu_D2_3_RNA_ExpSam	RNA	IM001-P	IM001_VN1203_10^4_2d_3_proteomics	Protein
IM001	IM001_VN1203_10^4pfu_D2_4	Mus musculus	IM001_VN1203_10^4pfu_D2_4_lung	Tissue	IM001-R	IM001_VN1203_10^4pfu_D2_4_RNA_ExpSam	RNA	IM001-P	IM001_VN1203_10^4_2d_4_proteomics	Protein
IM001	IM001_VN1203_10^4pfu_D2_5	Mus musculus	IM001_VN1203_10^4pfu_D2_5_lung	Tissue	IM001-R	IM001_VN1203_10^4pfu_D2_5_RNA_ExpSam	RNA	IM001-P	IM001_VN1203_10^4_2d_5_proteomics	Protein
IM001	IM001_VN1203_10^4pfu_D4_1	Mus musculus	IM001_VN1203_10^4pfu_D4_1_lung	Tissue	IM001-R	IM001_VN1203_10^4pfu_D4_1_RNA_ExpSam	RNA	IM001-P	IM001_VN1203_10^4_4d_1_proteomics	Protein
IM001	IM001_VN1203_10^4pfu_D4_2	Mus musculus	IM001_VN1203_10^4pfu_D4_2_lung	Tissue	IM001-R	IM001_VN1203_10^4pfu_D4_2_RNA_ExpSam	RNA	IM001-P	IM001_VN1203_10^4_4d_2_proteomics	Protein
IM001	IM001_VN1203_10^4pfu_D4_3	Mus musculus	IM001_VN1203_10^4pfu_D4_3_lung	Tissue	IM001-R	IM001_VN1203_10^4pfu_D4_3_RNA_ExpSam	RNA	IM001-P	IM001_VN1203_10^4_4d_3_proteomics	Protein
IM001	IM001_VN1203_10^4pfu_D4_4	Mus musculus	IM001_VN1203_10^4pfu_D4_4_lung	Tissue	IM001-R	IM001_VN1203_10^4pfu_D4_4_RNA_ExpSam	RNA	IM001-P	IM001_VN1203_10^4_4d_4_proteomics	Protein
IM001	IM001_VN1203_10^4pfu_D4_5	Mus musculus	IM001_VN1203_10^4pfu_D4_5_lung	Tissue	IM001-R	IM001_VN1203_10^4pfu_D4_5_RNA_ExpSam	RNA	IM001-P	IM001_VN1203_10^4_4d_5_proteomics	Protein
IM002	IM002_Brisbane_10^6pfu_D1_2	Mus musculus	IM002_Brisbane_10^6pfu_D1_2_Lung	Tissue	IM002-R	IM002_Brisbane_10^6pfu_D1_2_array	RNA			
IM002	IM002_Brisbane_10^6pfu_D1_3	Mus musculus	IM002_Brisbane_10^6pfu_D1_3_Lung	Tissue	IM002-R	IM002_Brisbane_10^6pfu_D1_3_array	RNA			
IM002	IM002_Brisbane_10^6pfu_D3_2	Mus musculus	IM002_Brisbane_10^6pfu_D3_2_Lung	Tissue	IM002-R	IM002_Brisbane_10^6pfu_D3_2_array	RNA			
IM002	IM002_Brisbane_10^6pfu_D3_3	Mus musculus	IM002_Brisbane_10^6pfu_D3_3_Lung	Tissue	IM002-R	IM002_Brisbane_10^6pfu_D3_3_array	RNA			
IM002	IM002_Brisbane_10^6pfu_D5_1	Mus musculus	IM002_Brisbane_10^6pfu_D5_1_Lung	Tissue	IM002-R	IM002_Brisbane_10^6pfu_D5_1_array	RNA			
IM002	IM002_Brisbane_10^6pfu_D5_2	Mus musculus	IM002_Brisbane_10^6pfu_D5_2_Lung	Tissue	IM002-R	IM002_Brisbane_10^6pfu_D5_2_array	RNA			
IM002	IM002_Brisbane_10^6pfu_D5_3	Mus musculus	IM002_Brisbane_10^6pfu_D5_3_Lung	Tissue	IM002-R	IM002_Brisbane_10^6pfu_D5_3_array	RNA			
IM002	IM002_Mex_10^6pfu_D1_1	Mus musculus	IM002_Mex_10^6pfu_D1_1_Lung	Tissue	IM002-R	IM002_Mex_10^6pfu_D1_1_array	RNA			
IM002	IM002_Mex_10^6pfu_D1_2	Mus musculus	IM002_Mex_10^6pfu_D1_2_Lung	Tissue	IM002-R	IM002_Mex_10^6pfu_D1_2_array	RNA			
IM002	IM002_Mex_10^6pfu_D1_3	Mus musculus	IM002_Mex_10^6pfu_D1_3_Lung	Tissue	IM002-R	IM002_Mex_10^6pfu_D1_3_array	RNA			
IM002	IM002_Mex_10^6pfu_D3_1	Mus musculus	IM002_Mex_10^6pfu_D3_1_Lung	Tissue	IM002-R	IM002_Mex_10^6pfu_D3_1_array	RNA			
IM002	IM002_Mex_10^6pfu_D3_2	Mus musculus	IM002_Mex_10^6pfu_D3_2_Lung	Tissue	IM002-R	IM002_Mex_10^6pfu_D3_2_array	RNA			
IM002	IM002_Mex_10^6pfu_D3_3	Mus musculus	IM002_Mex_10^6pfu_D3_3_Lung	Tissue	IM002-R	IM002_Mex_10^6pfu_D3_3_array	RNA			
IM002	IM002_Mex_10^6pfu_D5_1	Mus musculus	IM002_Mex_10^6pfu_D5_1_Lung	Tissue	IM002-R	IM002_Mex_10^6pfu_D5_1_array	RNA			
IM002	IM002_Mex_10^6pfu_D5_2	Mus musculus	IM002_Mex_10^6pfu_D5_2_Lung	Tissue	IM002-R	IM002_Mex_10^6pfu_D5_2_array	RNA			
IM002	IM002_Mex_10^6pfu_D5_3	Mus musculus	IM002_Mex_10^6pfu_D5_3_Lung	Tissue	IM002-R	IM002_Mex_10^6pfu_D5_3_array	RNA			
IM002	IM002_Mock_D1_1	Mus musculus	IM002_Mock_D1_1_Lung	Tissue	IM002-R	IM002_Mock_D1_1_array	RNA			
IM002	IM002_Mock_D1_2	Mus musculus	IM002_Mock_D1_2_Lung	Tissue	IM002-R	IM002_Mock_D1_2_array	RNA			
IM002	IM002_Mock_D3_1	Mus musculus	IM002_Mock_D3_1_Lung	Tissue	IM002-R	IM002_Mock_D3_1_array	RNA			
IM002	IM002_Mock_D3_2	Mus musculus	IM002_Mock_D3_2_Lung	Tissue	IM002-R	IM002_Mock_D3_2_array	RNA			
IM002	IM002_Mock_D5_2	Mus musculus	IM002_Mock_D5_2_Lung	Tissue	IM002-R	IM002_Mock_D5_2_array	RNA			
IM002	IM002_NJ_10^6pfu_D1_1	Mus musculus	IM002_NJ_10^6pfu_D1_1_Lung	Tissue	IM002-R	IM002_NJ_10^6pfu_D1_1_array	RNA			
IM002	IM002_NJ_10^6pfu_D1_3	Mus musculus	IM002_NJ_10^6pfu_D1_3_Lung	Tissue	IM002-R	IM002_NJ_10^6pfu_D1_3_array	RNA			
IM002	IM002_NJ_10^6pfu_D3_1	Mus musculus	IM002_NJ_10^6pfu_D3_1_Lung	Tissue	IM002-R	IM002_NJ_10^6pfu_D3_1_array	RNA			
IM002	IM002_NJ_10^6pfu_D3_2	Mus musculus	IM002_NJ_10^6pfu_D3_2_Lung	Tissue	IM002-R	IM002_NJ_10^6pfu_D3_2_array	RNA			
IM002	IM002_NJ_10^6pfu_D3_3	Mus musculus	IM002_NJ_10^6pfu_D3_3_Lung	Tissue	IM002-R	IM002_NJ_10^6pfu_D3_3_array	RNA			
IM002	IM002_NJ_10^6pfu_D5_2	Mus musculus	IM002_NJ_10^6pfu_D5_2_Lung	Tissue	IM002-R	IM002_NJ_10^6pfu_D5_2_array	RNA			
IM002	IM002_NJ_10^6pfu_D5_3	Mus musculus	IM002_NJ_10^6pfu_D5_3_Lung	Tissue	IM002-R	IM002_NJ_10^6pfu_D5_3_array	RNA			
IM002	IM002_r1918_10^6pfu_D1_1	Mus musculus	IM002_r1918_10^6pfu_D1_1_Lung	Tissue	IM002-R	IM002_r1918_10^6pfu_D1_1_array	RNA			
IM002	IM002_r1918_10^6pfu_D1_2	Mus musculus	IM002_r1918_10^6pfu_D1_2_Lung	Tissue	IM002-R	IM002_r1918_10^6pfu_D1_2_array	RNA			
IM002	IM002_r1918_10^6pfu_D1_3	Mus musculus	IM002_r1918_10^6pfu_D1_3_Lung	Tissue	IM002-R	IM002_r1918_10^6pfu_D1_3_array	RNA			
IM002	IM002_r1918_10^6pfu_D3_1	Mus musculus	IM002_r1918_10^6pfu_D3_1_Lung	Tissue	IM002-R	IM002_r1918_10^6pfu_D3_1_array	RNA			
IM002	IM002_r1918_10^6pfu_D3_2	Mus musculus	IM002_r1918_10^6pfu_D3_2_Lung	Tissue	IM002-R	IM002_r1918_10^6pfu_D3_2_array	RNA			
IM002	IM002_r1918_10^6pfu_D3_3	Mus musculus	IM002_r1918_10^6pfu_D3_3_Lung	Tissue	IM002-R	IM002_r1918_10^6pfu_D3_3_array	RNA			
IM002	IM002_r1918_10^6pfu_D5_1	Mus musculus	IM002_r1918_10^6pfu_D5_1_Lung	Tissue	IM002-R	IM002_r1918_10^6pfu_D5_1_array	RNA			
IM002	IM002_r1918_10^6pfu_D5_2	Mus musculus	IM002_r1918_10^6pfu_D5_2_Lung	Tissue	IM002-R	IM002_r1918_10^6pfu_D5_2_array	RNA			
IM002	IM002_r1918_10^6pfu_D5_3	Mus musculus	IM002_r1918_10^6pfu_D5_3_Lung	Tissue	IM002-R	IM002_r1918_10^6pfu_D5_3_array	RNA			
IM004	IM004_HAavirulent_10^4pfu_1d_1	Mus musculus	IM004_HAavirulent_10^4pfu_1d_1_lung	Tissue	IM004-R	IM004_HAavirulent_10^4pfu_1d_1_RNA_ExpSam	RNA	IM004-P	IM004_HAavir_10^4_1d_1_proteomics	Protein
IM004	IM004_HAavirulent_10^4pfu_1d_2	Mus musculus	IM004_HAavirulent_10^4pfu_1d_2_lung	Tissue	IM004-R	IM004_HAavirulent_10^4pfu_1d_2_RNA_ExpSam	RNA	IM004-P	IM004_HAavir_10^4_1d_2_proteomics	Protein
IM004	IM004_HAavirulent_10^4pfu_1d_3	Mus musculus	IM004_HAavirulent_10^4pfu_1d_3_lung	Tissue	IM004-R	IM004_HAavirulent_10^4pfu_1d_3_RNA_ExpSam	RNA	IM004-P	IM004_HAavir_10^4_1d_3_proteomics	Protein
IM004	IM004_HAavirulent_10^4pfu_1d_4	Mus musculus	IM004_HAavirulent_10^4pfu_1d_4_lung	Tissue	IM004-R	IM004_HAavirulent_10^4pfu_1d_4_RNA_ExpSam	RNA	IM004-P	IM004_HAavir_10^4_1d_4_proteomics	Protein
IM004	IM004_HAavirulent_10^4pfu_1d_5	Mus musculus	IM004_HAavirulent_10^4pfu_1d_5_lung	Tissue	IM004-R	IM004_HAavirulent_10^4pfu_1d_5_RNA_ExpSam	RNA	IM004-P	IM004_HAavir_10^4_1d_5_proteomics	Protein
IM004	IM004_HAavirulent_10^4pfu_2d_1	Mus musculus	IM004_HAavirulent_10^4pfu_2d_1_lung	Tissue	IM004-R	IM004_HAavirulent_10^4pfu_2d_1_RNA_ExpSam	RNA	IM004-P	IM004_HAavir_10^4_2d_1_proteomics	Protein
IM004	IM004_HAavirulent_10^4pfu_2d_2	Mus musculus	IM004_HAavirulent_10^4pfu_2d_2_lung	Tissue	IM004-R	IM004_HAavirulent_10^4pfu_2d_2_RNA_ExpSam	RNA	IM004-P	IM004_HAavir_10^4_2d_2_proteomics	Protein
IM004	IM004_HAavirulent_10^4pfu_2d_3	Mus musculus	IM004_HAavirulent_10^4pfu_2d_3_lung	Tissue	IM004-R	IM004_HAavirulent_10^4pfu_2d_3_RNA_ExpSam	RNA	IM004-P	IM004_HAavir_10^4_2d_3_proteomics	Protein
IM004	IM004_HAavirulent_10^4pfu_2d_4	Mus musculus	IM004_HAavirulent_10^4pfu_2d_4_lung	Tissue	IM004-R	IM004_HAavirulent_10^4pfu_2d_4_RNA_ExpSam	RNA	IM004-P	IM004_HAavir_10^4_2d_4_proteomics	Protein
IM004	IM004_HAavirulent_10^4pfu_2d_5	Mus musculus	IM004_HAavirulent_10^4pfu_2d_5_lung	Tissue	IM004-R	IM004_HAavirulent_10^4pfu_2d_5_RNA_ExpSam	RNA	IM004-P	IM004_HAavir_10^4_2d_5_proteomics	Protein
IM004	IM004_HAavirulent_10^4pfu_4d_1	Mus musculus	IM004_HAavirulent_10^4pfu_4d_1_lung	Tissue	IM004-R	IM004_HAavirulent_10^4pfu_4d_1_RNA_ExpSam	RNA	IM004-P	IM004_HAavir_10^4_4d_1_proteomics	Protein
IM004	IM004_HAavirulent_10^4pfu_4d_2	Mus musculus	IM004_HAavirulent_10^4pfu_4d_2_lung	Tissue	IM004-R	IM004_HAavirulent_10^4pfu_4d_2_RNA_ExpSam	RNA	IM004-P	IM004_HAavir_10^4_4d_2_proteomics	Protein
IM004	IM004_HAavirulent_10^4pfu_4d_3	Mus musculus	IM004_HAavirulent_10^4pfu_4d_3_lung	Tissue	IM004-R	IM004_HAavirulent_10^4pfu_4d_3_RNA_ExpSam	RNA	IM004-P	IM004_HAavir_10^4_4d_3_proteomics	Protein
IM004	IM004_HAavirulent_10^4pfu_4d_4	Mus musculus	IM004_HAavirulent_10^4pfu_4d_4_lung	Tissue	IM004-R	IM004_HAavirulent_10^4pfu_4d_4_RNA_ExpSam	RNA	IM004-P	IM004_HAavir_10^4_4d_4_proteomics	Protein
IM004	IM004_HAavirulent_10^4pfu_4d_5	Mus musculus	IM004_HAavirulent_10^4pfu_4d_5_lung	Tissue	IM004-R	IM004_HAavirulent_10^4pfu_4d_5_RNA_ExpSam	RNA	IM004-P	IM004_HAavir_10^4_4d_5_proteomics	Protein
IM004	IM004_HAavirulent_10^4pfu_7d_1	Mus musculus	IM004_HAavirulent_10^4pfu_7d_1_lung	Tissue	IM004-R	IM004_HAavirulent_10^4pfu_7d_1_RNA_ExpSam	RNA	IM004-P	IM004_HAavir_10^4_7d_1_proteomics	Protein
IM004	IM004_HAavirulent_10^4pfu_7d_2	Mus musculus	IM004_HAavirulent_10^4pfu_7d_2_lung	Tissue	IM004-R	IM004_HAavirulent_10^4pfu_7d_2_RNA_ExpSam	RNA	IM004-P	IM004_HAavir_10^4_7d_2_proteomics	Protein
IM004	IM004_HAavirulent_10^4pfu_7d_3	Mus musculus	IM004_HAavirulent_10^4pfu_7d_3_lung	Tissue	IM004-R	IM004_HAavirulent_10^4pfu_7d_3_RNA_ExpSam	RNA	IM004-P	IM004_HAavir_10^4_7d_3_proteomics	Protein
IM004	IM004_HAavirulent_10^4pfu_7d_4	Mus musculus	IM004_HAavirulent_10^4pfu_7d_4_lung	Tissue	IM004-R	IM004_HAavirulent_10^4pfu_7d_4_RNA_ExpSam	RNA	IM004-P	IM004_HAavir_10^4_7d_4_proteomics	Protein
IM004	IM004_HAavirulent_10^4pfu_7d_5	Mus musculus	IM004_HAavirulent_10^4pfu_7d_5_lung	Tissue	IM004-R	IM004_HAavirulent_10^4pfu_7d_5_RNA_ExpSam	RNA	IM004-P	IM004_HAavir_10^4_7d_5_proteomics	Protein
IM004	IM004_mock_1d_1	Mus musculus	IM004_mock_1d_1_lung	Tissue	IM004-R	IM004_mock_1d_1_RNA_ExpSam	RNA	IM004-P	IM004_Mock_1d_1_proteomics	Protein
IM004	IM004_mock_1d_2	Mus musculus	IM004_mock_1d_2_lung	Tissue	IM004-R	IM004_mock_1d_2_RNA_ExpSam	RNA	IM004-P	IM004_Mock_1d_2_proteomics	Protein
IM004	IM004_mock_1d_3	Mus musculus	IM004_mock_1d_3_lung	Tissue	IM004-R	IM004_mock_1d_3_RNA_ExpSam	RNA	IM004-P	IM004_Mock_1d_3_proteomics	Protein
IM004	IM004_mock_2d_1	Mus musculus	IM004_mock_2d_1_lung	Tissue	IM004-R	IM004_mock_2d_1_RNA_ExpSam	RNA	IM004-P	IM004_Mock_2d_1_proteomics	Protein
IM004	IM004_mock_2d_2	Mus musculus	IM004_mock_2d_2_lung	Tissue	IM004-R	IM004_mock_2d_2_RNA_ExpSam	RNA	IM004-P	IM004_Mock_2d_2_proteomics	Protein
IM004	IM004_mock_2d_3	Mus musculus	IM004_mock_2d_3_lung	Tissue	IM004-R	IM004_mock_2d_3_RNA_ExpSam	RNA	IM004-P	IM004_Mock_2d_3_proteomics	Protein
IM004	IM004_mock_4d_1	Mus musculus	IM004_mock_4d_1_lung	Tissue	IM004-R	IM004_mock_4d_1_RNA_ExpSam	RNA	IM004-P	IM004_Mock_4d_1_proteomics	Protein
IM004	IM004_mock_4d_2	Mus musculus	IM004_mock_4d_2_lung	Tissue	IM004-R	IM004_mock_4d_2_RNA_ExpSam	RNA	IM004-P	IM004_Mock_4d_2_proteomics	Protein
IM004	IM004_mock_4d_3	Mus musculus	IM004_mock_4d_3_lung	Tissue	IM004-R	IM004_mock_4d_3_RNA_ExpSam	RNA	IM004-P	IM004_Mock_4d_3_proteomics	Protein
IM004	IM004_mock_7d_1	Mus musculus	IM004_mock_7d_1_lung	Tissue	IM004-R	IM004_mock_7d_1_RNA_ExpSam	RNA	IM004-P	IM004_Mock_7d_1_proteomics	Protein
IM004	IM004_mock_7d_2	Mus musculus	IM004_mock_7d_2_lung	Tissue	IM004-R	IM004_mock_7d_2_RNA_ExpSam	RNA	IM004-P	IM004_Mock_7d_2_proteomics	Protein
IM004	IM004_mock_7d_3	Mus musculus	IM004_mock_7d_3_lung	Tissue	IM004-R	IM004_mock_7d_3_RNA_ExpSam	RNA	IM004-P	IM004_Mock_7d_3_proteomics	Protein
IM005	IM005_mock_1d_1	Mus musculus	IM005_mock_1d_1_Lung	Tissue	IM005-R	IM005_Mock_1d_1_array	RNA	IM005-P	IM005_Mock_1d_1_proteomics	Protein
IM005	IM005_mock_1d_2	Mus musculus	IM005_mock_1d_2_Lung	Tissue	IM005-R	IM005_Mock_1d_2_array	RNA	IM005-P	IM005_Mock_1d_2_proteomics	Protein
IM005	IM005_mock_1d_3	Mus musculus	IM005_mock_1d_3_Lung	Tissue	IM005-R	IM005_Mock_1d_3_array	RNA	IM005-P	IM005_Mock_1d_3_proteomics	Protein
IM005	IM005_mock_2d_1	Mus musculus	IM005_mock_2d_1_Lung	Tissue	IM005-R	IM005_Mock_2d_1_array	RNA	IM005-P	IM005_Mock_2d_1_proteomics	Protein
IM005	IM005_mock_2d_2	Mus musculus	IM005_mock_2d_2_Lung	Tissue	IM005-R	IM005_Mock_2d_2_array	RNA	IM005-P	IM005_Mock_2d_2_proteomics	Protein
IM005	IM005_mock_2d_3	Mus musculus	IM005_mock_2d_3_Lung	Tissue	IM005-R	IM005_Mock_2d_3_array	RNA	IM005-P	IM005_Mock_2d_3_proteomics	Protein
IM005	IM005_mock_4d_1	Mus musculus	IM005_mock_4d_1_Lung	Tissue	IM005-R	IM005_Mock_4d_1_array	RNA	IM005-P	IM005_Mock_4d_1_proteomics	Protein
IM005	IM005_mock_4d_2	Mus musculus	IM005_mock_4d_2_Lung	Tissue	IM005-R	IM005_Mock_4d_2_array	RNA	IM005-P	IM005_Mock_4d_2_proteomics	Protein
IM005	IM005_mock_4d_3	Mus musculus	IM005_mock_4d_3_Lung	Tissue	IM005-R	IM005_Mock_4d_3_array	RNA	IM005-P	IM005_Mock_4d_3_proteomics	Protein
IM005	IM005_mock_7d_1	Mus musculus	IM005_mock_7d_1_Lung	Tissue	IM005-R	IM005_Mock_7d_1_array	RNA	IM005-P	IM005_Mock_7d_1_proteomics	Protein
IM005	IM005_mock_7d_2	Mus musculus	IM005_mock_7d_2_Lung	Tissue	IM005-R	IM005_Mock_7d_2_array	RNA	IM005-P	IM005_Mock_7d_2_proteomics	Protein
IM005	IM005_mock_7d_3	Mus musculus	IM005_mock_7d_3_Lung	Tissue	IM005-R	IM005_Mock_7d_3_array	RNA	IM005-P	IM005_Mock_7d_3_proteomics	Protein
IM005	IM005_PB2_627E_10^4pfu_1d_1	Mus musculus	IM005_PB2_627E_10^4pfu_1d_1_Lung	Tissue	IM005-R	IM005_PB2_627E_10^4pfu_1d_1_array	RNA	IM005-P	IM005_PB2_627E_10^4pfu_1d_1_proteomics	Protein
IM005	IM005_PB2_627E_10^4pfu_1d_2	Mus musculus	IM005_PB2_627E_10^4pfu_1d_2_Lung	Tissue	IM005-R	IM005_PB2_627E_10^4pfu_1d_2_array	RNA	IM005-P	IM005_PB2_627E_10^4pfu_1d_2_proteomics	Protein
IM005	IM005_PB2_627E_10^4pfu_1d_3	Mus musculus	IM005_PB2_627E_10^4pfu_1d_3_Lung	Tissue	IM005-R	IM005_PB2_627E_10^4pfu_1d_3_array	RNA	IM005-P	IM005_PB2_627E_10^4pfu_1d_3_proteomics	Protein
IM005	IM005_PB2_627E_10^4pfu_1d_4	Mus musculus	IM005_PB2_627E_10^4pfu_1d_4_Lung	Tissue	IM005-R	IM005_PB2_627E_10^4pfu_1d_4_array	RNA	IM005-P	IM005_PB2_627E_10^4pfu_1d_4_proteomics	Protein
IM005	IM005_PB2_627E_10^4pfu_1d_5	Mus musculus	IM005_PB2_627E_10^4pfu_1d_5_Lung	Tissue	IM005-R	IM005_PB2_627E_10^4pfu_1d_5_array	RNA	IM005-P	IM005_PB2_627E_10^4pfu_1d_5_proteomics	Protein
IM005	IM005_PB2_627E_10^4pfu_2d_1	Mus musculus	IM005_PB2_627E_10^4pfu_2d_1_Lung	Tissue	IM005-R	IM005_PB2_627E_10^4pfu_2d_1_array	RNA	IM005-P	IM005_PB2_627E_10^4pfu_2d_1_proteomics	Protein
IM005	IM005_PB2_627E_10^4pfu_2d_2	Mus musculus	IM005_PB2_627E_10^4pfu_2d_2_Lung	Tissue	IM005-R	IM005_PB2_627E_10^4pfu_2d_2_array	RNA	IM005-P	IM005_PB2_627E_10^4pfu_2d_2_proteomics	Protein
IM005	IM005_PB2_627E_10^4pfu_2d_3	Mus musculus	IM005_PB2_627E_10^4pfu_2d_3_Lung	Tissue	IM005-R	IM005_PB2_627E_10^4pfu_2d_3_array	RNA	IM005-P	IM005_PB2_627E_10^4pfu_2d_3_proteomics	Protein
IM005	IM005_PB2_627E_10^4pfu_2d_4	Mus musculus	IM005_PB2_627E_10^4pfu_2d_4_Lung	Tissue	IM005-R	IM005_PB2_627E_10^4pfu_2d_4_array	RNA	IM005-P	IM005_PB2_627E_10^4pfu_2d_4_proteomics	Protein
IM005	IM005_PB2_627E_10^4pfu_2d_5	Mus musculus	IM005_PB2_627E_10^4pfu_2d_5_Lung	Tissue	IM005-R	IM005_PB2_627E_10^4pfu_2d_5_array	RNA	IM005-P	IM005_PB2_627E_10^4pfu_2d_5_proteomics	Protein
IM005	IM005_PB2_627E_10^4pfu_4d_1	Mus musculus	IM005_PB2_627E_10^4pfu_4d_1_Lung	Tissue	IM005-R	IM005_PB2_627E_10^4pfu_4d_1_array	RNA	IM005-P	IM005_PB2_627E_10^4pfu_4d_1_proteomics	Protein
IM005	IM005_PB2_627E_10^4pfu_4d_2	Mus musculus	IM005_PB2_627E_10^4pfu_4d_2_Lung	Tissue	IM005-R	IM005_PB2_627E_10^4pfu_4d_2_array	RNA	IM005-P	IM005_PB2_627E_10^4pfu_4d_2_proteomics	Protein
IM005	IM005_PB2_627E_10^4pfu_4d_3	Mus musculus	IM005_PB2_627E_10^4pfu_4d_3_Lung	Tissue	IM005-R	IM005_PB2_627E_10^4pfu_4d_3_array	RNA	IM005-P	IM005_PB2_627E_10^4pfu_4d_3_proteomics	Protein
IM005	IM005_PB2_627E_10^4pfu_4d_4	Mus musculus	IM005_PB2_627E_10^4pfu_4d_4_Lung	Tissue	IM005-R	IM005_PB2_627E_10^4pfu_4d_4_array	RNA	IM005-P	IM005_PB2_627E_10^4pfu_4d_4_proteomics	Protein
IM005	IM005_PB2_627E_10^4pfu_4d_5	Mus musculus	IM005_PB2_627E_10^4pfu_4d_5_Lung	Tissue	IM005-R	IM005_PB2_627E_10^4pfu_4d_5_array	RNA	IM005-P	IM005_PB2_627E_10^4pfu_4d_5_proteomics	Protein
IM005	IM005_PB2_627E_10^4pfu_7d_1	Mus musculus	IM005_PB2_627E_10^4pfu_7d_1_Lung	Tissue	IM005-R	IM005_PB2_627E_10^4pfu_7d_1_array	RNA	IM005-P	IM005_PB2_627E_10^4pfu_7d_1_proteomics	Protein
IM005	IM005_PB2_627E_10^4pfu_7d_2	Mus musculus	IM005_PB2_627E_10^4pfu_7d_2_Lung	Tissue	IM005-R	IM005_PB2_627E_10^4pfu_7d_2_array	RNA	IM005-P	IM005_PB2_627E_10^4pfu_7d_2_proteomics	Protein
IM005	IM005_PB2_627E_10^4pfu_7d_3	Mus musculus	IM005_PB2_627E_10^4pfu_7d_3_Lung	Tissue	IM005-R	IM005_PB2_627E_10^4pfu_7d_3_array	RNA	IM005-P	IM005_PB2_627E_10^4pfu_7d_3_proteomics	Protein
IM005	IM005_PB2_627E_10^4pfu_7d_4	Mus musculus	IM005_PB2_627E_10^4pfu_7d_4_Lung	Tissue	IM005-R	IM005_PB2_627E_10^4pfu_7d_4_array	RNA	IM005-P	IM005_PB2_627E_10^4pfu_7d_4_proteomics	Protein
IM005	IM005_PB2_627E_10^4pfu_7d_5	Mus musculus	IM005_PB2_627E_10^4pfu_7d_5_Lung	Tissue	IM005-R	IM005_PB2_627E_10^4pfu_7d_5_array	RNA	IM005-P	IM005_PB2_627E_10^4pfu_7d_5_proteomics	Protein
IM006A	IM006A_mock_1d_1	Mus musculus	IM006A_mock_1d_1_Lung	Tissue	IM006A-R	IM006A_Mock_1d_1_array	RNA	IM006A-P	IM006A_Mock_1d_1_proteomics	Protein
IM006A	IM006A_mock_1d_2	Mus musculus	IM006A_mock_1d_2_Lung	Tissue	IM006A-R	IM006A_Mock_1d_2_array	RNA	IM006A-P	IM006A_Mock_1d_2_proteomics	Protein
IM006A	IM006A_mock_1d_3	Mus musculus	IM006A_mock_1d_3_Lung	Tissue	IM006A-R	IM006A_Mock_1d_3_array	RNA	IM006A-P	IM006A_Mock_1d_3_proteomics	Protein
IM006A	IM006A_mock_2d_1	Mus musculus	IM006A_mock_2d_1_Lung	Tissue	IM006A-R	IM006A_Mock_2d_1_array	RNA	IM006A-P	IM006A_Mock_2d_1_proteomics	Protein
IM006A	IM006A_mock_2d_2	Mus musculus	IM006A_mock_2d_2_Lung	Tissue	IM006A-R	IM006A_Mock_2d_2_array	RNA	IM006A-P	IM006A_Mock_2d_2_proteomics	Protein
IM006A	IM006A_mock_2d_3	Mus musculus	IM006A_mock_2d_3_Lung	Tissue	IM006A-R	IM006A_Mock_2d_3_array	RNA	IM006A-P	IM006A_Mock_2d_3_proteomics	Protein
IM006A	IM006A_mock_4d_1	Mus musculus	IM006A_mock_4d_1_Lung	Tissue	IM006A-R	IM006A_Mock_4d_1_array	RNA	IM006A-P	IM006A_Mock_4d_1_proteomics	Protein
IM006A	IM006A_mock_4d_2	Mus musculus	IM006A_mock_4d_2_Lung	Tissue	IM006A-R	IM006A_Mock_4d_2_array	RNA	IM006A-P	IM006A_Mock_4d_2_proteomics	Protein
IM006A	IM006A_mock_4d_3	Mus musculus	IM006A_mock_4d_3_Lung	Tissue	IM006A-R	IM006A_Mock_4d_3_array	RNA	IM006A-P	IM006A_Mock_4d_3_proteomics	Protein
IM006A	IM006A_mock_7d_1	Mus musculus	IM006A_mock_7d_1_Lung	Tissue	IM006A-R	IM006A_Mock_7d_1_array	RNA	IM006A-P	IM006A_Mock_7d_1_proteomics	Protein
IM006A	IM006A_mock_7d_2	Mus musculus	IM006A_mock_7d_2_Lung	Tissue	IM006A-R	IM006A_Mock_7d_2_array	RNA	IM006A-P	IM006A_Mock_7d_2_proteomics	Protein
IM006A	IM006A_mock_7d_3	Mus musculus	IM006A_mock_7d_3_Lung	Tissue	IM006A-R	IM006A_Mock_7d_3_array	RNA	IM006A-P	IM006A_Mock_7d_3_proteomics	Protein
IM006A	IM006A_PB1-F2 Del_10^3pfu_1d_1	Mus musculus	IM006A_PB1-F2 Del_10^3pfu_1d_1_Lung	Tissue	IM006A-R	IM006A_PB1_F2del_10^3pfu_1d_1_array	RNA	IM006A-P	IM006A_PB1_F2del_10^3pfu_1d_1_proteomics	Protein
IM006A	IM006A_PB1-F2 Del_10^3pfu_1d_2	Mus musculus	IM006A_PB1-F2 Del_10^3pfu_1d_2_Lung	Tissue	IM006A-R	IM006A_PB1_F2del_10^3pfu_1d_2_array	RNA	IM006A-P	IM006A_PB1_F2del_10^3pfu_1d_2_proteomics	Protein
IM006A	IM006A_PB1-F2 Del_10^3pfu_1d_3	Mus musculus	IM006A_PB1-F2 Del_10^3pfu_1d_3_Lung	Tissue	IM006A-R	IM006A_PB1_F2del_10^3pfu_1d_3_array	RNA	IM006A-P	IM006A_PB1_F2del_10^3pfu_1d_3_proteomics	Protein
IM006A	IM006A_PB1-F2 Del_10^3pfu_1d_4	Mus musculus	IM006A_PB1-F2 Del_10^3pfu_1d_4_Lung	Tissue	IM006A-R	IM006A_PB1_F2del_10^3pfu_1d_4_array	RNA	IM006A-P	IM006A_PB1_F2del_10^3pfu_1d_4_proteomics	Protein
IM006A	IM006A_PB1-F2 Del_10^3pfu_1d_5	Mus musculus	IM006A_PB1-F2 Del_10^3pfu_1d_5_Lung	Tissue	IM006A-R	IM006A_PB1_F2del_10^3pfu_1d_5_array	RNA	IM006A-P	IM006A_PB1_F2del_10^3pfu_1d_5_proteomics	Protein
IM006A	IM006A_PB1-F2 Del_10^3pfu_2d_1	Mus musculus	IM006A_PB1-F2 Del_10^3pfu_2d_1_Lung	Tissue	IM006A-R	IM006A_PB1_F2del_10^3pfu_2d_1_array	RNA	IM006A-P	IM006A_PB1_F2del_10^3pfu_2d_1_proteomics	Protein
IM006A	IM006A_PB1-F2 Del_10^3pfu_2d_2	Mus musculus	IM006A_PB1-F2 Del_10^3pfu_2d_2_Lung	Tissue	IM006A-R	IM006A_PB1_F2del_10^3pfu_2d_2_array	RNA	IM006A-P	IM006A_PB1_F2del_10^3pfu_2d_2_proteomics	Protein
IM006A	IM006A_PB1-F2 Del_10^3pfu_2d_3	Mus musculus	IM006A_PB1-F2 Del_10^3pfu_2d_3_Lung	Tissue	IM006A-R	IM006A_PB1_F2del_10^3pfu_2d_3_array	RNA	IM006A-P	IM006A_PB1_F2del_10^3pfu_2d_3_proteomics	Protein
IM006A	IM006A_PB1-F2 Del_10^3pfu_2d_4	Mus musculus	IM006A_PB1-F2 Del_10^3pfu_2d_4_Lung	Tissue	IM006A-R	IM006A_PB1_F2del_10^3pfu_2d_4_array	RNA	IM006A-P	IM006A_PB1_F2del_10^3pfu_2d_4_proteomics	Protein
IM006A	IM006A_PB1-F2 Del_10^3pfu_2d_5	Mus musculus	IM006A_PB1-F2 Del_10^3pfu_2d_5_Lung	Tissue	IM006A-R	IM006A_PB1_F2del_10^3pfu_2d_5_array	RNA	IM006A-P	IM006A_PB1_F2del_10^3pfu_2d_5_proteomics	Protein
IM006A	IM006A_PB1-F2 Del_10^3pfu_4d_1	Mus musculus	IM006A_PB1-F2 Del_10^3pfu_4d_1_Lung	Tissue	IM006A-R	IM006A_PB1_F2del_10^3pfu_4d_1_array	RNA	IM006A-P	IM006A_PB1_F2del_10^3pfu_4d_1_proteomics	Protein
IM006A	IM006A_PB1-F2 Del_10^3pfu_4d_2	Mus musculus	IM006A_PB1-F2 Del_10^3pfu_4d_2_Lung	Tissue	IM006A-R	IM006A_PB1_F2del_10^3pfu_4d_2_array	RNA	IM006A-P	IM006A_PB1_F2del_10^3pfu_4d_2_proteomics	Protein
IM006A	IM006A_PB1-F2 Del_10^3pfu_4d_3	Mus musculus	IM006A_PB1-F2 Del_10^3pfu_4d_3_Lung	Tissue	IM006A-R	IM006A_PB1_F2del_10^3pfu_4d_3_array	RNA	IM006A-P	IM006A_PB1_F2del_10^3pfu_4d_3_proteomics	Protein
IM006A	IM006A_PB1-F2 Del_10^3pfu_4d_4	Mus musculus	IM006A_PB1-F2 Del_10^3pfu_4d_4_Lung	Tissue	IM006A-R	IM006A_PB1_F2del_10^3pfu_4d_4_array	RNA	IM006A-P	IM006A_PB1_F2del_10^3pfu_4d_4_proteomics	Protein
IM006A	IM006A_PB1-F2 Del_10^3pfu_4d_5	Mus musculus	IM006A_PB1-F2 Del_10^3pfu_4d_5_Lung	Tissue	IM006A-R	IM006A_PB1_F2del_10^3pfu_4d_5_array	RNA	IM006A-P	IM006A_PB1_F2del_10^3pfu_4d_5_proteomics	Protein
IM006A	IM006A_PB1-F2 Del_10^3pfu_7d_1	Mus musculus	IM006A_PB1-F2 Del_10^3pfu_7d_1_Lung	Tissue	IM006A-R	IM006A_PB1_F2del_10^3pfu_7d_1_array	RNA	IM006A-P	IM006A_PB1_F2del_10^3pfu_7d_1_proteomics	Protein
IM006A	IM006A_PB1-F2 Del_10^3pfu_7d_2	Mus musculus	IM006A_PB1-F2 Del_10^3pfu_7d_2_Lung	Tissue	IM006A-R	IM006A_PB1_F2del_10^3pfu_7d_2_array	RNA	IM006A-P	IM006A_PB1_F2del_10^3pfu_7d_2_proteomics	Protein
IM006A	IM006A_PB1-F2 Del_10^3pfu_7d_3	Mus musculus	IM006A_PB1-F2 Del_10^3pfu_7d_3_Lung	Tissue	IM006A-R	IM006A_PB1_F2del_10^3pfu_7d_3_array	RNA	IM006A-P	IM006A_PB1_F2del_10^3pfu_7d_3_proteomics	Protein
IM006A	IM006A_PB1-F2 Del_10^3pfu_7d_4	Mus musculus	IM006A_PB1-F2 Del_10^3pfu_7d_4_Lung	Tissue	IM006A-R		RNA	IM006A-P	IM006A_PB1_F2del_10^3pfu_7d_4_proteomics	Protein
IM006A	IM006A_PB1-F2 Del_10^3pfu_7d_5	Mus musculus	IM006A_PB1-F2 Del_10^3pfu_7d_5_Lung	Tissue	IM006B-R	IM006A_PB1_F2del_10^3pfu_7d_5_array	RNA	IM006A-P	IM006A_PB1_F2del_10^3pfu_7d_5_proteomics	Protein
IM006B	IM006B_mock_1d_1	Mus musculus	IM006B_mock_1d_1_Lung	Tissue	IM006B-R	IM006B_Mock_1d_1_array	RNA	IM006B-P	IM006B_Mock_1d_1_proteomics	Protein
IM006B	IM006B_mock_1d_2	Mus musculus	IM006B_mock_1d_2_Lung	Tissue	IM006B-R	IM006B_Mock_1d_2_array	RNA	IM006B-P	IM006B_Mock_1d_2_proteomics	Protein
IM006B	IM006B_mock_1d_3	Mus musculus	IM006B_mock_1d_3_Lung	Tissue	IM006B-R	IM006B_Mock_1d_3_array	RNA	IM006B-P	IM006B_Mock_1d_3_proteomics	Protein
IM006B	IM006B_mock_2d_1	Mus musculus	IM006B_mock_2d_1_Lung	Tissue	IM006B-R	IM006B_Mock_2d_1_array	RNA	IM006B-P	IM006B_Mock_2d_1_proteomics	Protein
IM006B	IM006B_mock_2d_2	Mus musculus	IM006B_mock_2d_2_Lung	Tissue	IM006B-R	IM006B_Mock_2d_2_array	RNA	IM006B-P	IM006B_Mock_2d_2_proteomics	Protein
IM006B	IM006B_mock_2d_3	Mus musculus	IM006B_mock_2d_3_Lung	Tissue	IM006B-R	IM006B_Mock_2d_3_array	RNA	IM006B-P	IM006B_Mock_2d_3_proteomics	Protein
IM006B	IM006B_mock_4d_1	Mus musculus	IM006B_mock_4d_1_Lung	Tissue	IM006B-R	IM006B_Mock_4d_1_array	RNA	IM006B-P	IM006B_Mock_4d_1_proteomics	Protein
IM006B	IM006B_mock_4d_2	Mus musculus	IM006B_mock_4d_2_Lung	Tissue	IM006B-R	IM006B_Mock_4d_2_array	RNA	IM006B-P	IM006B_Mock_4d_2_proteomics	Protein
IM006B	IM006B_mock_4d_3	Mus musculus	IM006B_mock_4d_3_Lung	Tissue	IM006B-R	IM006B_Mock_4d_3_array	RNA	IM006B-P	IM006B_Mock_4d_3_proteomics	Protein
IM006B	IM006B_mock_7d_1	Mus musculus	IM006B_mock_7d_1_Lung	Tissue	IM006B-R	IM006B_Mock_7d_1_array	RNA			
IM006B	IM006B_mock_7d_2	Mus musculus	IM006B_mock_7d_2_Lung	Tissue	IM006B-R	IM006B_Mock_7d_2_array	RNA			
IM006B	IM006B_mock_7d_3	Mus musculus	IM006B_mock_7d_3_Lung	Tissue	IM006B-R		RNA			
IM006B	IM006B_PB1-F2 Del_10^4pfu_1d_1	Mus musculus	IM006B_PB1-F2 Del_10^4pfu_1d_1_Lung	Tissue	IM006B-R	IM006B_PB1_F2del_10^4pfu_1d_1_array	RNA	IM006B-P	IM006B_PB1_F2del_10^4pfu_1d_1_proteomics	Protein
IM006B	IM006B_PB1-F2 Del_10^4pfu_1d_2	Mus musculus	IM006B_PB1-F2 Del_10^4pfu_1d_2_Lung	Tissue	IM006B-R	IM006B_PB1_F2del_10^4pfu_1d_2_array	RNA	IM006B-P	IM006B_PB1_F2del_10^4pfu_1d_2_proteomics	Protein
IM006B	IM006B_PB1-F2 Del_10^4pfu_1d_3	Mus musculus	IM006B_PB1-F2 Del_10^4pfu_1d_3_Lung	Tissue	IM006B-R	IM006B_PB1_F2del_10^4pfu_1d_3_array	RNA	IM006B-P	IM006B_PB1_F2del_10^4pfu_1d_3_proteomics	Protein
IM006B	IM006B_PB1-F2 Del_10^4pfu_1d_4	Mus musculus	IM006B_PB1-F2 Del_10^4pfu_1d_4_Lung	Tissue	IM006B-R	IM006B_PB1_F2del_10^4pfu_1d_4_array	RNA	IM006B-P	IM006B_PB1_F2del_10^4pfu_1d_4_proteomics	Protein
IM006B	IM006B_PB1-F2 Del_10^4pfu_1d_5	Mus musculus	IM006B_PB1-F2 Del_10^4pfu_1d_5_Lung	Tissue	IM006B-R	IM006B_PB1_F2del_10^4pfu_1d_5_array	RNA	IM006B-P	IM006B_PB1_F2del_10^4pfu_1d_5_proteomics	Protein
IM006B	IM006B_PB1-F2 Del_10^4pfu_2d_1	Mus musculus	IM006B_PB1-F2 Del_10^4pfu_2d_1_Lung	Tissue	IM006B-R	IM006B_PB1_F2del_10^4pfu_2d_1_array	RNA	IM006B-P	IM006B_PB1_F2del_10^4pfu_2d_1_proteomics	Protein
IM006B	IM006B_PB1-F2 Del_10^4pfu_2d_2	Mus musculus	IM006B_PB1-F2 Del_10^4pfu_2d_2_Lung	Tissue	IM006B-R	IM006B_PB1_F2del_10^4pfu_2d_2_array	RNA	IM006B-P	IM006B_PB1_F2del_10^4pfu_2d_2_proteomics	Protein
IM006B	IM006B_PB1-F2 Del_10^4pfu_2d_3	Mus musculus	IM006B_PB1-F2 Del_10^4pfu_2d_3_Lung	Tissue	IM006B-R	IM006B_PB1_F2del_10^4pfu_2d_3_array	RNA	IM006B-P	IM006B_PB1_F2del_10^4pfu_2d_3_proteomics	Protein
IM006B	IM006B_PB1-F2 Del_10^4pfu_2d_4	Mus musculus	IM006B_PB1-F2 Del_10^4pfu_2d_4_Lung	Tissue	IM006B-R	IM006B_PB1_F2del_10^4pfu_2d_4_array	RNA	IM006B-P	IM006B_PB1_F2del_10^4pfu_2d_4_proteomics	Protein
IM006B	IM006B_PB1-F2 Del_10^4pfu_2d_5	Mus musculus	IM006B_PB1-F2 Del_10^4pfu_2d_5_Lung	Tissue	IM006B-R	IM006B_PB1_F2del_10^4pfu_2d_5_array	RNA	IM006B-P	IM006B_PB1_F2del_10^4pfu_2d_5_proteomics	Protein
IM006B	IM006B_PB1-F2 Del_10^4pfu_4d_1	Mus musculus	IM006B_PB1-F2 Del_10^4pfu_4d_1_Lung	Tissue	IM006B-R	IM006B_PB1_F2del_10^4pfu_4d_1_array	RNA	IM006B-P	IM006B_PB1_F2del_10^4pfu_4d_1_proteomics	Protein
IM006B	IM006B_PB1-F2 Del_10^4pfu_4d_2	Mus musculus	IM006B_PB1-F2 Del_10^4pfu_4d_2_Lung	Tissue	IM006B-R	IM006B_PB1_F2del_10^4pfu_4d_2_array	RNA	IM006B-P	IM006B_PB1_F2del_10^4pfu_4d_2_proteomics	Protein
IM006B	IM006B_PB1-F2 Del_10^4pfu_4d_3	Mus musculus	IM006B_PB1-F2 Del_10^4pfu_4d_3_Lung	Tissue	IM006B-R	IM006B_PB1_F2del_10^4pfu_4d_3_array	RNA	IM006B-P	IM006B_PB1_F2del_10^4pfu_4d_3_proteomics	Protein
IM006B	IM006B_PB1-F2 Del_10^4pfu_4d_4	Mus musculus	IM006B_PB1-F2 Del_10^4pfu_4d_4_Lung	Tissue	IM006B-R	IM006B_PB1_F2del_10^4pfu_4d_4_array	RNA	IM006B-P	IM006B_PB1_F2del_10^4pfu_4d_4_proteomics	Protein
IM006B	IM006B_PB1-F2 Del_10^4pfu_4d_5	Mus musculus	IM006B_PB1-F2 Del_10^4pfu_4d_5_Lung	Tissue	IM007-R	IM006B_PB1_F2del_10^4pfu_4d_5_array	RNA	IM006B-P	IM006B_PB1_F2del_10^4pfu_4d_5_proteomics	Protein
IM006B	IM006B_PB1-F2 Del_10^4pfu_7d_1	Mus musculus	IM006B_PB1-F2 Del_10^4pfu_7d_1_Lung	Tissue	IM007-R	IM006B_PB1_F2del_10^4pfu_7d_1_array	RNA			
IM007	IM007_mock_1d_1	Mus musculus	IM007_mock_1d_1_Lung	Tissue	IM007-R	IM007_Mock_1d_1_array	RNA	IM007-P	IM007_Mock_1d_1_proteomics	Protein
IM007	IM007_mock_1d_2	Mus musculus	IM007_mock_1d_2_Lung	Tissue	IM007-R	IM007_Mock_1d_2_array	RNA	IM007-P	IM007_Mock_1d_2_proteomics	Protein
IM007	IM007_mock_1d_3	Mus musculus	IM007_mock_1d_3_Lung	Tissue	IM007-R	IM007_Mock_1d_3_array	RNA	IM007-P	IM007_Mock_1d_3_proteomics	Protein
IM007	IM007_mock_2d_1	Mus musculus	IM007_mock_2d_1_Lung	Tissue	IM007-R	IM007_Mock_2d_1_array	RNA	IM007-P	IM007_Mock_2d_1_proteomics	Protein
IM007	IM007_mock_2d_2	Mus musculus	IM007_mock_2d_2_Lung	Tissue	IM007-R	IM007_Mock_2d_2_array	RNA	IM007-P	IM007_Mock_2d_2_proteomics	Protein
IM007	IM007_mock_2d_3	Mus musculus	IM007_mock_2d_3_Lung	Tissue	IM007-R	IM007_Mock_2d_3_array	RNA	IM007-P	IM007_Mock_2d_3_proteomics	Protein
IM007	IM007_mock_4d_1	Mus musculus	IM007_mock_4d_1_Lung	Tissue	IM007-R	IM007_Mock_4d_1_array	RNA	IM007-P	IM007_Mock_4d_1_proteomics	Protein
IM007	IM007_mock_4d_2	Mus musculus	IM007_mock_4d_2_Lung	Tissue	IM007-R	IM007_Mock_4d_2_array	RNA	IM007-P	IM007_Mock_4d_2_proteomics	Protein
IM007	IM007_mock_4d_3	Mus musculus	IM007_mock_4d_3_Lung	Tissue	IM007-R	IM007_Mock_4d_3_array	RNA	IM007-P	IM007_Mock_4d_3_proteomics	Protein
IM007	IM007_mock_7d_1	Mus musculus	IM007_mock_7d_1_Lung	Tissue	IM007-R	IM007_Mock_7d_1_array	RNA	IM007-P	IM007_Mock_7d_1_proteomics	Protein
IM007	IM007_mock_7d_2	Mus musculus	IM007_mock_7d_2_Lung	Tissue	IM007-R	IM007_Mock_7d_2_array	RNA	IM007-P	IM007_Mock_7d_2_proteomics	Protein
IM007	IM007_mock_7d_3	Mus musculus	IM007_mock_7d_3_Lung	Tissue	IM007-R	IM007_Mock_7d_3_array	RNA	IM007-P	IM007_Mock_7d_3_proteomics	Protein
IM007	IM007_VN1203-NS1trunc-124_10^3_1d_1	Mus musculus	IM007_VN1203-NS1trunc-124_10^3_1d_1_Lung	Tissue	IM007-R	IM007_NS1trunc124_10^3pfu_1d_1_array	RNA	IM007-P	IM007_NS1trunc124_10^3pfu_1d_1_proteomics	Protein
IM007	IM007_VN1203-NS1trunc-124_10^3_1d_2	Mus musculus	IM007_VN1203-NS1trunc-124_10^3_1d_2_Lung	Tissue	IM007-R	IM007_NS1trunc124_10^3pfu_1d_2_array	RNA	IM007-P	IM007_NS1trunc124_10^3pfu_1d_2_proteomics	Protein
IM007	IM007_VN1203-NS1trunc-124_10^3_1d_3	Mus musculus	IM007_VN1203-NS1trunc-124_10^3_1d_3_Lung	Tissue	IM007-R	IM007_NS1trunc124_10^3pfu_1d_3_array	RNA	IM007-P	IM007_NS1trunc124_10^3pfu_1d_3_proteomics	Protein
IM007	IM007_VN1203-NS1trunc-124_10^3_1d_4	Mus musculus	IM007_VN1203-NS1trunc-124_10^3_1d_4_Lung	Tissue	IM007-R	IM007_NS1trunc124_10^3pfu_1d_4_array	RNA	IM007-P	IM007_NS1trunc124_10^3pfu_1d_4_proteomics	Protein
IM007	IM007_VN1203-NS1trunc-124_10^3_1d_5	Mus musculus	IM007_VN1203-NS1trunc-124_10^3_1d_5_Lung	Tissue	IM007-R	IM007_NS1trunc124_10^3pfu_1d_5_array	RNA	IM007-P	IM007_NS1trunc124_10^3pfu_1d_5_proteomics	Protein
IM007	IM007_VN1203-NS1trunc-124_10^3_2d_1	Mus musculus	IM007_VN1203-NS1trunc-124_10^3_2d_1_Lung	Tissue	IM007-R	IM007_NS1trunc124_10^3pfu_2d_1_array	RNA	IM007-P	IM007_NS1trunc124_10^3pfu_2d_1_proteomics	Protein
IM007	IM007_VN1203-NS1trunc-124_10^3_2d_2	Mus musculus	IM007_VN1203-NS1trunc-124_10^3_2d_2_Lung	Tissue	IM007-R	IM007_NS1trunc124_10^3pfu_2d_2_array	RNA	IM007-P	IM007_NS1trunc124_10^3pfu_2d_2_proteomics	Protein
IM007	IM007_VN1203-NS1trunc-124_10^3_2d_3	Mus musculus	IM007_VN1203-NS1trunc-124_10^3_2d_3_Lung	Tissue	IM007-R	IM007_NS1trunc124_10^3pfu_2d_3_array	RNA	IM007-P	IM007_NS1trunc124_10^3pfu_2d_3_proteomics	Protein
IM007	IM007_VN1203-NS1trunc-124_10^3_2d_4	Mus musculus	IM007_VN1203-NS1trunc-124_10^3_2d_4_Lung	Tissue	IM007-R	IM007_NS1trunc124_10^3pfu_2d_4_array	RNA	IM007-P	IM007_NS1trunc124_10^3pfu_2d_4_proteomics	Protein
IM007	IM007_VN1203-NS1trunc-124_10^3_2d_5	Mus musculus	IM007_VN1203-NS1trunc-124_10^3_2d_5_Lung	Tissue	IM007-R	IM007_NS1trunc124_10^3pfu_2d_5_array	RNA	IM007-P	IM007_NS1trunc124_10^3pfu_2d_5_proteomics	Protein
IM007	IM007_VN1203-NS1trunc-124_10^3_4d_1	Mus musculus	IM007_VN1203-NS1trunc-124_10^3_4d_1_Lung	Tissue	IM007-R	IM007_NS1trunc124_10^3pfu_4d_1_array	RNA	IM007-P	IM007_NS1trunc124_10^3pfu_4d_1_proteomics	Protein
IM007	IM007_VN1203-NS1trunc-124_10^3_4d_2	Mus musculus	IM007_VN1203-NS1trunc-124_10^3_4d_2_Lung	Tissue	IM007-R	IM007_NS1trunc124_10^3pfu_4d_2_array	RNA	IM007-P	IM007_NS1trunc124_10^3pfu_4d_2_proteomics	Protein
IM007	IM007_VN1203-NS1trunc-124_10^3_4d_3	Mus musculus	IM007_VN1203-NS1trunc-124_10^3_4d_3_Lung	Tissue	IM007-R	IM007_NS1trunc124_10^3pfu_4d_3_array	RNA	IM007-P	IM007_NS1trunc124_10^3pfu_4d_3_proteomics	Protein
IM007	IM007_VN1203-NS1trunc-124_10^3_4d_4	Mus musculus	IM007_VN1203-NS1trunc-124_10^3_4d_4_Lung	Tissue	IM007-R	IM007_NS1trunc124_10^3pfu_4d_4_array	RNA	IM007-P	IM007_NS1trunc124_10^3pfu_4d_4_proteomics	Protein
IM007	IM007_VN1203-NS1trunc-124_10^3_4d_5	Mus musculus	IM007_VN1203-NS1trunc-124_10^3_4d_5_Lung	Tissue	IM007-R	IM007_NS1trunc124_10^3pfu_4d_5_array	RNA	IM007-P	IM007_NS1trunc124_10^3pfu_4d_5_proteomics	Protein
IM007	IM007_VN1203-NS1trunc-124_10^3_7d_1	Mus musculus	IM007_VN1203-NS1trunc-124_10^3_7d_1_Lung	Tissue	IM007-R	IM007_NS1trunc124_10^3pfu_7d_1_array	RNA	IM007-P	IM007_NS1trunc124_10^3pfu_7d_1_proteomics	Protein
IM007	IM007_VN1203-NS1trunc-124_10^3_7d_2	Mus musculus	IM007_VN1203-NS1trunc-124_10^3_7d_2_Lung	Tissue	IM007-R	IM007_NS1trunc124_10^3pfu_7d_2_array	RNA	IM007-P	IM007_NS1trunc124_10^3pfu_7d_2_proteomics	Protein
IM007	IM007_VN1203-NS1trunc-124_10^3_7d_3	Mus musculus	IM007_VN1203-NS1trunc-124_10^3_7d_3_Lung	Tissue	IM007-R	IM007_NS1trunc124_10^3pfu_7d_3_array	RNA	IM007-P	IM007_NS1trunc124_10^3pfu_7d_3_proteomics	Protein
IM007	IM007_VN1203-NS1trunc-124_10^3_7d_4	Mus musculus	IM007_VN1203-NS1trunc-124_10^3_7d_4_Lung	Tissue	IM007-R	IM007_NS1trunc124_10^3pfu_7d_4_array	RNA	IM007-P	IM007_NS1trunc124_10^3pfu_7d_4_proteomics	Protein
IM007	IM007_VN1203-NS1trunc-124_10^3_7d_5	Mus musculus	IM007_VN1203-NS1trunc-124_10^3_7d_5_Lung	Tissue	IM007-R	IM007_NS1trunc124_10^3pfu_7d_5_array	RNA	IM007-P	IM007_NS1trunc124_10^3pfu_7d_5_proteomics	Protein
IM007	IM007_VN1203-NS1trunc-124_10^4_1d_1	Mus musculus	IM007_VN1203-NS1trunc-124_10^4_1d_1_Lung	Tissue	IM007-R	IM007_NS1trunc124_10^4pfu_1d_1_array	RNA	IM007-P	IM007_NS1trunc124_10^4pfu_1d_1_proteomics	Protein
IM007	IM007_VN1203-NS1trunc-124_10^4_1d_2	Mus musculus	IM007_VN1203-NS1trunc-124_10^4_1d_2_Lung	Tissue	IM007-R	IM007_NS1trunc124_10^4pfu_1d_2_array	RNA	IM007-P	IM007_NS1trunc124_10^4pfu_1d_2_proteomics	Protein
IM007	IM007_VN1203-NS1trunc-124_10^4_1d_3	Mus musculus	IM007_VN1203-NS1trunc-124_10^4_1d_3_Lung	Tissue	IM007-R	IM007_NS1trunc124_10^4pfu_1d_3_array	RNA	IM007-P	IM007_NS1trunc124_10^4pfu_1d_3_proteomics	Protein
IM007	IM007_VN1203-NS1trunc-124_10^4_1d_4	Mus musculus	IM007_VN1203-NS1trunc-124_10^4_1d_4_Lung	Tissue	IM007-R	IM007_NS1trunc124_10^4pfu_1d_4_array	RNA	IM007-P	IM007_NS1trunc124_10^4pfu_1d_4_proteomics	Protein
IM007	IM007_VN1203-NS1trunc-124_10^4_1d_5	Mus musculus	IM007_VN1203-NS1trunc-124_10^4_1d_5_Lung	Tissue	IM007-R	IM007_NS1trunc124_10^4pfu_1d_5_array	RNA	IM007-P	IM007_NS1trunc124_10^4pfu_1d_5_proteomics	Protein
IM007	IM007_VN1203-NS1trunc-124_10^4_2d_1	Mus musculus	IM007_VN1203-NS1trunc-124_10^4_2d_1_Lung	Tissue	IM007-R	IM007_NS1trunc124_10^4pfu_2d_1_array	RNA	IM007-P	IM007_NS1trunc124_10^4pfu_2d_1_proteomics	Protein
IM007	IM007_VN1203-NS1trunc-124_10^4_2d_2	Mus musculus	IM007_VN1203-NS1trunc-124_10^4_2d_2_Lung	Tissue	IM007-R	IM007_NS1trunc124_10^4pfu_2d_2_array	RNA	IM007-P	IM007_NS1trunc124_10^4pfu_2d_2_proteomics	Protein
IM007	IM007_VN1203-NS1trunc-124_10^4_2d_3	Mus musculus	IM007_VN1203-NS1trunc-124_10^4_2d_3_Lung	Tissue	IM007-R	IM007_NS1trunc124_10^4pfu_2d_3_array	RNA	IM007-P	IM007_NS1trunc124_10^4pfu_2d_3_proteomics	Protein
IM007	IM007_VN1203-NS1trunc-124_10^4_2d_4	Mus musculus	IM007_VN1203-NS1trunc-124_10^4_2d_4_Lung	Tissue	IM007-R	IM007_NS1trunc124_10^4pfu_2d_4_array	RNA	IM007-P	IM007_NS1trunc124_10^4pfu_2d_4_proteomics	Protein
IM007	IM007_VN1203-NS1trunc-124_10^4_2d_5	Mus musculus	IM007_VN1203-NS1trunc-124_10^4_2d_5_Lung	Tissue	IM007-R	IM007_NS1trunc124_10^4pfu_2d_5_array	RNA	IM007-P	IM007_NS1trunc124_10^4pfu_2d_5_proteomics	Protein
IM007	IM007_VN1203-NS1trunc-124_10^4_4d_1	Mus musculus	IM007_VN1203-NS1trunc-124_10^4_4d_1_Lung	Tissue	IM007-R	IM007_NS1trunc124_10^4pfu_4d_1_array	RNA	IM007-P	IM007_NS1trunc124_10^4pfu_4d_1_proteomics	Protein
IM007	IM007_VN1203-NS1trunc-124_10^4_4d_2	Mus musculus	IM007_VN1203-NS1trunc-124_10^4_4d_2_Lung	Tissue	IM007-R	IM007_NS1trunc124_10^4pfu_4d_2_array	RNA	IM007-P	IM007_NS1trunc124_10^4pfu_4d_2_proteomics	Protein
IM007	IM007_VN1203-NS1trunc-124_10^4_4d_3	Mus musculus	IM007_VN1203-NS1trunc-124_10^4_4d_3_Lung	Tissue	IM007-R	IM007_NS1trunc124_10^4pfu_4d_3_array	RNA	IM007-P	IM007_NS1trunc124_10^4pfu_4d_3_proteomics	Protein
IM007	IM007_VN1203-NS1trunc-124_10^4_4d_4	Mus musculus	IM007_VN1203-NS1trunc-124_10^4_4d_4_Lung	Tissue	IM007-R	IM007_NS1trunc124_10^4pfu_4d_4_array	RNA	IM007-P	IM007_NS1trunc124_10^4pfu_4d_4_proteomics	Protein
IM007	IM007_VN1203-NS1trunc-124_10^4_4d_5	Mus musculus	IM007_VN1203-NS1trunc-124_10^4_4d_5_Lung	Tissue	IM007-R	IM007_NS1trunc124_10^4pfu_4d_5_array	RNA	IM007-P	IM007_NS1trunc124_10^4pfu_4d_5_proteomics	Protein
IM007	IM007_VN1203-NS1trunc-124_10^4_7d_1	Mus musculus	IM007_VN1203-NS1trunc-124_10^4_7d_1_Lung	Tissue	IM007-R	IM007_NS1trunc124_10^4pfu_7d_1_array	RNA	IM007-P	IM007_NS1trunc124_10^4pfu_7d_1_proteomics	Protein
IM007	IM007_VN1203-NS1trunc-124_10^4_7d_2	Mus musculus	IM007_VN1203-NS1trunc-124_10^4_7d_2_Lung	Tissue	IM007-R	IM007_NS1trunc124_10^4pfu_7d_2_array	RNA	IM007-P	IM007_NS1trunc124_10^4pfu_7d_2_proteomics	Protein
IM007	IM007_VN1203-NS1trunc-124_10^4_7d_3	Mus musculus	IM007_VN1203-NS1trunc-124_10^4_7d_3_Lung	Tissue	IM007-R	IM007_NS1trunc124_10^4pfu_7d_3_array	RNA	IM007-P	IM007_NS1trunc124_10^4pfu_7d_3_proteomics	Protein
IM007	IM007_VN1203-NS1trunc-124_10^4_7d_4	Mus musculus	IM007_VN1203-NS1trunc-124_10^4_7d_4_Lung	Tissue	IM009-R	IM007_NS1trunc124_10^4pfu_7d_4_array	RNA	IM007-P	IM007_NS1trunc124_10^4pfu_7d_4_proteomics	Protein
IM007	IM007_VN1203-NS1trunc-124_10^4_7d_5	Mus musculus	IM007_VN1203-NS1trunc-124_10^4_7d_5_Lung	Tissue	IM009-R	IM007_NS1trunc124_10^4pfu_7d_5_array	RNA	IM007-P	IM007_NS1trunc124_10^4pfu_7d_5_proteomics	Protein
IM009	IM009_CA04_10^6pfu_D1_1	Mus musculus	IM009_CA04_10^6pfu_D1_1_Lung	Tissue	IM009-R	IM009_CA04_10^6pfu_D1_1_array	RNA			
IM009	IM009_CA04_10^6pfu_D1_2	Mus musculus	IM009_CA04_10^6pfu_D1_2_Lung	Tissue	IM009-R	IM009_CA04_10^6pfu_D1_2_array	RNA			
IM009	IM009_CA04_10^6pfu_D1_3	Mus musculus	IM009_CA04_10^6pfu_D1_3_Lung	Tissue	IM009-R	IM009_CA04_10^6pfu_D1_3_array	RNA			
IM009	IM009_CA04_10^6pfu_D3_1	Mus musculus	IM009_CA04_10^6pfu_D3_1_Lung	Tissue	IM009-R	IM009_CA04_10^6pfu_D3_1_array	RNA			
IM009	IM009_CA04_10^6pfu_D3_2	Mus musculus	IM009_CA04_10^6pfu_D3_2_Lung	Tissue	IM009-R	IM009_CA04_10^6pfu_D3_2_array	RNA			
IM009	IM009_CA04_10^6pfu_D3_3	Mus musculus	IM009_CA04_10^6pfu_D3_3_Lung	Tissue	IM009-R	IM009_CA04_10^6pfu_D3_3_array	RNA			
IM009	IM009_CA04_10^6pfu_D5_1	Mus musculus	IM009_CA04_10^6pfu_D5_1_Lung	Tissue	IM009-R	IM009_CA04_10^6pfu_D5_1_array	RNA			
IM009	IM009_CA04_10^6pfu_D5_2	Mus musculus	IM009_CA04_10^6pfu_D5_2_Lung	Tissue	IM009-R	IM009_CA04_10^6pfu_D5_2_array	RNA			
IM009	IM009_CA04_10^6pfu_D5_3	Mus musculus	IM009_CA04_10^6pfu_D5_3_Lung	Tissue	IM009-R	IM009_CA04_10^6pfu_D5_3_array	RNA			
IM009	IM009_MA-CA04_10^6pfu_D1_1	Mus musculus	IM009_MA-CA04_10^6pfu_D1_1_Lung	Tissue	IM009-R	IM009_MA-CA04_10^6pfu_D1_1_array	RNA			
IM009	IM009_MA-CA04_10^6pfu_D1_2	Mus musculus	IM009_MA-CA04_10^6pfu_D1_2_Lung	Tissue	IM009-R	IM009_MA-CA04_10^6pfu_D1_2_array	RNA			
IM009	IM009_MA-CA04_10^6pfu_D1_3	Mus musculus	IM009_MA-CA04_10^6pfu_D1_3_Lung	Tissue	IM009-R	IM009_MA-CA04_10^6pfu_D1_3_array	RNA			
IM009	IM009_MA-CA04_10^6pfu_D3_1	Mus musculus	IM009_MA-CA04_10^6pfu_D3_1_Lung	Tissue	IM009-R	IM009_MA-CA04_10^6pfu_D3_1_array	RNA			
IM009	IM009_MA-CA04_10^6pfu_D3_2	Mus musculus	IM009_MA-CA04_10^6pfu_D3_2_Lung	Tissue	IM009-R	IM009_MA-CA04_10^6pfu_D3_2_array	RNA			
IM009	IM009_MA-CA04_10^6pfu_D3_3	Mus musculus	IM009_MA-CA04_10^6pfu_D3_3_Lung	Tissue	IM009-R	IM009_MA-CA04_10^6pfu_D3_3_array	RNA			
IM009	IM009_MA-CA04_10^6pfu_D5_1	Mus musculus	IM009_MA-CA04_10^6pfu_D5_1_Lung	Tissue	IM009-R	IM009_MA-CA04_10^6pfu_D5_1_array	RNA			
IM009	IM009_MA-CA04_10^6pfu_D5_2	Mus musculus	IM009_MA-CA04_10^6pfu_D5_2_Lung	Tissue	IM009-R	IM009_MA-CA04_10^6pfu_D5_2_array	RNA			
IM009	IM009_MA-CA04_10^6pfu_D5_3	Mus musculus	IM009_MA-CA04_10^6pfu_D5_3_Lung	Tissue	IM009-R	IM009_MA-CA04_10^6pfu_D5_3_array	RNA			
IM009	IM009_Mock_D1_1	Mus musculus	IM009_Mock_D1_1_Lung	Tissue	IM009-R	IM009_Mock_D1_1_array	RNA			
IM009	IM009_Mock_D1_2	Mus musculus	IM009_Mock_D1_2_Lung	Tissue	IM009-R	IM009_Mock_D1_2_array	RNA			
IM009	IM009_Mock_D1_3	Mus musculus	IM009_Mock_D1_3_Lung	Tissue	IM009-R	IM009_Mock_D1_3_array	RNA			
IM009	IM009_Mock_D3_1	Mus musculus	IM009_Mock_D3_1_Lung	Tissue	IM009-R	IM009_Mock_D3_1_array	RNA			
IM009	IM009_Mock_D3_2	Mus musculus	IM009_Mock_D3_2_Lung	Tissue	IM009-R	IM009_Mock_D3_2_array	RNA			
IM009	IM009_Mock_D3_3	Mus musculus	IM009_Mock_D3_3_Lung	Tissue	IM009-R	IM009_Mock_D3_3_array	RNA			
IM009	IM009_Mock_D5_1	Mus musculus	IM009_Mock_D5_1_Lung	Tissue	IM010-R	IM009_Mock_D5_1_array	RNA			
IM009	IM009_Mock_D5_2	Mus musculus	IM009_Mock_D5_2_Lung	Tissue	IM010-R	IM009_Mock_D5_2_array	RNA			
IM010	IM010_B6_Mock_2d_1	Mus musculus	IM010_B6_Mock_2d_1_Lung	Tissue	IM010-R	IM010_B6_Mock_2d_1_array	RNA			
IM010	IM010_B6_Mock_2d_2	Mus musculus	IM010_B6_Mock_2d_2_Lung	Tissue	IM010-R	IM010_B6_Mock_2d_2_array	RNA			
IM010	IM010_B6_Mock_2d_3	Mus musculus	IM010_B6_Mock_2d_3_Lung	Tissue	IM010-R	IM010_B6_Mock_2d_3_array	RNA			
IM010	IM010_B6_VN1203_2d_1	Mus musculus	IM010_B6_VN1203_2d_1_Lung	Tissue	IM010-R	IM010_B6_VN1203_2d_1_array	RNA			
IM010	IM010_B6_VN1203_2d_2	Mus musculus	IM010_B6_VN1203_2d_2_Lung	Tissue	IM010-R	IM010_B6_VN1203_2d_2_array	RNA			
IM010	IM010_B6_VN1203_2d_3	Mus musculus	IM010_B6_VN1203_2d_3_Lung	Tissue	IM010-R	IM010_B6_VN1203_2d_3_array	RNA			
IM010	IM010_IDO1_Mock_2d_1	Mus musculus	IM010_IDO1_Mock_2d_1_Lung	Tissue	IM010-R	IM010_IDO1_Mock_2d_1_array	RNA			
IM010	IM010_IDO1_Mock_2d_2	Mus musculus	IM010_IDO1_Mock_2d_2_Lung	Tissue	IM010-R	IM010_IDO1_Mock_2d_2_array	RNA			
IM010	IM010_IDO1_Mock_2d_3	Mus musculus	IM010_IDO1_Mock_2d_3_Lung	Tissue	IM010-R	IM010_IDO1_Mock_2d_3_array	RNA			
IM010	IM010_IDO1_Mock_6d_1	Mus musculus	IM010_IDO1_Mock_6d_1_Lung	Tissue	IM010-R	IM010_IDO1_Mock_6d_1_array	RNA			
IM010	IM010_IDO1_Mock_6d_2	Mus musculus	IM010_IDO1_Mock_6d_2_Lung	Tissue	IM010-R	IM010_IDO1_Mock_6d_2_array	RNA			
IM010	IM010_IDO1_Mock_6d_3	Mus musculus	IM010_IDO1_Mock_6d_3_Lung	Tissue	IM010-R	IM010_IDO1_Mock_6d_3_array	RNA			
IM010	IM010_IDO1_VN1203_2d_1	Mus musculus	IM010_IDO1_VN1203_2d_1_Lung	Tissue	IM010-R	IM010_IDO1_VN1203_2d_1_array	RNA			
IM010	IM010_IDO1_VN1203_2d_2	Mus musculus	IM010_IDO1_VN1203_2d_2_Lung	Tissue	IM010-R	IM010_IDO1_VN1203_2d_2_array	RNA			
IM010	IM010_IDO1_VN1203_2d_3	Mus musculus	IM010_IDO1_VN1203_2d_3_Lung	Tissue	IM010-R	IM010_IDO1_VN1203_2d_3_array	RNA			
IM010	IM010_IDO1_VN1203_6d_1	Mus musculus	IM010_IDO1_VN1203_6d_1_Lung	Tissue	IM010-R	IM010_IDO1_VN1203_6d_1_array	RNA			
IM010	IM010_IDO1_VN1203_6d_2	Mus musculus	IM010_IDO1_VN1203_6d_2_Lung	Tissue	IM010-R	IM010_IDO1_VN1203_6d_2_array	RNA			
IM010	IM010_Tnfrsf1b_Mock_2d_1	Mus musculus	IM010_Tnfrsf1b_Mock_2d_1_Lung	Tissue	IM010-R	IM010_Tnfrsf1b_Mock_2d_1_array	RNA			
IM010	IM010_Tnfrsf1b_Mock_2d_2	Mus musculus	IM010_Tnfrsf1b_Mock_2d_2_Lung	Tissue	IM010-R	IM010_Tnfrsf1b_Mock_2d_2_array	RNA			
IM010	IM010_Tnfrsf1b_Mock_2d_3	Mus musculus	IM010_Tnfrsf1b_Mock_2d_3_Lung	Tissue	IM010-R	IM010_Tnfrsf1b_Mock_2d_3_array	RNA			
IM010	IM010_Tnfrsf1b_Mock_6d_1	Mus musculus	IM010_Tnfrsf1b_Mock_6d_1_Lung	Tissue	IM010-R	IM010_Tnfrsf1b_Mock_6d_1_array	RNA			
IM010	IM010_Tnfrsf1b_Mock_6d_2	Mus musculus	IM010_Tnfrsf1b_Mock_6d_2_Lung	Tissue	IM010-R	IM010_Tnfrsf1b_Mock_6d_2_array	RNA			
IM010	IM010_Tnfrsf1b_Mock_6d_3	Mus musculus	IM010_Tnfrsf1b_Mock_6d_3_Lung	Tissue	IM010-R	IM010_Tnfrsf1b_Mock_6d_3_array	RNA			
IM010	IM010_Tnfrsf1b_VN1203_2d_1	Mus musculus	IM010_Tnfrsf1b_VN1203_2d_1_Lung	Tissue	IM010-R	IM010_Tnfrsf1b_VN1203_2d_1_array	RNA			
IM010	IM010_Tnfrsf1b_VN1203_2d_2	Mus musculus	IM010_Tnfrsf1b_VN1203_2d_2_Lung	Tissue	IM010-R	IM010_Tnfrsf1b_VN1203_2d_2_array	RNA			
IM010	IM010_Tnfrsf1b_VN1203_2d_3	Mus musculus	IM010_Tnfrsf1b_VN1203_2d_3_Lung	Tissue	IM010-R	IM010_Tnfrsf1b_VN1203_2d_3_array	RNA			
IM010	IM010_Tnfrsf1b_VN1203_6d_1	Mus musculus	IM010_Tnfrsf1b_VN1203_6d_1_Lung	Tissue	SBRI_AA_E1	IM010_Tnfrsf1b_VN1203_6d_1_array	RNA			
IM010	IM010_Tnfrsf1b_VN1203_6d_2	Mus musculus	IM010_Tnfrsf1b_VN1203_6d_2_Lung	Tissue	SBRI_AA_E1	IM010_Tnfrsf1b_VN1203_6d_2_array	RNA			
SBRI_AA	AA001_Mock	Mus musculus	AA001_Mock_lung	Tissue	SBRI_AA_E1	AA_Mock_001	RNA			
SBRI_AA	AA002_Mock	Mus musculus	AA002_Mock_lung	Tissue	SBRI_AA_E1	AA_Mock_002	RNA			
SBRI_AA	AA003_Mock	Mus musculus	AA003_Mock_lung	Tissue	SBRI_AA_E1	AA_Mock_003	RNA			
SBRI_AA	AA005_Mock	Mus musculus	AA005_Mock_lung	Tissue	SBRI_AA_E1	AA_Mock_005	RNA			
SBRI_AA	AA006_PR8	Mus musculus	AA006_PR8_lung	Tissue	SBRI_AA_E1	AA_PR8_006	RNA			
SBRI_AA	AA007_PR8	Mus musculus	AA007_PR8_lung	Tissue	SBRI_AA_E1	AA_PR8_007	RNA			
SBRI_AA	AA008_PR8	Mus musculus	AA008_PR8_lung	Tissue	SBRI_AA_E1	AA_PR8_008	RNA			
SBRI_AA	AA009_PR8	Mus musculus	AA009_PR8_lung	Tissue	SBRI_AA_E1	AA_PR8_009	RNA			
SBRI_AA	AA010_PR8	Mus musculus	AA010_PR8_lung	Tissue	SBRI_AA_E1	AA_PR8_010	RNA			
SBRI_AA	AA011_X31	Mus musculus	AA011_X31_lung	Tissue	SBRI_AA_E1	AA_VN6+2_016	RNA			
SBRI_AA	AA012_X31	Mus musculus	AA012_X31_lung	Tissue	SBRI_AA_E1	AA_VN6+2_017	RNA			
SBRI_AA	AA013_X31	Mus musculus	AA013_X31_lung	Tissue	SBRI_AA_E1	AA_VN6+2_018	RNA			
SBRI_AA	AA014_X31	Mus musculus	AA014_X31_lung	Tissue	SBRI_AA_E1	AA_VN6+2_019	RNA			
SBRI_AA	AA015_X31	Mus musculus	AA015_X31_lung	Tissue	SBRI_AA_E1	AA_VN6+2_020	RNA			
SBRI_AA	AA016_VN6+2	Mus musculus	AA016_VN6+2_lung	Tissue	SBRI_AA_E1	AA_X31_011	RNA			
SBRI_AA	AA017_VN6+2	Mus musculus	AA017_VN6+2_lung	Tissue	SBRI_AA_E1	AA_X31_012	RNA			
SBRI_AA	AA018_VN6+2	Mus musculus	AA018_VN6+2_lung	Tissue	SBRI_AA_E1	AA_X31_013	RNA			
SBRI_AA	AA019_VN6+2	Mus musculus	AA019_VN6+2_lung	Tissue	SCL005-R	AA_X31_014	RNA			
SBRI_AA	AA020_VN6+2	Mus musculus	AA020_VN6+2_lung	Tissue	SCL005-R	AA_X31_015	RNA			
SCL005	SCL005 2B-4 Cells	Homo sapiens	SCL005_DORF6_0H_1_cell	Cells	SCL005-R	SCL005_DORF6_0H_1_RNA_ExpSam	RNA	SCL005-P	SCL005_dORF6_0h_1_proteomics	Protein
SCL005	SCL005 2B-4 Cells	Homo sapiens	SCL005_DORF6_0H_2_cell	Cells	SCL005-R	SCL005_DORF6_0H_2_RNA_ExpSam	RNA	SCL005-P	SCL005_dORF6_0h_2_proteomics	Protein
SCL005	SCL005 2B-4 Cells	Homo sapiens	SCL005_DORF6_0H_3_cell	Cells	SCL005-R	SCL005_DORF6_0H_3_RNA_ExpSam	RNA	SCL005-P	SCL005_dORF6_0h_3_proteomics	Protein
SCL005	SCL005 2B-4 Cells	Homo sapiens	SCL005_DORF6_12H_1_cell	Cells	SCL005-R	SCL005_DORF6_12H_1_RNA_ExpSam	RNA	SCL005-P	SCL005_dORF6_12h_1_proteomics	Protein
SCL005	SCL005 2B-4 Cells	Homo sapiens	SCL005_DORF6_12H_2_cell	Cells	SCL005-R	SCL005_DORF6_12H_2_RNA_ExpSam	RNA	SCL005-P	SCL005_dORF6_12h_2_proteomics	Protein
SCL005	SCL005 2B-4 Cells	Homo sapiens	SCL005_DORF6_12H_3_cell	Cells	SCL005-R	SCL005_DORF6_12H_3_RNA_ExpSam	RNA	SCL005-P	SCL005_dORF6_12h_3_proteomics	Protein
SCL005	SCL005 2B-4 Cells	Homo sapiens	SCL005_DORF6_24H_1_cell	Cells	SCL005-R	SCL005_DORF6_24H_1_RNA_ExpSam	RNA	SCL005-P	SCL005_dORF6_24h_1_proteomics	Protein
SCL005	SCL005 2B-4 Cells	Homo sapiens	SCL005_DORF6_24H_2_cell	Cells	SCL005-R	SCL005_DORF6_24H_2_RNA_ExpSam	RNA	SCL005-P	SCL005_dORF6_24h_2_proteomics	Protein
SCL005	SCL005 2B-4 Cells	Homo sapiens	SCL005_DORF6_24H_3_cell	Cells	SCL005-R	SCL005_DORF6_24H_3_RNA_ExpSam	RNA	SCL005-P	SCL005_dORF6_24h_3_proteomics	Protein
SCL005	SCL005 2B-4 Cells	Homo sapiens	SCL005_DORF6_30H_1_cell	Cells	SCL005-R	SCL005_DORF6_30H_1_RNA_ExpSam	RNA	SCL005-P	SCL005_dORF6_30h_1_proteomics	Protein
SCL005	SCL005 2B-4 Cells	Homo sapiens	SCL005_DORF6_30H_2_cell	Cells	SCL005-R	SCL005_DORF6_30H_2_RNA_ExpSam	RNA	SCL005-P	SCL005_dORF6_30h_2_proteomics	Protein
SCL005	SCL005 2B-4 Cells	Homo sapiens	SCL005_DORF6_30H_3_cell	Cells	SCL005-R	SCL005_DORF6_30H_3_RNA_ExpSam	RNA	SCL005-P	SCL005_dORF6_30h_3_proteomics	Protein
SCL005	SCL005 2B-4 Cells	Homo sapiens	SCL005_DORF6_36H_1_cell	Cells	SCL005-R	SCL005_DORF6_36H_1_RNA_ExpSam	RNA	SCL005-P	SCL005_dORF6_36h_1_proteomics	Protein
SCL005	SCL005 2B-4 Cells	Homo sapiens	SCL005_DORF6_36H_2_cell	Cells	SCL005-R	SCL005_DORF6_36H_2_RNA_ExpSam	RNA	SCL005-P	SCL005_dORF6_36h_2_proteomics	Protein
SCL005	SCL005 2B-4 Cells	Homo sapiens	SCL005_DORF6_36H_3_cell	Cells	SCL005-R	SCL005_DORF6_36H_3_RNA_ExpSam	RNA	SCL005-P	SCL005_dORF6_36h_3_proteomics	Protein
SCL005	SCL005 2B-4 Cells	Homo sapiens	SCL005_DORF6_3H_1_cell	Cells	SCL005-R	SCL005_DORF6_3H_1_RNA_ExpSam	RNA	SCL005-P	SCL005_dORF6_3h_1_proteomics	Protein
SCL005	SCL005 2B-4 Cells	Homo sapiens	SCL005_DORF6_3H_2_cell	Cells	SCL005-R	SCL005_DORF6_3H_2_RNA_ExpSam	RNA	SCL005-P	SCL005_dORF6_3h_2_proteomics	Protein
SCL005	SCL005 2B-4 Cells	Homo sapiens	SCL005_DORF6_3H_3_cell	Cells	SCL005-R	SCL005_DORF6_3H_3_RNA_ExpSam	RNA	SCL005-P	SCL005_dORF6_3h_3_proteomics	Protein
SCL005	SCL005 2B-4 Cells	Homo sapiens	SCL005_DORF6_48H_1_cell	Cells	SCL005-R	SCL005_DORF6_48H_1_RNA_ExpSam	RNA	SCL005-P	SCL005_dORF6_48h_1_proteomics	Protein
SCL005	SCL005 2B-4 Cells	Homo sapiens	SCL005_DORF6_48H_2_cell	Cells	SCL005-R	SCL005_DORF6_48H_2_RNA_ExpSam	RNA	SCL005-P	SCL005_dORF6_48h_2_proteomics	Protein
SCL005	SCL005 2B-4 Cells	Homo sapiens	SCL005_DORF6_48H_3_cell	Cells	SCL005-R	SCL005_DORF6_48H_3_RNA_ExpSam	RNA	SCL005-P	SCL005_dORF6_48h_3_proteomics	Protein
SCL005	SCL005 2B-4 Cells	Homo sapiens	SCL005_DORF6_54H_1_cell	Cells	SCL005-R	SCL005_DORF6_54H_1_RNA_ExpSam	RNA	SCL005-P	SCL005_dORF6_54h_1_proteomics	Protein
SCL005	SCL005 2B-4 Cells	Homo sapiens	SCL005_DORF6_54H_2_cell	Cells	SCL005-R	SCL005_DORF6_54H_2_RNA_ExpSam	RNA	SCL005-P	SCL005_dORF6_54h_2_proteomics	Protein
SCL005	SCL005 2B-4 Cells	Homo sapiens	SCL005_DORF6_54H_3_cell	Cells	SCL005-R	SCL005_DORF6_54H_3_RNA_ExpSam	RNA			
SCL005	SCL005 2B-4 Cells	Homo sapiens	SCL005_DORF6_60H_1_cell	Cells	SCL005-R	SCL005_DORF6_60H_1_RNA_ExpSam	RNA	SCL005-P	SCL005_dORF6_60h_1_proteomics	Protein
SCL005	SCL005 2B-4 Cells	Homo sapiens	SCL005_DORF6_60H_2_cell	Cells	SCL005-R	SCL005_DORF6_60H_2_RNA_ExpSam	RNA	SCL005-P	SCL005_dORF6_60h_2_proteomics	Protein
SCL005	SCL005 2B-4 Cells	Homo sapiens	SCL005_DORF6_60H_3_cell	Cells	SCL005-R	SCL005_DORF6_60H_3_RNA_ExpSam	RNA	SCL005-P	SCL005_dORF6_60h_3_proteomics	Protein
SCL005	SCL005 2B-4 Cells	Homo sapiens	SCL005_DORF6_72H_1_cell	Cells	SCL005-R	SCL005_DORF6_72H_1_RNA_ExpSam	RNA	SCL005-P	SCL005_dORF6_72h_1_proteomics	Protein
SCL005	SCL005 2B-4 Cells	Homo sapiens	SCL005_DORF6_72H_2_cell	Cells	SCL005-R	SCL005_DORF6_72H_2_RNA_ExpSam	RNA	SCL005-P	SCL005_dORF6_72h_2_proteomics	Protein
SCL005	SCL005 2B-4 Cells	Homo sapiens	SCL005_DORF6_72H_3_cell	Cells	SCL005-R	SCL005_DORF6_72H_3_RNA_ExpSam	RNA	SCL005-P	SCL005_dORF6_72h_3_proteomics	Protein
SCL005	SCL005 2B-4 Cells	Homo sapiens	SCL005_DORF6_7H_1_cell	Cells	SCL005-R	SCL005_DORF6_7H_1_RNA_ExpSam	RNA	SCL005-P	SCL005_dORF6_7h_1_proteomics	Protein
SCL005	SCL005 2B-4 Cells	Homo sapiens	SCL005_DORF6_7H_2_cell	Cells	SCL005-R	SCL005_DORF6_7H_2_RNA_ExpSam	RNA	SCL005-P	SCL005_dORF6_7h_2_proteomics	Protein
SCL005	SCL005 2B-4 Cells	Homo sapiens	SCL005_DORF6_7H_3_cell	Cells	SCL005-R	SCL005_DORF6_7H_3_RNA_ExpSam	RNA	SCL005-P	SCL005_dORF6_7h_3_proteomics	Protein
SCL005	SCL005 2B-4 Cells	Homo sapiens	SCL005_mock_0H_1_cell	Cells	SCL005-R	SCL005_mock_0H_1_RNA_ExpSam	RNA	SCL005-P	SCL005_Mock_0h_1_proteomics	Protein
SCL005	SCL005 2B-4 Cells	Homo sapiens	SCL005_mock_0H_2_cell	Cells	SCL005-R	SCL005_mock_0H_2_RNA_ExpSam	RNA	SCL005-P	SCL005_Mock_0h_2_proteomics	Protein
SCL005	SCL005 2B-4 Cells	Homo sapiens	SCL005_mock_0H_3_cell	Cells	SCL005-R	SCL005_mock_0H_3_RNA_ExpSam	RNA	SCL005-P	SCL005_Mock_0h_3_proteomics	Protein
SCL005	SCL005 2B-4 Cells	Homo sapiens	SCL005_mock_12H_1_cell	Cells	SCL005-R	SCL005_mock_12H_1_RNA_ExpSam	RNA	SCL005-P	SCL005_Mock_12h_1_proteomics	Protein
SCL005	SCL005 2B-4 Cells	Homo sapiens	SCL005_mock_12H_2_cell	Cells	SCL005-R	SCL005_mock_12H_2_RNA_ExpSam	RNA	SCL005-P	SCL005_Mock_12h_2_proteomics	Protein
SCL005	SCL005 2B-4 Cells	Homo sapiens	SCL005_mock_12H_3_cell	Cells	SCL005-R	SCL005_mock_12H_3_RNA_ExpSam	RNA	SCL005-P	SCL005_Mock_12h_3_proteomics	Protein
SCL005	SCL005 2B-4 Cells	Homo sapiens	SCL005_mock_24H_1_cell	Cells	SCL005-R	SCL005_mock_24H_1_RNA_ExpSam	RNA	SCL005-P	SCL005_Mock_24h_1_proteomics	Protein
SCL005	SCL005 2B-4 Cells	Homo sapiens	SCL005_mock_24H_2_cell	Cells	SCL005-R	SCL005_mock_24H_2_RNA_ExpSam	RNA	SCL005-P	SCL005_Mock_24h_2_proteomics	Protein
SCL005	SCL005 2B-4 Cells	Homo sapiens	SCL005_mock_24H_3_cell	Cells	SCL005-R	SCL005_mock_24H_3_RNA_ExpSam	RNA	SCL005-P	SCL005_Mock_24h_3_proteomics	Protein
SCL005	SCL005 2B-4 Cells	Homo sapiens	SCL005_mock_30H_1_cell	Cells	SCL005-R	SCL005_mock_30H_1_RNA_ExpSam	RNA	SCL005-P	SCL005_Mock_30h_1_proteomics	Protein
SCL005	SCL005 2B-4 Cells	Homo sapiens	SCL005_mock_30H_2_cell	Cells	SCL005-R	SCL005_mock_30H_2_RNA_ExpSam	RNA	SCL005-P	SCL005_Mock_30h_2_proteomics	Protein
SCL005	SCL005 2B-4 Cells	Homo sapiens	SCL005_mock_30H_3_cell	Cells	SCL005-R	SCL005_mock_30H_3_RNA_ExpSam	RNA	SCL005-P	SCL005_Mock_30h_3_proteomics	Protein
SCL005	SCL005 2B-4 Cells	Homo sapiens	SCL005_mock_36H_1_cell	Cells	SCL005-R	SCL005_mock_36H_1_RNA_ExpSam	RNA	SCL005-P	SCL005_Mock_36h_1_proteomics	Protein
SCL005	SCL005 2B-4 Cells	Homo sapiens	SCL005_mock_36H_2_cell	Cells	SCL005-R	SCL005_mock_36H_2_RNA_ExpSam	RNA	SCL005-P	SCL005_Mock_36h_2_proteomics	Protein
SCL005	SCL005 2B-4 Cells	Homo sapiens	SCL005_mock_36H_3_cell	Cells	SCL005-R	SCL005_mock_36H_3_RNA_ExpSam	RNA	SCL005-P	SCL005_Mock_36h_3_proteomics	Protein
SCL005	SCL005 2B-4 Cells	Homo sapiens	SCL005_mock_3H_1_cell	Cells	SCL005-R	SCL005_mock_3H_1_RNA_ExpSam	RNA	SCL005-P	SCL005_Mock_3h_1_proteomics	Protein
SCL005	SCL005 2B-4 Cells	Homo sapiens	SCL005_mock_3H_2_cell	Cells	SCL005-R	SCL005_mock_3H_2_RNA_ExpSam	RNA	SCL005-P	SCL005_Mock_3h_2_proteomics	Protein
SCL005	SCL005 2B-4 Cells	Homo sapiens	SCL005_mock_3H_3_cell	Cells	SCL005-R	SCL005_mock_3H_3_RNA_ExpSam	RNA	SCL005-P	SCL005_Mock_3h_3_proteomics	Protein
SCL005	SCL005 2B-4 Cells	Homo sapiens	SCL005_mock_48H_1_cell	Cells	SCL005-R	SCL005_mock_48H_1_RNA_ExpSam	RNA	SCL005-P	SCL005_Mock_48h_1_proteomics	Protein
SCL005	SCL005 2B-4 Cells	Homo sapiens	SCL005_mock_48H_2_cell	Cells	SCL005-R	SCL005_mock_48H_2_RNA_ExpSam	RNA	SCL005-P	SCL005_Mock_48h_2_proteomics	Protein
SCL005	SCL005 2B-4 Cells	Homo sapiens	SCL005_mock_48H_3_cell	Cells	SCL005-R	SCL005_mock_48H_3_RNA_ExpSam	RNA	SCL005-P	SCL005_Mock_48h_3_proteomics	Protein
SCL005	SCL005 2B-4 Cells	Homo sapiens	SCL005_mock_54H_1_cell	Cells	SCL005-R	SCL005_mock_54H_1_RNA_ExpSam	RNA	SCL005-P	SCL005_Mock_54h_1_proteomics	Protein
SCL005	SCL005 2B-4 Cells	Homo sapiens	SCL005_mock_54H_2_cell	Cells	SCL005-R	SCL005_mock_54H_2_RNA_ExpSam	RNA	SCL005-P	SCL005_Mock_54h_2_proteomics	Protein
SCL005	SCL005 2B-4 Cells	Homo sapiens	SCL005_mock_54H_3_cell	Cells	SCL005-R	SCL005_mock_54H_3_RNA_ExpSam	RNA	SCL005-P	SCL005_Mock_54h_3_proteomics	Protein
SCL005	SCL005 2B-4 Cells	Homo sapiens	SCL005_mock_60H_1_cell	Cells	SCL005-R	SCL005_mock_60H_1_RNA_ExpSam	RNA	SCL005-P	SCL005_Mock_60h_1_proteomics	Protein
SCL005	SCL005 2B-4 Cells	Homo sapiens	SCL005_mock_60H_2_cell	Cells	SCL005-R	SCL005_mock_60H_2_RNA_ExpSam	RNA	SCL005-P	SCL005_Mock_60h_2_proteomics	Protein
SCL005	SCL005 2B-4 Cells	Homo sapiens	SCL005_mock_60H_3_cell	Cells	SCL005-R	SCL005_mock_60H_3_RNA_ExpSam	RNA	SCL005-P	SCL005_Mock_60h_3_proteomics	Protein
SCL005	SCL005 2B-4 Cells	Homo sapiens	SCL005_mock_72H_1_cell	Cells	SCL005-R	SCL005_mock_72H_1_RNA_ExpSam	RNA	SCL005-P	SCL005_Mock_72h_1_proteomics	Protein
SCL005	SCL005 2B-4 Cells	Homo sapiens	SCL005_mock_72H_2_cell	Cells	SCL005-R	SCL005_mock_72H_2_RNA_ExpSam	RNA	SCL005-P	SCL005_Mock_72h_2_proteomics	Protein
SCL005	SCL005 2B-4 Cells	Homo sapiens	SCL005_mock_72H_3_cell	Cells	SCL005-R	SCL005_mock_72H_3_RNA_ExpSam	RNA	SCL005-P	SCL005_Mock_72h_3_proteomics	Protein
SCL005	SCL005 2B-4 Cells	Homo sapiens	SCL005_mock_7H_1_cell	Cells	SCL005-R	SCL005_mock_7H_1_RNA_ExpSam	RNA	SCL005-P	SCL005_Mock_7h_1_proteomics	Protein
SCL005	SCL005 2B-4 Cells	Homo sapiens	SCL005_mock_7H_2_cell	Cells	SCL005-R	SCL005_mock_7H_2_RNA_ExpSam	RNA	SCL005-P	SCL005_Mock_7h_2_proteomics	Protein
SCL005	SCL005 2B-4 Cells	Homo sapiens	SCL005_mock_7H_3_cell	Cells	SCL005-R	SCL005_mock_7H_3_RNA_ExpSam	RNA			Protein
SCL005	SCL005 2B-4 Cells	Homo sapiens	SCL005_WT_0H_1_cell	Cells	SCL005-R	SCL005_WT_0H_1_RNA_ExpSam	RNA	SCL005-P	SCL005_icSARS_0h_1_proteomics	Protein
SCL005	SCL005 2B-4 Cells	Homo sapiens	SCL005_WT_0H_2_cell	Cells	SCL005-R	SCL005_WT_0H_2_RNA_ExpSam	RNA	SCL005-P	SCL005_icSARS_0h_2_proteomics	Protein
SCL005	SCL005 2B-4 Cells	Homo sapiens	SCL005_WT_0H_3_cell	Cells	SCL005-R	SCL005_WT_0H_3_RNA_ExpSam	RNA	SCL005-P	SCL005_icSARS_0h_3_proteomics	Protein
SCL005	SCL005 2B-4 Cells	Homo sapiens	SCL005_WT_12H_1_cell	Cells	SCL005-R	SCL005_WT_12H_1_RNA_ExpSam	RNA	SCL005-P	SCL005_icSARS_12h_1_proteomics	Protein
SCL005	SCL005 2B-4 Cells	Homo sapiens	SCL005_WT_12H_2_cell	Cells	SCL005-R	SCL005_WT_12H_2_RNA_ExpSam	RNA	SCL005-P	SCL005_icSARS_12h_2_proteomics	Protein
SCL005	SCL005 2B-4 Cells	Homo sapiens	SCL005_WT_12H_3_cell	Cells	SCL005-R	SCL005_WT_12H_3_RNA_ExpSam	RNA			
SCL005	SCL005 2B-4 Cells	Homo sapiens	SCL005_WT_24H_1_cell	Cells	SCL005-R	SCL005_WT_24H_1_RNA_ExpSam	RNA	SCL005-P	SCL005_icSARS_24h_1_proteomics	Protein
SCL005	SCL005 2B-4 Cells	Homo sapiens	SCL005_WT_24H_2_cell	Cells	SCL005-R	SCL005_WT_24H_2_RNA_ExpSam	RNA	SCL005-P	SCL005_icSARS_24h_2_proteomics	Protein
SCL005	SCL005 2B-4 Cells	Homo sapiens	SCL005_WT_24H_3_cell	Cells	SCL005-R	SCL005_WT_24H_3_RNA_ExpSam	RNA	SCL005-P	SCL005_icSARS_24h_3_proteomics	Protein
SCL005	SCL005 2B-4 Cells	Homo sapiens	SCL005_WT_30H_1_cell	Cells	SCL005-R	SCL005_WT_30H_1_RNA_ExpSam	RNA	SCL005-P	SCL005_icSARS_30h_1_proteomics	Protein
SCL005	SCL005 2B-4 Cells	Homo sapiens	SCL005_WT_30H_2_cell	Cells	SCL005-R	SCL005_WT_30H_2_RNA_ExpSam	RNA	SCL005-P	SCL005_icSARS_30h_2_proteomics	Protein
SCL005	SCL005 2B-4 Cells	Homo sapiens	SCL005_WT_30H_3_cell	Cells	SCL005-R	SCL005_WT_30H_3_RNA_ExpSam	RNA	SCL005-P	SCL005_icSARS_30h_3_proteomics	Protein
SCL005	SCL005 2B-4 Cells	Homo sapiens	SCL005_WT_36H_1_cell	Cells	SCL005-R	SCL005_WT_36H_1_RNA_ExpSam	RNA	SCL005-P	SCL005_icSARS_36h_1_proteomics	Protein
SCL005	SCL005 2B-4 Cells	Homo sapiens	SCL005_WT_36H_2_cell	Cells	SCL005-R	SCL005_WT_36H_2_RNA_ExpSam	RNA	SCL005-P	SCL005_icSARS_36h_2_proteomics	Protein
SCL005	SCL005 2B-4 Cells	Homo sapiens	SCL005_WT_36H_3_cell	Cells	SCL005-R	SCL005_WT_36H_3_RNA_ExpSam	RNA	SCL005-P	SCL005_icSARS_36h_3_proteomics	Protein
SCL005	SCL005 2B-4 Cells	Homo sapiens	SCL005_WT_3H_1_cell	Cells	SCL005-R	SCL005_WT_3H_1_RNA_ExpSam	RNA	SCL005-P	SCL005_icSARS_3h_1_proteomics	Protein
SCL005	SCL005 2B-4 Cells	Homo sapiens	SCL005_WT_3H_2_cell	Cells	SCL005-R	SCL005_WT_3H_2_RNA_ExpSam	RNA	SCL005-P	SCL005_icSARS_3h_2_proteomics	Protein
SCL005	SCL005 2B-4 Cells	Homo sapiens	SCL005_WT_3H_3_cell	Cells	SCL005-R	SCL005_WT_3H_3_RNA_ExpSam	RNA	SCL005-P	SCL005_icSARS_3h_3_proteomics	Protein
SCL005	SCL005 2B-4 Cells	Homo sapiens	SCL005_WT_48H_1_cell	Cells	SCL005-R	SCL005_WT_48H_1_RNA_ExpSam	RNA	SCL005-P	SCL005_icSARS_48h_1_proteomics	Protein
SCL005	SCL005 2B-4 Cells	Homo sapiens	SCL005_WT_48H_2_cell	Cells	SCL005-R	SCL005_WT_48H_2_RNA_ExpSam	RNA	SCL005-P	SCL005_icSARS_48h_2_proteomics	Protein
SCL005	SCL005 2B-4 Cells	Homo sapiens	SCL005_WT_48H_3_cell	Cells	SCL005-R	SCL005_WT_48H_3_RNA_ExpSam	RNA	SCL005-P	SCL005_icSARS_48h_3_proteomics	Protein
SCL005	SCL005 2B-4 Cells	Homo sapiens	SCL005_WT_54H_1_cell	Cells	SCL005-R	SCL005_WT_54H_1_RNA_ExpSam	RNA	SCL005-P	SCL005_icSARS_54h_1_proteomics	Protein
SCL005	SCL005 2B-4 Cells	Homo sapiens	SCL005_WT_54H_2_cell	Cells	SCL005-R	SCL005_WT_54H_2_RNA_ExpSam	RNA	SCL005-P	SCL005_icSARS_54h_2_proteomics	Protein
SCL005	SCL005 2B-4 Cells	Homo sapiens	SCL005_WT_54H_3_cell	Cells	SCL005-R	SCL005_WT_54H_3_RNA_ExpSam	RNA	SCL005-P	SCL005_icSARS_54h_3_proteomics	Protein
SCL005	SCL005 2B-4 Cells	Homo sapiens	SCL005_WT_60H_1_cell	Cells	SCL005-R	SCL005_WT_60H_1_RNA_ExpSam	RNA	SCL005-P	SCL005_icSARS_60h_1_proteomics	Protein
SCL005	SCL005 2B-4 Cells	Homo sapiens	SCL005_WT_60H_2_cell	Cells	SCL005-R	SCL005_WT_60H_2_RNA_ExpSam	RNA	SCL005-P	SCL005_icSARS_60h_2_proteomics	Protein
SCL005	SCL005 2B-4 Cells	Homo sapiens	SCL005_WT_60H_3_cell	Cells	SCL005-R	SCL005_WT_60H_3_RNA_ExpSam	RNA	SCL005-P	SCL005_icSARS_60h_3_proteomics	Protein
SCL005	SCL005 2B-4 Cells	Homo sapiens	SCL005_WT_72H_1_cell	Cells	SCL005-R	SCL005_WT_72H_1_RNA_ExpSam	RNA	SCL005-P	SCL005_icSARS_72h_1_proteomics	Protein
SCL005	SCL005 2B-4 Cells	Homo sapiens	SCL005_WT_72H_2_cell	Cells	SCL005-R	SCL005_WT_72H_2_RNA_ExpSam	RNA	SCL005-P	SCL005_icSARS_72h_2_proteomics	Protein
SCL005	SCL005 2B-4 Cells	Homo sapiens	SCL005_WT_72H_3_cell	Cells	SCL005-R	SCL005_WT_72H_3_RNA_ExpSam	RNA	SCL005-P	SCL005_icSARS_72h_3_proteomics	Protein
SCL005	SCL005 2B-4 Cells	Homo sapiens	SCL005_WT_7H_1_cell	Cells	SCL005-R	SCL005_WT_7H_1_RNA_ExpSam	RNA	SCL005-P	SCL005_icSARS_7h_1_proteomics	Protein
SCL005	SCL005 2B-4 Cells	Homo sapiens	SCL005_WT_7H_2_cell	Cells	SCL006-R	SCL005_WT_7H_2_RNA_ExpSam	RNA	SCL006-P	SCL005_icSARS_7h_2_proteomics	Protein
SCL005	SCL005 2B-4 Cells	Homo sapiens	SCL005_WT_7H_3_cell	Cells	SCL006-R	SCL005_WT_7H_3_RNA_ExpSam	RNA			Protein
SCL006	SCL006 2B-4 Cells	Homo sapiens	SCL006_BatSRBD_0h_1_cell	Cells	SCL006-R	SCL006_BatSRBD_0h_1_RNA_ExpSam	RNA	SCL006-P	SCL006_BatSRBD_0h_1_proteomics	Protein
SCL006	SCL006 2B-4 Cells	Homo sapiens	SCL006_BatSRBD_0h_2_cell	Cells	SCL006-R	SCL006_BatSRBD_0h_2_RNA_ExpSam	RNA	SCL006-P	SCL006_BatSRBD_0h_2_proteomics	Protein
SCL006	SCL006 2B-4 Cells	Homo sapiens	SCL006_BatSRBD_0h_3_cell	Cells	SCL006-R	SCL006_BatSRBD_0h_3_RNA_ExpSam	RNA	SCL006-P	SCL006_BatSRBD_0h_3_proteomics	Protein
SCL006	SCL006 2B-4 Cells	Homo sapiens	SCL006_BatSRBD_3h_1_Cells	Cells				SCL006-P	SCL006_BatSRBD_7h_1_proteomics	Protein
SCL006	SCL006 2B-4 Cells	Homo sapiens	SCL006_BatSRBD_3h_2_Cells	Cells				SCL006-P	SCL006_BatSRBD_7h_2_proteomics	Protein
SCL006	SCL006 2B-4 Cells	Homo sapiens	SCL006_BatSRBD_3h_3_Cells	Cells				SCL006-P	SCL006_BatSRBD_7h_3_proteomics	Protein
SCL006	SCL006 2B-4 Cells	Homo sapiens	SCL006_BatSRBD_7h_1_Cells	Cells				SCL006-P	SCL006_BatSRBD_3h_1_proteomics	Protein
SCL006	SCL006 2B-4 Cells	Homo sapiens	SCL006_BatSRBD_7h_2_Cells	Cells				SCL006-P	SCL006_BatSRBD_3h_2_proteomics	Protein
SCL006	SCL006 2B-4 Cells	Homo sapiens	SCL006_BatSRBD_7h_3_Cells	Cells				SCL006-P	SCL006_BatSRBD_3h_3_proteomics	Protein
SCL006	SCL006 2B-4 Cells	Homo sapiens	SCL006_BatSRBD_12h_1_cell	Cells	SCL006-R	SCL006_BatSRBD_12h_1_RNA_ExpSam	RNA	SCL006-P	SCL006_BatSRBD_12h_1_proteomics	Protein
SCL006	SCL006 2B-4 Cells	Homo sapiens	SCL006_BatSRBD_12h_2_cell	Cells	SCL006-R	SCL006_BatSRBD_12h_2_RNA_ExpSam	RNA	SCL006-P	SCL006_BatSRBD_12h_2_proteomics	Protein
SCL006	SCL006 2B-4 Cells	Homo sapiens	SCL006_BatSRBD_12h_3_cell	Cells	SCL006-R	SCL006_BatSRBD_12h_3_RNA_ExpSam	RNA	SCL006-P	SCL006_BatSRBD_12h_3_proteomics	Protein
SCL006	SCL006 2B-4 Cells	Homo sapiens	SCL006_BatSRBD_24h_1_cell	Cells	SCL006-R	SCL006_BatSRBD_24h_1_RNA_ExpSam	RNA	SCL006-P	SCL006_BatSRBD_24h_1_proteomics	Protein
SCL006	SCL006 2B-4 Cells	Homo sapiens	SCL006_BatSRBD_24h_2_cell	Cells	SCL006-R	SCL006_BatSRBD_24h_2_RNA_ExpSam	RNA	SCL006-P	SCL006_BatSRBD_24h_2_proteomics	Protein
SCL006	SCL006 2B-4 Cells	Homo sapiens	SCL006_BatSRBD_24h_3_cell	Cells	SCL006-R	SCL006_BatSRBD_24h_3_RNA_ExpSam	RNA	SCL006-P	SCL006_BatSRBD_24h_3_proteomics	Protein
SCL006	SCL006 2B-4 Cells	Homo sapiens	SCL006_BatSRBD_30h_1_cell	Cells	SCL006-R	SCL006_BatSRBD_30h_1_RNA_ExpSam	RNA			
SCL006	SCL006 2B-4 Cells	Homo sapiens	SCL006_BatSRBD_30h_2_cell	Cells	SCL006-R	SCL006_BatSRBD_30h_2_RNA_ExpSam	RNA	SCL006-P	SCL006_BatSRBD_30h_2_proteomics	Protein
SCL006	SCL006 2B-4 Cells	Homo sapiens	SCL006_BatSRBD_30h_3_cell	Cells	SCL006-R	SCL006_BatSRBD_30h_3_RNA_ExpSam	RNA	SCL006-P	SCL006_BatSRBD_30h_3_proteomics	Protein
SCL006	SCL006 2B-4 Cells	Homo sapiens	SCL006_BatSRBD_36h_1_cell	Cells	SCL006-R	SCL006_BatSRBD_36h_1_RNA_ExpSam	RNA	SCL006-P	SCL006_BatSRBD_36h_1_proteomics	Protein
SCL006	SCL006 2B-4 Cells	Homo sapiens	SCL006_BatSRBD_36h_2_cell	Cells	SCL006-R	SCL006_BatSRBD_36h_2_RNA_ExpSam	RNA	SCL006-P	SCL006_BatSRBD_36h_2_proteomics	Protein
SCL006	SCL006 2B-4 Cells	Homo sapiens	SCL006_BatSRBD_36h_3_cell	Cells	SCL006-R	SCL006_BatSRBD_36h_3_RNA_ExpSam	RNA	SCL006-P	SCL006_BatSRBD_36h_3_proteomics	Protein
SCL006	SCL006 2B-4 Cells	Homo sapiens	SCL006_BatSRBD_48h_1_B_cell	Cells	SCL006-R	SCL006_BatSRBD_48h_1_B_RNA_ExpSam	RNA			
SCL006	SCL006 2B-4 Cells	Homo sapiens	SCL006_BatSRBD_48h_2_cell	Cells	SCL006-R	SCL006_BatSRBD_48h_2_RNA_ExpSam	RNA	SCL006-P	SCL006_BatSRBD_48h_2_proteomics	Protein
SCL006	SCL006 2B-4 Cells	Homo sapiens	SCL006_BatSRBD_48h_3_cell	Cells	SCL006-R	SCL006_BatSRBD_48h_3_RNA_ExpSam	RNA	SCL006-P	SCL006_BatSRBD_48h_3_proteomics	Protein
SCL006	SCL006 2B-4 Cells	Homo sapiens	SCL006_BatSRBD_54h_1_B_cell	Cells	SCL006-R	SCL006_BatSRBD_54h_1_B_RNA_ExpSam	RNA	SCL006-P	SCL006_BatSRBD_54h_1_proteomics	Protein
SCL006	SCL006 2B-4 Cells	Homo sapiens	SCL006_BatSRBD_54h_2_cell	Cells	SCL006-R	SCL006_BatSRBD_54h_2_RNA_ExpSam	RNA	SCL006-P	SCL006_BatSRBD_54h_2_proteomics	Protein
SCL006	SCL006 2B-4 Cells	Homo sapiens	SCL006_BatSRBD_54h_3_cell	Cells	SCL006-R	SCL006_BatSRBD_54h_3_RNA_ExpSam	RNA			
SCL006	SCL006 2B-4 Cells	Homo sapiens	SCL006_BatSRBD_60h_1_cell	Cells	SCL006-R	SCL006_BatSRBD_60h_1_RNA_ExpSam	RNA	SCL006-P	SCL006_BatSRBD_60h_1_proteomics	Protein
SCL006	SCL006 2B-4 Cells	Homo sapiens	SCL006_BatSRBD_60h_2_cell	Cells	SCL006-R	SCL006_BatSRBD_60h_2_RNA_ExpSam	RNA	SCL006-P	SCL006_BatSRBD_60h_2_proteomics	Protein
SCL006	SCL006 2B-4 Cells	Homo sapiens	SCL006_BatSRBD_60h_3_cell	Cells	SCL006-R	SCL006_BatSRBD_60h_3_RNA_ExpSam	RNA	SCL006-P	SCL006_BatSRBD_60h_3_proteomics	Protein
SCL006	SCL006 2B-4 Cells	Homo sapiens	SCL006_BatSRBD_72h_1_cell	Cells	SCL006-R	SCL006_BatSRBD_72h_1_RNA_ExpSam	RNA	SCL006-P	SCL006_BatSRBD_72h_1_proteomics	Protein
SCL006	SCL006 2B-4 Cells	Homo sapiens	SCL006_BatSRBD_72h_2_cell	Cells	SCL006-R	SCL006_BatSRBD_72h_2_RNA_ExpSam	RNA	SCL006-P	SCL006_BatSRBD_72h_2_proteomics	Protein
SCL006	SCL006 2B-4 Cells	Homo sapiens	SCL006_BatSRBD_72h_3_cell	Cells	SCL006-R	SCL006_BatSRBD_72h_3_RNA_ExpSam	RNA	SCL006-P	SCL006_BatSRBD_72h_3_proteomics	Protein
SCL006	SCL006 2B-4 Cells	Homo sapiens	SCL006_icSARSCoV_0h_1_cell	Cells	SCL006-R	SCL006_icSARSCoV_0h_1_RNA_ExpSam	RNA	SCL006-P	SCL006_icSARS_0h_1_proteomics	Protein
SCL006	SCL006 2B-4 Cells	Homo sapiens	SCL006_icSARSCoV_0h_2_cell	Cells	SCL006-R	SCL006_icSARSCoV_0h_2_RNA_ExpSam	RNA	SCL006-P	SCL006_icSARS_0h_2_proteomics	Protein
SCL006	SCL006 2B-4 Cells	Homo sapiens	SCL006_icSARSCoV_0h_3_cell	Cells	SCL006-R	SCL006_icSARSCoV_0h_3_RNA_ExpSam	RNA	SCL006-P	SCL006_icSARS_0h_3_proteomics	Protein
SCL006	SCL006 2B-4 Cells	Homo sapiens	SCL006_icSARSCoV_12h_1_cell	Cells	SCL006-R	SCL006_icSARSCoV_12h_1_RNA_ExpSam	RNA	SCL006-P	SCL006_icSARS_12h_1_proteomics	Protein
SCL006	SCL006 2B-4 Cells	Homo sapiens	SCL006_icSARSCoV_12h_2_cell	Cells	SCL006-R	SCL006_icSARSCoV_12h_2_RNA_ExpSam	RNA	SCL006-P	SCL006_icSARS_12h_2_proteomics	Protein
SCL006	SCL006 2B-4 Cells	Homo sapiens	SCL006_icSARSCoV_12h_3_B_cell	Cells	SCL006-R	SCL006_icSARSCoV_12h_3_B_RNA_ExpSam	RNA	SCL006-P	SCL006_icSARS_12h_3_proteomics	Protein
SCL006	SCL006 2B-4 Cells	Homo sapiens	SCL006_icSARSCoV_24h_1_cell	Cells	SCL006-R	SCL006_icSARSCoV_24h_1_RNA_ExpSam	RNA	SCL006-P	SCL006_icSARS_24h_1_proteomics	Protein
SCL006	SCL006 2B-4 Cells	Homo sapiens	SCL006_icSARSCoV_24h_2_cell	Cells	SCL006-R	SCL006_icSARSCoV_24h_2_RNA_ExpSam	RNA	SCL006-P	SCL006_icSARS_24h_2_proteomics	Protein
SCL006	SCL006 2B-4 Cells	Homo sapiens	SCL006_icSARSCoV_24h_3_cell	Cells	SCL006-R	SCL006_icSARSCoV_24h_3_RNA_ExpSam	RNA	SCL006-P	SCL006_icSARS_24h_3_proteomics	Protein
SCL006	SCL006 2B-4 Cells	Homo sapiens	SCL006_icSARSCoV_30h_1_cell	Cells	SCL006-R	SCL006_icSARSCoV_30h_1_RNA_ExpSam	RNA			
SCL006	SCL006 2B-4 Cells	Homo sapiens	SCL006_icSARSCoV_30h_2_cell	Cells	SCL006-R	SCL006_icSARSCoV_30h_2_RNA_ExpSam	RNA	SCL006-P	SCL006_icSARS_30h_2_proteomics	Protein
SCL006	SCL006 2B-4 Cells	Homo sapiens	SCL006_icSARSCoV_30h_3_cell	Cells	SCL006-R	SCL006_icSARSCoV_30h_3_RNA_ExpSam	RNA	SCL006-P	SCL006_icSARS_30h_3_proteomics	Protein
SCL006	SCL006 2B-4 Cells	Homo sapiens	SCL006_icSARSCoV_36h_1_cell	Cells	SCL006-R	SCL006_icSARSCoV_36h_1_RNA_ExpSam	RNA	SCL006-P	SCL006_icSARS_36h_1_proteomics	Protein
SCL006	SCL006 2B-4 Cells	Homo sapiens	SCL006_icSARSCoV_36h_2_cell	Cells	SCL006-R	SCL006_icSARSCoV_36h_2_RNA_ExpSam	RNA	SCL006-P	SCL006_icSARS_36h_2_proteomics	Protein
SCL006	SCL006 2B-4 Cells	Homo sapiens	SCL006_icSARSCoV_36h_3_cell	Cells	SCL006-R	SCL006_icSARSCoV_36h_3_RNA_ExpSam	RNA	SCL006-P	SCL006_icSARS_36h_3_proteomics	Protein
SCL006	SCL006 2B-4 Cells	Homo sapiens	SCL006_icSARSCoV_48h_1_cell	Cells	SCL006-R	SCL006_icSARSCoV_48h_1_RNA_ExpSam	RNA	SCL006-P	SCL006_icSARS_48h_1_proteomics	Protein
SCL006	SCL006 2B-4 Cells	Homo sapiens	SCL006_icSARSCoV_48h_2_cell	Cells	SCL006-R	SCL006_icSARSCoV_48h_2_RNA_ExpSam	RNA	SCL006-P	SCL006_icSARS_48h_2_proteomics	Protein
SCL006	SCL006 2B-4 Cells	Homo sapiens	SCL006_icSARSCoV_48h_3_cell	Cells	SCL006-R	SCL006_icSARSCoV_48h_3_RNA_ExpSam	RNA	SCL006-P	SCL006_icSARS_48h_3_proteomics	Protein
SCL006	SCL006 2B-4 Cells	Homo sapiens	SCL006_icSARSCoV_54h_1_cell	Cells	SCL006-R	SCL006_icSARSCoV_54h_1_RNA_ExpSam	RNA	SCL006-P	SCL006_icSARS_54h_1_proteomics	Protein
SCL006	SCL006 2B-4 Cells	Homo sapiens	SCL006_icSARSCoV_54h_2_cell	Cells	SCL006-R	SCL006_icSARSCoV_54h_2_RNA_ExpSam	RNA	SCL006-P	SCL006_icSARS_54h_2_proteomics	Protein
SCL006	SCL006 2B-4 Cells	Homo sapiens	SCL006_icSARSCoV_54h_3_cell	Cells	SCL006-R	SCL006_icSARSCoV_54h_3_RNA_ExpSam	RNA	SCL006-P	SCL006_icSARS_54h_3_proteomics	Protein
SCL006	SCL006 2B-4 Cells	Homo sapiens	SCL006_icSARSCoV_60h_1_cell	Cells	SCL006-R	SCL006_icSARSCoV_60h_1_RNA_ExpSam	RNA	SCL006-P	SCL006_icSARS_60h_1_proteomics	Protein
SCL006	SCL006 2B-4 Cells	Homo sapiens	SCL006_icSARSCoV_60h_2_cell	Cells	SCL006-R	SCL006_icSARSCoV_60h_2_RNA_ExpSam	RNA	SCL006-P	SCL006_icSARS_60h_2_proteomics	Protein
SCL006	SCL006 2B-4 Cells	Homo sapiens	SCL006_icSARSCoV_60h_3_B_cell	Cells	SCL006-R	SCL006_icSARSCoV_60h_3_B_RNA_ExpSam	RNA	SCL006-P	SCL006_icSARS_60h_3_proteomics	Protein
SCL006	SCL006 2B-4 Cells	Homo sapiens	SCL006_icSARSCoV_72h_1_cell	Cells	SCL006-R	SCL006_icSARSCoV_72h_1_RNA_ExpSam	RNA	SCL006-P	SCL006_icSARS_72h_1_proteomics	Protein
SCL006	SCL006 2B-4 Cells	Homo sapiens	SCL006_icSARSCoV_72h_2_cell	Cells	SCL006-R	SCL006_icSARSCoV_72h_2_RNA_ExpSam	RNA	SCL006-P	SCL006_icSARS_72h_2_proteomics	Protein
SCL006	SCL006 2B-4 Cells	Homo sapiens	SCL006_icSARSCoV_72h_3_cell	Cells	SCL006-R	SCL006_icSARSCoV_72h_3_RNA_ExpSam	RNA	SCL006-P	SCL006_icSARS_72h_3_proteomics	Protein
SCL006	SCL006 2B-4 Cells	Homo sapiens	SCL006_icSARSCoV_7h_1_cell	Cells	SCL006-R	SCL006_icSARSCoV_7h_1_RNA_ExpSam	RNA	SCL006-P	SCL006_icSARS_7h_1_proteomics	Protein
SCL006	SCL006 2B-4 Cells	Homo sapiens	SCL006_icSARSCoV_7h_2_cell	Cells	SCL006-R	SCL006_icSARSCoV_7h_2_RNA_ExpSam	RNA	SCL006-P	SCL006_icSARS_7h_2_proteomics	Protein
SCL006	SCL006 2B-4 Cells	Homo sapiens	SCL006_icSARSCoV_7h_3_cell	Cells	SCL006-R	SCL006_icSARSCoV_7h_3_RNA_ExpSam	RNA	SCL006-P	SCL006_icSARS_7h_3_proteomics	Protein
SCL006	SCL006 2B-4 Cells	Homo sapiens	SCL006_mock_0h_1_cell	Cells	SCL006-R	SCL006_mock_0h_1_RNA_ExpSam	RNA	SCL006-P	SCL006_Mock_0h_2_proteomics	Protein
SCL006	SCL006 2B-4 Cells	Homo sapiens	SCL006_mock_0h_2_cell	Cells	SCL006-R	SCL006_mock_0h_2_RNA_ExpSam	RNA	SCL006-P	SCL006_Mock_0h_3_proteomics	Protein
SCL006	SCL006 2B-4 Cells	Homo sapiens	SCL006_mock_0h_3_cell	Cells	SCL006-R	SCL006_mock_0h_3_RNA_ExpSam	RNA	SCL006-P	SCL006_Mock_12h_1_proteomics	Protein
SCL006	SCL006 2B-4 Cells	Homo sapiens	SCL006_mock_12h_1_cell	Cells	SCL006-R	SCL006_mock_12h_1_RNA_ExpSam	RNA	SCL006-P	SCL006_Mock_12h_2_proteomics	Protein
SCL006	SCL006 2B-4 Cells	Homo sapiens	SCL006_mock_12h_2_cell	Cells	SCL006-R	SCL006_mock_12h_2_RNA_ExpSam	RNA	SCL006-P	SCL006_Mock_12h_3_proteomics	Protein
SCL006	SCL006 2B-4 Cells	Homo sapiens	SCL006_mock_12h_3_cell	Cells	SCL006-R	SCL006_mock_12h_3_RNA_ExpSam	RNA	SCL006-P	SCL006_Mock_24h_1_proteomics	Protein
SCL006	SCL006 2B-4 Cells	Homo sapiens	SCL006_mock_24h_1_cell	Cells	SCL006-R	SCL006_mock_24h_1_RNA_ExpSam	RNA	SCL006-P	SCL006_Mock_24h_2_proteomics	Protein
SCL006	SCL006 2B-4 Cells	Homo sapiens	SCL006_mock_24h_2_cell	Cells	SCL006-R	SCL006_mock_24h_2_RNA_ExpSam	RNA	SCL006-P	SCL006_Mock_24h_3_proteomics	Protein
SCL006	SCL006 2B-4 Cells	Homo sapiens	SCL006_mock_24h_3_cell	Cells	SCL006-R	SCL006_mock_24h_3_RNA_ExpSam	RNA	SCL006-P	SCL006_Mock_30h_1_proteomics	Protein
SCL006	SCL006 2B-4 Cells	Homo sapiens	SCL006_mock_30h_1_cell	Cells	SCL006-R	SCL006_mock_30h_1_RNA_ExpSam	RNA	SCL006-P	SCL006_Mock_30h_2_proteomics	Protein
SCL006	SCL006 2B-4 Cells	Homo sapiens	SCL006_mock_30h_2_cell	Cells	SCL006-R	SCL006_mock_30h_2_RNA_ExpSam	RNA	SCL006-P	SCL006_Mock_30h_3_proteomics	Protein
SCL006	SCL006 2B-4 Cells	Homo sapiens	SCL006_mock_30h_3_cell	Cells	SCL006-R	SCL006_mock_30h_3_RNA_ExpSam	RNA	SCL006-P	SCL006_Mock_36h_1_proteomics	Protein
SCL006	SCL006 2B-4 Cells	Homo sapiens	SCL006_mock_36h_1_cell	Cells	SCL006-R	SCL006_mock_36h_1_RNA_ExpSam	RNA	SCL006-P	SCL006_Mock_36h_2_proteomics	Protein
SCL006	SCL006 2B-4 Cells	Homo sapiens	SCL006_mock_36h_2_cell	Cells	SCL006-R	SCL006_mock_36h_2_RNA_ExpSam	RNA	SCL006-P	SCL006_Mock_36h_3_proteomics	Protein
SCL006	SCL006 2B-4 Cells	Homo sapiens	SCL006_mock_36h_3_cell	Cells	SCL006-R	SCL006_mock_36h_3_RNA_ExpSam	RNA	SCL006-P	SCL006_Mock_3h_1_proteomics	Protein
SCL006	SCL006 2B-4 Cells	Homo sapiens	SCL006_mock_48h_1_cell	Cells	SCL006-R	SCL006_mock_48h_1_RNA_ExpSam	RNA	SCL006-P	SCL006_Mock_3h_2_proteomics	Protein
SCL006	SCL006 2B-4 Cells	Homo sapiens	SCL006_mock_48h_2_cell	Cells	SCL006-R	SCL006_mock_48h_2_RNA_ExpSam	RNA	SCL006-P	SCL006_Mock_3h_3_proteomics	Protein
SCL006	SCL006 2B-4 Cells	Homo sapiens	SCL006_mock_48h_3_cell	Cells	SCL006-R	SCL006_mock_48h_3_RNA_ExpSam	RNA	SCL006-P	SCL006_Mock_48h_1_proteomics	Protein
SCL006	SCL006 2B-4 Cells	Homo sapiens	SCL006_mock_54h_1_cell	Cells	SCL006-R	SCL006_mock_54h_1_RNA_ExpSam	RNA	SCL006-P	SCL006_Mock_48h_2_proteomics	Protein
SCL006	SCL006 2B-4 Cells	Homo sapiens	SCL006_mock_54h_2_cell	Cells	SCL006-R	SCL006_mock_54h_2_RNA_ExpSam	RNA	SCL006-P	SCL006_Mock_48h_3_proteomics	Protein
SCL006	SCL006 2B-4 Cells	Homo sapiens	SCL006_mock_54h_3_cell	Cells	SCL006-R	SCL006_mock_54h_3_RNA_ExpSam	RNA	SCL006-P	SCL006_Mock_54h_1_proteomics	Protein
SCL006	SCL006 2B-4 Cells	Homo sapiens	SCL006_mock_60h_1_cell	Cells	SCL006-R	SCL006_mock_60h_1_RNA_ExpSam	RNA	SCL006-P	SCL006_Mock_54h_2_proteomics	Protein
SCL006	SCL006 2B-4 Cells	Homo sapiens	SCL006_mock_60h_2_cell	Cells	SCL006-R	SCL006_mock_60h_2_RNA_ExpSam	RNA	SCL006-P	SCL006_Mock_54h_3_proteomics	Protein
SCL006	SCL006 2B-4 Cells	Homo sapiens	SCL006_mock_60h_3_cell	Cells	SCL006-R	SCL006_mock_60h_3_RNA_ExpSam	RNA	SCL006-P	SCL006_Mock_60h_1_proteomics	Protein
SCL006	SCL006 2B-4 Cells	Homo sapiens	SCL006_mock_72h_1_cell	Cells	SCL006-R	SCL006_mock_72h_1_RNA_ExpSam	RNA	SCL006-P	SCL006_Mock_60h_2_proteomics	Protein
SCL006	SCL006 2B-4 Cells	Homo sapiens	SCL006_mock_72h_2_cell	Cells	SCL006-R	SCL006_mock_72h_2_RNA_ExpSam	RNA	SCL006-P	SCL006_Mock_72h_1_proteomics	Protein
SCL006	SCL006 2B-4 Cells	Homo sapiens	SCL006_mock_72h_3_cell	Cells	SCL006-R	SCL006_mock_72h_3_RNA_ExpSam	RNA	SCL006-P	SCL006_Mock_72h_3_proteomics	Protein
SCL006	SCL006 2B-4 Cells	Homo sapiens	SCL006_mock_7h_1_cell	Cells	SCL006-R	SCL006_mock_7h_1_RNA_ExpSam	RNA	SCL006-P	SCL006_Mock_7h_1_proteomics	Protein
SCL006	SCL006 2B-4 Cells	Homo sapiens	SCL006_mock_7h_2_cell	Cells	SHAE002-R	SCL006_mock_7h_2_RNA_ExpSam	RNA	SCL006-P	SCL006_Mock_7h_2_proteomics	Protein
SCL006	SCL006 2B-4 Cells	Homo sapiens	SCL006_mock_7h_3_cell	Cells	SHAE002-R	SCL006_mock_7h_3_RNA_ExpSam	RNA	SCL006-P	SCL006_Mock_7h_3_proteomics	Protein
SHAE005	SHAE002 HAE cells	Homo sapiens	SHAE002_SARS_0h_1_Cells	Cells	SHAE002-R	SHAE002_SARS_0h_1_array	RNA			
SHAE005	SHAE002 HAE cells	Homo sapiens	SHAE002_SARS_0h_2_Cells	Cells	SHAE002-R	SHAE002_SARS_0h_2_array	RNA			
SHAE005	SHAE002 HAE cells	Homo sapiens	SHAE002_SARS_0h_3_Cells	Cells	SHAE002-R	SHAE002_SARS_0h_3_array	RNA			
SHAE005	SHAE002 HAE cells	Homo sapiens	SHAE002_SARS_0h_4_Cells	Cells	SHAE002-R	SHAE002_SARS_0h_4_array	RNA			
SHAE005	SHAE002 HAE cells	Homo sapiens	SHAE002_SARS_12h_1_Cells	Cells	SHAE002-R	SHAE002_SARS_12h_1_array	RNA			
SHAE005	SHAE002 HAE cells	Homo sapiens	SHAE002_SARS_12h_2_Cells	Cells	SHAE002-R	SHAE002_SARS_12h_2_array	RNA			
SHAE005	SHAE002 HAE cells	Homo sapiens	SHAE002_SARS_12h_3_Cells	Cells	SHAE002-R	SHAE002_SARS_12h_3_array	RNA			
SHAE005	SHAE002 HAE cells	Homo sapiens	SHAE002_SARS_12h_4_Cells	Cells	SHAE002-R	SHAE002_SARS_12h_4_array	RNA			
SHAE005	SHAE002 HAE cells	Homo sapiens	SHAE002_SARS_24h_1_Cells	Cells	SHAE002-R	SHAE002_SARS_24h_1_array	RNA			
SHAE005	SHAE002 HAE cells	Homo sapiens	SHAE002_SARS_24h_2_Cells	Cells	SHAE002-R	SHAE002_SARS_24h_2_array	RNA			
SHAE005	SHAE002 HAE cells	Homo sapiens	SHAE002_SARS_24h_3_Cells	Cells	SHAE002-R	SHAE002_SARS_24h_3_array	RNA			
SHAE005	SHAE002 HAE cells	Homo sapiens	SHAE002_SARS_24h_4_Cells	Cells	SHAE002-R	SHAE002_SARS_24h_4_array	RNA			
SHAE005	SHAE002 HAE cells	Homo sapiens	SHAE002_SARS_36h_1_Cells	Cells	SHAE002-R	SHAE002_SARS_36h_1_array	RNA			
SHAE005	SHAE002 HAE cells	Homo sapiens	SHAE002_SARS_36h_2_Cells	Cells	SHAE002-R	SHAE002_SARS_36h_2_array	RNA			
SHAE005	SHAE002 HAE cells	Homo sapiens	SHAE002_SARS_36h_3_Cells	Cells	SHAE002-R		RNA			
SHAE005	SHAE002 HAE cells	Homo sapiens	SHAE002_SARS_36h_4_Cells	Cells	SHAE002-R		RNA			
SHAE005	SHAE002 HAE cells	Homo sapiens	SHAE002_SARS_48h_1_Cells	Cells	SHAE002-R	SHAE002_SARS_48h_1_array	RNA			
SHAE005	SHAE002 HAE cells	Homo sapiens	SHAE002_SARS_48h_2_Cells	Cells	SHAE002-R	SHAE002_SARS_48h_2_array	RNA			
SHAE005	SHAE002 HAE cells	Homo sapiens	SHAE002_SARS_48h_3_Cells	Cells	SHAE002-R	SHAE002_SARS_48h_3_array	RNA			
SHAE005	SHAE002 HAE cells	Homo sapiens	SHAE002_SARS_48h_4_Cells	Cells	SHAE002-R	SHAE002_SARS_48h_4_array	RNA			
SHAE005	SHAE002 HAE cells	Homo sapiens	SHAE002_SARS_60h_1_Cells	Cells	SHAE002-R	SHAE002_SARS_60h_1_array	RNA			
SHAE005	SHAE002 HAE cells	Homo sapiens	SHAE002_SARS_60h_2_Cells	Cells	SHAE002-R	SHAE002_SARS_60h_2_array	RNA			
SHAE005	SHAE002 HAE cells	Homo sapiens	SHAE002_SARS_60h_3_Cells	Cells	SHAE002-R	SHAE002_SARS_60h_3_array	RNA			
SHAE005	SHAE002 HAE cells	Homo sapiens	SHAE002_SARS_60h_4_Cells	Cells	SHAE002-R	SHAE002_SARS_60h_4_array	RNA			
SHAE005	SHAE002 HAE cells	Homo sapiens	SHAE002_SARS_72h_1_Cells	Cells	SHAE002-R	SHAE002_SARS_72h_1_array	RNA			
SHAE005	SHAE002 HAE cells	Homo sapiens	SHAE002_SARS_72h_2_Cells	Cells	SHAE002-R	SHAE002_SARS_72h_2_array	RNA			
SHAE005	SHAE002 HAE cells	Homo sapiens	SHAE002_SARS_72h_3_Cells	Cells	SHAE002-R	SHAE002_SARS_72h_3_array	RNA			
SHAE005	SHAE002 HAE cells	Homo sapiens	SHAE002_SARS_72h_4_Cells	Cells	SHAE002-R	SHAE002_SARS_72h_4_array	RNA			
SHAE005	SHAE002 HAE cells	Homo sapiens	SHAE002_SARS_84h_1_Cells	Cells	SHAE002-R	SHAE002_SARS_84h_1_array	RNA			
SHAE005	SHAE002 HAE cells	Homo sapiens	SHAE002_SARS_84h_2_Cells	Cells	SHAE002-R	SHAE002_SARS_84h_2_array	RNA			
SHAE005	SHAE002 HAE cells	Homo sapiens	SHAE002_SARS_84h_3_Cells	Cells	SHAE002-R	SHAE002_SARS_84h_3_array	RNA			
SHAE005	SHAE002 HAE cells	Homo sapiens	SHAE002_SARS_84h_4_Cells	Cells	SHAE002-R	SHAE002_SARS_84h_4_array	RNA			
SHAE005	SHAE002 HAE cells	Homo sapiens	SHAE002_SARS_96h_1_Cells	Cells	SHAE002-R	SHAE002_SARS_96h_1_array	RNA			
SHAE005	SHAE002 HAE cells	Homo sapiens	SHAE002_SARS_96h_2_Cells	Cells	SHAE002-R	SHAE002_SARS_96h_2_array	RNA			
SHAE005	SHAE002 HAE cells	Homo sapiens	SHAE002_SARS_96h_3_Cells	Cells	SHAE002-R	SHAE002_SARS_96h_3_array	RNA			
SHAE005	SHAE002 HAE cells	Homo sapiens	SHAE002_SARS_96h_4_Cells	Cells	SHAE002-R	SHAE002_SARS_96h_4_array	RNA			
SHAE005	SHAE002 HAE cells	Homo sapiens	SHAE002_BAT_0h_1_Cells	Cells	SHAE002-R	SHAE002_BAT_0h_1_array	RNA			
SHAE005	SHAE002 HAE cells	Homo sapiens	SHAE002_BAT_0h_2_Cells	Cells	SHAE002-R	SHAE002_BAT_0h_2_array	RNA			
SHAE005	SHAE002 HAE cells	Homo sapiens	SHAE002_BAT_0h_3_Cells	Cells	SHAE002-R	SHAE002_BAT_0h_3_array	RNA			
SHAE005	SHAE002 HAE cells	Homo sapiens	SHAE002_BAT_0h_4_Cells	Cells	SHAE002-R	SHAE002_BAT_0h_4_array	RNA			
SHAE005	SHAE002 HAE cells	Homo sapiens	SHAE002_BAT_12h_1_Cells	Cells	SHAE002-R	SHAE002_BAT_12h_1_array	RNA			
SHAE005	SHAE002 HAE cells	Homo sapiens	SHAE002_BAT_12h_2_Cells	Cells	SHAE002-R	SHAE002_BAT_12h_2_array	RNA			
SHAE005	SHAE002 HAE cells	Homo sapiens	SHAE002_BAT_12h_3_Cells	Cells	SHAE002-R	SHAE002_BAT_12h_3_array	RNA			
SHAE005	SHAE002 HAE cells	Homo sapiens	SHAE002_BAT_12h_4_Cells	Cells	SHAE002-R	SHAE002_BAT_12h_4_array	RNA			
SHAE005	SHAE002 HAE cells	Homo sapiens	SHAE002_BAT_24h_1_Cells	Cells	SHAE002-R	SHAE002_BAT_24h_1_array	RNA			
SHAE005	SHAE002 HAE cells	Homo sapiens	SHAE002_BAT_24h_2_Cells	Cells	SHAE002-R	SHAE002_BAT_24h_2_array	RNA			
SHAE005	SHAE002 HAE cells	Homo sapiens	SHAE002_BAT_24h_3_Cells	Cells	SHAE002-R	SHAE002_BAT_24h_3_array	RNA			
SHAE005	SHAE002 HAE cells	Homo sapiens	SHAE002_BAT_24h_4_Cells	Cells	SHAE002-R	SHAE002_BAT_24h_4_array	RNA			
SHAE005	SHAE002 HAE cells	Homo sapiens	SHAE002_BAT_36h_1_Cells	Cells	SHAE002-R	SHAE002_BAT_36h_1_array	RNA			
SHAE005	SHAE002 HAE cells	Homo sapiens	SHAE002_BAT_36h_2_Cells	Cells	SHAE002-R	SHAE002_BAT_36h_2_array	RNA			
SHAE005	SHAE002 HAE cells	Homo sapiens	SHAE002_BAT_36h_3_Cells	Cells	SHAE002-R	SHAE002_BAT_36h_3_array	RNA			
SHAE005	SHAE002 HAE cells	Homo sapiens	SHAE002_BAT_36h_4_Cells	Cells	SHAE002-R	SHAE002_BAT_36h_4_array	RNA			
SHAE005	SHAE002 HAE cells	Homo sapiens	SHAE002_BAT_48h_1_Cells	Cells	SHAE002-R	SHAE002_BAT_48h_1_array	RNA			
SHAE005	SHAE002 HAE cells	Homo sapiens	SHAE002_BAT_48h_2_Cells	Cells	SHAE002-R		RNA			
SHAE005	SHAE002 HAE cells	Homo sapiens	SHAE002_BAT_48h_3_Cells	Cells	SHAE002-R	SHAE002_BAT_48h_3_array	RNA			
SHAE005	SHAE002 HAE cells	Homo sapiens	SHAE002_BAT_48h_4_Cells	Cells	SHAE002-R	SHAE002_BAT_48h_4_array	RNA			
SHAE005	SHAE002 HAE cells	Homo sapiens	SHAE002_BAT_60h_1_Cells	Cells	SHAE002-R	SHAE002_BAT_60h_1_array	RNA			
SHAE005	SHAE002 HAE cells	Homo sapiens	SHAE002_BAT_60h_2_Cells	Cells	SHAE002-R	SHAE002_BAT_60h_2_array	RNA			
SHAE005	SHAE002 HAE cells	Homo sapiens	SHAE002_BAT_60h_3_Cells	Cells	SHAE002-R	SHAE002_BAT_60h_3_array	RNA			
SHAE005	SHAE002 HAE cells	Homo sapiens	SHAE002_BAT_60h_4_Cells	Cells	SHAE002-R	SHAE002_BAT_60h_4_array	RNA			
SHAE005	SHAE002 HAE cells	Homo sapiens	SHAE002_BAT_72h_1_Cells	Cells	SHAE002-R	SHAE002_BAT_72h_1_array	RNA			
SHAE005	SHAE002 HAE cells	Homo sapiens	SHAE002_BAT_72h_2_Cells	Cells	SHAE002-R	SHAE002_BAT_72h_2_array	RNA			
SHAE005	SHAE002 HAE cells	Homo sapiens	SHAE002_BAT_72h_3_Cells	Cells	SHAE002-R	SHAE002_BAT_72h_3_array	RNA			
SHAE005	SHAE002 HAE cells	Homo sapiens	SHAE002_BAT_72h_4_Cells	Cells	SHAE002-R	SHAE002_BAT_72h_4_array	RNA			
SHAE005	SHAE002 HAE cells	Homo sapiens	SHAE002_BAT_84h_1_Cells	Cells	SHAE002-R	SHAE002_BAT_84h_1_array	RNA			
SHAE005	SHAE002 HAE cells	Homo sapiens	SHAE002_BAT_84h_2_Cells	Cells	SHAE002-R	SHAE002_BAT_84h_2_array	RNA			
SHAE005	SHAE002 HAE cells	Homo sapiens	SHAE002_BAT_84h_3_Cells	Cells	SHAE002-R	SHAE002_BAT_84h_3_array	RNA			
SHAE005	SHAE002 HAE cells	Homo sapiens	SHAE002_BAT_84h_4_Cells	Cells	SHAE002-R	SHAE002_BAT_84h_4_array	RNA			
SHAE005	SHAE002 HAE cells	Homo sapiens	SHAE002_BAT_96h_1_Cells	Cells	SHAE002-R	SHAE002_BAT_96h_1_array	RNA			
SHAE005	SHAE002 HAE cells	Homo sapiens	SHAE002_BAT_96h_2_Cells	Cells	SHAE002-R	SHAE002_BAT_96h_2_array	RNA			
SHAE005	SHAE002 HAE cells	Homo sapiens	SHAE002_BAT_96h_3_Cells	Cells	SHAE002-R	SHAE002_BAT_96h_3_array	RNA			
SHAE005	SHAE002 HAE cells	Homo sapiens	SHAE002_BAT_96h_4_Cells	Cells	SHAE002-R	SHAE002_BAT_96h_4_array	RNA			
SHAE005	SHAE002 HAE cells	Homo sapiens	SHAE002_dORF6_0h_1_Cells	Cells	SHAE002-R	SHAE002_dORF6_0h_1_array	RNA			
SHAE005	SHAE002 HAE cells	Homo sapiens	SHAE002_dORF6_0h_2_Cells	Cells	SHAE002-R	SHAE002_dORF6_0h_2_array	RNA			
SHAE005	SHAE002 HAE cells	Homo sapiens	SHAE002_dORF6_0h_3_Cells	Cells	SHAE002-R	SHAE002_dORF6_0h_3_array	RNA			
SHAE005	SHAE002 HAE cells	Homo sapiens	SHAE002_dORF6_0h_4_Cells	Cells	SHAE002-R	SHAE002_dORF6_0h_4_array	RNA			
SHAE005	SHAE002 HAE cells	Homo sapiens	SHAE002_dORF6_12h_1_Cells	Cells	SHAE002-R	SHAE002_dORF6_12h_1_array	RNA			
SHAE005	SHAE002 HAE cells	Homo sapiens	SHAE002_dORF6_12h_2_Cells	Cells	SHAE002-R	SHAE002_dORF6_12h_2_array	RNA			
SHAE005	SHAE002 HAE cells	Homo sapiens	SHAE002_dORF6_12h_3_Cells	Cells	SHAE002-R	SHAE002_dORF6_12h_3_array	RNA			
SHAE005	SHAE002 HAE cells	Homo sapiens	SHAE002_dORF6_12h_4_Cells	Cells	SHAE002-R	SHAE002_dORF6_12h_4_array	RNA			
SHAE005	SHAE002 HAE cells	Homo sapiens	SHAE002_dORF6_24h_1_Cells	Cells	SHAE002-R	SHAE002_dORF6_24h_1_array	RNA			
SHAE005	SHAE002 HAE cells	Homo sapiens	SHAE002_dORF6_24h_2_Cells	Cells	SHAE002-R	SHAE002_dORF6_24h_2_array	RNA			
SHAE005	SHAE002 HAE cells	Homo sapiens	SHAE002_dORF6_24h_3_Cells	Cells	SHAE002-R		RNA			
SHAE005	SHAE002 HAE cells	Homo sapiens	SHAE002_dORF6_24h_4_Cells	Cells	SHAE002-R	SHAE002_dORF6_24h_4_array	RNA			
SHAE005	SHAE002 HAE cells	Homo sapiens	SHAE002_dORF6_36h_1_Cells	Cells	SHAE002-R	SHAE002_dORF6_36h_1_array	RNA			
SHAE005	SHAE002 HAE cells	Homo sapiens	SHAE002_dORF6_36h_2_Cells	Cells	SHAE002-R	SHAE002_dORF6_36h_2_array	RNA			
SHAE005	SHAE002 HAE cells	Homo sapiens	SHAE002_dORF6_36h_3_Cells	Cells	SHAE002-R	SHAE002_dORF6_36h_3_array	RNA			
SHAE005	SHAE002 HAE cells	Homo sapiens	SHAE002_dORF6_36h_4_Cells	Cells	SHAE002-R	SHAE002_dORF6_36h_4_array	RNA			
SHAE005	SHAE002 HAE cells	Homo sapiens	SHAE002_dORF6_48h_1_Cells	Cells	SHAE002-R	SHAE002_dORF6_48h_1_array	RNA			
SHAE005	SHAE002 HAE cells	Homo sapiens	SHAE002_dORF6_48h_2_Cells	Cells	SHAE002-R	SHAE002_dORF6_48h_2_array	RNA			
SHAE005	SHAE002 HAE cells	Homo sapiens	SHAE002_dORF6_48h_3_Cells	Cells	SHAE002-R	SHAE002_dORF6_48h_3_array	RNA			
SHAE005	SHAE002 HAE cells	Homo sapiens	SHAE002_dORF6_48h_4_Cells	Cells	SHAE002-R	SHAE002_dORF6_48h_4_array	RNA			
SHAE005	SHAE002 HAE cells	Homo sapiens	SHAE002_dORF6_60h_1_Cells	Cells	SHAE002-R	SHAE002_dORF6_60h_1_array	RNA			
SHAE005	SHAE002 HAE cells	Homo sapiens	SHAE002_dORF6_60h_2_Cells	Cells	SHAE002-R	SHAE002_dORF6_60h_2_array	RNA			
SHAE005	SHAE002 HAE cells	Homo sapiens	SHAE002_dORF6_60h_3_Cells	Cells	SHAE002-R	SHAE002_dORF6_60h_3_array	RNA			
SHAE005	SHAE002 HAE cells	Homo sapiens	SHAE002_dORF6_60h_4_Cells	Cells	SHAE002-R	SHAE002_dORF6_60h_4_array	RNA			
SHAE005	SHAE002 HAE cells	Homo sapiens	SHAE002_dORF6_72h_1_Cells	Cells	SHAE002-R	SHAE002_dORF6_72h_1_array	RNA			
SHAE005	SHAE002 HAE cells	Homo sapiens	SHAE002_dORF6_72h_2_Cells	Cells	SHAE002-R	SHAE002_dORF6_72h_2_array	RNA			
SHAE005	SHAE002 HAE cells	Homo sapiens	SHAE002_dORF6_72h_3_Cells	Cells	SHAE002-R	SHAE002_dORF6_72h_3_array	RNA			
SHAE005	SHAE002 HAE cells	Homo sapiens	SHAE002_dORF6_72h_4_Cells	Cells	SHAE002-R	SHAE002_dORF6_72h_4_array	RNA			
SHAE005	SHAE002 HAE cells	Homo sapiens	SHAE002_dORF6_84h_1_Cells	Cells	SHAE002-R	SHAE002_dORF6_84h_1_array	RNA			
SHAE005	SHAE002 HAE cells	Homo sapiens	SHAE002_dORF6_84h_2_Cells	Cells	SHAE002-R	SHAE002_dORF6_84h_2_array	RNA			
SHAE005	SHAE002 HAE cells	Homo sapiens	SHAE002_dORF6_84h_3_Cells	Cells	SHAE002-R	SHAE002_dORF6_84h_3_array	RNA			
SHAE005	SHAE002 HAE cells	Homo sapiens	SHAE002_dORF6_84h_4_Cells	Cells	SHAE002-R	SHAE002_dORF6_84h_4_array	RNA			
SHAE005	SHAE002 HAE cells	Homo sapiens	SHAE002_dORF6_96h_1_Cells	Cells	SHAE002-R	SHAE002_dORF6_96h_1_array	RNA			
SHAE005	SHAE002 HAE cells	Homo sapiens	SHAE002_dORF6_96h_2_Cells	Cells	SHAE002-R	SHAE002_dORF6_96h_2_array	RNA			
SHAE005	SHAE002 HAE cells	Homo sapiens	SHAE002_dORF6_96h_3_Cells	Cells	SHAE002-R	SHAE002_dORF6_96h_3_array	RNA			
SHAE005	SHAE002 HAE cells	Homo sapiens	SHAE002_dORF6_96h_4_Cells	Cells	SHAE002-R	SHAE002_dORF6_96h_4_array	RNA			
SHAE005	SHAE002 HAE cells	Homo sapiens	SHAE002_H1N1_0h_1_Cells	Cells	SHAE002-R	SHAE002_H1N1_0h_1_array	RNA			
SHAE005	SHAE002 HAE cells	Homo sapiens	SHAE002_H1N1_0h_2_Cells	Cells	SHAE002-R	SHAE002_H1N1_0h_2_array	RNA			
SHAE005	SHAE002 HAE cells	Homo sapiens	SHAE002_H1N1_0h_3_Cells	Cells	SHAE002-R	SHAE002_H1N1_0h_3_array	RNA			
SHAE005	SHAE002 HAE cells	Homo sapiens	SHAE002_H1N1_6h_1_Cells	Cells	SHAE002-R	SHAE002_H1N1_12h_1_array	RNA			
SHAE005	SHAE002 HAE cells	Homo sapiens	SHAE002_H1N1_6h_2_Cells	Cells	SHAE002-R	SHAE002_H1N1_12h_2_array	RNA			
SHAE005	SHAE002 HAE cells	Homo sapiens	SHAE002_H1N1_6h_3_Cells	Cells	SHAE002-R	SHAE002_H1N1_12h_3_array	RNA			
SHAE005	SHAE002 HAE cells	Homo sapiens	SHAE002_H1N1_12h_1_Cells	Cells	SHAE002-R	SHAE002_H1N1_18h_1_array	RNA			
SHAE005	SHAE002 HAE cells	Homo sapiens	SHAE002_H1N1_12h_2_Cells	Cells	SHAE002-R	SHAE002_H1N1_18h_2_array	RNA			
SHAE005	SHAE002 HAE cells	Homo sapiens	SHAE002_H1N1_12h_3_Cells	Cells	SHAE002-R	SHAE002_H1N1_18h_3_array	RNA			
SHAE005	SHAE002 HAE cells	Homo sapiens	SHAE002_H1N1_18h_1_Cells	Cells	SHAE002-R	SHAE002_H1N1_24h_1_array	RNA			
SHAE005	SHAE002 HAE cells	Homo sapiens	SHAE002_H1N1_18h_2_Cells	Cells	SHAE002-R	SHAE002_H1N1_24h_2_array	RNA			
SHAE005	SHAE002 HAE cells	Homo sapiens	SHAE002_H1N1_18h_3_Cells	Cells	SHAE002-R	SHAE002_H1N1_24h_3_array	RNA			
SHAE005	SHAE002 HAE cells	Homo sapiens	SHAE002_H1N1_24h_1_Cells	Cells	SHAE002-R	SHAE002_H1N1_36h_1_array	RNA			
SHAE005	SHAE002 HAE cells	Homo sapiens	SHAE002_H1N1_24h_2_Cells	Cells	SHAE002-R	SHAE002_H1N1_36h_2_array	RNA			
SHAE005	SHAE002 HAE cells	Homo sapiens	SHAE002_H1N1_24h_3_Cells	Cells	SHAE002-R	SHAE002_H1N1_36h_3_array	RNA			
SHAE005	SHAE002 HAE cells	Homo sapiens	SHAE002_H1N1_36h_1_Cells	Cells	SHAE002-R	SHAE002_H1N1_48h_1_array	RNA			
SHAE005	SHAE002 HAE cells	Homo sapiens	SHAE002_H1N1_36h_2_Cells	Cells	SHAE002-R	SHAE002_H1N1_48h_2_array	RNA			
SHAE005	SHAE002 HAE cells	Homo sapiens	SHAE002_H1N1_36h_3_Cells	Cells	SHAE002-R	SHAE002_H1N1_48h_3_array	RNA			
SHAE005	SHAE002 HAE cells	Homo sapiens	SHAE002_H1N1_48h_1_Cells	Cells	SHAE002-R	SHAE002_H1N1_48h_4_array	RNA			
SHAE005	SHAE002 HAE cells	Homo sapiens	SHAE002_H1N1_48h_2_Cells	Cells	SHAE002-R	SHAE002_H1N1_6h_1_array	RNA			
SHAE005	SHAE002 HAE cells	Homo sapiens	SHAE002_H1N1_48h_3_Cells	Cells	SHAE002-R	SHAE002_H1N1_6h_2_array	RNA			
SHAE005	SHAE002 HAE cells	Homo sapiens	SHAE002_H1N1_48h_4_Cells	Cells	SHAE002-R	SHAE002_H1N1_6h_3_array	RNA			
SHAE005	SHAE002 HAE cells	Homo sapiens	SHAE002_Mock_0h_1_Cells	Cells	SHAE002-R	SHAE002_Mock_0h_1_array	RNA			
SHAE005	SHAE002 HAE cells	Homo sapiens	SHAE002_Mock_0h_2_Cells	Cells	SHAE002-R	SHAE002_Mock_0h_2_array	RNA			
SHAE005	SHAE002 HAE cells	Homo sapiens	SHAE002_Mock_0h_3_Cells	Cells	SHAE002-R	SHAE002_Mock_0h_3_array	RNA			
SHAE005	SHAE002 HAE cells	Homo sapiens	SHAE002_Mock_6h_1_Cells	Cells	SHAE002-R	SHAE002_Mock_12h_1_array	RNA			
SHAE005	SHAE002 HAE cells	Homo sapiens	SHAE002_Mock_6h_2_Cells	Cells	SHAE002-R	SHAE002_Mock_12h_2_array	RNA			
SHAE005	SHAE002 HAE cells	Homo sapiens	SHAE002_Mock_6h_3_Cells	Cells	SHAE002-R	SHAE002_Mock_12h_3_array	RNA			
SHAE005	SHAE002 HAE cells	Homo sapiens	SHAE002_Mock_12h_1_Cells	Cells	SHAE002-R	SHAE002_Mock_18h_1_array	RNA			
SHAE005	SHAE002 HAE cells	Homo sapiens	SHAE002_Mock_12h_2_Cells	Cells	SHAE002-R	SHAE002_Mock_18h_2_array	RNA			
SHAE005	SHAE002 HAE cells	Homo sapiens	SHAE002_Mock_12h_3_Cells	Cells	SHAE002-R	SHAE002_Mock_18h_3_array	RNA			
SHAE005	SHAE002 HAE cells	Homo sapiens	SHAE002_Mock_18h_1_Cells	Cells	SHAE002-R	SHAE002_Mock_24h_1_array	RNA			
SHAE005	SHAE002 HAE cells	Homo sapiens	SHAE002_Mock_18h_2_Cells	Cells	SHAE002-R	SHAE002_Mock_24h_2_array	RNA			
SHAE005	SHAE002 HAE cells	Homo sapiens	SHAE002_Mock_18h_3_Cells	Cells	SHAE002-R	SHAE002_Mock_24h_3_array	RNA			
SHAE005	SHAE002 HAE cells	Homo sapiens	SHAE002_Mock_24h_1_Cells	Cells	SHAE002-R	SHAE002_Mock_36h_1_array	RNA			
SHAE005	SHAE002 HAE cells	Homo sapiens	SHAE002_Mock_24h_2_Cells	Cells	SHAE002-R	SHAE002_Mock_36h_2_array	RNA			
SHAE005	SHAE002 HAE cells	Homo sapiens	SHAE002_Mock_24h_3_Cells	Cells	SHAE002-R	SHAE002_Mock_36h_3_array	RNA			
SHAE005	SHAE002 HAE cells	Homo sapiens	SHAE002_Mock_36h_1_Cells	Cells	SHAE002-R	SHAE002_Mock_48h_1_array	RNA			
SHAE005	SHAE002 HAE cells	Homo sapiens	SHAE002_Mock_36h_2_Cells	Cells	SHAE002-R	SHAE002_Mock_48h_3_array	RNA			
SHAE005	SHAE002 HAE cells	Homo sapiens	SHAE002_Mock_36h_3_Cells	Cells	SHAE002-R	SHAE002_Mock_48h_4_array	RNA			
SHAE005	SHAE002 HAE cells	Homo sapiens	SHAE002_Mock_48h_1_Cells	Cells	SHAE002-R	SHAE002_Mock_60h_1_array	RNA			
SHAE005	SHAE002 HAE cells	Homo sapiens	SHAE002_Mock_48h_3_Cells	Cells	SHAE002-R	SHAE002_Mock_60h_2_array	RNA			
SHAE005	SHAE002 HAE cells	Homo sapiens	SHAE002_Mock_48h_4_Cells	Cells	SHAE002-R	SHAE002_Mock_60h_3_array	RNA			
SHAE005	SHAE002 HAE cells	Homo sapiens	SHAE002_Mock_60h_1_Cells	Cells	SHAE002-R	SHAE002_Mock_60h_4_array	RNA			
SHAE005	SHAE002 HAE cells	Homo sapiens	SHAE002_Mock_60h_2_Cells	Cells	SHAE002-R	SHAE002_Mock_6h_1_array	RNA			
SHAE005	SHAE002 HAE cells	Homo sapiens	SHAE002_Mock_60h_3_Cells	Cells	SHAE002-R	SHAE002_Mock_6h_2_array	RNA			
SHAE005	SHAE002 HAE cells	Homo sapiens	SHAE002_Mock_60h_4_Cells	Cells	SHAE002-R	SHAE002_Mock_6h_3_array	RNA			
SHAE005	SHAE002 HAE cells	Homo sapiens	SHAE002_Mock_72h_1_Cells	Cells	SHAE002-R	SHAE002_Mock_72h_1_array	RNA			
SHAE005	SHAE002 HAE cells	Homo sapiens	SHAE002_Mock_72h_2_Cells	Cells	SHAE002-R	SHAE002_Mock_72h_2_array	RNA			
SHAE005	SHAE002 HAE cells	Homo sapiens	SHAE002_Mock_72h_3_Cells	Cells	SHAE002-R	SHAE002_Mock_72h_3_array	RNA			
SHAE005	SHAE002 HAE cells	Homo sapiens	SHAE002_Mock_72h_4_Cells	Cells	SHAE002-R	SHAE002_Mock_72h_4_array	RNA			
SHAE005	SHAE002 HAE cells	Homo sapiens	SHAE002_Mock_84h_1_Cells	Cells	SHAE002-R	SHAE002_Mock_84h_1_array	RNA			
SHAE005	SHAE002 HAE cells	Homo sapiens	SHAE002_Mock_84h_2_Cells	Cells	SHAE002-R	SHAE002_Mock_84h_2_array	RNA			
SHAE005	SHAE002 HAE cells	Homo sapiens	SHAE002_Mock_84h_3_Cells	Cells	SHAE003-R	SHAE002_Mock_84h_3_array	RNA			
SHAE005	SHAE002 HAE cells	Homo sapiens	SHAE002_Mock_84h_4_Cells	Cells	SHAE003-R	SHAE002_Mock_84h_4_array	RNA			
SHAE005	SHAE002 HAE cells	Homo sapiens	SHAE002_Mock_96h_1_Cells	Cells	SHAE003-R	SHAE002_Mock_96h_1_array	RNA			
SHAE005	SHAE002 HAE cells	Homo sapiens	SHAE002_Mock_96h_2_Cells	Cells	SHAE003-R	SHAE002_Mock_96h_2_array	RNA			
SHAE005	SHAE002 HAE cells	Homo sapiens	SHAE002_Mock_96h_3_Cells	Cells	SHAE003-R	SHAE002_Mock_96h_3_array	RNA			
SHAE005	SHAE002 HAE cells	Homo sapiens	SHAE002_Mock_96h_4_Cells	Cells	SHAE003-R	SHAE002_Mock_96h_4_array	RNA			
SHAE005	SHAE003 HAE cells	Homo sapiens	SHAE003_SARS_0h_1_Cells	Cells	SHAE003-R	SHAE003_SARS_0h_1_array	RNA			
SHAE005	SHAE003 HAE cells	Homo sapiens	SHAE003_SARS_0h_2_Cells	Cells	SHAE003-R	SHAE003_SARS_0h_2_array	RNA			
SHAE005	SHAE003 HAE cells	Homo sapiens	SHAE003_SARS_0h_3_Cells	Cells	SHAE003-R	SHAE003_SARS_0h_3_array	RNA			
SHAE005	SHAE003 HAE cells	Homo sapiens	SHAE003_SARS_0h_4_Cells	Cells	SHAE003-R	SHAE003_SARS_0h_4_array	RNA			
SHAE005	SHAE003 HAE cells	Homo sapiens	SHAE003_SARS_24h_1_Cells	Cells	SHAE003-R	SHAE003_SARS_24h_1_array	RNA			
SHAE005	SHAE003 HAE cells	Homo sapiens	SHAE003_SARS_24h_2_Cells	Cells	SHAE003-R	SHAE003_SARS_24h_2_array	RNA			
SHAE005	SHAE003 HAE cells	Homo sapiens	SHAE003_SARS_24h_3_Cells	Cells	SHAE003-R	SHAE003_SARS_24h_3_array	RNA			
SHAE005	SHAE003 HAE cells	Homo sapiens	SHAE003_SARS_24h_4_Cells	Cells	SHAE003-R	SHAE003_SARS_24h_4_array	RNA			
SHAE005	SHAE003 HAE cells	Homo sapiens	SHAE003_SARS_48h_1_Cells	Cells	SHAE003-R	SHAE003_SARS_48h_1_array	RNA			
SHAE005	SHAE003 HAE cells	Homo sapiens	SHAE003_SARS_48h_2_Cells	Cells	SHAE003-R	SHAE003_SARS_48h_2_array	RNA			
SHAE005	SHAE003 HAE cells	Homo sapiens	SHAE003_SARS_48h_3_Cells	Cells	SHAE003-R	SHAE003_SARS_48h_3_array	RNA			
SHAE005	SHAE003 HAE cells	Homo sapiens	SHAE003_SARS_48h_4_Cells	Cells	SHAE003-R		RNA			
SHAE005	SHAE003 HAE cells	Homo sapiens	SHAE003_SARS_60h_1_Cells	Cells	SHAE003-R	SHAE003_SARS_60h_1_array	RNA			
SHAE005	SHAE003 HAE cells	Homo sapiens	SHAE003_SARS_60h_2_Cells	Cells	SHAE003-R	SHAE003_SARS_60h_2_array	RNA			
SHAE005	SHAE003 HAE cells	Homo sapiens	SHAE003_SARS_60h_3_Cells	Cells	SHAE003-R	SHAE003_SARS_60h_3_array	RNA			
SHAE005	SHAE003 HAE cells	Homo sapiens	SHAE003_SARS_60h_4_Cells	Cells	SHAE003-R	SHAE003_SARS_60h_4_array	RNA			
SHAE005	SHAE003 HAE cells	Homo sapiens	SHAE003_SARS_72h_1_Cells	Cells	SHAE003-R	SHAE003_SARS_72h_1_array	RNA			
SHAE005	SHAE003 HAE cells	Homo sapiens	SHAE003_SARS_72h_2_Cells	Cells	SHAE003-R	SHAE003_SARS_72h_2_array	RNA			
SHAE005	SHAE003 HAE cells	Homo sapiens	SHAE003_SARS_72h_3_Cells	Cells	SHAE003-R	SHAE003_SARS_72h_3_array	RNA			
SHAE005	SHAE003 HAE cells	Homo sapiens	SHAE003_SARS_72h_4_Cells	Cells	SHAE003-R	SHAE003_SARS_72h_4_array	RNA			
SHAE005	SHAE003 HAE cells	Homo sapiens	SHAE003_SARS_84h_1_Cells	Cells	SHAE003-R	SHAE003_SARS_84h_1_array	RNA			
SHAE005	SHAE003 HAE cells	Homo sapiens	SHAE003_SARS_84h_2_Cells	Cells	SHAE003-R	SHAE003_SARS_84h_2_array	RNA			
SHAE005	SHAE003 HAE cells	Homo sapiens	SHAE003_SARS_84h_3_Cells	Cells	SHAE003-R	SHAE003_SARS_84h_3_array	RNA			
SHAE005	SHAE003 HAE cells	Homo sapiens	SHAE003_SARS_84h_4_Cells	Cells	SHAE003-R	SHAE003_SARS_84h_4_array	RNA			
SHAE005	SHAE003 HAE cells	Homo sapiens	SHAE003_SARS_96h_1_Cells	Cells	SHAE003-R	SHAE003_SARS_96h_1_array	RNA			
SHAE005	SHAE003 HAE cells	Homo sapiens	SHAE003_SARS_96h_2_Cells	Cells	SHAE003-R	SHAE003_SARS_96h_2_array	RNA			
SHAE005	SHAE003 HAE cells	Homo sapiens	SHAE003_SARS_96h_3_Cells	Cells	SHAE003-R	SHAE003_SARS_96h_3_array	RNA			
SHAE005	SHAE003 HAE cells	Homo sapiens	SHAE003_SARS_96h_4_Cells	Cells	SHAE003-R	SHAE003_SARS_96h_4_array	RNA			
SHAE005	SHAE003 HAE cells	Homo sapiens	SHAE003_BAT_0h_1_Cells	Cells	SHAE003-R	SHAE003_BAT_0h_1_array	RNA			
SHAE005	SHAE003 HAE cells	Homo sapiens	SHAE003_BAT_0h_2_Cells	Cells	SHAE003-R	SHAE003_BAT_0h_2_array	RNA			
SHAE005	SHAE003 HAE cells	Homo sapiens	SHAE003_BAT_0h_3_Cells	Cells	SHAE003-R	SHAE003_BAT_0h_3_array	RNA			
SHAE005	SHAE003 HAE cells	Homo sapiens	SHAE003_BAT_0h_4_Cells	Cells	SHAE003-R	SHAE003_BAT_0h_4_array	RNA			
SHAE005	SHAE003 HAE cells	Homo sapiens	SHAE003_BAT_24h_3_Cells	Cells	SHAE003-R	SHAE003_BAT_24h_3_array	RNA			
SHAE005	SHAE003 HAE cells	Homo sapiens	SHAE003_BAT_24h_4_Cells	Cells	SHAE003-R	SHAE003_BAT_24h_4_array	RNA			
SHAE005	SHAE003 HAE cells	Homo sapiens	SHAE003_BAT_48h_1_Cells	Cells	SHAE003-R	SHAE003_BAT_48h_1_array	RNA			
SHAE005	SHAE003 HAE cells	Homo sapiens	SHAE003_BAT_48h_2_Cells	Cells	SHAE003-R	SHAE003_BAT_48h_2_array	RNA			
SHAE005	SHAE003 HAE cells	Homo sapiens	SHAE003_BAT_48h_3_Cells	Cells	SHAE003-R	SHAE003_BAT_48h_3_array	RNA			
SHAE005	SHAE003 HAE cells	Homo sapiens	SHAE003_BAT_48h_4_Cells	Cells	SHAE003-R	SHAE003_BAT_48h_4_array	RNA			
SHAE005	SHAE003 HAE cells	Homo sapiens	SHAE003_BAT_60h_1_Cells	Cells	SHAE003-R	SHAE003_BAT_60h_1_array	RNA			
SHAE005	SHAE003 HAE cells	Homo sapiens	SHAE003_BAT_60h_2_Cells	Cells	SHAE003-R	SHAE003_BAT_60h_2_array	RNA			
SHAE005	SHAE003 HAE cells	Homo sapiens	SHAE003_BAT_60h_3_Cells	Cells	SHAE003-R	SHAE003_BAT_60h_3_array	RNA			
SHAE005	SHAE003 HAE cells	Homo sapiens	SHAE003_BAT_60h_4_Cells	Cells	SHAE003-R	SHAE003_BAT_60h_4_array	RNA			
SHAE005	SHAE003 HAE cells	Homo sapiens	SHAE003_BAT_72h_1_Cells	Cells	SHAE003-R	SHAE003_BAT_72h_1_array	RNA			
SHAE005	SHAE003 HAE cells	Homo sapiens	SHAE003_BAT_72h_2_Cells	Cells	SHAE003-R	SHAE003_BAT_72h_2_array	RNA			
SHAE005	SHAE003 HAE cells	Homo sapiens	SHAE003_BAT_72h_3_Cells	Cells	SHAE003-R	SHAE003_BAT_72h_3_array	RNA			
SHAE005	SHAE003 HAE cells	Homo sapiens	SHAE003_BAT_72h_4_Cells	Cells	SHAE003-R	SHAE003_BAT_72h_4_array	RNA			
SHAE005	SHAE003 HAE cells	Homo sapiens	SHAE003_BAT_84h_1_Cells	Cells	SHAE003-R	SHAE003_BAT_84h_1_array	RNA			
SHAE005	SHAE003 HAE cells	Homo sapiens	SHAE003_BAT_84h_2_Cells	Cells	SHAE003-R	SHAE003_BAT_84h_2_array	RNA			
SHAE005	SHAE003 HAE cells	Homo sapiens	SHAE003_BAT_84h_3_Cells	Cells	SHAE003-R	SHAE003_BAT_84h_3_array	RNA			
SHAE005	SHAE003 HAE cells	Homo sapiens	SHAE003_BAT_84h_4_Cells	Cells	SHAE003-R	SHAE003_BAT_84h_4_array	RNA			
SHAE005	SHAE003 HAE cells	Homo sapiens	SHAE003_BAT_96h_1_Cells	Cells	SHAE003-R	SHAE003_BAT_96h_1_array	RNA			
SHAE005	SHAE003 HAE cells	Homo sapiens	SHAE003_BAT_96h_2_Cells	Cells	SHAE003-R	SHAE003_BAT_96h_2_array	RNA			
SHAE005	SHAE003 HAE cells	Homo sapiens	SHAE003_BAT_96h_3_Cells	Cells	SHAE003-R	SHAE003_BAT_96h_3_array	RNA			
SHAE005	SHAE003 HAE cells	Homo sapiens	SHAE003_BAT_96h_4_Cells	Cells	SHAE003-R	SHAE003_BAT_96h_4_array	RNA			
SHAE005	SHAE003 HAE cells	Homo sapiens	SHAE003_dORF6_0h_1_Cells	Cells	SHAE003-R	SHAE003_dORF6_0h_1_array	RNA			
SHAE005	SHAE003 HAE cells	Homo sapiens	SHAE003_dORF6_0h_2_Cells	Cells	SHAE003-R	SHAE003_dORF6_0h_2_array	RNA			
SHAE005	SHAE003 HAE cells	Homo sapiens	SHAE003_dORF6_0h_3_Cells	Cells	SHAE003-R	SHAE003_dORF6_0h_3_array	RNA			
SHAE005	SHAE003 HAE cells	Homo sapiens	SHAE003_dORF6_0h_4_Cells	Cells	SHAE003-R	SHAE003_dORF6_0h_4_array	RNA			
SHAE005	SHAE003 HAE cells	Homo sapiens	SHAE003_dORF6_24h_1_Cells	Cells	SHAE003-R	SHAE003_dORF6_24h_1_array	RNA			
SHAE005	SHAE003 HAE cells	Homo sapiens	SHAE003_dORF6_24h_2_Cells	Cells	SHAE003-R	SHAE003_dORF6_24h_2_array	RNA			
SHAE005	SHAE003 HAE cells	Homo sapiens	SHAE003_dORF6_24h_3_Cells	Cells	SHAE003-R	SHAE003_dORF6_24h_3_array	RNA			
SHAE005	SHAE003 HAE cells	Homo sapiens	SHAE003_dORF6_48h_1_Cells	Cells	SHAE003-R	SHAE003_dORF6_48h_1_array	RNA			
SHAE005	SHAE003 HAE cells	Homo sapiens	SHAE003_dORF6_48h_2_Cells	Cells	SHAE003-R	SHAE003_dORF6_48h_2_array	RNA			
SHAE005	SHAE003 HAE cells	Homo sapiens	SHAE003_dORF6_48h_3_Cells	Cells	SHAE003-R	SHAE003_dORF6_48h_3_array	RNA			
SHAE005	SHAE003 HAE cells	Homo sapiens	SHAE003_dORF6_48h_4_Cells	Cells	SHAE003-R	SHAE003_dORF6_48h_4_array	RNA			
SHAE005	SHAE003 HAE cells	Homo sapiens	SHAE003_dORF6_60h_1_Cells	Cells	SHAE003-R	SHAE003_dORF6_60h_1_array	RNA			
SHAE005	SHAE003 HAE cells	Homo sapiens	SHAE003_dORF6_60h_2_Cells	Cells	SHAE003-R	SHAE003_dORF6_60h_2_array	RNA			
SHAE005	SHAE003 HAE cells	Homo sapiens	SHAE003_dORF6_60h_3_Cells	Cells	SHAE003-R	SHAE003_dORF6_60h_3_array	RNA			
SHAE005	SHAE003 HAE cells	Homo sapiens	SHAE003_dORF6_60h_4_Cells	Cells	SHAE003-R	SHAE003_dORF6_60h_4_array	RNA			
SHAE005	SHAE003 HAE cells	Homo sapiens	SHAE003_dORF6_72h_1_Cells	Cells	SHAE003-R	SHAE003_dORF6_72h_1_array	RNA			
SHAE005	SHAE003 HAE cells	Homo sapiens	SHAE003_dORF6_72h_2_Cells	Cells	SHAE003-R	SHAE003_dORF6_72h_2_array	RNA			
SHAE005	SHAE003 HAE cells	Homo sapiens	SHAE003_dORF6_72h_3_Cells	Cells	SHAE003-R	SHAE003_dORF6_72h_3_array	RNA			
SHAE005	SHAE003 HAE cells	Homo sapiens	SHAE003_dORF6_72h_4_Cells	Cells	SHAE003-R	SHAE003_dORF6_72h_4_array	RNA			
SHAE005	SHAE003 HAE cells	Homo sapiens	SHAE003_dORF6_84h_1_Cells	Cells	SHAE003-R	SHAE003_dORF6_84h_1_array	RNA			
SHAE005	SHAE003 HAE cells	Homo sapiens	SHAE003_dORF6_84h_2_Cells	Cells	SHAE003-R	SHAE003_dORF6_84h_2_array	RNA			
SHAE005	SHAE003 HAE cells	Homo sapiens	SHAE003_dORF6_84h_3_Cells	Cells	SHAE003-R	SHAE003_dORF6_84h_3_array	RNA			
SHAE005	SHAE003 HAE cells	Homo sapiens	SHAE003_dORF6_84h_4_Cells	Cells	SHAE003-R	SHAE003_dORF6_84h_4_array	RNA			
SHAE005	SHAE003 HAE cells	Homo sapiens	SHAE003_dORF6_96h_1_Cells	Cells	SHAE003-R	SHAE003_dORF6_96h_1_array	RNA			
SHAE005	SHAE003 HAE cells	Homo sapiens	SHAE003_dORF6_96h_2_Cells	Cells	SHAE003-R	SHAE003_dORF6_96h_2_array	RNA			
SHAE005	SHAE003 HAE cells	Homo sapiens	SHAE003_dORF6_96h_3_Cells	Cells	SHAE003-R	SHAE003_dORF6_96h_3_array	RNA			
SHAE005	SHAE003 HAE cells	Homo sapiens	SHAE003_dORF6_96h_4_Cells	Cells	SHAE003-R	SHAE003_dORF6_96h_4_array	RNA			
SHAE005	SHAE003 HAE cells	Homo sapiens	SHAE003_H1N1_0h_1_Cells	Cells	SHAE003-R	SHAE003_H1N1_0h_1_array	RNA			
SHAE005	SHAE003 HAE cells	Homo sapiens	SHAE003_H1N1_0h_2_Cells	Cells	SHAE003-R	SHAE003_H1N1_0h_2_array	RNA			
SHAE005	SHAE003 HAE cells	Homo sapiens	SHAE003_H1N1_0h_3_Cells	Cells	SHAE003-R	SHAE003_H1N1_0h_3_array	RNA			
SHAE005	SHAE003 HAE cells	Homo sapiens	SHAE003_H1N1_0h_4_Cells	Cells	SHAE003-R		RNA			
SHAE005	SHAE003 HAE cells	Homo sapiens	SHAE003_H1N1_6h_1_Cells	Cells	SHAE003-R	SHAE003_H1N1_6h_1_array	RNA			
SHAE005	SHAE003 HAE cells	Homo sapiens	SHAE003_H1N1_6h_2_Cells	Cells	SHAE003-R	SHAE003_H1N1_6h_2_array	RNA			
SHAE005	SHAE003 HAE cells	Homo sapiens	SHAE003_H1N1_6h_3_Cells	Cells	SHAE003-R	SHAE003_H1N1_6h_3_array	RNA			
SHAE005	SHAE003 HAE cells	Homo sapiens	SHAE003_H1N1_6h_4_Cells	Cells	SHAE003-R		RNA			
SHAE005	SHAE003 HAE cells	Homo sapiens	SHAE003_H1N1_12h_1_Cells	Cells	SHAE003-R	SHAE003_H1N1_12h_1_array	RNA			
SHAE005	SHAE003 HAE cells	Homo sapiens	SHAE003_H1N1_12h_2_Cells	Cells	SHAE003-R	SHAE003_H1N1_12h_2_array	RNA			
SHAE005	SHAE003 HAE cells	Homo sapiens	SHAE003_H1N1_12h_3_Cells	Cells	SHAE003-R	SHAE003_H1N1_12h_3_array	RNA			
SHAE005	SHAE003 HAE cells	Homo sapiens	SHAE003_H1N1_12h_4_Cells	Cells	SHAE003-R		RNA			
SHAE005	SHAE003 HAE cells	Homo sapiens	SHAE003_H1N1_18h_1_Cells	Cells	SHAE003-R	SHAE003_H1N1_18h_1_array	RNA			
SHAE005	SHAE003 HAE cells	Homo sapiens	SHAE003_H1N1_18h_2_Cells	Cells	SHAE003-R	SHAE003_H1N1_18h_2_array	RNA			
SHAE005	SHAE003 HAE cells	Homo sapiens	SHAE003_H1N1_18h_3_Cells	Cells	SHAE003-R	SHAE003_H1N1_18h_3_array	RNA			
SHAE005	SHAE003 HAE cells	Homo sapiens	SHAE003_H1N1_18h_4_Cells	Cells	SHAE003-R		RNA			
SHAE005	SHAE003 HAE cells	Homo sapiens	SHAE003_H1N1_24h_1_Cells	Cells	SHAE003-R	SHAE003_H1N1_24h_1_array	RNA			
SHAE005	SHAE003 HAE cells	Homo sapiens	SHAE003_H1N1_24h_2_Cells	Cells	SHAE003-R	SHAE003_H1N1_24h_2_array	RNA			
SHAE005	SHAE003 HAE cells	Homo sapiens	SHAE003_H1N1_24h_3_Cells	Cells	SHAE003-R	SHAE003_H1N1_24h_3_array	RNA			
SHAE005	SHAE003 HAE cells	Homo sapiens	SHAE003_H1N1_24h_4_Cells	Cells	SHAE003-R		RNA			
SHAE005	SHAE003 HAE cells	Homo sapiens	SHAE003_H1N1_36h_1_Cells	Cells	SHAE003-R	SHAE003_H1N1_36h_1_array	RNA			
SHAE005	SHAE003 HAE cells	Homo sapiens	SHAE003_H1N1_36h_2_Cells	Cells	SHAE003-R	SHAE003_H1N1_36h_2_array	RNA			
SHAE005	SHAE003 HAE cells	Homo sapiens	SHAE003_H1N1_36h_3_Cells	Cells	SHAE003-R	SHAE003_H1N1_36h_3_array	RNA			
SHAE005	SHAE003 HAE cells	Homo sapiens	SHAE003_H1N1_36h_4_Cells	Cells	SHAE003-R		RNA			
SHAE005	SHAE003 HAE cells	Homo sapiens	SHAE003_H1N1_48h_1_Cells	Cells	SHAE003-R	SHAE003_H1N1_48h_1_array	RNA			
SHAE005	SHAE003 HAE cells	Homo sapiens	SHAE003_H1N1_48h_2_Cells	Cells	SHAE003-R	SHAE003_H1N1_48h_2_array	RNA			
SHAE005	SHAE003 HAE cells	Homo sapiens	SHAE003_H1N1_48h_3_Cells	Cells	SHAE003-R	SHAE003_H1N1_48h_3_array	RNA			
SHAE005	SHAE003 HAE cells	Homo sapiens	SHAE003_H1N1_48h_4_Cells	Cells	SHAE003-R	SHAE003_H1N1_48h_4_array	RNA			
SHAE005	SHAE003 HAE cells	Homo sapiens	SHAE003_Mock_0h_1_Cells	Cells	SHAE003-R	SHAE003_Mock_0h_1_array	RNA			
SHAE005	SHAE003 HAE cells	Homo sapiens	SHAE003_Mock_0h_2_Cells	Cells	SHAE003-R	SHAE003_Mock_0h_2_array	RNA			
SHAE005	SHAE003 HAE cells	Homo sapiens	SHAE003_Mock_0h_3_Cells	Cells	SHAE003-R	SHAE003_Mock_0h_3_array	RNA			
SHAE005	SHAE003 HAE cells	Homo sapiens	SHAE003_Mock_6h_1_Cells	Cells	SHAE003-R	SHAE003_Mock_6h_1_array	RNA			
SHAE005	SHAE003 HAE cells	Homo sapiens	SHAE003_Mock_6h_2_Cells	Cells	SHAE003-R	SHAE003_Mock_6h_2_array	RNA			
SHAE005	SHAE003 HAE cells	Homo sapiens	SHAE003_Mock_6h_3_Cells	Cells	SHAE003-R	SHAE003_Mock_6h_3_array	RNA			
SHAE005	SHAE003 HAE cells	Homo sapiens	SHAE003_Mock_12h_1_Cells	Cells	SHAE003-R	SHAE003_Mock_12h_1_array	RNA			
SHAE005	SHAE003 HAE cells	Homo sapiens	SHAE003_Mock_12h_2_Cells	Cells	SHAE003-R	SHAE003_Mock_12h_2_array	RNA			
SHAE005	SHAE003 HAE cells	Homo sapiens	SHAE003_Mock_12h_3_Cells	Cells	SHAE003-R	SHAE003_Mock_12h_3_array	RNA			
SHAE005	SHAE003 HAE cells	Homo sapiens	SHAE003_Mock_18h_1_Cells	Cells	SHAE003-R	SHAE003_Mock_18h_1_array	RNA			
SHAE005	SHAE003 HAE cells	Homo sapiens	SHAE003_Mock_18h_2_Cells	Cells	SHAE003-R		RNA			
SHAE005	SHAE003 HAE cells	Homo sapiens	SHAE003_Mock_18h_3_Cells	Cells	SHAE003-R	SHAE003_Mock_18h_3_array	RNA			
SHAE005	SHAE003 HAE cells	Homo sapiens	SHAE003_Mock_24h_1_Cells	Cells	SHAE003-R	SHAE003_Mock_24h_1_array	RNA			
SHAE005	SHAE003 HAE cells	Homo sapiens	SHAE003_Mock_24h_2_Cells	Cells	SHAE003-R	SHAE003_Mock_24h_2_array	RNA			
SHAE005	SHAE003 HAE cells	Homo sapiens	SHAE003_Mock_24h_3_Cells	Cells	SHAE003-R	SHAE003_Mock_24h_3_array	RNA			
SHAE005	SHAE003 HAE cells	Homo sapiens	SHAE003_Mock_36h_1_Cells	Cells	SHAE003-R	SHAE003_Mock_36h_1_array	RNA			
SHAE005	SHAE003 HAE cells	Homo sapiens	SHAE003_Mock_36h_2_Cells	Cells	SHAE003-R	SHAE003_Mock_36h_2_array	RNA			
SHAE005	SHAE003 HAE cells	Homo sapiens	SHAE003_Mock_36h_3_Cells	Cells	SHAE003-R	SHAE003_Mock_36h_3_array	RNA			
SHAE005	SHAE003 HAE cells	Homo sapiens	SHAE003_Mock_48h_1_Cells	Cells	SHAE003-R	SHAE003_Mock_48h_1_array	RNA			
SHAE005	SHAE003 HAE cells	Homo sapiens	SHAE003_Mock_48h_2_Cells	Cells	SHAE004-R	SHAE003_Mock_48h_2_array	RNA			
SHAE005	SHAE003 HAE cells	Homo sapiens	SHAE003_Mock_48h_3_Cells	Cells	SHAE004-R	SHAE003_Mock_48h_3_array	RNA			
SHAE005	SHAE003 HAE cells	Homo sapiens	SHAE003_Mock_60h_1_Cells	Cells	SHAE004-R	SHAE003_Mock_60h_1_array	RNA			
SHAE005	SHAE003 HAE cells	Homo sapiens	SHAE003_Mock_60h_2_Cells	Cells	SHAE004-R	SHAE003_Mock_60h_2_array	RNA			
SHAE005	SHAE003 HAE cells	Homo sapiens	SHAE003_Mock_60h_3_Cells	Cells	SHAE004-R	SHAE003_Mock_60h_3_array	RNA			
SHAE005	SHAE003 HAE cells	Homo sapiens	SHAE003_Mock_72h_1_Cells	Cells	SHAE004-R	SHAE003_Mock_72h_1_array	RNA			
SHAE005	SHAE003 HAE cells	Homo sapiens	SHAE003_Mock_72h_2_Cells	Cells	SHAE004-R	SHAE003_Mock_72h_2_array	RNA			
SHAE005	SHAE003 HAE cells	Homo sapiens	SHAE003_Mock_72h_3_Cells	Cells	SHAE004-R	SHAE003_Mock_72h_3_array	RNA			
SHAE005	SHAE003 HAE cells	Homo sapiens	SHAE003_Mock_84h_1_Cells	Cells	SHAE004-R	SHAE003_Mock_84h_1_array	RNA			
SHAE005	SHAE003 HAE cells	Homo sapiens	SHAE003_Mock_84h_2_Cells	Cells	SHAE004-R	SHAE003_Mock_84h_2_array	RNA			
SHAE005	SHAE003 HAE cells	Homo sapiens	SHAE003_Mock_84h_3_Cells	Cells	SHAE004-R	SHAE003_Mock_84h_3_array	RNA			
SHAE005	SHAE003 HAE cells	Homo sapiens	SHAE003_Mock_96h_1_Cells	Cells	SHAE004-R	SHAE003_Mock_96h_1_array	RNA			
SHAE005	SHAE003 HAE cells	Homo sapiens	SHAE003_Mock_96h_2_Cells	Cells	SHAE004-R	SHAE003_Mock_96h_2_array	RNA			
SHAE005	SHAE003 HAE cells	Homo sapiens	SHAE003_Mock_96h_3_Cells	Cells	SHAE004-R	SHAE003_Mock_96h_3_array	RNA			
SHAE005	SHAE004 HAE cells	Homo sapiens	SHAE004_Mock_18h_3_Cells	Cells	SHAE004-R	SHAE004_Mock_18h_3_array	RNA			
SHAE005	SHAE004 HAE cells	Homo sapiens	SHAE004_Mock_24h_1_Cells	Cells	SHAE004-R	SHAE004_Mock_24h_1_array	RNA			
SHAE005	SHAE004 HAE cells	Homo sapiens	SHAE004_Mock_24h_2_Cells	Cells	SHAE004-R	SHAE004_Mock_24h_2_array	RNA			
SHAE005	SHAE004 HAE cells	Homo sapiens	SHAE004_Mock_24h_3_Cells	Cells	SHAE004-R	SHAE004_Mock_24h_3_array	RNA			
SHAE005	SHAE004 HAE cells	Homo sapiens	SHAE004_Mock_36h_1_Cells	Cells	SHAE004-R	SHAE004_Mock_36h_1_array	RNA			
SHAE005	SHAE004 HAE cells	Homo sapiens	SHAE004_Mock_36h_2_Cells	Cells	SHAE004-R	SHAE004_Mock_36h_2_array	RNA			
SHAE005	SHAE004 HAE cells	Homo sapiens	SHAE004_Mock_36h_3_Cells	Cells	SHAE004-R	SHAE004_Mock_36h_3_array	RNA			
SHAE005	SHAE004 HAE cells	Homo sapiens	SHAE004_Mock_48h_1_Cells	Cells	SHAE004-R	SHAE004_Mock_48h_1_array	RNA			
SHAE005	SHAE004 HAE cells	Homo sapiens	SHAE004_Mock_48h_2_Cells	Cells	SHAE004-R	SHAE004_Mock_48h_2_array	RNA			
SHAE005	SHAE004 HAE cells	Homo sapiens	SHAE004_Mock_48h_3_Cells	Cells	SHAE004-R	SHAE004_Mock_48h_3_array	RNA			
SHAE005	SHAE004 HAE cells	Homo sapiens	SHAE004_Mock_60h_1_Cells	Cells	SHAE004-R	SHAE004_Mock_60h_1_array	RNA			
SHAE005	SHAE004 HAE cells	Homo sapiens	SHAE004_Mock_60h_2_Cells	Cells	SHAE004-R	SHAE004_Mock_60h_2_array	RNA			
SHAE005	SHAE004 HAE cells	Homo sapiens	SHAE004_Mock_60h_3_Cells	Cells	SHAE004-R	SHAE004_Mock_60h_3_array	RNA			
SHAE005	SHAE004 HAE cells	Homo sapiens	SHAE004_Mock_72h_1_Cells	Cells	SHAE004-R	SHAE004_Mock_72h_1_array	RNA			
SHAE005	SHAE004 HAE cells	Homo sapiens	SHAE004_Mock_72h_2_Cells	Cells	SHAE004-R	SHAE004_Mock_72h_2_array	RNA			
SHAE005	SHAE004 HAE cells	Homo sapiens	SHAE004_Mock_72h_3_Cells	Cells	SHAE004-R	SHAE004_Mock_72h_3_array	RNA			
SHAE005	SHAE004 HAE cells	Homo sapiens	SHAE004_Mock_84h_1_Cells	Cells	SHAE004-R	SHAE004_Mock_84h_1_array	RNA			
SHAE005	SHAE004 HAE cells	Homo sapiens	SHAE004_Mock_84h_2_Cells	Cells	SHAE004-R	SHAE004_Mock_84h_2_array	RNA			
SHAE005	SHAE004 HAE cells	Homo sapiens	SHAE004_Mock_84h_3_Cells	Cells	SHAE004-R	SHAE004_Mock_84h_3_array	RNA			
SHAE005	SHAE004 HAE cells	Homo sapiens	SHAE004_Mock_96h_1_Cells	Cells	SHAE004-R	SHAE004_Mock_96h_1_array	RNA			
SHAE005	SHAE004 HAE cells	Homo sapiens	SHAE004_Mock_96h_2_Cells	Cells	SHAE004-R	SHAE004_Mock_96h_2_array	RNA			
SHAE005	SHAE004 HAE cells	Homo sapiens	SHAE004_Mock_96h_3_Cells	Cells	SHAE004-R	SHAE004_Mock_96h_3_array	RNA			
SHAE005	SHAE004 HAE cells	Homo sapiens	SHAE004_SARS_0h_1_Cells	Cells	SHAE004-R	SHAE004_SARS_0h_1_array	RNA			
SHAE005	SHAE004 HAE cells	Homo sapiens	SHAE004_SARS_0h_2_Cells	Cells	SHAE004-R	SHAE004_SARS_0h_2_array	RNA			
SHAE005	SHAE004 HAE cells	Homo sapiens	SHAE004_SARS_0h_3_Cells	Cells	SHAE004-R	SHAE004_SARS_0h_3_array	RNA			
SHAE005	SHAE004 HAE cells	Homo sapiens	SHAE004_SARS_12h_1_Cells	Cells	SHAE004-R	SHAE004_SARS_12h_1_array	RNA			
SHAE005	SHAE004 HAE cells	Homo sapiens	SHAE004_SARS_12h_2_Cells	Cells	SHAE004-R	SHAE004_SARS_12h_2_array	RNA			
SHAE005	SHAE004 HAE cells	Homo sapiens	SHAE004_SARS_12h_3_Cells	Cells	SHAE004-R	SHAE004_SARS_12h_3_array	RNA			
SHAE005	SHAE004 HAE cells	Homo sapiens	SHAE004_SARS_24h_1_Cells	Cells	SHAE004-R	SHAE004_SARS_24h_1_array	RNA			
SHAE005	SHAE004 HAE cells	Homo sapiens	SHAE004_SARS_24h_2_Cells	Cells	SHAE004-R	SHAE004_SARS_24h_2_array	RNA			
SHAE005	SHAE004 HAE cells	Homo sapiens	SHAE004_SARS_24h_3_Cells	Cells	SHAE004-R	SHAE004_SARS_24h_3_array	RNA			
SHAE005	SHAE004 HAE cells	Homo sapiens	SHAE004_SARS_36h_1_Cells	Cells	SHAE004-R	SHAE004_SARS_36h_1_array	RNA			
SHAE005	SHAE004 HAE cells	Homo sapiens	SHAE004_SARS_36h_2_Cells	Cells	SHAE004-R	SHAE004_SARS_36h_2_array	RNA			
SHAE005	SHAE004 HAE cells	Homo sapiens	SHAE004_SARS_36h_3_Cells	Cells	SHAE004-R	SHAE004_SARS_36h_3_array	RNA			
SHAE005	SHAE004 HAE cells	Homo sapiens	SHAE004_SARS_48h_1_Cells	Cells	SHAE004-R	SHAE004_SARS_48h_1_array	RNA			
SHAE005	SHAE004 HAE cells	Homo sapiens	SHAE004_SARS_48h_2_Cells	Cells	SHAE004-R	SHAE004_SARS_48h_2_array	RNA			
SHAE005	SHAE004 HAE cells	Homo sapiens	SHAE004_SARS_48h_3_Cells	Cells	SHAE004-R	SHAE004_SARS_48h_3_array	RNA			
SHAE005	SHAE004 HAE cells	Homo sapiens	SHAE004_SARS_60h_1_Cells	Cells	SHAE004-R	SHAE004_SARS_60h_1_array	RNA			
SHAE005	SHAE004 HAE cells	Homo sapiens	SHAE004_SARS_60h_2_Cells	Cells	SHAE004-R	SHAE004_SARS_60h_2_array	RNA			
SHAE005	SHAE004 HAE cells	Homo sapiens	SHAE004_SARS_60h_3_Cells	Cells	SHAE004-R	SHAE004_SARS_60h_3_array	RNA			
SHAE005	SHAE004 HAE cells	Homo sapiens	SHAE004_SARS_72h_1_Cells	Cells	SHAE004-R	SHAE004_SARS_72h_1_array	RNA			
SHAE005	SHAE004 HAE cells	Homo sapiens	SHAE004_SARS_72h_2_Cells	Cells	SHAE004-R	SHAE004_SARS_72h_2_array	RNA			
SHAE005	SHAE004 HAE cells	Homo sapiens	SHAE004_SARS_72h_3_Cells	Cells	SHAE004-R	SHAE004_SARS_72h_3_array	RNA			
SHAE005	SHAE004 HAE cells	Homo sapiens	SHAE004_SARS_84h_1_Cells	Cells	SHAE004-R	SHAE004_SARS_84h_1_array	RNA			
SHAE005	SHAE004 HAE cells	Homo sapiens	SHAE004_SARS_84h_2_Cells	Cells	SHAE004-R	SHAE004_SARS_84h_2_array	RNA			
SHAE005	SHAE004 HAE cells	Homo sapiens	SHAE004_SARS_84h_3_Cells	Cells	SHAE004-R	SHAE004_SARS_84h_3_array	RNA			
SHAE005	SHAE004 HAE cells	Homo sapiens	SHAE004_SARS_96h_1_Cells	Cells	SHAE004-R	SHAE004_SARS_96h_1_array	RNA			
SHAE005	SHAE004 HAE cells	Homo sapiens	SHAE004_SARS_96h_2_Cells	Cells	SHAE004-R	SHAE004_SARS_96h_2_array	RNA			
SHAE005	SHAE004 HAE cells	Homo sapiens	SHAE004_SARS_96h_3_Cells	Cells	SHAE004-R	SHAE004_SARS_96h_3_array	RNA			
SHAE005	SHAE004 HAE cells	Homo sapiens	SHAE004_BAT_0h_1_Cells	Cells	SHAE004-R	SHAE004_BAT_0h_1_array	RNA			
SHAE005	SHAE004 HAE cells	Homo sapiens	SHAE004_BAT_0h_2_Cells	Cells	SHAE004-R	SHAE004_BAT_0h_2_array	RNA			
SHAE005	SHAE004 HAE cells	Homo sapiens	SHAE004_BAT_0h_3_Cells	Cells	SHAE004-R	SHAE004_BAT_0h_3_array	RNA			
SHAE005	SHAE004 HAE cells	Homo sapiens	SHAE004_BAT_12h_1_Cells	Cells	SHAE004-R	SHAE004_BAT_12h_1_array	RNA			
SHAE005	SHAE004 HAE cells	Homo sapiens	SHAE004_BAT_12h_2_Cells	Cells	SHAE004-R	SHAE004_BAT_12h_2_array	RNA			
SHAE005	SHAE004 HAE cells	Homo sapiens	SHAE004_BAT_12h_3_Cells	Cells	SHAE004-R	SHAE004_BAT_12h_3_array	RNA			
SHAE005	SHAE004 HAE cells	Homo sapiens	SHAE004_BAT_24h_1_Cells	Cells	SHAE004-R	SHAE004_BAT_24h_1_array	RNA			
SHAE005	SHAE004 HAE cells	Homo sapiens	SHAE004_BAT_24h_2_Cells	Cells	SHAE004-R	SHAE004_BAT_24h_2_array	RNA			
SHAE005	SHAE004 HAE cells	Homo sapiens	SHAE004_BAT_36h_1_Cells	Cells	SHAE004-R	SHAE004_BAT_36h_1_array	RNA			
SHAE005	SHAE004 HAE cells	Homo sapiens	SHAE004_BAT_36h_2_Cells	Cells	SHAE004-R	SHAE004_BAT_36h_2_array	RNA			
SHAE005	SHAE004 HAE cells	Homo sapiens	SHAE004_BAT_36h_3_Cells	Cells	SHAE004-R	SHAE004_BAT_36h_3_array	RNA			
SHAE005	SHAE004 HAE cells	Homo sapiens	SHAE004_BAT_48h_1_Cells	Cells	SHAE004-R	SHAE004_BAT_48h_1_array	RNA			
SHAE005	SHAE004 HAE cells	Homo sapiens	SHAE004_BAT_48h_2_Cells	Cells	SHAE004-R	SHAE004_BAT_48h_2_array	RNA			
SHAE005	SHAE004 HAE cells	Homo sapiens	SHAE004_BAT_48h_3_Cells	Cells	SHAE004-R	SHAE004_BAT_48h_3_array	RNA			
SHAE005	SHAE004 HAE cells	Homo sapiens	SHAE004_BAT_60h_1_Cells	Cells	SHAE004-R	SHAE004_BAT_60h_1_array	RNA			
SHAE005	SHAE004 HAE cells	Homo sapiens	SHAE004_BAT_60h_2_Cells	Cells	SHAE004-R	SHAE004_BAT_60h_2_array	RNA			
SHAE005	SHAE004 HAE cells	Homo sapiens	SHAE004_BAT_60h_3_Cells	Cells	SHAE004-R	SHAE004_BAT_60h_3_array	RNA			
SHAE005	SHAE004 HAE cells	Homo sapiens	SHAE004_BAT_72h_1_Cells	Cells	SHAE004-R	SHAE004_BAT_72h_1_array	RNA			
SHAE005	SHAE004 HAE cells	Homo sapiens	SHAE004_BAT_72h_2_Cells	Cells	SHAE004-R	SHAE004_BAT_72h_2_array	RNA			
SHAE005	SHAE004 HAE cells	Homo sapiens	SHAE004_BAT_72h_3_Cells	Cells	SHAE004-R	SHAE004_BAT_72h_3_array	RNA			
SHAE005	SHAE004 HAE cells	Homo sapiens	SHAE004_BAT_84h_1_Cells	Cells	SHAE004-R	SHAE004_BAT_84h_1_array	RNA			
SHAE005	SHAE004 HAE cells	Homo sapiens	SHAE004_BAT_84h_2_Cells	Cells	SHAE004-R	SHAE004_BAT_84h_2_array	RNA			
SHAE005	SHAE004 HAE cells	Homo sapiens	SHAE004_BAT_84h_3_Cells	Cells	SHAE004-R	SHAE004_BAT_84h_3_array	RNA			
SHAE005	SHAE004 HAE cells	Homo sapiens	SHAE004_BAT_96h_1_Cells	Cells	SHAE004-R	SHAE004_BAT_96h_1_array	RNA			
SHAE005	SHAE004 HAE cells	Homo sapiens	SHAE004_BAT_96h_2_Cells	Cells	SHAE004-R	SHAE004_BAT_96h_2_array	RNA			
SHAE005	SHAE004 HAE cells	Homo sapiens	SHAE004_BAT_96h_3_Cells	Cells	SHAE004-R	SHAE004_BAT_96h_3_array	RNA			
SHAE005	SHAE004 HAE cells	Homo sapiens	SHAE004_dORF6_0h_1_Cells	Cells	SHAE004-R	SHAE004_dORF6_0h_1_array	RNA			
SHAE005	SHAE004 HAE cells	Homo sapiens	SHAE004_dORF6_0h_2_Cells	Cells	SHAE004-R	SHAE004_dORF6_0h_2_array	RNA			
SHAE005	SHAE004 HAE cells	Homo sapiens	SHAE004_dORF6_0h_3_Cells	Cells	SHAE004-R	SHAE004_dORF6_0h_3_array	RNA			
SHAE005	SHAE004 HAE cells	Homo sapiens	SHAE004_dORF6_12h_1_Cells	Cells	SHAE004-R	SHAE004_dORF6_12h_1_array	RNA			
SHAE005	SHAE004 HAE cells	Homo sapiens	SHAE004_dORF6_12h_2_Cells	Cells	SHAE004-R	SHAE004_dORF6_12h_2_array	RNA			
SHAE005	SHAE004 HAE cells	Homo sapiens	SHAE004_dORF6_12h_3_Cells	Cells	SHAE004-R	SHAE004_dORF6_12h_3_array	RNA			
SHAE005	SHAE004 HAE cells	Homo sapiens	SHAE004_dORF6_24h_1_Cells	Cells	SHAE004-R	SHAE004_dORF6_24h_1_array	RNA			
SHAE005	SHAE004 HAE cells	Homo sapiens	SHAE004_dORF6_24h_2_Cells	Cells	SHAE004-R	SHAE004_dORF6_24h_2_array	RNA			
SHAE005	SHAE004 HAE cells	Homo sapiens	SHAE004_dORF6_24h_3_Cells	Cells	SHAE004-R	SHAE004_dORF6_24h_3_array	RNA			
SHAE005	SHAE004 HAE cells	Homo sapiens	SHAE004_dORF6_36h_1_Cells	Cells	SHAE004-R	SHAE004_dORF6_36h_1_array	RNA			
SHAE005	SHAE004 HAE cells	Homo sapiens	SHAE004_dORF6_36h_2_Cells	Cells	SHAE004-R	SHAE004_dORF6_36h_2_array	RNA			
SHAE005	SHAE004 HAE cells	Homo sapiens	SHAE004_dORF6_36h_3_Cells	Cells	SHAE004-R	SHAE004_dORF6_36h_3_array	RNA			
SHAE005	SHAE004 HAE cells	Homo sapiens	SHAE004_dORF6_48h_1_Cells	Cells	SHAE004-R	SHAE004_dORF6_48h_1_array	RNA			
SHAE005	SHAE004 HAE cells	Homo sapiens	SHAE004_dORF6_48h_2_Cells	Cells	SHAE004-R	SHAE004_dORF6_48h_2_array	RNA			
SHAE005	SHAE004 HAE cells	Homo sapiens	SHAE004_dORF6_48h_3_Cells	Cells	SHAE004-R	SHAE004_dORF6_48h_3_array	RNA			
SHAE005	SHAE004 HAE cells	Homo sapiens	SHAE004_dORF6_60h_1_Cells	Cells	SHAE004-R	SHAE004_dORF6_60h_1_array	RNA			
SHAE005	SHAE004 HAE cells	Homo sapiens	SHAE004_dORF6_60h_2_Cells	Cells	SHAE004-R	SHAE004_dORF6_60h_2_array	RNA			
SHAE005	SHAE004 HAE cells	Homo sapiens	SHAE004_dORF6_60h_3_Cells	Cells	SHAE004-R	SHAE004_dORF6_60h_3_array	RNA			
SHAE005	SHAE004 HAE cells	Homo sapiens	SHAE004_dORF6_72h_1_Cells	Cells	SHAE004-R	SHAE004_dORF6_72h_1_array	RNA			
SHAE005	SHAE004 HAE cells	Homo sapiens	SHAE004_dORF6_72h_2_Cells	Cells	SHAE004-R	SHAE004_dORF6_72h_2_array	RNA			
SHAE005	SHAE004 HAE cells	Homo sapiens	SHAE004_dORF6_72h_3_Cells	Cells	SHAE004-R	SHAE004_dORF6_72h_3_array	RNA			
SHAE005	SHAE004 HAE cells	Homo sapiens	SHAE004_dORF6_84h_1_Cells	Cells	SHAE004-R	SHAE004_dORF6_84h_1_array	RNA			
SHAE005	SHAE004 HAE cells	Homo sapiens	SHAE004_dORF6_84h_2_Cells	Cells	SHAE004-R	SHAE004_dORF6_84h_2_array	RNA			
SHAE005	SHAE004 HAE cells	Homo sapiens	SHAE004_dORF6_84h_3_Cells	Cells	SHAE004-R	SHAE004_dORF6_84h_3_array	RNA			
SHAE005	SHAE004 HAE cells	Homo sapiens	SHAE004_dORF6_96h_1_Cells	Cells	SHAE004-R	SHAE004_dORF6_96h_1_array	RNA			
SHAE005	SHAE004 HAE cells	Homo sapiens	SHAE004_dORF6_96h_2_Cells	Cells	SHAE004-R	SHAE004_dORF6_96h_2_array	RNA			
SHAE005	SHAE004 HAE cells	Homo sapiens	SHAE004_dORF6_96h_3_Cells	Cells	SHAE004-R	SHAE004_dORF6_96h_3_array	RNA			
SHAE005	SHAE004 HAE cells	Homo sapiens	SHAE004_H1N1_0h_1_Cells	Cells	SHAE004-R	SHAE004_H1N1_0h_1_array	RNA			
SHAE005	SHAE004 HAE cells	Homo sapiens	SHAE004_H1N1_0h_2_Cells	Cells	SHAE004-R	SHAE004_H1N1_0h_2_array	RNA			
SHAE005	SHAE004 HAE cells	Homo sapiens	SHAE004_H1N1_0h_3_Cells	Cells	SHAE004-R	SHAE004_H1N1_0h_3_array	RNA			
SHAE005	SHAE004 HAE cells	Homo sapiens	SHAE004_H1N1_6h_1_Cells	Cells	SHAE004-R	SHAE004_H1N1_12h_1_array	RNA			
SHAE005	SHAE004 HAE cells	Homo sapiens	SHAE004_H1N1_6h_2_Cells	Cells	SHAE004-R	SHAE004_H1N1_12h_2_array	RNA			
SHAE005	SHAE004 HAE cells	Homo sapiens	SHAE004_H1N1_6h_3_Cells	Cells	SHAE004-R	SHAE004_H1N1_12h_3_array	RNA			
SHAE005	SHAE004 HAE cells	Homo sapiens	SHAE004_H1N1_12h_1_Cells	Cells	SHAE004-R	SHAE004_H1N1_18h_1_array	RNA			
SHAE005	SHAE004 HAE cells	Homo sapiens	SHAE004_H1N1_12h_2_Cells	Cells	SHAE004-R	SHAE004_H1N1_18h_2_array	RNA			
SHAE005	SHAE004 HAE cells	Homo sapiens	SHAE004_H1N1_12h_3_Cells	Cells	SHAE004-R	SHAE004_H1N1_18h_3_array	RNA			
SHAE005	SHAE004 HAE cells	Homo sapiens	SHAE004_H1N1_18h_1_Cells	Cells	SHAE004-R	SHAE004_H1N1_24h_1_array	RNA			
SHAE005	SHAE004 HAE cells	Homo sapiens	SHAE004_H1N1_18h_2_Cells	Cells	SHAE004-R	SHAE004_H1N1_24h_2_array	RNA			
SHAE005	SHAE004 HAE cells	Homo sapiens	SHAE004_H1N1_18h_3_Cells	Cells	SHAE004-R	SHAE004_H1N1_24h_3_array	RNA			
SHAE005	SHAE004 HAE cells	Homo sapiens	SHAE004_H1N1_24h_1_Cells	Cells	SHAE004-R	SHAE004_H1N1_36h_1_array	RNA			
SHAE005	SHAE004 HAE cells	Homo sapiens	SHAE004_H1N1_24h_2_Cells	Cells	SHAE004-R	SHAE004_H1N1_36h_2_array	RNA			
SHAE005	SHAE004 HAE cells	Homo sapiens	SHAE004_H1N1_24h_3_Cells	Cells	SHAE004-R	SHAE004_H1N1_36h_3_array	RNA			
SHAE005	SHAE004 HAE cells	Homo sapiens	SHAE004_H1N1_36h_1_Cells	Cells	SHAE004-R	SHAE004_H1N1_48h_1_array	RNA			
SHAE005	SHAE004 HAE cells	Homo sapiens	SHAE004_H1N1_36h_2_Cells	Cells	SHAE004-R	SHAE004_H1N1_48h_2_array	RNA			
SHAE005	SHAE004 HAE cells	Homo sapiens	SHAE004_H1N1_36h_3_Cells	Cells	SHAE004-R	SHAE004_H1N1_48h_3_array	RNA			
SHAE005	SHAE004 HAE cells	Homo sapiens	SHAE004_H1N1_48h_1_Cells	Cells	SHAE004-R	SHAE004_H1N1_6h_1_array	RNA			
SHAE005	SHAE004 HAE cells	Homo sapiens	SHAE004_H1N1_48h_2_Cells	Cells	SHAE004-R	SHAE004_H1N1_6h_2_array	RNA			
SHAE005	SHAE004 HAE cells	Homo sapiens	SHAE004_H1N1_48h_3_Cells	Cells	SHAE004-R	SHAE004_H1N1_6h_3_array	RNA			
SHAE005	SHAE004 HAE cells	Homo sapiens	SHAE004_Mock_0h_1_Cells	Cells	SHAE004-R	SHAE004_Mock_0h_1_array	RNA			
SHAE005	SHAE004 HAE cells	Homo sapiens	SHAE004_Mock_0h_2_Cells	Cells	SHAE004-R	SHAE004_Mock_0h_2_array	RNA			
SHAE005	SHAE004 HAE cells	Homo sapiens	SHAE004_Mock_0h_3_Cells	Cells	SHAE004-R	SHAE004_Mock_0h_3_array	RNA			
SHAE005	SHAE004 HAE cells	Homo sapiens	SHAE004_Mock_6h_1_Cells	Cells	SHAE004-R	SHAE004_Mock_6h_1_array	RNA			
SHAE005	SHAE004 HAE cells	Homo sapiens	SHAE004_Mock_6h_2_Cells	Cells	SHAE004-R	SHAE004_Mock_6h_2_array	RNA			
SHAE005	SHAE004 HAE cells	Homo sapiens	SHAE004_Mock_6h_3_Cells	Cells	SHAE004-R	SHAE004_Mock_6h_3_array	RNA			
SHAE005	SHAE004 HAE cells	Homo sapiens	SHAE004_Mock_12h_1_Cells	Cells	SHAE004-R	SHAE004_Mock_12h_1_array	RNA			
SHAE005	SHAE004 HAE cells	Homo sapiens	SHAE004_Mock_12h_2_Cells	Cells	SHAE004-R	SHAE004_Mock_12h_2_array	RNA			
SHAE005	SHAE004 HAE cells	Homo sapiens	SHAE004_Mock_12h_3_Cells	Cells	SHAE004-R	SHAE004_Mock_12h_3_array	RNA			
SHAE005	SHAE004 HAE cells	Homo sapiens	SHAE004_Mock_18h_1_Cells	Cells	SHAE004-R	SHAE004_Mock_18h_1_array	RNA			
SHAE005	SHAE004 HAE cells	Homo sapiens	SHAE004_Mock_18h_2_Cells	Cells	SHAE004-R	SHAE004_Mock_18h_2_array	RNA			
SM001	SM001_Mock_D1_1	Mus musculus	SM001_Mock_D1_1_lung	Cells	SM001-R	SM001_Mock_D1_1_RNA_ExpSam	RNA	SM001-P	SM001_Mock_1d_1_proteomics	Protein
SM001	SM001_Mock_D1_2	Mus musculus	SM001_Mock_D1_2_lung	Cells	SM001-R	SM001_Mock_D1_2_RNA_ExpSam	RNA	SM001-P	SM001_Mock_1d_2_proteomics	Protein
SM001	SM001_Mock_D1_3	Mus musculus	SM001_Mock_D1_3_lung	Cells	SM001-R	SM001_Mock_D1_3_RNA_ExpSam	RNA	SM001-P	SM001_Mock_1d_3_proteomics	Protein
SM001	SM001_Mock_D2_1	Mus musculus	SM001_Mock_D2_1_lung	Cells	SM001-R	SM001_Mock_D2_1_RNA_ExpSam	RNA	SM001-P	SM001_Mock_2d_1_proteomics	Protein
SM001	SM001_Mock_D2_2	Mus musculus	SM001_Mock_D2_2_lung	Cells	SM001-R	SM001_Mock_D2_2_RNA_ExpSam	RNA	SM001-P	SM001_Mock_2d_2_proteomics	Protein
SM001	SM001_Mock_D2_3	Mus musculus	SM001_Mock_D2_3_lung	Cells	SM001-R	SM001_Mock_D2_3_RNA_ExpSam	RNA	SM001-P	SM001_Mock_2d_3_proteomics	Protein
SM001	SM001_Mock_D4_1	Mus musculus	SM001_Mock_D4_1_lung	Cells	SM001-R	SM001_Mock_D4_1_RNA_ExpSam	RNA	SM001-P	SM001_Mock_4d_1_proteomics	Protein
SM001	SM001_Mock_D4_2	Mus musculus	SM001_Mock_D4_2_lung	Cells	SM001-R	SM001_Mock_D4_2_RNA_ExpSam	RNA	SM001-P	SM001_Mock_4d_2_proteomics	Protein
SM001	SM001_Mock_D4_3	Mus musculus	SM001_Mock_D4_3_lung	Cells	SM001-R	SM001_Mock_D4_3_RNA_ExpSam	RNA	SM001-P	SM001_Mock_4d_3_proteomics	Protein
SM001	SM001_Mock_D7_1	Mus musculus	SM001_Mock_D7_1_lung	Cells	SM001-R	SM001_Mock_D7_1_RNA_ExpSam	RNA	SM001-P	SM001_Mock_7d_1_proteomics	Protein
SM001	SM001_Mock_D7_2	Mus musculus	SM001_Mock_D7_2_lung	Cells	SM001-R	SM001_Mock_D7_2_RNA_ExpSam	RNA	SM001-P	SM001_Mock_7d_2_proteomics	Protein
SM001	SM001_Mock_D7_3	Mus musculus	SM001_Mock_D7_3_lung	Cells	SM001-R	SM001_Mock_D7_3_RNA_ExpSam	RNA	SM001-P	SM001_Mock_7d_3_proteomics	Protein
SM001	SM001_SARS_10^2pfu_D1_1	Mus musculus	SM001_SARS_10^2pfu_D1_1_lung	Cells	SM001-R	SM001_SARS_10^2pfu_D1_1_RNA_ExpSam	RNA	SM001-P	SM001_SARS_10^2_1d_1_proteomics	Protein
SM001	SM001_SARS_10^2pfu_D1_2	Mus musculus	SM001_SARS_10^2pfu_D1_2_lung	Cells	SM001-R	SM001_SARS_10^2pfu_D1_2_RNA_ExpSam	RNA	SM001-P	SM001_SARS_10^2_1d_2_proteomics	Protein
SM001	SM001_SARS_10^2pfu_D1_3	Mus musculus	SM001_SARS_10^2pfu_D1_3_lung	Cells	SM001-R	SM001_SARS_10^2pfu_D1_3_RNA_ExpSam	RNA	SM001-P	SM001_SARS_10^2_1d_3_proteomics	Protein
SM001	SM001_SARS_10^2pfu_D1_4	Mus musculus	SM001_SARS_10^2pfu_D1_4_lung	Cells	SM001-R	SM001_SARS_10^2pfu_D1_4_RNA_ExpSam	RNA	SM001-P	SM001_SARS_10^2_1d_4_proteomics	Protein
SM001	SM001_SARS_10^2pfu_D1_5	Mus musculus	SM001_SARS_10^2pfu_D1_5_lung	Cells	SM001-R	SM001_SARS_10^2pfu_D1_5_RNA_ExpSam	RNA	SM001-P	SM001_SARS_10^2_1d_5_proteomics	Protein
SM001	SM001_SARS_10^2pfu_D2_1	Mus musculus	SM001_SARS_10^2pfu_D2_1_lung	Cells	SM001-R	SM001_SARS_10^2pfu_D2_1_RNA_ExpSam	RNA	SM001-P	SM001_SARS_10^2_2d_1_proteomics	Protein
SM001	SM001_SARS_10^2pfu_D2_2	Mus musculus	SM001_SARS_10^2pfu_D2_2_lung	Cells	SM001-R	SM001_SARS_10^2pfu_D2_2_RNA_ExpSam	RNA	SM001-P	SM001_SARS_10^2_2d_2_proteomics	Protein
SM001	SM001_SARS_10^2pfu_D2_3	Mus musculus	SM001_SARS_10^2pfu_D2_3_lung	Cells	SM001-R	SM001_SARS_10^2pfu_D2_3_RNA_ExpSam	RNA	SM001-P	SM001_SARS_10^2_2d_3_proteomics	Protein
SM001	SM001_SARS_10^2pfu_D2_4	Mus musculus	SM001_SARS_10^2pfu_D2_4_lung	Cells	SM001-R	SM001_SARS_10^2pfu_D2_4_RNA_ExpSam	RNA	SM001-P	SM001_SARS_10^2_2d_4_proteomics	Protein
SM001	SM001_SARS_10^2pfu_D2_5	Mus musculus	SM001_SARS_10^2pfu_D2_5_lung	Cells	SM001-R	SM001_SARS_10^2pfu_D2_5_RNA_ExpSam	RNA	SM001-P	SM001_SARS_10^2_2d_5_proteomics	Protein
SM001	SM001_SARS_10^2pfu_D4_1	Mus musculus	SM001_SARS_10^2pfu_D4_1_lung	Cells	SM001-R	SM001_SARS_10^2pfu_D4_1_RNA_ExpSam	RNA	SM001-P	SM001_SARS_10^2_4d_1_proteomics	Protein
SM001	SM001_SARS_10^2pfu_D4_2	Mus musculus	SM001_SARS_10^2pfu_D4_2_lung	Cells	SM001-R	SM001_SARS_10^2pfu_D4_2_RNA_ExpSam	RNA	SM001-P	SM001_SARS_10^2_4d_2_proteomics	Protein
SM001	SM001_SARS_10^2pfu_D4_3	Mus musculus	SM001_SARS_10^2pfu_D4_3_lung	Cells	SM001-R	SM001_SARS_10^2pfu_D4_3_RNA_ExpSam	RNA	SM001-P	SM001_SARS_10^2_4d_3_proteomics	Protein
SM001	SM001_SARS_10^2pfu_D4_4	Mus musculus	SM001_SARS_10^2pfu_D4_4_lung	Cells	SM001-R	SM001_SARS_10^2pfu_D4_4_RNA_ExpSam	RNA	SM001-P	SM001_SARS_10^2_4d_4_proteomics	Protein
SM001	SM001_SARS_10^2pfu_D4_5	Mus musculus	SM001_SARS_10^2pfu_D4_5_lung	Cells	SM001-R	SM001_SARS_10^2pfu_D4_5_RNA_ExpSam	RNA	SM001-P	SM001_SARS_10^2_4d_5_proteomics	Protein
SM001	SM001_SARS_10^2pfu_D7_1	Mus musculus	SM001_SARS_10^2pfu_D7_1_lung	Cells	SM001-R	SM001_SARS_10^2pfu_D7_1_RNA_ExpSam	RNA	SM001-P	SM001_SARS_10^2_7d_1_proteomics	Protein
SM001	SM001_SARS_10^2pfu_D7_2	Mus musculus	SM001_SARS_10^2pfu_D7_2_lung	Cells	SM001-R	SM001_SARS_10^2pfu_D7_2_RNA_ExpSam	RNA	SM001-P	SM001_SARS_10^2_7d_2_proteomics	Protein
SM001	SM001_SARS_10^2pfu_D7_3	Mus musculus	SM001_SARS_10^2pfu_D7_3_lung	Cells	SM001-R	SM001_SARS_10^2pfu_D7_3_RNA_ExpSam	RNA	SM001-P	SM001_SARS_10^2_7d_3_proteomics	Protein
SM001	SM001_SARS_10^2pfu_D7_4	Mus musculus	SM001_SARS_10^2pfu_D7_4_lung	Cells	SM001-R	SM001_SARS_10^2pfu_D7_4_RNA_ExpSam	RNA	SM001-P	SM001_SARS_10^2_7d_4_proteomics	Protein
SM001	SM001_SARS_10^2pfu_D7_5	Mus musculus	SM001_SARS_10^2pfu_D7_5_lung	Cells	SM001-R	SM001_SARS_10^2pfu_D7_5_RNA_ExpSam	RNA	SM001-P	SM001_SARS_10^2_7d_5_proteomics	Protein
SM001	SM001_SARS_10^3pfu_D1_1	Mus musculus	SM001_SARS_10^3pfu_D1_1_lung	Cells	SM001-R	SM001_SARS_10^3pfu_D1_1_RNA_ExpSam	RNA	SM001-P	SM001_SARS_10^3_1d_1_proteomics	Protein
SM001	SM001_SARS_10^3pfu_D1_2	Mus musculus	SM001_SARS_10^3pfu_D1_2_lung	Cells	SM001-R	SM001_SARS_10^3pfu_D1_2_RNA_ExpSam	RNA	SM001-P	SM001_SARS_10^3_1d_2_proteomics	Protein
SM001	SM001_SARS_10^3pfu_D1_3	Mus musculus	SM001_SARS_10^3pfu_D1_3_lung	Cells	SM001-R	SM001_SARS_10^3pfu_D1_3_RNA_ExpSam	RNA	SM001-P	SM001_SARS_10^3_1d_3_proteomics	Protein
SM001	SM001_SARS_10^3pfu_D1_4	Mus musculus	SM001_SARS_10^3pfu_D1_4_lung	Cells	SM001-R	SM001_SARS_10^3pfu_D1_4_RNA_ExpSam	RNA	SM001-P	SM001_SARS_10^3_1d_4_proteomics	Protein
SM001	SM001_SARS_10^3pfu_D1_5	Mus musculus	SM001_SARS_10^3pfu_D1_5_lung	Cells	SM001-R	SM001_SARS_10^3pfu_D1_5_RNA_ExpSam	RNA	SM001-P	SM001_SARS_10^3_1d_5_proteomics	Protein
SM001	SM001_SARS_10^3pfu_D2_1	Mus musculus	SM001_SARS_10^3pfu_D2_1_lung	Cells	SM001-R	SM001_SARS_10^3pfu_D2_1_RNA_ExpSam	RNA	SM001-P	SM001_SARS_10^3_2d_1_proteomics	Protein
SM001	SM001_SARS_10^3pfu_D2_2	Mus musculus	SM001_SARS_10^3pfu_D2_2_lung	Cells	SM001-R	SM001_SARS_10^3pfu_D2_2_RNA_ExpSam	RNA	SM001-P	SM001_SARS_10^3_2d_2_proteomics	Protein
SM001	SM001_SARS_10^3pfu_D2_3	Mus musculus	SM001_SARS_10^3pfu_D2_3_lung	Cells	SM001-R	SM001_SARS_10^3pfu_D2_3_RNA_ExpSam	RNA	SM001-P	SM001_SARS_10^3_2d_3_proteomics	Protein
SM001	SM001_SARS_10^3pfu_D2_4	Mus musculus	SM001_SARS_10^3pfu_D2_4_lung	Cells	SM001-R	SM001_SARS_10^3pfu_D2_4_RNA_ExpSam	RNA	SM001-P	SM001_SARS_10^3_2d_4_proteomics	Protein
SM001	SM001_SARS_10^3pfu_D2_5	Mus musculus	SM001_SARS_10^3pfu_D2_5_lung	Cells	SM001-R	SM001_SARS_10^3pfu_D2_5_RNA_ExpSam	RNA	SM001-P	SM001_SARS_10^3_2d_5_proteomics	Protein
SM001	SM001_SARS_10^3pfu_D4_1	Mus musculus	SM001_SARS_10^3pfu_D4_1_lung	Cells	SM001-R	SM001_SARS_10^3pfu_D4_1_RNA_ExpSam	RNA	SM001-P	SM001_SARS_10^3_4d_1_proteomics	Protein
SM001	SM001_SARS_10^3pfu_D4_2	Mus musculus	SM001_SARS_10^3pfu_D4_2_lung	Cells	SM001-R	SM001_SARS_10^3pfu_D4_2_RNA_ExpSam	RNA	SM001-P	SM001_SARS_10^3_4d_2_proteomics	Protein
SM001	SM001_SARS_10^3pfu_D4_3	Mus musculus	SM001_SARS_10^3pfu_D4_3_lung	Cells	SM001-R	SM001_SARS_10^3pfu_D4_3_RNA_ExpSam	RNA	SM001-P	SM001_SARS_10^3_4d_3_proteomics	Protein
SM001	SM001_SARS_10^3pfu_D4_4	Mus musculus	SM001_SARS_10^3pfu_D4_4_lung	Cells	SM001-R	SM001_SARS_10^3pfu_D4_4_RNA_ExpSam	RNA	SM001-P	SM001_SARS_10^3_4d_4_proteomics	Protein
SM001	SM001_SARS_10^3pfu_D4_5	Mus musculus	SM001_SARS_10^3pfu_D4_5_lung	Cells	SM001-R	SM001_SARS_10^3pfu_D4_5_RNA_ExpSam	RNA	SM001-P	SM001_SARS_10^3_4d_5_proteomics	Protein
SM001	SM001_SARS_10^3pfu_D7_1	Mus musculus	SM001_SARS_10^3pfu_D7_1_lung	Cells	SM001-R	SM001_SARS_10^3pfu_D7_1_RNA_ExpSam	RNA	SM001-P	SM001_SARS_10^3_7d_1_proteomics	Protein
SM001	SM001_SARS_10^3pfu_D7_2	Mus musculus	SM001_SARS_10^3pfu_D7_2_lung	Cells	SM001-R	SM001_SARS_10^3pfu_D7_2_RNA_ExpSam	RNA	SM001-P	SM001_SARS_10^3_7d_2_proteomics	Protein
SM001	SM001_SARS_10^3pfu_D7_3	Mus musculus	SM001_SARS_10^3pfu_D7_3_lung	Cells	SM001-R	SM001_SARS_10^3pfu_D7_3_RNA_ExpSam	RNA	SM001-P	SM001_SARS_10^3_7d_3_proteomics	Protein
SM001	SM001_SARS_10^3pfu_D7_4	Mus musculus	SM001_SARS_10^3pfu_D7_4_lung	Cells	SM001-R	SM001_SARS_10^3pfu_D7_4_RNA_ExpSam	RNA	SM001-P	SM001_SARS_10^3_7d_4_proteomics	Protein
SM001	SM001_SARS_10^3pfu_D7_5	Mus musculus	SM001_SARS_10^3pfu_D7_5_lung	Cells	SM001-R	SM001_SARS_10^3pfu_D7_5_RNA_ExpSam	RNA	SM001-P	SM001_SARS_10^3_7d_5_proteomics	Protein
SM001	SM001_SARS_10^4pfu_D1_1	Mus musculus	SM001_SARS_10^4pfu_D1_1_lung	Cells	SM001-R	SM001_SARS_10^4pfu_D1_1_RNA_ExpSam	RNA	SM001-P	SM001_SARS_10^4_1d_1_proteomics	Protein
SM001	SM001_SARS_10^4pfu_D1_2	Mus musculus	SM001_SARS_10^4pfu_D1_2_lung	Cells	SM001-R	SM001_SARS_10^4pfu_D1_2_RNA_ExpSam	RNA	SM001-P	SM001_SARS_10^4_1d_2_proteomics	Protein
SM001	SM001_SARS_10^4pfu_D1_3	Mus musculus	SM001_SARS_10^4pfu_D1_3_lung	Cells	SM001-R	SM001_SARS_10^4pfu_D1_3_RNA_ExpSam	RNA	SM001-P	SM001_SARS_10^4_1d_3_proteomics	Protein
SM001	SM001_SARS_10^4pfu_D1_4	Mus musculus	SM001_SARS_10^4pfu_D1_4_lung	Cells	SM001-R	SM001_SARS_10^4pfu_D1_4_RNA_ExpSam	RNA	SM001-P	SM001_SARS_10^4_1d_4_proteomics	Protein
SM001	SM001_SARS_10^4pfu_D1_5	Mus musculus	SM001_SARS_10^4pfu_D1_5_lung	Cells	SM001-R	SM001_SARS_10^4pfu_D1_5_RNA_ExpSam	RNA	SM001-P	SM001_SARS_10^4_1d_5_proteomics	Protein
SM001	SM001_SARS_10^4pfu_D2_1	Mus musculus	SM001_SARS_10^4pfu_D2_1_lung	Cells	SM001-R	SM001_SARS_10^4pfu_D2_1_RNA_ExpSam	RNA	SM001-P	SM001_SARS_10^4_2d_1_proteomics	Protein
SM001	SM001_SARS_10^4pfu_D2_2	Mus musculus	SM001_SARS_10^4pfu_D2_2_lung	Cells	SM001-R	SM001_SARS_10^4pfu_D2_2_RNA_ExpSam	RNA	SM001-P	SM001_SARS_10^4_2d_2_proteomics	Protein
SM001	SM001_SARS_10^4pfu_D2_3	Mus musculus	SM001_SARS_10^4pfu_D2_3_lung	Cells	SM001-R	SM001_SARS_10^4pfu_D2_3_RNA_ExpSam	RNA	SM001-P	SM001_SARS_10^4_2d_3_proteomics	Protein
SM001	SM001_SARS_10^4pfu_D2_4	Mus musculus	SM001_SARS_10^4pfu_D2_4_lung	Cells	SM001-R	SM001_SARS_10^4pfu_D2_4_RNA_ExpSam	RNA	SM001-P	SM001_SARS_10^4_2d_4_proteomics	Protein
SM001	SM001_SARS_10^4pfu_D2_5	Mus musculus	SM001_SARS_10^4pfu_D2_5_lung	Cells	SM001-R	SM001_SARS_10^4pfu_D2_5_RNA_ExpSam	RNA	SM001-P	SM001_SARS_10^4_2d_5_proteomics	Protein
SM001	SM001_SARS_10^4pfu_D4_1	Mus musculus	SM001_SARS_10^4pfu_D4_1_lung	Cells	SM001-R	SM001_SARS_10^4pfu_D4_1_RNA_ExpSam	RNA	SM001-P	SM001_SARS_10^4_4d_1_proteomics	Protein
SM001	SM001_SARS_10^4pfu_D4_2	Mus musculus	SM001_SARS_10^4pfu_D4_2_lung	Cells	SM001-R	SM001_SARS_10^4pfu_D4_2_RNA_ExpSam	RNA	SM001-P	SM001_SARS_10^4_4d_2_proteomics	Protein
SM001	SM001_SARS_10^4pfu_D4_3	Mus musculus	SM001_SARS_10^4pfu_D4_3_lung	Cells	SM001-R	SM001_SARS_10^4pfu_D4_3_RNA_ExpSam	RNA	SM001-P	SM001_SARS_10^4_4d_3_proteomics	Protein
SM001	SM001_SARS_10^4pfu_D4_4	Mus musculus	SM001_SARS_10^4pfu_D4_4_lung	Cells	SM001-R	SM001_SARS_10^4pfu_D4_4_RNA_ExpSam	RNA	SM001-P	SM001_SARS_10^4_4d_4_proteomics	Protein
SM001	SM001_SARS_10^4pfu_D4_5	Mus musculus	SM001_SARS_10^4pfu_D4_5_lung	Cells	SM001-R	SM001_SARS_10^4pfu_D4_5_RNA_ExpSam	RNA	SM001-P	SM001_SARS_10^4_4d_5_proteomics	Protein
SM001	SM001_SARS_10^4pfu_D7_1	Mus musculus	SM001_SARS_10^4pfu_D7_1_lung	Cells	SM001-R	SM001_SARS_10^4pfu_D7_1_RNA_ExpSam	RNA	SM001-P	SM001_SARS_10^4_7d_1_proteomics	Protein
SM001	SM001_SARS_10^4pfu_D7_2	Mus musculus	SM001_SARS_10^4pfu_D7_2_lung	Cells	SM001-R	SM001_SARS_10^4pfu_D7_2_RNA_ExpSam	RNA	SM001-P	SM001_SARS_10^4_7d_2_proteomics	Protein
SM001	SM001_SARS_10^4pfu_D7_3	Mus musculus	SM001_SARS_10^4pfu_D7_3_lung	Tissue	SM001-R	SM001_SARS_10^4pfu_D7_3_RNA_ExpSam	RNA	SM001-P	SM001_SARS_10^4_7d_3_proteomics	Protein
SM001	SM001_SARS_10^4pfu_D7_4	Mus musculus	SM001_SARS_10^4pfu_D7_4_lung	Tissue	SM001-R	SM001_SARS_10^4pfu_D7_4_RNA_ExpSam	RNA	SM001-P	SM001_SARS_10^4_7d_4_proteomics	Protein
SM001	SM001_SARS_10^4pfu_D7_5	Mus musculus	SM001_SARS_10^4pfu_D7_5_lung	Tissue	SM001-R	SM001_SARS_10^4pfu_D7_5_RNA_ExpSam	RNA	SM001-P	SM001_SARS_10^4_7d_5_proteomics	Protein
SM001	SM001_SARS_10^5pfu_D1_1	Mus musculus	SM001_SARS_10^5pfu_D1_1_lung	Tissue	SM001-R	SM001_SARS_10^5pfu_D1_1_RNA_ExpSam	RNA	SM001-P	SM001_SARS_10^5_1d_1_proteomics	Protein
SM001	SM001_SARS_10^5pfu_D1_2	Mus musculus	SM001_SARS_10^5pfu_D1_2_lung	Tissue	SM001-R	SM001_SARS_10^5pfu_D1_2_RNA_ExpSam	RNA	SM001-P	SM001_SARS_10^5_1d_2_proteomics	Protein
SM001	SM001_SARS_10^5pfu_D1_3	Mus musculus	SM001_SARS_10^5pfu_D1_3_lung	Tissue	SM001-R	SM001_SARS_10^5pfu_D1_3_RNA_ExpSam	RNA	SM001-P	SM001_SARS_10^5_1d_3_proteomics	Protein
SM001	SM001_SARS_10^5pfu_D1_4	Mus musculus	SM001_SARS_10^5pfu_D1_4_lung	Tissue	SM001-R	SM001_SARS_10^5pfu_D1_4_RNA_ExpSam	RNA	SM001-P	SM001_SARS_10^5_1d_4_proteomics	Protein
SM001	SM001_SARS_10^5pfu_D1_5	Mus musculus	SM001_SARS_10^5pfu_D1_5_lung	Tissue	SM001-R	SM001_SARS_10^5pfu_D1_5_RNA_ExpSam	RNA	SM001-P	SM001_SARS_10^5_1d_5_proteomics	Protein
SM001	SM001_SARS_10^5pfu_D2_1	Mus musculus	SM001_SARS_10^5pfu_D2_1_lung	Tissue	SM001-R	SM001_SARS_10^5pfu_D2_1_RNA_ExpSam	RNA	SM001-P	SM001_SARS_10^5_2d_1_proteomics	Protein
SM001	SM001_SARS_10^5pfu_D2_2	Mus musculus	SM001_SARS_10^5pfu_D2_2_lung	Tissue	SM001-R	SM001_SARS_10^5pfu_D2_2_RNA_ExpSam	RNA	SM001-P	SM001_SARS_10^5_2d_2_proteomics	Protein
SM001	SM001_SARS_10^5pfu_D2_3	Mus musculus	SM001_SARS_10^5pfu_D2_3_lung	Tissue	SM001-R	SM001_SARS_10^5pfu_D2_3_RNA_ExpSam	RNA	SM001-P	SM001_SARS_10^5_2d_3_proteomics	Protein
SM001	SM001_SARS_10^5pfu_D2_4	Mus musculus	SM001_SARS_10^5pfu_D2_4_lung	Tissue	SM001-R	SM001_SARS_10^5pfu_D2_4_RNA_ExpSam	RNA	SM001-P	SM001_SARS_10^5_2d_4_proteomics	Protein
SM001	SM001_SARS_10^5pfu_D2_5	Mus musculus	SM001_SARS_10^5pfu_D2_5_lung	Tissue	SM001-R	SM001_SARS_10^5pfu_D2_5_RNA_ExpSam	RNA	SM001-P	SM001_SARS_10^5_2d_5_proteomics	Protein
SM001	SM001_SARS_10^5pfu_D4_1	Mus musculus	SM001_SARS_10^5pfu_D4_1_lung	Tissue	SM001-R	SM001_SARS_10^5pfu_D4_1_RNA_ExpSam	RNA	SM001-P	SM001_SARS_10^5_4d_1_proteomics	Protein
SM001	SM001_SARS_10^5pfu_D4_2	Mus musculus	SM001_SARS_10^5pfu_D4_2_lung	Tissue	SM001-R	SM001_SARS_10^5pfu_D4_2_RNA_ExpSam	RNA	SM001-P	SM001_SARS_10^5_4d_2_proteomics	Protein
SM001	SM001_SARS_10^5pfu_D4_3	Mus musculus	SM001_SARS_10^5pfu_D4_3_lung	Tissue	SM001-R	SM001_SARS_10^5pfu_D4_3_RNA_ExpSam	RNA	SM001-P	SM001_SARS_10^5_4d_3_proteomics	Protein
SM001	SM001_SARS_10^5pfu_D4_4	Mus musculus	SM001_SARS_10^5pfu_D4_4_lung	Tissue	SM001-R	SM001_SARS_10^5pfu_D4_4_RNA_ExpSam	RNA	SM001-P	SM001_SARS_10^5_4d_4_proteomics	Protein
SM001	SM001_SARS_10^5pfu_D4_5	Mus musculus	SM001_SARS_10^5pfu_D4_5_lung	Tissue	SM001-R	SM001_SARS_10^5pfu_D4_5_RNA_ExpSam	RNA	SM001-P	SM001_SARS_10^5_4d_5_proteomics	Protein
SM001	SM001_SARS_10^5pfu_D7_1	Mus musculus	SM001_SARS_10^5pfu_D7_1_lung	Tissue	SM001-R	SM001_SARS_10^5pfu_D7_1_RNA_ExpSam	RNA	SM001-P	SM001_SARS_10^5_7d_1_proteomics	Protein
SM001	SM001_SARS_10^5pfu_D7_2	Mus musculus	SM001_SARS_10^5pfu_D7_2_lung	Tissue	SM001-R	SM001_SARS_10^5pfu_D7_2_RNA_ExpSam	RNA	SM001-P	SM001_SARS_10^5_7d_2_proteomics	Protein
SM001	SM001_SARS_10^5pfu_D7_3	Mus musculus	SM001_SARS_10^5pfu_D7_3_lung	Tissue	SM001-R	SM001_SARS_10^5pfu_D7_3_RNA_ExpSam	RNA	SM001-P	SM001_SARS_10^5_7d_3_proteomics	Protein
SM001	SM001_SARS_10^5pfu_D7_4	Mus musculus	SM001_SARS_10^5pfu_D7_4_lung	Tissue	SM001-R	SM001_SARS_10^5pfu_D7_4_RNA_ExpSam	RNA	SM001-P	SM001_SARS_10^5_7d_4_proteomics	Protein
SM001	SM001_SARS_10^5pfu_D7_5	Mus musculus	SM001_SARS_10^5pfu_D7_5_lung	Tissue	SM001-R	SM001_SARS_10^5pfu_D7_5_RNA_ExpSam	RNA	SM001-P	SM001_SARS_10^5_7d_5_proteomics	Protein
SM003	SM003_10^4_MA15_d1_1	Mus musculus	SM003_10^4_MA15_d1_1_Lung	Tissue	SM003-R	SM003_10^4_MA15_d1_1_array	RNA			
SM003	SM003_10^4_MA15_d1_2	Mus musculus	SM003_10^4_MA15_d1_2_Lung	Tissue	SM003-R	SM003_10^4_MA15_d1_2_array	RNA			
SM003	SM003_10^4_MA15_d1_3	Mus musculus	SM003_10^4_MA15_d1_3_Lung	Tissue	SM003-R	SM003_10^4_MA15_d1_3_array	RNA			
SM003	SM003_10^4_MA15_d1_4	Mus musculus	SM003_10^4_MA15_d1_4_Lung	Tissue	SM003-R	SM003_10^4_MA15_d1_4_array	RNA			
SM003	SM003_10^4_MA15_d2_1	Mus musculus	SM003_10^4_MA15_d2_1_Lung	Tissue	SM003-R	SM003_10^4_MA15_d2_1_array	RNA			
SM003	SM003_10^4_MA15_d2_2	Mus musculus	SM003_10^4_MA15_d2_2_Lung	Tissue	SM003-R	SM003_10^4_MA15_d2_2_array	RNA			
SM003	SM003_10^4_MA15_d2_3	Mus musculus	SM003_10^4_MA15_d2_3_Lung	Tissue	SM003-R	SM003_10^4_MA15_d2_3_array	RNA			
SM003	SM003_10^4_MA15_d2_4	Mus musculus	SM003_10^4_MA15_d2_4_Lung	Tissue	SM003-R	SM003_10^4_MA15_d2_4_array	RNA			
SM003	SM003_10^4_MA15_d4_1	Mus musculus	SM003_10^4_MA15_d4_1_Lung	Tissue	SM003-R	SM003_10^4_MA15_d4_1_array	RNA			
SM003	SM003_10^4_MA15_d4_2	Mus musculus	SM003_10^4_MA15_d4_2_Lung	Tissue	SM003-R	SM003_10^4_MA15_d4_2_array	RNA			
SM003	SM003_10^4_MA15_d4_3	Mus musculus	SM003_10^4_MA15_d4_3_Lung	Tissue	SM003-R	SM003_10^4_MA15_d4_3_array	RNA			
SM003	SM003_10^4_MA15_d4_4	Mus musculus	SM003_10^4_MA15_d4_4_Lung	Tissue	SM003-R	SM003_10^4_MA15_d4_4_array	RNA			
SM003	SM003_10^4_MA15_d7_1	Mus musculus	SM003_10^4_MA15_d7_1_Lung	Tissue	SM003-R	SM003_10^4_MA15_d7_1_array	RNA			
SM003	SM003_10^4_MA15_d7_2	Mus musculus	SM003_10^4_MA15_d7_2_Lung	Tissue	SM003-R	SM003_10^4_MA15_d7_2_array	RNA			
SM003	SM003_10^4_MA15_d7_3	Mus musculus	SM003_10^4_MA15_d7_3_Lung	Tissue	SM003-R	SM003_10^4_MA15_d7_3_array	RNA			
SM003	SM003_10^4_MA15_d7_4	Mus musculus	SM003_10^4_MA15_d7_4_Lung	Tissue	SM003-R		RNA			
SM003	SM003_10^5_MA15_d1_1	Mus musculus	SM003_10^5_MA15_d1_1_Lung	Tissue	SM003-R	SM003_10^5_MA15_d1_1_array	RNA			
SM003	SM003_10^5_MA15_d1_3	Mus musculus	SM003_10^5_MA15_d1_3_Lung	Tissue	SM003-R	SM003_10^5_MA15_d1_3_array	RNA			
SM003	SM003_10^5_MA15_d1_4	Mus musculus	SM003_10^5_MA15_d1_4_Lung	Tissue	SM003-R	SM003_10^5_MA15_d1_4_array	RNA			
SM003	SM003_10^5_MA15_d2_1	Mus musculus	SM003_10^5_MA15_d2_1_Lung	Tissue	SM003-R		RNA			
SM003	SM003_10^5_MA15_d2_2	Mus musculus	SM003_10^5_MA15_d2_2_Lung	Tissue	SM003-R	SM003_10^5_MA15_d2_2_array	RNA			
SM003	SM003_10^5_MA15_d2_3	Mus musculus	SM003_10^5_MA15_d2_3_Lung	Tissue	SM003-R	SM003_10^5_MA15_d2_3_array	RNA			
SM003	SM003_10^5_MA15_d2_4	Mus musculus	SM003_10^5_MA15_d2_4_Lung	Tissue	SM003-R	SM003_10^5_MA15_d2_4_array	RNA			
SM003	SM003_10^5_MA15_d4_1	Mus musculus	SM003_10^5_MA15_d4_1_Lung	Tissue	SM003-R	SM003_10^5_MA15_d4_1_array	RNA			
SM003	SM003_10^5_MA15_d4_2	Mus musculus	SM003_10^5_MA15_d4_2_Lung	Tissue	SM003-R	SM003_10^5_MA15_d4_2_array	RNA			
SM003	SM003_10^5_MA15_d4_3	Mus musculus	SM003_10^5_MA15_d4_3_Lung	Tissue	SM003-R	SM003_10^5_MA15_d4_3_array	RNA			
SM003	SM003_10^5_MA15_d4_4	Mus musculus	SM003_10^5_MA15_d4_4_Lung	Tissue	SM003-R	SM003_10^5_MA15_d4_4_array	RNA			
SM003	SM003_10^5_MA15_d4_5	Mus musculus	SM003_10^5_MA15_d4_5_Lung	Tissue	SM003-R	SM003_10^5_MA15_d4_5_array	RNA			
SM003	SM003_10^5_MA15_d7_1	Mus musculus	SM003_10^5_MA15_d7_1_Lung	Tissue	SM003-R	SM003_10^5_MA15_d7_1_array	RNA			
SM003	SM003_10^5_MA15_d7_2	Mus musculus	SM003_10^5_MA15_d7_2_Lung	Tissue	SM003-R	SM003_10^5_MA15_d7_2_array	RNA			
SM003	SM003_10^5_MA15_d7_3	Mus musculus	SM003_10^5_MA15_d7_3_Lung	Tissue	SM003-R	SM003_10^5_MA15_d7_3_array	RNA			
SM003	SM003_BAT_d1_1	Mus musculus	SM003_BAT_d1_1_Lung	Tissue	SM003-R	SM003_BAT_d1_1_array	RNA			
SM003	SM003_BAT_d1_2	Mus musculus	SM003_BAT_d1_2_Lung	Tissue	SM003-R	SM003_BAT_d1_2_array	RNA			
SM003	SM003_BAT_d1_3	Mus musculus	SM003_BAT_d1_3_Lung	Tissue	SM003-R	SM003_BAT_d1_3_array	RNA			
SM003	SM003_BAT_d1_4	Mus musculus	SM003_BAT_d1_4_Lung	Tissue	SM003-R	SM003_BAT_d1_4_array	RNA			
SM003	SM003_BAT_d1_5	Mus musculus	SM003_BAT_d1_5_Lung	Tissue	SM003-R	SM003_BAT_d1_5_array	RNA			
SM003	SM003_BAT_d2_1	Mus musculus	SM003_BAT_d2_1_Lung	Tissue	SM003-R	SM003_BAT_d2_1_array	RNA			
SM003	SM003_BAT_d2_2	Mus musculus	SM003_BAT_d2_2_Lung	Tissue	SM003-R	SM003_BAT_d2_2_array	RNA			
SM003	SM003_BAT_d2_3	Mus musculus	SM003_BAT_d2_3_Lung	Tissue	SM003-R	SM003_BAT_d2_3_array	RNA			
SM003	SM003_BAT_d2_4	Mus musculus	SM003_BAT_d2_4_Lung	Tissue	SM003-R	SM003_BAT_d2_4_array	RNA			
SM003	SM003_BAT_d2_5	Mus musculus	SM003_BAT_d2_5_Lung	Tissue	SM003-R	SM003_BAT_d2_5_array	RNA			
SM003	SM003_BAT_d4_1	Mus musculus	SM003_BAT_d4_1_Lung	Tissue	SM003-R	SM003_BAT_d4_1_array	RNA			
SM003	SM003_BAT_d4_2	Mus musculus	SM003_BAT_d4_2_Lung	Tissue	SM003-R	SM003_BAT_d4_2_array	RNA			
SM003	SM003_BAT_d4_3	Mus musculus	SM003_BAT_d4_3_Lung	Tissue	SM003-R	SM003_BAT_d4_3_array	RNA			
SM003	SM003_BAT_d4_4	Mus musculus	SM003_BAT_d4_4_Lung	Tissue	SM003-R	SM003_BAT_d4_4_array	RNA			
SM003	SM003_BAT_d4_5	Mus musculus	SM003_BAT_d4_5_Lung	Tissue	SM003-R	SM003_BAT_d4_5_array	RNA			
SM003	SM003_BAT_d7_1	Mus musculus	SM003_BAT_d7_1_Lung	Tissue	SM003-R	SM003_BAT_d7_1_array	RNA			
SM003	SM003_BAT_d7_3	Mus musculus	SM003_BAT_d7_3_Lung	Tissue	SM003-R	SM003_BAT_d7_3_array	RNA			
SM003	SM003_BAT_d7_4	Mus musculus	SM003_BAT_d7_4_Lung	Tissue	SM003-R	SM003_BAT_d7_4_array	RNA			
SM003	SM003_BAT_d7_5	Mus musculus	SM003_BAT_d7_5_Lung	Tissue	SM003-R	SM003_BAT_d7_5_array	RNA			
SM003	SM003_Mock_d1_1	Mus musculus	SM003_Mock_d1_1_Lung	Tissue	SM003-R	SM003_Mock_d1_1_array	RNA			
SM003	SM003_Mock_d1_2	Mus musculus	SM003_Mock_d1_2_Lung	Tissue	SM003-R	SM003_Mock_d1_2_array	RNA			
SM003	SM003_Mock_d1_3	Mus musculus	SM003_Mock_d1_3_Lung	Tissue	SM003-R	SM003_Mock_d1_3_array	RNA			
SM003	SM003_Mock_d1_4	Mus musculus	SM003_Mock_d1_4_Lung	Tissue	SM003-R	SM003_Mock_d1_4_array	RNA			
SM003	SM003_Mock_d2_1	Mus musculus	SM003_Mock_d2_1_Lung	Tissue	SM003-R	SM003_Mock_d2_1_array	RNA			
SM003	SM003_Mock_d2_2	Mus musculus	SM003_Mock_d2_2_Lung	Tissue	SM003-R	SM003_Mock_d2_2_array	RNA			
SM003	SM003_Mock_d2_3	Mus musculus	SM003_Mock_d2_3_Lung	Tissue	SM003-R	SM003_Mock_d2_3_array	RNA			
SM003	SM003_Mock_d2_4	Mus musculus	SM003_Mock_d2_4_Lung	Tissue	SM003-R	SM003_Mock_d2_4_array	RNA			
SM003	SM003_Mock_d4_1	Mus musculus	SM003_Mock_d4_1_Lung	Tissue	SM003-R	SM003_Mock_d4_1_array	RNA			
SM003	SM003_Mock_d4_2	Mus musculus	SM003_Mock_d4_2_Lung	Tissue	SM003-R	SM003_Mock_d4_2_array	RNA			
SM003	SM003_Mock_d4_3	Mus musculus	SM003_Mock_d4_3_Lung	Tissue	SM003-R	SM003_Mock_d4_3_array	RNA			
SM003	SM003_Mock_d4_4	Mus musculus	SM003_Mock_d4_4_Lung	Tissue	SM003-R	SM003_Mock_d4_4_array	RNA			
SM003	SM003_Mock_d7_1	Mus musculus	SM003_Mock_d7_1_Lung	Tissue	SM003-R	SM003_Mock_d7_1_array	RNA			
SM003	SM003_Mock_d7_2	Mus musculus	SM003_Mock_d7_2_Lung	Tissue	SM003-R	SM003_Mock_d7_2_array	RNA			
SM003	SM003_Mock_d7_3	Mus musculus	SM003_Mock_d7_3_Lung	Tissue	SM003-R	SM003_Mock_d7_3_array	RNA			
SM003	SM003_Mock_d7_4	Mus musculus	SM003_Mock_d7_4_Lung	Tissue	SM003-R	SM003_Mock_d7_4_array	RNA			
SM003	SM003_SARS_d1_1	Mus musculus	SM003_SARS_d1_1_Lung	Tissue	SM003-R	SM003_SARS_d1_1_array	RNA			
SM003	SM003_SARS_d1_2	Mus musculus	SM003_SARS_d1_2_Lung	Tissue	SM003-R	SM003_SARS_d1_2_array	RNA			
SM003	SM003_SARS_d1_3	Mus musculus	SM003_SARS_d1_3_Lung	Tissue	SM003-R	SM003_SARS_d1_3_array	RNA			
SM003	SM003_SARS_d1_4	Mus musculus	SM003_SARS_d1_4_Lung	Tissue	SM003-R	SM003_SARS_d1_4_array	RNA			
SM003	SM003_SARS_d2_1	Mus musculus	SM003_SARS_d2_1_Lung	Tissue	SM003-R	SM003_SARS_d2_1_array	RNA			
SM003	SM003_SARS_d2_2	Mus musculus	SM003_SARS_d2_2_Lung	Tissue	SM003-R	SM003_SARS_d2_2_array	RNA			
SM003	SM003_SARS_d2_3	Mus musculus	SM003_SARS_d2_3_Lung	Tissue	SM003-R	SM003_SARS_d2_3_array	RNA			
SM003	SM003_SARS_d2_4	Mus musculus	SM003_SARS_d2_4_Lung	Tissue	SM003-R	SM003_SARS_d2_4_array	RNA			
SM003	SM003_SARS_d2_5	Mus musculus	SM003_SARS_d2_5_Lung	Tissue	SM003-R	SM003_SARS_d2_5_array	RNA			
SM003	SM003_SARS_d4_1	Mus musculus	SM003_SARS_d4_1_Lung	Tissue	SM003-R	SM003_SARS_d4_1_array	RNA			
SM003	SM003_SARS_d4_2	Mus musculus	SM003_SARS_d4_2_Lung	Tissue	SM003-R	SM003_SARS_d4_2_array	RNA			
SM003	SM003_SARS_d4_3	Mus musculus	SM003_SARS_d4_3_Lung	Tissue	SM003-R	SM003_SARS_d4_3_array	RNA			
SM003	SM003_SARS_d4_4	Mus musculus	SM003_SARS_d4_4_Lung	Tissue	SM003-R	SM003_SARS_d4_4_array	RNA			
SM003	SM003_SARS_d4_5	Mus musculus	SM003_SARS_d4_5_Lung	Tissue	SM003-R	SM003_SARS_d4_5_array	RNA			
SM003	SM003_SARS_d7_1	Mus musculus	SM003_SARS_d7_1_Lung	Tissue	SM003-R	SM003_SARS_d7_1_array	RNA			
SM003	SM003_SARS_d7_2	Mus musculus	SM003_SARS_d7_2_Lung	Tissue	SM003-R	SM003_SARS_d7_2_array	RNA			
SM003	SM003_SARS_d7_3	Mus musculus	SM003_SARS_d7_3_Lung	Tissue	SM003-R	SM003_SARS_d7_3_array	RNA			
SM003	SM003_SARS_d7_4	Mus musculus	SM003_SARS_d7_4_Lung	Tissue	SM003-R	SM003_SARS_d7_4_array	RNA			
SM003	SM003_SARS_d7_5	Mus musculus	SM003_SARS_d7_5_Lung	Tissue	SM003-R	SM003_SARS_d7_5_array	RNA			
SM004	SM004_B6_MA15_d4_1	Mus musculus	SM004_B6_MA15_d4_1_Lung	Tissue	SM004-R	SM004_B6_MA15_d4_1_array	RNA			
SM004	SM004_B6_MA15_d4_2	Mus musculus	SM004_B6_MA15_d4_2_Lung	Tissue	SM004-R	SM004_B6_MA15_d4_2_array	RNA			
SM004	SM004_B6_MA15_d4_3	Mus musculus	SM004_B6_MA15_d4_3_Lung	Tissue	SM004-R	SM004_B6_MA15_d4_3_array	RNA			
SM004	SM004_B6_MA15_d4_4	Mus musculus	SM004_B6_MA15_d4_4_Lung	Tissue	SM004-R	SM004_B6_MA15_d4_4_array	RNA			
SM004	SM004_B6_MA15_d7_1	Mus musculus	SM004_B6_MA15_d7_1_Lung	Tissue	SM004-R	SM004_B6_MA15_d7_1_array	RNA			
SM004	SM004_B6_MA15_d7_3	Mus musculus	SM004_B6_MA15_d7_3_Lung	Tissue	SM004-R	SM004_B6_MA15_d7_3_array	RNA			
SM004	SM004_B6_MA15_d7_4	Mus musculus	SM004_B6_MA15_d7_4_Lung	Tissue	SM004-R	SM004_B6_MA15_d7_4_array	RNA			
SM004	SM004_B6_Mock_d4_1	Mus musculus	SM004_B6_Mock_d4_1_Lung	Tissue	SM004-R	SM004_B6_Mock_d4_1_array	RNA			
SM004	SM004_B6_Mock_d4_2	Mus musculus	SM004_B6_Mock_d4_2_Lung	Tissue	SM004-R	SM004_B6_Mock_d4_2_array	RNA			
SM004	SM004_B6_Mock_d4_3	Mus musculus	SM004_B6_Mock_d4_3_Lung	Tissue	SM004-R	SM004_B6_Mock_d4_3_array	RNA			
SM004	SM004_B6_Mock_d4_4	Mus musculus	SM004_B6_Mock_d4_4_Lung	Tissue	SM004-R	SM004_B6_Mock_d4_4_array	RNA			
SM004	SM004_B6_Mock_d7_1	Mus musculus	SM004_B6_Mock_d7_1_Lung	Tissue	SM004-R	SM004_B6_Mock_d7_1_array	RNA			
SM004	SM004_B6_Mock_d7_2	Mus musculus	SM004_B6_Mock_d7_2_Lung	Tissue	SM004-R	SM004_B6_Mock_d7_2_array	RNA			
SM004	SM004_B6_Mock_d7_3	Mus musculus	SM004_B6_Mock_d7_3_Lung	Tissue	SM004-R	SM004_B6_Mock_d7_3_array	RNA			
SM004	SM004_B6_Mock_d7_4	Mus musculus	SM004_B6_Mock_d7_4_Lung	Tissue	SM004-R	SM004_B6_Mock_d7_4_array	RNA			
SM004	SM004_PAI1_MA15_d4_1	Mus musculus	SM004_PAI1_MA15_d4_1_Lung	Tissue	SM004-R	SM004_PAI1_MA15_d4_1_array	RNA			
SM004	SM004_PAI1_MA15_d4_2	Mus musculus	SM004_PAI1_MA15_d4_2_Lung	Tissue	SM004-R	SM004_PAI1_MA15_d4_2_array	RNA			
SM004	SM004_PAI1_MA15_d4_3	Mus musculus	SM004_PAI1_MA15_d4_3_Lung	Tissue	SM004-R	SM004_PAI1_MA15_d4_3_array	RNA			
SM004	SM004_PAI1_MA15_d4_4	Mus musculus	SM004_PAI1_MA15_d4_4_Lung	Tissue	SM004-R	SM004_PAI1_MA15_d4_4_array	RNA			
SM004	SM004_PAI1_MA15_d7_1	Mus musculus	SM004_PAI1_MA15_d7_1_Lung	Tissue	SM004-R	SM004_PAI1_MA15_d7_1_array	RNA			
SM004	SM004_PAI1_MA15_d7_2	Mus musculus	SM004_PAI1_MA15_d7_2_Lung	Tissue	SM004-R	SM004_PAI1_MA15_d7_2_array	RNA			
SM004	SM004_PAI1_MA15_d7_3	Mus musculus	SM004_PAI1_MA15_d7_3_Lung	Tissue	SM004-R	SM004_PAI1_MA15_d7_3_array	RNA			
SM004	SM004_PAI1_Mock_d4_1	Mus musculus	SM004_PAI1_Mock_d4_1_Lung	Tissue	SM004-R	SM004_PAI1_Mock_d4_1_array	RNA			
SM004	SM004_PAI1_Mock_d4_2	Mus musculus	SM004_PAI1_Mock_d4_2_Lung	Tissue	SM004-R	SM004_PAI1_Mock_d4_2_array	RNA			
SM004	SM004_PAI1_Mock_d7_1	Mus musculus	SM004_PAI1_Mock_d7_1_Lung	Tissue	SM004-R	SM004_PAI1_Mock_d7_1_array	RNA			
SM004	SM004_PAI1_Mock_d7_2	Mus musculus	SM004_PAI1_Mock_d7_2_Lung	Tissue	SM004-R	SM004_PAI1_Mock_d7_2_array	RNA			
SM004	SM004_TIMP1_MA15_d4_1	Mus musculus	SM004_TIMP1_MA15_d4_1_Lung	Tissue	SM004-R	SM004_TIMP1_MA15_d4_1_array	RNA			
SM004	SM004_TIMP1_MA15_d4_2	Mus musculus	SM004_TIMP1_MA15_d4_2_Lung	Tissue	SM004-R	SM004_TIMP1_MA15_d4_2_array	RNA			
SM004	SM004_TIMP1_MA15_d4_3	Mus musculus	SM004_TIMP1_MA15_d4_3_Lung	Tissue	SM004-R	SM004_TIMP1_MA15_d4_3_array	RNA			
SM004	SM004_TIMP1_MA15_d4_4	Mus musculus	SM004_TIMP1_MA15_d4_4_Lung	Tissue	SM004-R	SM004_TIMP1_MA15_d4_4_array	RNA			
SM004	SM004_TIMP1_MA15_d7_1	Mus musculus	SM004_TIMP1_MA15_d7_1_Lung	Tissue	SM004-R	SM004_TIMP1_MA15_d7_1_array	RNA			
SM004	SM004_TIMP1_MA15_d7_2	Mus musculus	SM004_TIMP1_MA15_d7_2_Lung	Tissue	SM004-R	SM004_TIMP1_MA15_d7_2_array	RNA			
SM004	SM004_TIMP1_MA15_d7_3	Mus musculus	SM004_TIMP1_MA15_d7_3_Lung	Tissue	SM004-R	SM004_TIMP1_MA15_d7_3_array	RNA			
SM004	SM004_TIMP1_MA15_d7_4	Mus musculus	SM004_TIMP1_MA15_d7_4_Lung	Tissue	SM004-R	SM004_TIMP1_MA15_d7_4_array	RNA			
SM004	SM004_TIMP1_Mock_d4_1	Mus musculus	SM004_TIMP1_Mock_d4_1_Lung	Tissue	SM004-R	SM004_TIMP1_Mock_d4_1_array	RNA			
SM004	SM004_TIMP1_Mock_d4_2	Mus musculus	SM004_TIMP1_Mock_d4_2_Lung	Tissue	SM004-R	SM004_TIMP1_Mock_d4_2_array	RNA			
SM004	SM004_TIMP1_Mock_d7_1	Mus musculus	SM004_TIMP1_Mock_d7_1_Lung	Tissue	SM004-R	SM004_TIMP1_Mock_d7_1_array	RNA			
SM004	SM004_TIMP1_Mock_d7_2	Mus musculus	SM004_TIMP1_Mock_d7_2_Lung	Tissue	SM004-R	SM004_TIMP1_Mock_d7_2_array	RNA			
SM007	SM007_B6_MA15_d2_1	Mus musculus	SM007_B6_MA15_d2_1_Lung	Tissue	SM007-R	SM007_B6_MA15_d2_1_array	RNA			
SM007	SM007_B6_MA15_d2_2	Mus musculus	SM007_B6_MA15_d2_2_Lung	Tissue	SM007-R	SM007_B6_MA15_d2_2_array	RNA			
SM007	SM007_B6_MA15_d2_3	Mus musculus	SM007_B6_MA15_d2_3_Lung	Tissue	SM007-R	SM007_B6_MA15_d2_3_array	RNA			
SM007	SM007_B6_MA15_d4_1	Mus musculus	SM007_B6_MA15_d4_1_Lung	Tissue	SM007-R	SM007_B6_MA15_d4_1_array	RNA			
SM007	SM007_B6_MA15_d4_2	Mus musculus	SM007_B6_MA15_d4_2_Lung	Tissue	SM007-R	SM007_B6_MA15_d4_2_array	RNA			
SM007	SM007_B6_MA15_d7_1	Mus musculus	SM007_B6_MA15_d7_1_Lung	Tissue	SM007-R	SM007_B6_MA15_d7_1_array	RNA			
SM007	SM007_B6_MA15_d7_2	Mus musculus	SM007_B6_MA15_d7_2_Lung	Tissue	SM007-R	SM007_B6_MA15_d7_2_array	RNA			
SM007	SM007_B6_MA15_d7_3	Mus musculus	SM007_B6_MA15_d7_3_Lung	Tissue	SM007-R	SM007_B6_MA15_d7_3_array	RNA			
SM007	SM007_B6_Mock_d2_1	Mus musculus	SM007_B6_Mock_d2_1_Lung	Tissue	SM007-R	SM007_B6_Mock_d2_1_array	RNA			
SM007	SM007_B6_Mock_d2_2	Mus musculus	SM007_B6_Mock_d2_2_Lung	Tissue	SM007-R	SM007_B6_Mock_d2_2_array	RNA			
SM007	SM007_B6_Mock_d2_3	Mus musculus	SM007_B6_Mock_d2_3_Lung	Tissue	SM007-R	SM007_B6_Mock_d2_3_array	RNA			
SM007	SM007_B6_Mock_d4_1	Mus musculus	SM007_B6_Mock_d4_1_Lung	Tissue	SM007-R	SM007_B6_Mock_d4_1_array	RNA			
SM007	SM007_B6_Mock_d4_2	Mus musculus	SM007_B6_Mock_d4_2_Lung	Tissue	SM007-R	SM007_B6_Mock_d4_2_array	RNA			
SM007	SM007_B6_Mock_d4_3	Mus musculus	SM007_B6_Mock_d4_3_Lung	Tissue	SM007-R	SM007_B6_Mock_d4_3_array	RNA			
SM007	SM007_B6_Mock_d7_1	Mus musculus	SM007_B6_Mock_d7_1_Lung	Tissue	SM007-R	SM007_B6_Mock_d7_1_array	RNA			
SM007	SM007_B6_Mock_d7_2	Mus musculus	SM007_B6_Mock_d7_2_Lung	Tissue	SM007-R	SM007_B6_Mock_d7_2_array	RNA			
SM007	SM007_B6_Mock_d7_3	Mus musculus	SM007_B6_Mock_d7_3_Lung	Tissue	SM007-R	SM007_B6_Mock_d7_3_array	RNA			
SM007	SM007_CXCR3_MA15_d2_1	Mus musculus	SM007_CXCR3_MA15_d2_1_Lung	Tissue	SM007-R	SM007_CXCR3_MA15_d2_1_array	RNA			
SM007	SM007_CXCR3_MA15_d2_2	Mus musculus	SM007_CXCR3_MA15_d2_2_Lung	Tissue	SM007-R	SM007_CXCR3_MA15_d2_2_array	RNA			
SM007	SM007_CXCR3_MA15_d2_3	Mus musculus	SM007_CXCR3_MA15_d2_3_Lung	Tissue	SM007-R	SM007_CXCR3_MA15_d2_3_array	RNA			
SM007	SM007_CXCR3_MA15_d4_2	Mus musculus	SM007_CXCR3_MA15_d4_2_Lung	Tissue	SM007-R	SM007_CXCR3_MA15_d4_2_array	RNA			
SM007	SM007_CXCR3_MA15_d4_3	Mus musculus	SM007_CXCR3_MA15_d4_3_Lung	Tissue	SM007-R	SM007_CXCR3_MA15_d4_3_array	RNA			
SM007	SM007_CXCR3_MA15_d7_1	Mus musculus	SM007_CXCR3_MA15_d7_1_Lung	Tissue	SM007-R	SM007_CXCR3_MA15_d7_1_array	RNA			
SM007	SM007_CXCR3_MA15_d7_2	Mus musculus	SM007_CXCR3_MA15_d7_2_Lung	Tissue	SM007-R	SM007_CXCR3_MA15_d7_2_array	RNA			
SM007	SM007_CXCR3_MA15_d7_3	Mus musculus	SM007_CXCR3_MA15_d7_3_Lung	Tissue	SM007-R	SM007_CXCR3_MA15_d7_3_array	RNA			
SM007	SM007_CXCR3_MA15_d7_4	Mus musculus	SM007_CXCR3_MA15_d7_4_Lung	Tissue	SM007-R	SM007_CXCR3_MA15_d7_4_array	RNA			
SM007	SM007_CXCR3_Mock_d2_2	Mus musculus	SM007_CXCR3_Mock_d2_2_Lung	Tissue	SM007-R	SM007_CXCR3_Mock_d2_2_array	RNA			
SM007	SM007_CXCR3_Mock_d2_3	Mus musculus	SM007_CXCR3_Mock_d2_3_Lung	Tissue	SM007-R	SM007_CXCR3_Mock_d2_3_array	RNA			
SM007	SM007_CXCR3_Mock_d4_1	Mus musculus	SM007_CXCR3_Mock_d4_1_Lung	Tissue	SM007-R	SM007_CXCR3_Mock_d4_1_array	RNA			
SM007	SM007_CXCR3_Mock_d4_2	Mus musculus	SM007_CXCR3_Mock_d4_2_Lung	Tissue	SM007-R	SM007_CXCR3_Mock_d4_2_array	RNA			
SM007	SM007_CXCR3_Mock_d7_1	Mus musculus	SM007_CXCR3_Mock_d7_1_Lung	Tissue	SM007-R	SM007_CXCR3_Mock_d7_1_array	RNA			
SM007	SM007_CXCR3_Mock_d7_2	Mus musculus	SM007_CXCR3_Mock_d7_2_Lung	Tissue	SM007-R	SM007_CXCR3_Mock_d7_2_array	RNA			
SM007	SM007_CXCR3_Mock_d7_3	Mus musculus	SM007_CXCR3_Mock_d7_3_Lung	Tissue	SM007-R	SM007_CXCR3_Mock_d7_3_array	RNA			
SM009	SM009_B6_MA15_d4_2	Mus musculus	SM009_B6_MA15_d4_2_Lung	Tissue	SM009-R	SM009_B6_MA15_d4_2_array	RNA			
SM009	SM009_B6_MA15_d4_3	Mus musculus	SM009_B6_MA15_d4_3_Lung	Tissue	SM009-R	SM009_B6_MA15_d4_3_array	RNA			
SM009	SM009_B6_MA15_d4_4	Mus musculus	SM009_B6_MA15_d4_4_Lung	Tissue	SM009-R	SM009_B6_MA15_d4_4_array	RNA			
SM009	SM009_B6_MA15_d7_2	Mus musculus	SM009_B6_MA15_d7_2_Lung	Tissue	SM009-R	SM009_B6_MA15_d7_2_array	RNA			
SM009	SM009_B6_MA15_d7_4	Mus musculus	SM009_B6_MA15_d7_4_Lung	Tissue	SM009-R	SM009_B6_MA15_d7_4_array	RNA			
SM009	SM009_B6_Mock_d4_1	Mus musculus	SM009_B6_Mock_d4_1_Lung	Tissue	SM009-R	SM009_B6_Mock_d4_1_array	RNA			
SM009	SM009_B6_Mock_d4_2	Mus musculus	SM009_B6_Mock_d4_2_Lung	Tissue	SM009-R	SM009_B6_Mock_d4_2_array	RNA			
SM009	SM009_B6_Mock_d7_1	Mus musculus	SM009_B6_Mock_d7_1_Lung	Tissue	SM009-R	SM009_B6_Mock_d7_1_array	RNA			
SM009	SM009_B6_Mock_d7_2	Mus musculus	SM009_B6_Mock_d7_2_Lung	Tissue	SM009-R	SM009_B6_Mock_d7_2_array	RNA			
SM009	SM009_PLAT_MA15_d4_3	Mus musculus	SM009_PLAT_MA15_d4_3_Lung	Tissue	SM009-R	SM009_PLAT_MA15_d4_3_array	RNA			
SM009	SM009_PLAT_MA15_d4_4	Mus musculus	SM009_PLAT_MA15_d4_4_Lung	Tissue	SM009-R	SM009_PLAT_MA15_d4_4_array	RNA			
SM009	SM009_PLAT_MA15_d7_1	Mus musculus	SM009_PLAT_MA15_d7_1_Lung	Tissue	SM009-R	SM009_PLAT_MA15_d7_1_array	RNA			
SM009	SM009_PLAT_MA15_d7_2	Mus musculus	SM009_PLAT_MA15_d7_2_Lung	Tissue	SM009-R	SM009_PLAT_MA15_d7_2_array	RNA			
SM009	SM009_PLAT_MA15_d7_4	Mus musculus	SM009_PLAT_MA15_d7_4_Lung	Tissue	SM009-R	SM009_PLAT_MA15_d7_4_array	RNA			
SM009	SM009_PLAT_Mock_d4_1	Mus musculus	SM009_PLAT_Mock_d4_1_Lung	Tissue	SM009-R	SM009_PLAT_Mock_d4_1_array	RNA			
SM009	SM009_PLAT_Mock_d4_2	Mus musculus	SM009_PLAT_Mock_d4_2_Lung	Tissue	SM009-R	SM009_PLAT_Mock_d4_2_array	RNA			
SM009	SM009_PLAT_Mock_d7_1	Mus musculus	SM009_PLAT_Mock_d7_1_Lung	Tissue	SM009-R	SM009_PLAT_Mock_d7_1_array	RNA			
SM009	SM009_PLAT_Mock_d7_2	Mus musculus	SM009_PLAT_Mock_d7_2_Lung	Tissue	SM009-R	SM009_PLAT_Mock_d7_2_array	RNA			
SM012	SM012_dORF6_10^5_d1_1	Mus musculus	SM012_dORF6_10^5_d1_1_Lung	Tissue	SM012-R	SM012_dORF6_10^5pfu_1d_1_array	RNA			
SM012	SM012_dORF6_10^5_d1_3	Mus musculus	SM012_dORF6_10^5_d1_3_Lung	Tissue	SM012-R	SM012_dORF6_10^5pfu_1d_3_array	RNA			
SM012	SM012_dORF6_10^5_d1_5	Mus musculus	SM012_dORF6_10^5_d1_5_Lung	Tissue	SM012-R	SM012_dORF6_10^5pfu_1d_5_array	RNA			
SM012	SM012_dORF6_10^5_d2_1	Mus musculus	SM012_dORF6_10^5_d2_1_Lung	Tissue	SM012-R	SM012_dORF6_10^5pfu_2d_1_array	RNA			
SM012	SM012_dORF6_10^5_d2_3	Mus musculus	SM012_dORF6_10^5_d2_3_Lung	Tissue	SM012-R	SM012_dORF6_10^5pfu_2d_3_array	RNA			
SM012	SM012_dORF6_10^5_d2_4	Mus musculus	SM012_dORF6_10^5_d2_4_Lung	Tissue	SM012-R	SM012_dORF6_10^5pfu_2d_4_array	RNA			
SM012	SM012_dORF6_10^5_d4_1	Mus musculus	SM012_dORF6_10^5_d4_1_Lung	Tissue	SM012-R	SM012_dORF6_10^5pfu_4d_1_array	RNA			
SM012	SM012_dORF6_10^5_d4_2	Mus musculus	SM012_dORF6_10^5_d4_2_Lung	Tissue	SM012-R	SM012_dORF6_10^5pfu_4d_2_array	RNA			
SM012	SM012_dORF6_10^5_d4_3	Mus musculus	SM012_dORF6_10^5_d4_3_Lung	Tissue	SM012-R	SM012_dORF6_10^5pfu_4d_3_array	RNA			
SM012	SM012_dORF6_10^5_d7_1	Mus musculus	SM012_dORF6_10^5_d7_1_Lung	Tissue	SM012-R	SM012_dORF6_10^5pfu_7d_1_array	RNA			
SM012	SM012_dORF6_10^5_d7_2	Mus musculus	SM012_dORF6_10^5_d7_2_Lung	Tissue	SM012-R	SM012_dORF6_10^5pfu_7d_2_array	RNA			
SM012	SM012_dORF6_10^5_d7_3	Mus musculus	SM012_dORF6_10^5_d7_3_Lung	Tissue	SM012-R	SM012_dORF6_10^5pfu_7d_3_array	RNA			
SM012	SM012_MA15_10^5_d1_2	Mus musculus	SM012_MA15_10^5_d1_2_Lung	Tissue	SM012-R	SM012_MA15_10^5pfu_1d_2_array	RNA			
SM012	SM012_MA15_10^5_d1_3	Mus musculus	SM012_MA15_10^5_d1_3_Lung	Tissue	SM012-R	SM012_MA15_10^5pfu_1d_3_array	RNA			
SM012	SM012_MA15_10^5_d1_4	Mus musculus	SM012_MA15_10^5_d1_4_Lung	Tissue	SM012-R	SM012_MA15_10^5pfu_1d_4_array	RNA			
SM012	SM012_MA15_10^5_d2_1	Mus musculus	SM012_MA15_10^5_d2_1_Lung	Tissue	SM012-R	SM012_MA15_10^5pfu_2d_1_array	RNA			
SM012	SM012_MA15_10^5_d2_3	Mus musculus	SM012_MA15_10^5_d2_3_Lung	Tissue	SM012-R	SM012_MA15_10^5pfu_2d_3_array	RNA			
SM012	SM012_MA15_10^5_d2_5	Mus musculus	SM012_MA15_10^5_d2_5_Lung	Tissue	SM012-R	SM012_MA15_10^5pfu_2d_5_array	RNA			
SM012	SM012_MA15_10^5_d4_1	Mus musculus	SM012_MA15_10^5_d4_1_Lung	Tissue	SM012-R	SM012_MA15_10^5pfu_4d_1_array	RNA			
SM012	SM012_MA15_10^5_d4_2	Mus musculus	SM012_MA15_10^5_d4_2_Lung	Tissue	SM012-R	SM012_MA15_10^5pfu_4d_2_array	RNA			
SM012	SM012_MA15_10^5_d4_3	Mus musculus	SM012_MA15_10^5_d4_3_Lung	Tissue	SM012-R	SM012_MA15_10^5pfu_4d_3_array	RNA			
SM012	SM012_MA15_10^5_d7_1	Mus musculus	SM012_MA15_10^5_d7_1_Lung	Tissue	SM012-R	SM012_MA15_10^5pfu_7d_1_array	RNA			
SM012	SM012_MA15_10^5_d7_2	Mus musculus	SM012_MA15_10^5_d7_2_Lung	Tissue	SM012-R	SM012_MA15_10^5pfu_7d_2_array	RNA			
SM012	SM012_MA15_10^5_d7_3	Mus musculus	SM012_MA15_10^5_d7_3_Lung	Tissue	SM012-R	SM012_MA15_10^5pfu_7d_3_array	RNA			
SM012	SM012_Mock_d1_1	Mus musculus	SM012_Mock_d1_1_Lung	Tissue	SM012-R	SM012_Mock_1d_1_array	RNA			
SM012	SM012_Mock_d1_4	Mus musculus	SM012_Mock_d1_4_Lung	Tissue	SM012-R	SM012_Mock_1d_4_array	RNA			
SM012	SM012_Mock_d1_5	Mus musculus	SM012_Mock_d1_5_Lung	Tissue	SM012-R	SM012_Mock_1d_5_array	RNA			
SM012	SM012_Mock_d2_2	Mus musculus	SM012_Mock_d2_2_Lung	Tissue	SM012-R	SM012_Mock_2d_2_array	RNA			
SM012	SM012_Mock_d2_3	Mus musculus	SM012_Mock_d2_3_Lung	Tissue	SM012-R	SM012_Mock_2d_3_array	RNA			
SM012	SM012_Mock_d2_4	Mus musculus	SM012_Mock_d2_4_Lung	Tissue	SM012-R	SM012_Mock_2d_4_array	RNA			
SM012	SM012_Mock_d4_1	Mus musculus	SM012_Mock_d4_1_Lung	Tissue	SM012-R	SM012_Mock_4d_1_array	RNA			
SM012	SM012_Mock_d4_2	Mus musculus	SM012_Mock_d4_2_Lung	Tissue	SM012-R	SM012_Mock_4d_2_array	RNA			
SM012	SM012_Mock_d4_3	Mus musculus	SM012_Mock_d4_3_Lung	Tissue	SM012-R	SM012_Mock_4d_3_array	RNA			
SM012	SM012_Mock_d7_1	Mus musculus	SM012_Mock_d7_1_Lung	Tissue	SM012-R	SM012_Mock_7d_1_array	RNA			
SM012	SM012_Mock_d7_3	Mus musculus	SM012_Mock_d7_3_Lung	Tissue	SM012-R	SM012_Mock_7d_3_array	RNA			
SM014	SM014_MA15_10^5_d1_1	Mus musculus	SM014_MA15_10^5_d1_1_Lung	Tissue	SM014-R	SM014_MA15_10^5pfu_1d_1_array	RNA			
SM014	SM014_MA15_10^5_d1_2	Mus musculus	SM014_MA15_10^5_d1_2_Lung	Tissue	SM014-R	SM014_MA15_10^5pfu_1d_2_array	RNA			
SM014	SM014_MA15_10^5_d1_3	Mus musculus	SM014_MA15_10^5_d1_3_Lung	Tissue	SM014-R	SM014_MA15_10^5pfu_1d_3_array	RNA			
SM014	SM014_MA15_10^5_d1_4	Mus musculus	SM014_MA15_10^5_d1_4_Lung	Tissue	SM014-R	SM014_MA15_10^5pfu_1d_4_array	RNA			
SM014	SM014_MA15_10^5_d2_1	Mus musculus	SM014_MA15_10^5_d2_1_Lung	Tissue	SM014-R	SM014_MA15_10^5pfu_2d_1_array	RNA			
SM014	SM014_MA15_10^5_d2_2	Mus musculus	SM014_MA15_10^5_d2_2_Lung	Tissue	SM014-R	SM014_MA15_10^5pfu_2d_2_array	RNA			
SM014	SM014_MA15_10^5_d2_3	Mus musculus	SM014_MA15_10^5_d2_3_Lung	Tissue	SM014-R	SM014_MA15_10^5pfu_2d_3_array	RNA			
SM014	SM014_MA15_10^5_d2_4	Mus musculus	SM014_MA15_10^5_d2_4_Lung	Tissue	SM014-R	SM014_MA15_10^5pfu_2d_4_array	RNA			
SM014	SM014_MA15_10^5_d4_1	Mus musculus	SM014_MA15_10^5_d4_1_Lung	Tissue	SM014-R	SM014_MA15_10^5pfu_4d_1_array	RNA			
SM014	SM014_MA15_10^5_d4_2	Mus musculus	SM014_MA15_10^5_d4_2_Lung	Tissue	SM014-R	SM014_MA15_10^5pfu_4d_2_array	RNA			
SM014	SM014_MA15_10^5_d4_3	Mus musculus	SM014_MA15_10^5_d4_3_Lung	Tissue	SM014-R	SM014_MA15_10^5pfu_4d_3_array	RNA			
SM014	SM014_MA15_10^5_d4_4	Mus musculus	SM014_MA15_10^5_d4_4_Lung	Tissue	SM014-R	SM014_MA15_10^5pfu_4d_4_array	RNA			
SM014	SM014_MA15_10^5_d7_1	Mus musculus	SM014_MA15_10^5_d7_1_Lung	Tissue	SM014-R	SM014_MA15_10^5pfu_7d_1_array	RNA			
SM014	SM014_MA15_10^5_d7_2	Mus musculus	SM014_MA15_10^5_d7_2_Lung	Tissue	SM014-R	SM014_MA15_10^5pfu_7d_2_array	RNA			
SM014	SM014_MA15_10^5_d7_4	Mus musculus	SM014_MA15_10^5_d7_4_Lung	Tissue	SM014-R	SM014_MA15_10^5pfu_7d_4_array	RNA			
SM014	SM014_MA15_10^5_d7_5	Mus musculus	SM014_MA15_10^5_d7_5_Lung	Tissue	SM014-R		RNA			
SM014	SM014_Mock_d1_1	Mus musculus	SM014_Mock_d1_1_Lung	Tissue	SM014-R		RNA			
SM014	SM014_Mock_d1_2	Mus musculus	SM014_Mock_d1_2_Lung	Tissue	SM014-R	SM014_Mock_1d_2_array	RNA			
SM014	SM014_Mock_d1_3	Mus musculus	SM014_Mock_d1_3_Lung	Tissue	SM014-R	SM014_Mock_1d_3_array	RNA			
SM014	SM014_Mock_d2_1	Mus musculus	SM014_Mock_d2_1_Lung	Tissue	SM014-R	SM014_Mock_2d_1_array	RNA			
SM014	SM014_Mock_d2_2	Mus musculus	SM014_Mock_d2_2_Lung	Tissue	SM014-R	SM014_Mock_2d_2_array	RNA			
SM014	SM014_Mock_d2_3	Mus musculus	SM014_Mock_d2_3_Lung	Tissue	SM014-R	SM014_Mock_2d_3_array	RNA			
SM014	SM014_Mock_d4_1	Mus musculus	SM014_Mock_d4_1_Lung	Tissue	SM014-R	SM014_Mock_4d_1_array	RNA			
SM014	SM014_Mock_d4_2	Mus musculus	SM014_Mock_d4_2_Lung	Tissue	SM014-R	SM014_Mock_4d_2_array	RNA			
SM014	SM014_Mock_d4_3	Mus musculus	SM014_Mock_d4_3_Lung	Tissue	SM014-R	SM014_Mock_4d_3_array	RNA			
SM014	SM014_Mock_d7_1	Mus musculus	SM014_Mock_d7_1_Lung	Tissue	SM014-R	SM014_Mock_7d_1_array	RNA			
SM014	SM014_Mock_d7_2	Mus musculus	SM014_Mock_d7_2_Lung	Tissue	SM014-R	SM014_Mock_7d_2_array	RNA			
SM014	SM014_Mock_d7_3	Mus musculus	SM014_Mock_d7_3_Lung	Tissue	SM014-R	SM014_Mock_7d_3_array	RNA			
SM014	SM014_nsp16_10^5_d1_2	Mus musculus	SM014_nsp16_10^5_d1_2_Lung	Tissue	SM014-R	SM014_nsp16_10^5pfu_1d_2_array	RNA			
SM014	SM014_nsp16_10^5_d1_3	Mus musculus	SM014_nsp16_10^5_d1_3_Lung	Tissue	SM014-R	SM014_nsp16_10^5pfu_1d_3_array	RNA			
SM014	SM014_nsp16_10^5_d1_4	Mus musculus	SM014_nsp16_10^5_d1_4_Lung	Tissue	SM014-R	SM014_nsp16_10^5pfu_1d_4_array	RNA			
SM014	SM014_nsp16_10^5_d2_1	Mus musculus	SM014_nsp16_10^5_d2_1_Lung	Tissue	SM014-R	SM014_nsp16_10^5pfu_2d_1_array	RNA			
SM014	SM014_nsp16_10^5_d2_2	Mus musculus	SM014_nsp16_10^5_d2_2_Lung	Tissue	SM014-R	SM014_nsp16_10^5pfu_2d_2_array	RNA			
SM014	SM014_nsp16_10^5_d2_3	Mus musculus	SM014_nsp16_10^5_d2_3_Lung	Tissue	SM014-R	SM014_nsp16_10^5pfu_2d_3_array	RNA			
SM014	SM014_nsp16_10^5_d2_4	Mus musculus	SM014_nsp16_10^5_d2_4_Lung	Tissue	SM014-R	SM014_nsp16_10^5pfu_2d_4_array	RNA			
SM014	SM014_nsp16_10^5_d4_1	Mus musculus	SM014_nsp16_10^5_d4_1_Lung	Tissue	SM014-R	SM014_nsp16_10^5pfu_4d_1_array	RNA			
SM014	SM014_nsp16_10^5_d4_3	Mus musculus	SM014_nsp16_10^5_d4_3_Lung	Tissue	SM014-R	SM014_nsp16_10^5pfu_4d_3_array	RNA			
SM014	SM014_nsp16_10^5_d4_4	Mus musculus	SM014_nsp16_10^5_d4_4_Lung	Tissue	SM014-R	SM014_nsp16_10^5pfu_4d_4_array	RNA			
SM014	SM014_nsp16_10^5_d7_1	Mus musculus	SM014_nsp16_10^5_d7_1_Lung	Tissue	SM014-R	SM014_nsp16_10^5pfu_7d_1_array	RNA			
SM014	SM014_nsp16_10^5_d7_2	Mus musculus	SM014_nsp16_10^5_d7_2_Lung	Tissue	SM014-R	SM014_nsp16_10^5pfu_7d_2_array	RNA			
SM014	SM014_nsp16_10^5_d7_3	Mus musculus	SM014_nsp16_10^5_d7_3_Lung	Tissue	SM014-R	SM014_nsp16_10^5pfu_7d_3_array	RNA			
SM014	SM014_nsp16_10^5_d7_4	Mus musculus	SM014_nsp16_10^5_d7_4_Lung	Tissue	SM014-R	SM014_nsp16_10^5pfu_7d_4_array	RNA			
SM015	SM015_B6_MA15_d4_1	Mus musculus	SM015_B6_MA15_d4_1_Lung	Tissue	SM015-R		RNA			
SM015	SM015_B6_MA15_d4_2	Mus musculus	SM015_B6_MA15_d4_2_Lung	Tissue	SM015-R	SM015_B6_MA15_d4_2_array	RNA			
SM015	SM015_B6_MA15_d7_1	Mus musculus	SM015_B6_MA15_d7_1_Lung	Tissue	SM015-R	SM015_B6_MA15_d7_1_array	RNA			
SM015	SM015_B6_MA15_d7_2	Mus musculus	SM015_B6_MA15_d7_2_Lung	Tissue	SM015-R	SM015_B6_MA15_d7_2_array	RNA			
SM015	SM015_B6_MA15_d7_3	Mus musculus	SM015_B6_MA15_d7_3_Lung	Tissue	SM015-R	SM015_B6_MA15_d7_3_array	RNA			
SM015	SM015_B6_Mock_d4_1	Mus musculus	SM015_B6_Mock_d4_1_Lung	Tissue	SM015-R	SM015_B6_Mock_d4_1_array	RNA			
SM015	SM015_B6_Mock_d4_2	Mus musculus	SM015_B6_Mock_d4_2_Lung	Tissue	SM015-R	SM015_B6_Mock_d4_2_array	RNA			
SM015	SM015_B6_Mock_d7_1	Mus musculus	SM015_B6_Mock_d7_1_Lung	Tissue	SM015-R	SM015_B6_Mock_d7_1_array	RNA			
SM015	SM015_B6_Mock_d7_2	Mus musculus	SM015_B6_Mock_d7_2_Lung	Tissue	SM015-R	SM015_B6_Mock_d7_2_array	RNA			
SM015	SM015_B6_Mock_d7_3	Mus musculus	SM015_B6_Mock_d7_3_Lung	Tissue	SM015-R	SM015_B6_Mock_d7_3_array	RNA			
SM015	SM015_Tnfrsf1a/1b_MA15_d4_1	Mus musculus	SM015_Tnfrsf1a/1b_MA15_d4_1_Lung	Tissue	SM015-R	SM015_Tnfrsf1a/1b_MA15_d4_1_array	RNA			
SM015	SM015_Tnfrsf1a/1b_MA15_d4_2	Mus musculus	SM015_Tnfrsf1a/1b_MA15_d4_2_Lung	Tissue	SM015-R	SM015_Tnfrsf1a/1b_MA15_d4_2_array	RNA			
SM015	SM015_Tnfrsf1a/1b_MA15_d7_1	Mus musculus	SM015_Tnfrsf1a/1b_MA15_d7_1_Lung	Tissue	SM015-R	SM015_Tnfrsf1a/1b_MA15_d7_1_array	RNA			
SM015	SM015_Tnfrsf1a/1b_MA15_d7_2	Mus musculus	SM015_Tnfrsf1a/1b_MA15_d7_2_Lung	Tissue	SM015-R	SM015_Tnfrsf1a/1b_MA15_d7_2_array	RNA			
SM015	SM015_Tnfrsf1a/1b_MA15_d7_3	Mus musculus	SM015_Tnfrsf1a/1b_MA15_d7_3_Lung	Tissue	SM015-R	SM015_Tnfrsf1a/1b_MA15_d7_3_array	RNA			
SM015	SM015_Tnfrsf1a/1b_Mock_d4_1	Mus musculus	SM015_Tnfrsf1a/1b_Mock_d4_1_Lung	Tissue	SM015-R	SM015_Tnfrsf1a/1b_Mock_d4_1_array	RNA			
SM015	SM015_Tnfrsf1a/1b_Mock_d4_2	Mus musculus	SM015_Tnfrsf1a/1b_Mock_d4_2_Lung	Tissue	SM015-R	SM015_Tnfrsf1a/1b_Mock_d4_2_array	RNA			
SM015	SM015_Tnfrsf1a/1b_Mock_d7_1	Mus musculus	SM015_Tnfrsf1a/1b_Mock_d7_1_Lung	Tissue	SM015-R	SM015_Tnfrsf1a/1b_Mock_d7_1_array	RNA			
SM015	SM015_Tnfrsf1a/1b_Mock_d7_2	Mus musculus	SM015_Tnfrsf1a/1b_Mock_d7_2_Lung	Tissue	SM015-R	SM015_Tnfrsf1a/1b_Mock_d7_2_array	RNA			
SM015	SM015_Tnfrsf1a/1b_Mock_d7_3	Mus musculus	SM015_Tnfrsf1a/1b_Mock_d7_3_Lung	Tissue	SM015-R	SM015_Tnfrsf1a/1b_Mock_d7_3_array	RNA			
SM019	SM019_B6_MA15_d4_1	Mus musculus	SM019_B6_MA15_d4_1_Lung	Tissue	SM019-R	SM019_B6_MA15_d4_1_array	RNA			
SM019	SM019_B6_MA15_d4_2	Mus musculus	SM019_B6_MA15_d4_2_Lung	Tissue	SM019-R	SM019_B6_MA15_d4_2_array	RNA			
SM019	SM019_B6_MA15_d4_3	Mus musculus	SM019_B6_MA15_d4_3_Lung	Tissue	SM019-R	SM019_B6_MA15_d4_3_array	RNA			
SM019	SM019_B6_MA15_d7_1	Mus musculus	SM019_B6_MA15_d7_1_Lung	Tissue	SM019-R	SM019_B6_MA15_d7_1_array	RNA			
SM019	SM019_B6_MA15_d7_2	Mus musculus	SM019_B6_MA15_d7_2_Lung	Tissue	SM019-R	SM019_B6_MA15_d7_2_array	RNA			
SM019	SM019_B6_MA15_d7_3	Mus musculus	SM019_B6_MA15_d7_3_Lung	Tissue	SM019-R	SM019_B6_MA15_d7_3_array	RNA			
SM019	SM019_B6_Mock_d4_1	Mus musculus	SM019_B6_Mock_d4_1_Lung	Tissue	SM019-R	SM019_B6_Mock_d4_1_array	RNA			
SM019	SM019_B6_Mock_d4_2	Mus musculus	SM019_B6_Mock_d4_2_Lung	Tissue	SM019-R	SM019_B6_Mock_d4_2_array	RNA			
SM019	SM019_B6_Mock_d4_3	Mus musculus	SM019_B6_Mock_d4_3_Lung	Tissue	SM019-R	SM019_B6_Mock_d4_3_array	RNA			
SM019	SM019_B6_Mock_d7_1	Mus musculus	SM019_B6_Mock_d7_1_Lung	Tissue	SM019-R	SM019_B6_Mock_d7_1_array	RNA			
SM019	SM019_B6_Mock_d7_2	Mus musculus	SM019_B6_Mock_d7_2_Lung	Tissue	SM019-R	SM019_B6_Mock_d7_2_array	RNA			
SM019	SM019_B6_Mock_d7_3	Mus musculus	SM019_B6_Mock_d7_3_Lung	Tissue	SM019-R	SM019_B6_Mock_d7_3_array	RNA			
SM019	SM019_Tnfrsf1b_MA15_d4_1	Mus musculus	SM019_Tnfrsf1b_MA15_d4_1_Lung	Tissue	SM019-R	SM019_Tnfrsf1b_MA15_d4_1_array	RNA			
SM019	SM019_Tnfrsf1b_MA15_d4_2	Mus musculus	SM019_Tnfrsf1b_MA15_d4_2_Lung	Tissue	SM019-R	SM019_Tnfrsf1b_MA15_d4_2_array	RNA			
SM019	SM019_Tnfrsf1b_MA15_d4_3	Mus musculus	SM019_Tnfrsf1b_MA15_d4_3_Lung	Tissue	SM019-R	SM019_Tnfrsf1b_MA15_d4_3_array	RNA			
SM019	SM019_Tnfrsf1b_MA15_d7_2	Mus musculus	SM019_Tnfrsf1b_MA15_d7_2_Lung	Tissue	SM019-R	SM019_Tnfrsf1b_MA15_d7_2_array	RNA			
SM019	SM019_Tnfrsf1b_MA15_d7_3	Mus musculus	SM019_Tnfrsf1b_MA15_d7_3_Lung	Tissue	SM019-R	SM019_Tnfrsf1b_MA15_d7_3_array	RNA			
SM019	SM019_Tnfrsf1b_Mock_d4_1	Mus musculus	SM019_Tnfrsf1b_Mock_d4_1_Lung	Tissue	SM019-R	SM019_Tnfrsf1b_Mock_d4_1_array				
SM019	SM019_Tnfrsf1b_Mock_d4_2	Mus musculus	SM019_Tnfrsf1b_Mock_d4_2_Lung	Tissue	SM019-R	SM019_Tnfrsf1b_Mock_d4_2_array				
SM019	SM019_Tnfrsf1b_Mock_d4_3	Mus musculus	SM019_Tnfrsf1b_Mock_d4_3_Lung	Tissue	SM019-R	SM019_Tnfrsf1b_Mock_d4_3_array				
SM019	SM019_Tnfrsf1b_Mock_d7_2	Mus musculus	SM019_Tnfrsf1b_Mock_d7_2_Lung	Tissue	SM019-R	SM019_Tnfrsf1b_Mock_d7_2_array				
SM019	SM019_Tnfrsf1b_Mock_d7_3	Mus musculus	SM019_Tnfrsf1b_Mock_d7_3_Lung	Tissue	SM019-R	SM019_Tnfrsf1b_Mock_d7_3_array				
SM020	SM020_B6_MA15_d4_1	Mus musculus	SM020_B6_MA15_d4_1_Lung	Tissue	SM020-R	SM020_B6_MA15_d4_1_array				
SM020	SM020_B6_MA15_d4_2	Mus musculus	SM020_B6_MA15_d4_2_Lung	Tissue	SM020-R	SM020_B6_MA15_d4_2_array				
SM020	SM020_B6_MA15_d7_1	Mus musculus	SM020_B6_MA15_d7_1_Lung	Tissue	SM020-R	SM020_B6_MA15_d7_1_array				
SM020	SM020_B6_MA15_d7_2	Mus musculus	SM020_B6_MA15_d7_2_Lung	Tissue	SM020-R	SM020_B6_MA15_d7_2_array				
SM020	SM020_B6_MA15_d7_3	Mus musculus	SM020_B6_MA15_d7_3_Lung	Tissue	SM020-R	SM020_B6_MA15_d7_3_array				
SM020	SM020_B6_Mock_d4_1	Mus musculus	SM020_B6_Mock_d4_1_Lung	Tissue	SM020-R	SM020_B6_Mock_d4_1_array				
SM020	SM020_B6_Mock_d4_2	Mus musculus	SM020_B6_Mock_d4_2_Lung	Tissue	SM020-R	SM020_B6_Mock_d4_2_array				
SM020	SM020_B6_Mock_d7_1	Mus musculus	SM020_B6_Mock_d7_1_Lung	Tissue	SM020-R	SM020_B6_Mock_d7_1_array				
SM020	SM020_B6_Mock_d7_2	Mus musculus	SM020_B6_Mock_d7_2_Lung	Tissue	SM020-R	SM020_B6_Mock_d7_2_array				
SM020	SM020_B6_Mock_d7_3	Mus musculus	SM020_B6_Mock_d7_3_Lung	Tissue	SM020-R	SM020_B6_Mock_d7_3_array				
SM020	SM020_ppp1r14c_MA15_d4_1	Mus musculus	SM020_ppp1r14c_MA15_d4_1_Lung	Tissue	SM020-R	SM020_ppp1r14c_MA15_d4_1_array				
SM020	SM020_ppp1r14c_MA15_d4_2	Mus musculus	SM020_ppp1r14c_MA15_d4_2_Lung	Tissue	SM020-R	SM020_ppp1r14c_MA15_d4_2_array				
SM020	SM020_ppp1r14c_MA15_d7_2	Mus musculus	SM020_ppp1r14c_MA15_d7_2_Lung	Tissue	SM020-R	SM020_ppp1r14c_MA15_d7_2_array				
SM020	SM020_ppp1r14c_MA15_d7_3	Mus musculus	SM020_ppp1r14c_MA15_d7_3_Lung	Tissue	SM020-R	SM020_ppp1r14c_MA15_d7_3_array				
SM020	SM020_ppp1r14c_Mock_d4_1	Mus musculus	SM020_ppp1r14c_Mock_d4_1_Lung	Tissue	SM020-R	SM020_ppp1r14c_Mock_d4_1_array				
SM020	SM020_ppp1r14c_Mock_d4_2	Mus musculus	SM020_ppp1r14c_Mock_d4_2_Lung	Tissue	SM020-R	SM020_ppp1r14c_Mock_d4_2_array				
SM020	SM020_ppp1r14c_Mock_d7_1	Mus musculus	SM020_ppp1r14c_Mock_d7_1_Lung	Tissue	SM020-R	SM020_ppp1r14c_Mock_d7_1_array				
SM020	SM020_ppp1r14c_Mock_d7_2	Mus musculus	SM020_ppp1r14c_Mock_d7_2_Lung	Tissue	SM020-R	SM020_ppp1r14c_Mock_d7_2_array				
SM020	SM020_ppp1r14c_Mock_d7_3	Mus musculus	SM020_ppp1r14c_Mock_d7_3_Lung	Tissue	SM020-R	SM020_ppp1r14c_Mock_d7_3_array				

**Table 5:** Description of viral strains

**Virus Strain**	**Virus Description**	**Representative Accessions** *	**Sequence Alterations**	**Description Source**
A/California/04/2009 (H1N1)	A/California/04/2009 (H1N1) - An isolate of the 2009 influenza pandemic	1=FJ966079, 2=FJ966080, 3=FJ966081, 4=FJ966082, 5=FJ966083, 6=FJ966084, 7=FJ969513, 8=FJ966086	None Specified	Josset *et al.*, 2012 ^6^
MERS-CoV	MERS-CoV - A member of the betacoronavirus genus isolated from patients in the Middle East that causes severe acute respitory syndrome (SARS) fomerly known as vHuman coronavirus EMC 2012 (HCoV-EMC) [PMID: 23631916]	NC_019843	None Specified	Josset *et al.*, 2013 ^5^
A/Vietnam/1203/2004 (H5N1)	A/Vietnam/1203/2004 (H5N1)–Highly pathogenic avian influenza (HPAI) H5N1	1=AY651719, 2=AY651665, 3=AY651611, 4=AY651334, 5=AY651499, 6=AY651447, 7=AY651388, 8=AY651553,	None Specified	Li *et al.*, 2011 ^4^
A/Netherlands/602/2009 (H1N1)	A/Netherlands/602/2009 (H1N1) - An isolate of the 2009 influenza pandemic	1=CY046940, 2=CY046941, 3=CY046942, 4=CY039527, 5=CY046943, 6=CY039528, 7=CY046944, 8=CY046945	None Specified	Mitchell *et al.*, 2013 ^11^
A/New Jersey/8/1976 (H1N1)	A/New Jersey/8/76 (H1N1) - Isolated from throat swab material of a young military recruit during an outbreak at Fort Dix, NJ, in 1976.	1=CY130125, 2=CY130124, 3=CY130123, 4=CY130118, 5=CY130121, 6=CY130120, 7=CY130119, 8=CY130122	None Specified	Josset *et al.*, 2012 ^6^
A/Mexico/4482/2009 (H1N1)	A/Mexico/4482/2009 (H1N1) - An Isolate from the 2009 H1N1 pandemic that caused severe respiratory illness in humans	1=CY098505, 2=GQ149675, 3=GQ149676, 4=GQ149671, 5=GQ149673, 6=GQ149675, 7=GQ149669, 8=GQ379818,	None Specified	Josset *et al.*, 2012 ^6^
Reconstructed 1918 (H1N1)	Reconstructed 1918 (H1N1) - The r1918 influenza virus segments were derived from the published sequences of strains A/South Carolina/1/18 (H1N1) HA, and A/Brevig Mission/1/1918 (H1N1) NA, M, NS, NP, PA, PB1 and PB2. The 1918 recombinant viruses also contained the 5′ and 3′ non-coding regions from strain A/WSN/1933 (H1N1).	1=DQ208309, 2=DQ208310 3=DQ208311, 4=AF117241, 5=AY744935, 6=AF250356, 7=AY130766, 8=AF333238	5′ and 3′ non-coding regions were supplied by strain A/WSN/1933 (H1N1)	Kash *et al.*, 2006 ^23^
A/Brisbane/59/2007 (H1N1)	A/Brisbane/59/2007 (H1N1) - H1N1 used in trivalent vaccine from 2008-2010 in Northern Hemisphere	1=CY058484, 2=CY058485, 3=CY058486, 4=CY058487, 5=CY058488, 6=CY058489, 7=CY058490, 8=CY058491	None Specified	Josset *et al.*, 2012 ^6^
A/Vietnam/1203-CIP048_RG1 (H5N1)	A/Vietnam/1203-CIP048_RG1 (H5N1) - The H5N1 VN1203-HAavir mutant virus harbors an altered multi-basic cleavage site – a virulence factor important for expanded tissue range – and exhibits restricted systemic viral spread due to limited HA susceptibility to furin protease activity.	Parent Strain: A/Vietnam/1203/2004 (H5N1)	hemagglutinin (HA) surface protein poly-basic cleavage site (RERRRKKR↓G) was mutated to (RETR↓G)	Tchitchek *et al.*, 2013 ^8^
A/Vietnam/1203-CIP048_RG2 (H5N1)	A/Vietnam/1203-CIP048_RG2 (H5N1) - The H5N1 VN1203-PB2627E mutant possesses an amino acid substitution (Lys-to-Glu) at position 627 in the PB2 polymerase subunit. This mutation is known to confer increased polymerase activity in mammalian cells, and also modulates anti-viral activity, apoptosis, and viral clearance.	Parent Strain: A/Vietnam/1203/2004 (H5N1)	PB2: K627E	Tchitchek *et al.*, 2013 ^8^
A/Vietnam/1203-CIP048_RG3 (H5N1)	A/Vietnam/1203-CIP048_RG3 (H5N1) - H5N1 VN1203-PB1F2del mutant lacks expression of the PB1-F2 protein, potentially impacting an array of functions. PB1-F2 is a viral pathogenicity factor in mammals and birds, and has been shown to modulate viral polymerase activity, enhance lung inflammation, modulate innate immune responses, and demonstrate pro-apoptotic activity.	Parent Strain: A/Vietnam/1203/2004 (H5N1)	PB1 gene segment: T120C, C153G, G291A (all stops)	Tchitchek *et al.*, 2013 ^8^
A/Vietnam/1203-CIP048_RG4 (H5N1)	A/Vietnam/1203-CIP048_RG4 (H5N1) - H5N1 VN1203-NS1trunc mutant virus produces a 91 amino acid C-terminal truncation in the effector domain of the NS1 host response antagonist protein. The NS1 protein inhibits RIG-I activation and cellular mRNA processing, and also promotes PI3K activation. The truncation results in the loss of the NS1 nuclear localization signal, a PI3K-binding motif, and binding domains.	Parent Strain: A/Vietnam/1203/2004 (H5N1)	NS1: 124(Stop), NS Gene segment: T400A	Tchitchek *et al.*, 2013 ^8^
MA1-A/ California/04/2009 (H1N1)	MA1-A/ California/04/2009 (H1N1) - Mouse adapted MA-CA/04 contains five genetic mutations (in HA, G155E, S183P, and D222G; in PB2, E158G; and in NP, D101G) and is associated with a dramatic increase in virulence in mice	Parent Strain: A/California/04/2009 (H1N1)	PB2: E158GPA: L295PHA: G155E S183P R221R D222GNP: D101G	Ilyushina NA *et al.*, 2010 ^27^
A/Aichi/02/68 (HA, NA) x A/Puerto Rico/8/34 (X31)	A/Aichi/02/68 (HA, NA) x A/Puerto Rico/8/34 (HKx31) - A reassortant virus that combines the surface HA and NA from an H3N2 virus (A/HK/x-31) with the 6 internal genes of PR8. Despite containing the same replication proteins as PR8, it is significantly less pathogenic in mice.	1=CY009451, 2=CY009450, 3=CY009449, 4=AB295605, 5=CY009447, 6=AB295606, 7=CY009445, 8=CY009448	None Specified	Askovich *et al.* ^9^
A/Puerto Rico/8/1934 (H1N1)	A/Puerto Rico/8/34 (H1N1) - The H1N1 influenza virus A/Puerto Rico/34 (PR8) has been adapted to grow efficiently in the airways of mice, and is lethal at relatively low doses.	1=CY009451, 2=CY009450, 3=CY009449, 4=CY009444, 5=CY009447, 6=CY009446, 7=CY009445, 8=CY009448	None Specified	Askovich *et al.*, 2013 ^9^
A/Vietnam/1203/04 (HA Vn1203, NA) x A/Puerto Rico/8/34 (VN6+2)	A/Vietnam/1203/04 (HA Vn1203, NA) x A/Puerto Rico/8/34 (VN6+2) - This H5N1 reassortant virus, designated rg/VN1203(6+2) contains the 6 internal genes from PR8 and the HA and NA from VN1203. The HA of this virus has had its polybasic cleavage site removed to restrict replication to the lung and airway epithelium. This virus is more pathogenic than PR8 and serves as a murine model for infection with a highly pathogenic strain.	1=CY009451, 2=CY009450, 3=CY009449, 4=AY651334, 5=CY009447, 6=AY651447, 7=CY009445, 8=CY009448	None Specified	Askovich *et al.*, 2013 ^9^
icSARS Bat SRBD	SARS Bat SRBD - A SARS-CoV like virus isolated from bats that contains the receptor binding domain RBD from wild type SARS-CoV Urbani	FJ211859	Bat-SCoV RBD (amino acid 323–505) was replaced with the SARS-CoV RBD (amino acid 319–518): FJ211860	Becker, M.M., 2008 ^28^
icSARS CoV Urbani	icSARS CoV Urbani - Wild type infectious clone of SARS-CoV derived from genome fragments that were amplified in E. coli, ligated, and purified prior to *in vitro* transcription reactions to synthesize full length genomic RNA which were transfected into VeroE6 cells.	NC_004718*	Numerous alterations were made, see publications for details. Breifly, alterations included removal of one endogenous BglI site and the addition of three. Nucleotide alterations included an A to G at position 6460, T to C at position 14178, T to C at position 15740, C to T at position 19814, A to G at position 20528, and a T to C at position 20555.	Yount, B. *et al.*, 2003 ^30^
icSARS dORF6	icSARS dORF6 - infectious clone of a mutant Urbani SARS CoV in which the ORF6 protein is not expressed	Parent Strain icSARS CoV Urbani	Entire ORF6 deleted (nucleotides 27,074-27265)	Sims *et al.*, 2013 ^12^
SARS CoV MA15	SARS CoV MA15 - The mouse-adapted SARS-CoV (MA15)	FJ882957		Gralinski *et al.*, 2013 ^7^
icSARS dNSP16	icSARS dNSP16 - Genetic modification of non-structural protein 16 in SARS-CoV was shown to give rise to enhanced susceptibility to type I and III interferon responses	Parent Strain icSARS CoV Urbani	NSP16: K46A, K170A, D130A	Menachery *et al.*, 2014 ^13^

*Strains used were not directly sequenced. Accessions given are representative sequences with necessary details for recreating the strain used given in the ‘Sequence Alterations’ column.

**Table 6:** Description of host animal strains and cell lines

**Host Species (Common)**	**Strain/Line (full name)**	**Description**
Mouse	C57BL/6J (stock no. 000664)	C57BL/6 is the most widely used inbred strain. It is commonly used as a general purpose strain and background strain for the generation of congenics carrying both spontaneous and induced mutations.
Mouse	IDO1: B6.129-Ido1tm1Alm/J (stock no. 005867)	In contrast to wild-type, anti-proliferative treatments (CTLA4-Ig, IFNalpha, or CpG-ODN) do not suppress T cell expansion both *in vivo* and *in vitro*.
Mouse	Tnfrsf1b: B6.129S2-Tnfrsf1btm1Mwm/J (stock no. 002620)	Homozygous mutant mice show normal T-cell development and activity, but are resistant to TNF-induced cell death. Subcutaneous injections of TNF into homozygotes elicit much less tissue necrosis.
Mouse	ppp1r14c: B6;129S6-Ppp1r14ctm1Uhl/J (stock no. 013041)	No gene product (mRNA) is detected by RT-PCR analysis of brain tissue from homozygotes. Homozygotes exhibit increased phosphatase 1 activity in the thalamus.
Mouse	Tnfrsf1a/1b: B6.129S7-Tnfrsf1btm1Imx/J (stock no. 003243)	Double homozygous mutant mice fail to bind TNF.
Mouse	PLAT: PLAT−/− (stock no. 002508)	Pulmonary clot lysis is 21% that of normal wildtype siblings. Endotoxin induced venous thrombosis is increased over that seen in normal wildtype siblings. Fibrin dissolution by PLAT-deficient macrophages is unaffected.
Mouse	Serpine1 (PAI1): Serpine1−/− (stock no. 002507)	Compared to wildtype mice, pulmonary clot lysis is increased in the heterozygote and further increased in the homozygote. Endotoxin induced venous thrombosis is decreased compared to wildtype mice. Thus, disruption of the PAI-1 (Serpine1) gene induces a mild hyperfibrinolytic state.
Mouse	TIMP1: B6.129S4-Timp1tm1Pds/J (stock no. 006243)	No endogenous transcript is detected in lung tissue from affected mice by Northern blot analysis. Homozygous mice have increased resistance to corneal and pulmonary infection with P. aeruginosa, and have altered immune, hematopoietic, and vascular permeability in bleomycin-induced lung injury trials.
Mouse	BALB/c	General purpose model used to study numerous biological processes including infectious disease
Mouse	CXCR3: B6.129P2-Cxcr3tm1Dgen/J (stock no. 005796)	‘Changes that may be related to genotype: Glucose level, elevated, hemizygous mutant males.’ (This targeted mutant was created and characterized by Deltagen, Inc)
Human	Human Airway Epithelial (HAE) cells	Human airway epithelium cultures (HAE) resemble *in vivo* pseudo- stratified mucociliary epithelium
Human	Calu-3 cells	Human lung adenocarcinoma derived from a caucasian male
Human	Calu-3 cells (clone 2B5)	Sub-population of Calu3 cells that express high levels of SARS-CoV cellular receptor, angiotensin converting enzyme 2 (ACE2)
